# Meeting the Global Target in Reproductive, Maternal, Newborn, and Child Health Care Services in Low- and Middle-Income Countries

**DOI:** 10.9745/GHSP-D-20-00097

**Published:** 2020-12-23

**Authors:** Md. Mehedi Hasan, Ricardo J. Soares Magalhaes, Saifuddin Ahmed, Sayem Ahmed, Tuhin Biswas, Yaqoot Fatima, Md. Saimul Islam, Md. Shahadut Hossain, Abdullah A. Mamun

**Affiliations:** a Institute for Social Science Research, The University of Queensland, Indooroopilly, Australia.; b The Australian Research Council Centre of Excellence for Children and Families over the Life Course, The University of Queensland, Indooroopilly, Australia.; c Spatial Epidemiology Laboratory, School of Veterinary Science, The University of Queensland, Gatton, Australia.; d Children’s Health and Environment Program, Child Health Research Centre, The University of Queensland, South Brisbane, Australia.; e Department of Population, Family and Reproductive Health, Johns Hopkins Bloomberg School of Public Health, Baltimore, MD, USA.; f Bill and Melinda Gates Institute for Population and Reproductive Health, Johns Hopkins Bloomberg School of Public Health, Baltimore, MD, USA.; g Health Economics and Policy Research Group, Department of Learning, Informatics, Management and Ethics, Karolinska Institutet, Stockholm, Sweden.; h Department of Tropical Disease Biology, Liverpool School of Tropical Medicine, Liverpool, United Kingdom.; i Health Systems and Population Studies Division, International Centre for Diarrhoeal Disease Research, Bangladesh, Dhaka, Bangladesh.; j Centre for Rural and Remote Health, James Cook University, Mount Isa, Australia.; k Department of Statistics, University of Rajshahi, Rajshahi, Bangladesh.; l Department of Statistics, College of Business & Economics, United Arab Emirates University, United Arab Emirates.

## Abstract

**Introduction:**

Improving reproductive, maternal, newborn, and child health (RMNCH) care services is imperative for reducing maternal and child mortality. Many low- and middle-income countries (LMICs) are striving to achieve RMNCH-related Sustainable Development Goals (SDGs). We monitored progress, made projections, and calculated the average annual rate of change needed to achieve universal (100%) access of RMNCH service indicators by 2030.

**Methods:**

We extracted Demographic and Health Survey (DHS) data of 75 LMICs to estimate the coverage of RMNCH indicators and composite coverage index (CCI) to measure health system strengths. Bayesian linear regression models were fitted to predict the coverage of indicators and the probability of achieving targets.

**Results:**

The projection analysis included 64 countries with available information for at least 2 DHS rounds. No countries are projected to reach universal CCI by 2030; only Brazil, Cambodia, Colombia, Honduras, Morocco, and Sierra Leone will have more than 90% CCI. None of the LMICs will achieve universal coverage of all RMNCH indicators by 2030, although some may achieve universal coverage for specific services. To meet targets for universal service access by 2030, most LMICs must attain a 2-fold increase in the coverage of indicators from 2019 to 2030. Coverage of RMNCH indicators, the probability of target attainments, and the required rate of increase vary significantly across the spectrum of sociodemographic disadvantages. Most countries with poor historical and current trends for RMNCH coverage are likely to experience a similar scenario in 2030. Countries with lower coverage had higher disparities across the subgroups of wealth, place of residence, and women’s/mother’s education and age; these disparities are projected to persist in 2030.

**Conclusion:**

None of the LMICs will meet the SDG RMNCH 2030 targets without scaling up essential RMNCH interventions, reducing gaps in coverage, and reaching marginalized and disadvantaged populations.

## Abstract (precis)

What progress has been achieved toward reproductive, maternal, newborn, and child health service related Sustainable Development Goals? Analyzing data to estimate coverage of these indicators, we observed that acceleration is needed in coordinated global efforts and government policies to ensure universal access to RMNCH care services by 2030.

## Teaser

**Key Findings:**

- Progress in reproductive, maternal, newborn,and child health care service coverage is increasing but is uneven between countries and across subgroups (in terms of wealth, place of residence, education, age, and sex) within countries. These coverage gaps are projected to continue.
- By 2030, none of the low- and middle-income countries would be able to achieve the target of universal coverage for oral rehydration therapy for diarrhea treatment or to seek care for acute respiratory infections. Only a few countries are likely to achieve universal coverage for demand for family planning satisfied with modern contraceptive methods, recommended visits for antenatal care, and skilled birth attendant for assistance during birth.

**Key Implications:**

- When designing appropriate interventions for increasing the coverage of reproductive, maternal, newborn, and child health care services, program managers should consider disadvantaged and marginalized populations.
- Acceleration is needed in coordinated global efforts and government policies focusing on marginalized groups, administering cost-effective interventions, and implementing proactive follow-up for routinely scheduled health care services.

## INTRODUCTION

Reducing maternal and child morbidity and mortality and improving reproductive, maternal, newborn, and child health (RMNCH) are top priorities of the global health agenda, particularly for low- and middle-income countries (LMICs).^1^ During the era of the Millennium Development Goals (MDGs) between 1990 and 2015, coverage of effective RMNCH interventions to reduce maternal and child morbidity and mortality was scaled up in LMICs.^2^ This global initiative showed rapid progress in increasing the coverage of RMNCH care services such as accelerated coverage of demand for family planning satisfied with modern contraceptive methods (mDFPS), presence of a skilled birth attendant (SBA), and radically increased coverage of child vaccinations, while other services had modest progress and a few were far behind in meeting the global targets.^3^ Despite significant improvements in health MDGs globally, the population-level inequality between the poorest and richest households and between urban and rural areas did not change in many LMICs.^1^ Most importantly, individual-level disparities in terms of gender, age, education, and geographical location suggested further review of global agendas for designing and implementing RMNCH interventions was needed.^1^

In 2015, the United Nations General Assembly summit global developmental agenda shifted from MDGs to Sustainable Development Goals (SDGs).^4^ The top priority of SDG target 3.8 is to achieve universal health coverage (UHC), which means that^5^:

*all individuals and communities receive the health services they need without suffering financial hardship.*

Forty years after the adoption of the historic Declaration of Alma-Ata, the World Health Organization (WHO) in partnership with the United Nations Children’s Fund (UNICEF) and the Ministry of Health of Kazakhstan hosted the Global Conference on Primary Health Care in October 2018 to recommit to primary health care as the cornerstone of UHC in the new Declaration of Astana.^5^ The aims of the declaration are to renew political commitment to primary health care from governments, nongovernmental organizations, professional organizations, academia, and global health and development organizations. RMNCH care services constitute a significant portion of UHC, and reaching and maintaining high rates of coverage of priority interventions indicate the strength of health systems of a country.^6^ The results of the Countdown Network suggest that in many LMICs with the highest burden of maternal and child mortality, coverage of some RMNCH care services remains poor, including mDFPS, oral rehydration therapy (ORT), and care seeking for acute respiratory infections (ARI care).^7^ However, no projections were made to identify which countries are unlikely to achieve global RMNCH targets. To bridge this evidence gap, the Global Burden of Diseases (GBD) collaborators recently examined trends and projected target attainments of 41 health-related SDG indicators in many countries and territories.^8^ Again, projections of these indicators across socioeconomically disadvantaged subgroups are still missing in the existing literature.

Trend analysis helps policy makers and program managers assess current progress, reformulate policies, and design necessary interventions. Projections for RMNCH care services across different sociodemographic dimensions are central to identifying the key priority areas or groups (i.e., identifying the most disadvantaged groups to be covered under interventions) to reinforce or reformulate current policies for achieving country goals. A number of studies, including those conducted by the Countdown Network and GBD, have evaluated the current status, examined trends, and made projections of RMNCH care services and some composite indices at the global, regional, or country level.^8^ ^–^ ^14^ However, none of these studies captured key interventions for RMNCH separately to make projections across subgroups by sociodemographic stratifications.

Projections for RMNCH care services across different socio-demographic dimensions are central to identifying the key priority areas or groups.

In this study, we used the most recent data to assess progress, make projections, and calculate the probability of target attainment and the required average annual rate of change (AARC) for achieving targets of RMNCH care services across various population subgroups within LMICs. We also calculated gaps in coverage of services across a set of sociodemographic dimensions. We did our analyses within and between countries to identify the most disadvantaged countries and groups within countries with inadequate access to RMNCH care services.

## METHODS

### Data Sources

To calculate the coverage of RMNCH care services, we used macro-level (aggregated) data from large-scale, population-based, nationally representative cross-sectional surveys conducted repeatedly between 1990 and 2018 under the Demographic and Health Surveys (DHS) program^15^ in LMICs. Established in 1984 by the United States Agency for International Development, the DHS program aims to provide decision makers in participating countries with improved information and analyses useful for informed policy choices, improve coordination and partnerships in data collection at the international and country levels, develop the skills and resources necessary to conduct high-quality demographic and health surveys, improve data collection and analysis tools and methodology, and improve the dissemination and utilization of data.^15^ The DHS program provides population-based, repeated cross-sectional data that capture a wide range of monitoring and impact evaluation indicators in the areas of population, health, and nutrition. Since the program began, more than 300 nationally representative household-based surveys have been completed under the DHS project in more than 90 countries. Many of the countries have conducted multiple DHS surveys to establish trend data that enable them to gauge progress in their programs. The samples of DHS surveys are generally representative at the national, residence (urban to rural), and regional level (departments, states, or divisions). The collection of the DHS sample is usually based on a stratified multistage cluster design. The data are made available by MEASURE DHS.

DHS obtained data through standardized interviews of women of reproductive age (15–49 years) from the countries under their program, which included a list of prioritized countries for the Countdown cycle.^7^ ^,^ ^16^ We downloaded, managed, and combined the data from the website to track the progress and make projections about coverage of RMNCH care services at national and subpopulation levels.

### RMNCH Care Service Indicators

We selected 8 indicators related to RMNCH care services from a range of intervention areas to assess health care systems or delivery for mothers and their children throughout their life stages, across the continuum of care and aligning with global targets.^7^ These indicators included mDFPS; antenatal care visits (ANC); presence of an SBA; child immunizations for measles, BCG, and 3 doses of diphtheria-pertussis-tetanus (DPT); ORT for diarrhea treatment; and ARI care. Global standard definitions were used in defining RMNCH care service indicators (Table). Notably, we considered ANC as receiving service at least 4 times from any provider or at least once from a medically trained provider to ensure that the estimates of ANC can be captured from the maximum number of study countries. In addition, we constructed a composite coverage index (CCI) by using the 8 RMNCH care service indicators according to the formula proposed by Boerma et al. The CCI is a weighted mean of the 8 RMNCH care service indicators (Supplement 1 includes more details).^17^ To construct the index, we considered all DHS surveys that contained information on all RMNCH care services. However, we performed trend analysis only for the countries with data available for at least 2 DHS rounds to ascertain the trends. The estimates of CCI were not computed for DHS surveys with missing information on any of the RMNCH care services.

We selected 8 indicators related to RMNCH care services to assess health care systems or delivery for mothers and their children.

**TABLE.** Reproductive, Maternal, Newborn, and Child Health Care Services Indicators for the Composite Coverage Index

**Indicators**	**Definitions**	**SDG Target**	**Target Used in This Study for Calculating Probability**
Prepregnancy	** **		
Demand for family planning satisfied with a modern method among married women	The proportion of married women aged 15–49 years who do not want any more children or want to wait 2 or more years before having another child and are using modern contraception	Universal access^a^	≥99%
Pregnancy			
Antenatal care visits	The proportion of women aged 15–49 years in the 3 years preceding the survey who received at least 4 visits from any provider or at least 1 visit from a medically trained provider (i.e., a doctor, nurse, or midwife) during their last pregnancy	Universal access	≥99%
Birth			
Skilled attendance at birth	The proportion of livebirths assisted by a skilled health provider (i.e., a doctor, nurse, or midwife) in the 3 years preceding the survey	Universal access	≥99%
Infancy and early childhood			
BCG immunization	The proportion of children aged 12–23 months who received 1 dose of the BCG vaccine	Universal access	≥99%
DPT immunization	The proportion of children aged 12–23 months who received 3 doses of the DPT vaccine	Universal access	≥99%
Measles immunization	The proportion of children aged 12–23 months vaccinated against measles	Universal access	≥99%
Childhood			
Oral rehydration therapy	The proportion of children aged 5 years or younger with diarrhea who received oral rehydration therapy (i.e., oral rehydration salts, recommended home solution, or increased fluids) in the previous 2 weeks	Universal access	≥99%
Care seeking for symptoms of acute respiratory infections	The proportion of children aged 5 years or younger with symptoms of acute respiratory infections for whom medical treatment was sought from an appropriate health provider in the previous 2 weeks	Universal access	≥99%

Abbreviations: BCG, bacille Calmette-Guérin; DPT, diphtheria, pertussis, and tetanus; SDG, Sustainable Development Goal.

a Universal access is 100%.

### Statistical Analyses

We estimated the weighted coverage of RMNCH care services as proportions along with 95% confidence intervals from the original survey data. We calculated the coverage of RMNCH care services across subgroups in terms of wealth quintiles, place of residence, education of women/mother, age of women/mother, and sex of child (for child health care services). We used the variables that DHS constructed to present the estimates in the reports. The socioeconomic status of households was determined according to the asset-based wealth index as a proxy measure of household socioeconomic status.^18^ The DHS constructed the household wealth index based on household characteristics and ownership of assets by principal component analysis.^19^ The households were ranked based on wealth scores and divided into quintiles, from the poorest quintile (lowest 20% of the index) to the richest quintile (highest 20% of the index). The DHS generated variables on place of residence (rural and urban) based on geographical and administrative locations and education (no education, primary, secondary and higher) based on year of schooling. For this study, we categorized the education variable and classified as less than secondary-level education (no education and primary level) and secondary-level or higher education to stratify the study population. See DHS reports for more details.^10^ Notably, we restricted our analysis at the country level but not at the regional level for 2 reasons. First, some regions had few numbers of countries and had heterogeneity between survey years, and second, we were interested in assessing progress across individual countries so that country-level programs and policies could be implemented.

To examine trends, Bayesian linear regression models that used a Markov Chain Monte Carlo algorithm of multiple imputations for missing data were applied to estimate the coverage of RMNCH care services and trends from 1990 to 2018 (Supplement 2). We extended this trend analysis to project the coverage of RMNCH care services up to 2030 as set for achieving the SGD target. We reported credible intervals drawn from Bayesian regression analysis along with the estimates. We calculated the probability of achieving the coverage of RMNCH care services as 99% or more by 2030 to understand which countries and populations within each country are on track to achieve universal coverage of these services. We also validated our estimates drawn from regression models with those drawn from the original micro-data (Supplement 2 and Supplement 3 Table S12).

We used Stata (version 15.1) and R (version 3.5) statistical software to analyze our data.

## RESULTS

### Sample Characteristics

We extracted data from a total of 283 surveys from 75 LMICs, of which 64 countries (272 surveys) were surveyed at least twice and included in the trend analysis. Projections of CCI were made for 59 countries that had information for all 8 RMNCH care services for at least 2 DHS rounds. More than 4.2 million women 15–49 years of age were included for reproductive and maternal health care services, and more than 2.5 million children under 5 years of age were included for newborn and child health care services. A detailed description of the survey year and number of participants are presented in Supplement 3 (Table S1). All the fitted models for projection analysis achieved convergence. The potential scale reduction factor values are summarized in the Supplement 3 (Table S2 to Table S11).

We extracted data from 283 surveys from 75 LMICs, of which 64 countries were surveyed at least twice and included in the trend analysis.

### Trends and Projections

**FIGURE 1.**
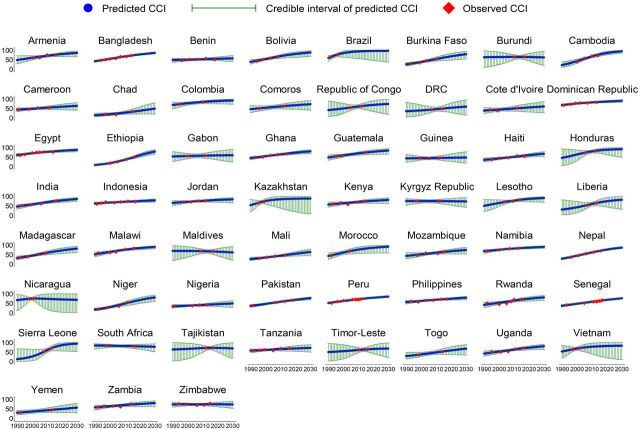
Progress and Projections of Composite Coverage Index in Low- and Middle-Income Countries Abbreviation: CCI, composite coverage index.

**FIGURE 2.**
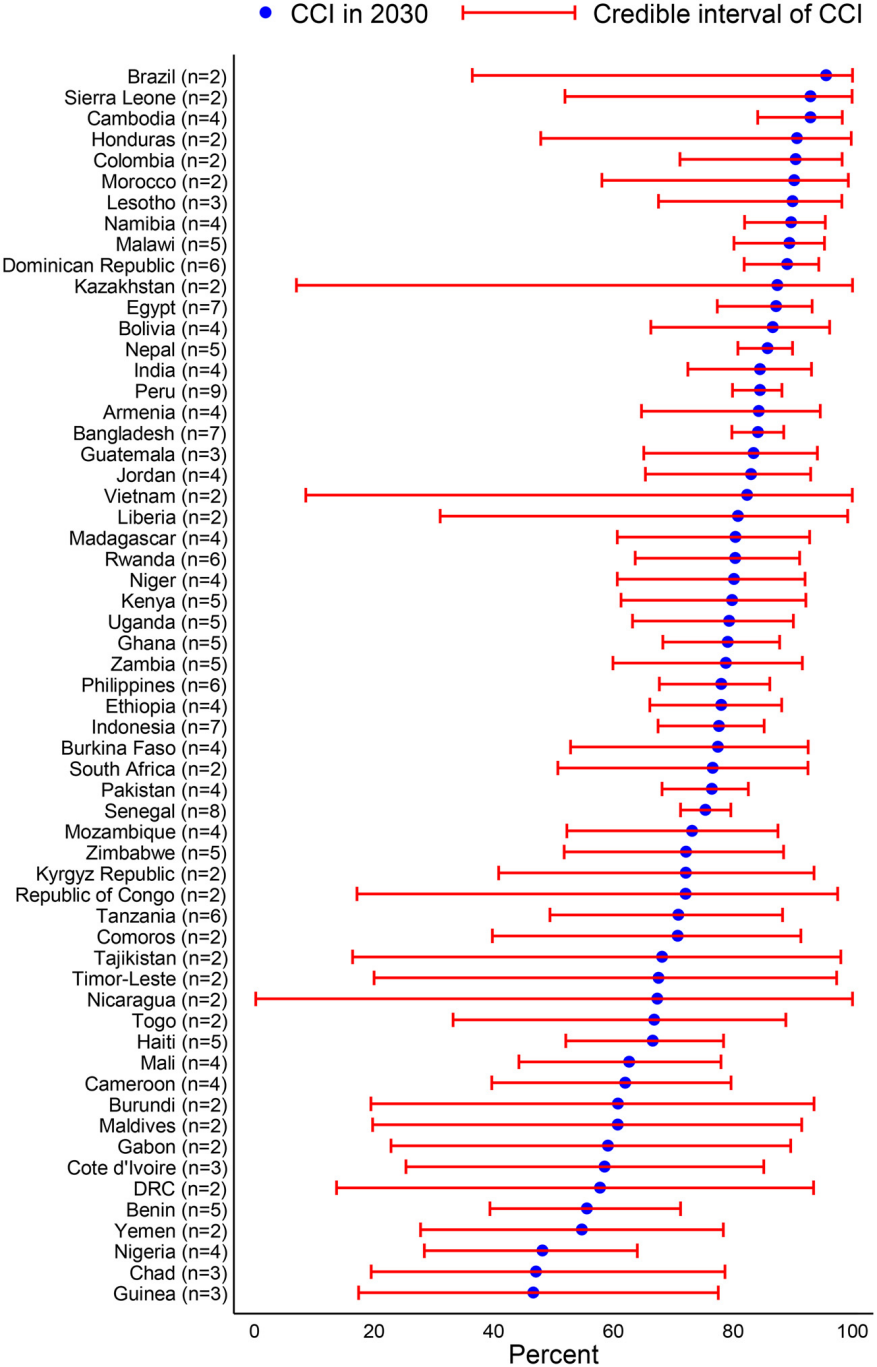
Projected Coverage in Percentages of Composite Coverage Index in 2030 Across Countries^a^ Abbreviation: CCI, composite coverage index.^a^ Values in parentheses represent the number of surveys of the respective country that were used in the analysis.

From 1990 to 2018, the CCI increased in all LMICs and is projected to continue increasing (Figure 1). However, the progressions varied between countries. Based on the current trend, 34 of 59 countries (56.7%) are projected to have less than 80% CCI by 2030. The country-specific projections showed that only Brazil (95.6%), Sierra Leone (93.0%), Cambodia (93.0%), Honduras (90.7%), Colombia (90.5%), and Morocco (90.3%) are likely to have more than 90% CCI. A number of countries (17 of 59 countries) are projected to have poor CCI (less than 70%) in 2030, with the lowest CCI in Guinea (46.7%), Chad (47.1%), Nigeria (48.2%), Yemen (54.6%), and Benin (55.6%) (Figure 2).

Among countries included in the trend analysis, more than 90% coverage is projected to be achieved by 14 of 62 countries for mDFPS, 41 of 64 countries for ANC, 29 of 63 countries for presence of an SBA, 22 of 61 countries for measles immunization, 28 of 60 countries for 3 doses of DPT vaccine, 42 of 61 countries for BCG, 3 of 61 countries for ORT, and 3 of 62 countries for ARI care by 2030. In 2030, the lowest levels of coverage are projected to be in Albania (1.5%) for mDFPS, in Burundi (0.1%) for ANC, in Angola (8.7%) for presence of an SBA, in Kazakhstan (2.4%) for BCG immunization, in Gabon (11.2%) for 3 doses of DPT vaccine, in Nicaragua (7.0%) for measles immunization, in Cameroon (15.3%) for ORT, and in Guinea (14.6%) for ARI care (Supplement 3 Figure S9 to Figure S16).

**FIGURE 3.**
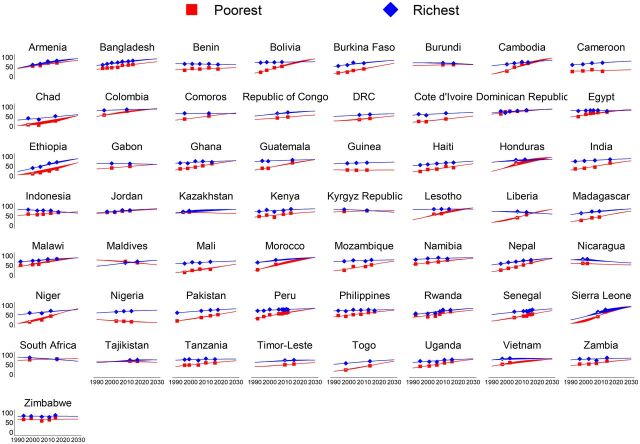
Trends in Predicted Composite Coverage Index Across Countries by Wealth Quintiles

### Inequalities

**FIGURE 4.**
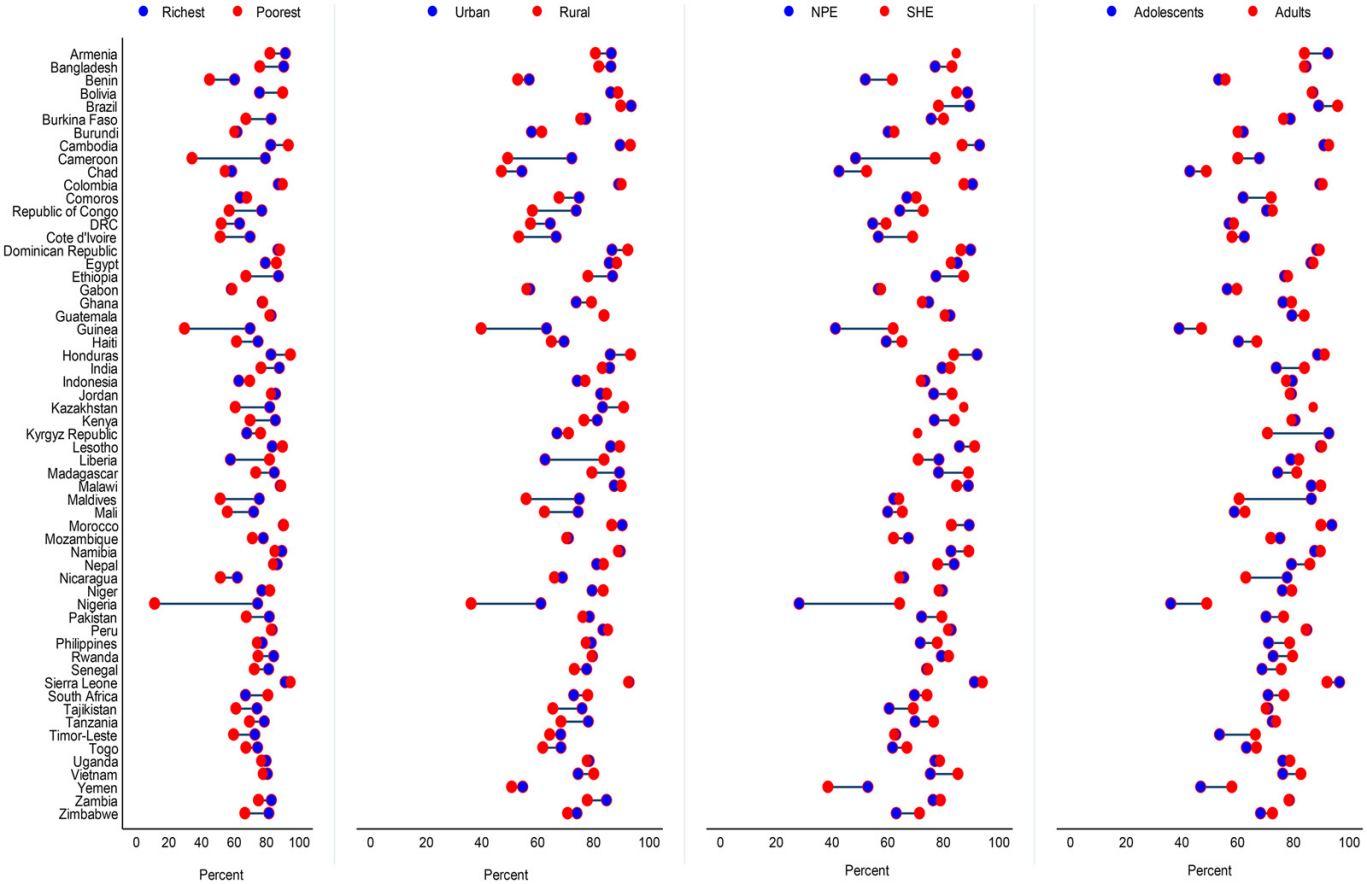
Projected Gaps in Composite Coverage Index Across Countries by Wealth Quintiles, Place of Residence, and Women’s/Mother’s Education and Age in 2030 Abbreviations: NPE, no education and primary-level education; SHE, secondary or higher-level education.

The intracountry inequalities show that significant gaps exist in the coverage of RMNCH care services across population subgroups, and these gaps are projected to continue into the future (Figure 3 and Supplement 3 Figure S17 to Figure S56). The gaps for CCI between the richest and poorest households are projected to be larger, yielding greater CCI among the richest compared to the poorest, with the largest gap in Nigeria by 63.4 percentage points and the smallest gap in Peru by 0.5 percentage point (Figure 4). In contrast, the CCI is projected to be greater among the poorest compared to the richest by 23.9 percentage points in Liberia. Most of the countries with the largest richest-poorest gaps are likely to experience larger urban-rural gaps as well in the CCI, with the greatest CCI gap in the urban population by 25.1 percentage points in Nigeria and the smallest gap in Guatemala by almost nil (Figure 4). In line with richest-poorest and urban-rural gaps, the coverage gaps between women with less than secondary-level education and women with secondary-level or higher education are also expected to remain larger in 2030, with the largest CCI gap among the women with secondary-level education or higher compared with women with less than secondary-level education in Nigeria by 36.1 percentage points and smallest gap in Indonesia by 0.1 percentage points. The CCI gaps between adolescent and adult women are also apparent, but these gaps are considerably narrower than gaps observed across wealth, residence, and education (Figure 4). Indicator-specific projections highlight that the gaps in the coverage of all 8 RMNCH care services are expected to be largely apparent in 2030, predominantly between the richest and poorest at the national level and across urban-rural residence (Supplement 3 Figure S57 to Figure S88).

Significant gaps exist in the coverage of RMNCH care services across population subgroups, and they are projected to continue into the future.

We also tracked progress in newborn and child health care services based on sex of the child. By 2030, the projected coverage of ORT will be less than 80% in most of the LMICs for both boys and girls (Supplement 3 Figure S92). Similarly, the coverage of ARI care for both boys and girls is projected to be less than 80% by 2030 in most of the countries (Supplement 3 Figure S93). The current sex-based gaps in child immunization rates are also likely to persist in some countries in 2030 (Supplement 3 Figure S89 to Figure S91).

### Probability of Target Attainment

According to the posterior probability, Brazil (72%) has the highest probability of achieving universal CCI, followed by Kazakhstan (40%) and Sierra Leone (20%) (Supplement 3 Table S13). Our results indicate that it is unlikely that any of the LMICs will achieve universal CCI by 2030. Some countries are likely to achieve universal coverage for some RMNCH care services, particularly ANC visits, presence of an SBA, and BCG immunization in Armenia, Brazil, Cambodia, and Jordan. But the probability of achieving universal coverage for other services is close to zero for the majority of the countries. The posterior probability of achieving universal coverage of RMNCH care services across subgroups is also zero for most of the countries (Supplement 3 Table S15 to Table S22). Additi-onally, we calculated the posterior probability of countries achieving at least 75% coverage for mDFPS. The results showed that nearly one-third (19 of 62 countries) of the countries are on track to achieve the target of at least 75% mDFPS coverage with at least 90% probability of attaining the goal (Supplement 3 Table S23).

None of the LMICs are likely to achieve universal CCI by 2030, although some may achieve universal coverage for certain RMNCH care services.

### Change Rates

**FIGURE 5.**
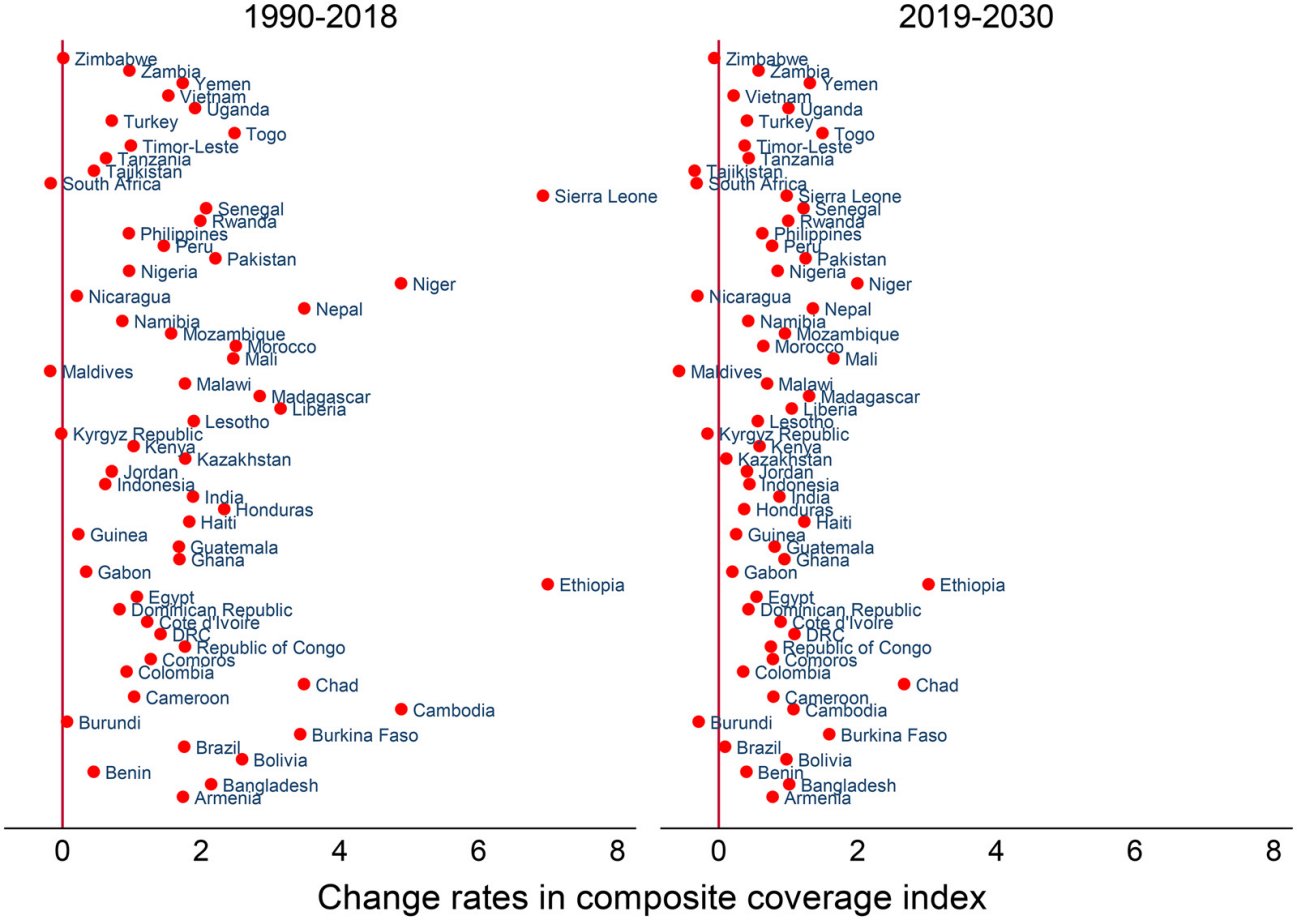
Average Annual Rate of Change^a^ in Composite Coverage Index in Low- and Middle-Income Countries ^a^ Annual rate of change is calculated as: ln[(rate in latest year/rate in earliest year)]/(latest year − earliest year), with positive values located on the right side of the diagonal line at 0 (in X-axis) denoting an increasing rate, while negative values located on the left side of the diagonal line at 0 (in X-axis) denoting a decreasing rate.

The progression rates in CCI varied over time; slower rates of progression in CCI are projected in most of the countries during 2019–2030 compared with the progression rate during 1990–2018 (Figure 5). Some countries (e.g., Maldives, −0.2%) had retrogression in CCI during 1990–2018 that will continue during 2019–2030. The calculated AARC shows that achieving the target will require ramping up the rate at which CCI increases annually between 2019 and 2030, particularly by 9.5% in Chad, 7.5% in Nigeria, 7.2% in Guinea, and 6.8% in Yemen (Figure 5). The largest improvements are required for mDFPS for most of the countries, urgently in Albania by 28.2%, Maldives by 15.0%, Democratic Republic of the Congo by 13.3%, Chad by 13.2%, and Yemen by 11.1% (Supplement 3 Table S28). Acceleration in improving the coverage of both ORT and ARI care needs to be at an annual rate of 3%–10% for almost all the countries to achieve the targets (Supplement 3 Table S61 and Table S67). However, the AARC varied across different sociodemographic dimensions within countries (Supplement 3 Table S24 to Table S72 includes details for all RMNCH care services).

## DISCUSSION

This study provides the most up-to-date estimates on the progress of LMICs toward the key RMNCH care services, and it predicts coverage of these services by 2030 to detect whether RMNCH targets can be achieved. Based on current trends, we demonstrated that none of the LMICs would be able to meet the target coverage for either ORT for diarrhea treatment or ARI care. Although the coverage of RMNCH care services is increasing, the coverage gaps across sociodemographic dimensions remain and are projected to persist. Substantial variations exist in the coverage of RMNCH care services between countries and between subgroup levels within countries. These results emphasize the need for effective policies focusing on marginalized groups, administering cost-effective interventions, and implementing proactive follow-up for routinely scheduled health care visits to ensure universal access to RMNCH care services. The results of this study provide evidence to inform global and country leaders and policy makers about the country-specific situations at national and subgroup levels and highlights key areas of interventions (such as improving ORT and ARI care services) that need urgent attention for increasing the coverage of these services through allocating national funding and resources toward achieving the 2030 target for RMNCH care services.

Although the coverage of RMNCH care services is increasing, coverage gaps across socio-demographic dimensions remain and are projected to persist.

### Specific Services

Our results indicate that all countries are unlikely to achieve universal CCI. Some countries are on track to achieve universal coverage for childhood immunization for BCG, DPT, and measles vaccines. Concurrently, some countries such as Maldives, Nigeria, Tajikistan, Yemen, Chad, and Zimbabwe are projected to have less than 80% childhood immunization coverage in 2030. The results of our study demonstrate that coverage of 2 care-seeking services for child morbidity, ORT, and ARI care will be remarkably lower (less than 50% in 25 countries of 61 for ORT and 18 countries of 62 for ARI care) than the target coverage in LMICs. The probability of achieving universal coverage for these 2 services by 2030 is roughly zero for all countries, except Sierra Leone (57% probability) for ORT and Brazil (39% probability) for ARI care.

By 2030, universal coverage is expected to be achieved by Liberia for mDFPS; Maldives, Armenia, and Cambodia for ANC; and Armenia, Honduras, and Jordan for presence of an SBA. However, our results demonstrate that most of the countries are struggling to achieve universal coverage of mDFPS, ANC, and presence of an SBA. In addition, the target coverage of these 3 services will not be achieved by most of the subgroups within each of the LMICs. The lower coverage of mDFPS, ANC, and presence of an SBA among the poorest populations, those living in rural areas, and women with less education will impede LMICs, particularly countries in South and Southeast Asia and sub-Saharan Africa, in achieving the target coverage for these 3 services. Although the overall CCI increased, we project that LMICs and all subgroups within LMICs will not be able to reach universal CCI by 2030, especially due to the lower CCI led by mDFPS, ORT, and ARI care among adolescent girls and mothers and among women and mothers who are poor, have less education, and live in rural areas. Our findings correspond with those from previous studies, with negligible variations,^8^ which were mainly driven by the number of time points with available data analyzed.

### Equity

Based on our results, large coverage gaps exist in childhood immunization coverage between poor and rich households, rural and urban populations, mothers with low and high education levels, and adolescent and adult mothers. To achieve universal immunization coverage by 2030, most countries need to further ramp up of coverage, particularly for the poorest and rural populations and less educated and adolescent mothers in LMICs with low coverage of RMNCH care services. To increase the coverage of RMNCH care services, equitable, appropriate, and focused programs need to be implemented, and resources need to be allocated to increase availability, accessibility, and use of services, particularly for those groups shown to be the furthest behind in the current study (such as poorest, rural, and less educated populations). These programs may help countries to reduce coverage gaps within countries toward achieving the global target of UHC.

Our analysis found considerable disparities in the coverage in ORT and ARI care in terms of wealth, place of residence, education and age of mother, and sex of the child. These gaps may persist until 2030 in some LMICs, predominantly in countries in sub-Saharan Africa. In most LMICs, the coverage for ORT and ARI care will be less than 80% across most subgroups. This projection may partly be explained by broader baseline gaps in ORT and ARI care among the subgroups in LMICs. In general, people who were poorest, resided in rural areas, or were adolescent and less educated mothers will remain vulnerable for achieving the target coverage by 2030. This finding suggests that children belonging to either of these vulnerable groups should be given special consideration in the design of interventions to scale up RMNCH care services.

The gap in CCI must be considered before planning for actions to improve the strengths of health systems. As the projected estimates reveal that none of the LMICs will be able to achieve the CCI target by 2030, we postulate that the lower CCI among the poorest, rural, women/mother with less than secondary-level education, and adolescent women/mother groups has a substantial contribution to the lower CCI. To achieve universal coverage, accelerations on improvements are essential in LMICs with nearly 4% improvements in annual national coverage and 2%–5% improvements in annual coverage at subgroup levels in LMICs. All countries are projected to fail to achieve the CCI target coverage by 2030 at national and subgroup levels, and only some Latin American and Caribbean countries will have more than 80% CCI and are on track for achieving the target if effective RMNCH strategies can be implemented. However, most sub-Saharan African countries will be far behind in reaching the CCI target. Similar to LMICs, the subgroup coverage gaps in RMNCH care services will constitute the key driver behind this target failure. To accomplish the goals of achieving universal access to RMNCH care services, sub-Saharan African countries need to increase the coverage of RMNCH care services by more than 3 times during 2019–2030 than what was calculated during 1990–2018, giving particular attention to the poorest, rural, and less educated and adolescent women/mothers.

For the future progress of RMNCH care services, it is imperative to understand the reasons for lower coverage or gaps in coverage and the associated factors for high or low coverage across different geographical settings. It is well known that between- and within-country inequalities and the lack of financial resources are major constraints for improving RMNCH.^17^ ^,^ ^20^ In line with previous evidence,^16^ our study also demonstrates that coverage of health care services that can be scheduled in advance, such as immunization coverage, were higher and are likely to be achieved by 2030, while those that require emergency on-demand availability of workforce and specialized equipment (e.g., presence of an SBA) and acute care for childhood illness (e.g., ARI care) had lower coverage and are highly unlikely to reach the target by most of the countries. To improve emergency on-demand care, acceleration of relevant actions and increase of investments are crucial for adequate access, human resources, and demand-based supplies for the population.

### Strengths and Limitations

In this study, we used globally recognized nationally representative data to calculate the coverage of RMNCH care services that provided reliable estimates of trends along with the AARC during different periods. We used a set of globally accepted standard outcome interventions that cover life stages of women during prepregnancy to childhood of their offspring at the population level and across the continuum of care. The use of large samples from population-based household surveys enabled us to estimate national and subgroup-level trends across countries as well as across subgroups within countries. The unique survey methodology and measurement of the DHS allowed this study to make cross-country comparison of estimates as well. However, the findings of our study need to be interpreted in light of some limitations.

For cross-country comparison, we considered a doctor, nurse, or midwife as skilled personnel for assisting birth as recommended.^21^ This underestimates the coverage estimates of skilled birth attendance for some countries that may have other skilled service providers, such as paramedics, family welfare visitors, and community skilled birth attendants. Because some countries had too few surveys with available information, we could not make projections of RMNCH care services for those individual countries. Interventions to improve RMNCH care services come in phases and may reach some subpopulations before others. However, we were unable to examine whether the past changes would proceed uniformly in the future within and across countries due to the heterogeneity in survey years within and across countries. Fewer data points for some RMNCH care services for some countries may have created wider credible intervals for the projected estimates of the service coverage (e.g., for CCI in Nicaragua). Credible intervals with a wide range are normal for projection analysis, but they could be narrowed by having multiple time points available (e.g., for CCI in Bangladesh). Calculating more realistic probability estimates is also possible with wider credible intervals. Estimates drawn from representative data collected from multiple sources may better project the future directions of the RMNCH care services with lower uncertainty. Moreover, all the estimates drawn from DHS data were mostly based on self-reports of respondents and hence may have recall bias in reporting. However, DHS followed standard methodology and questionnaires for more than 3 decades to provide population-based data that are representative at not only national but also subnational levels.

## CONCLUSIONS

Although the coverage of RMNCH care services is improving in LMICs, the progress is uneven within and between countries and insufficient to meet the health SDGs. Most sub-Saharan African and South and Southeast Asian countries are very unlikely to achieve target coverages by 2030 due to low coverage overall and high coverage gaps in RMNCH services between the richest and poorest, urban and rural, and high and low education subgroups. These results reflect the urgent need for health interventions targeting disadvantaged countries and their subgroups to achieve universal access to health services and to reduce health inequalities during the SDG era. Increasing funding for RMNCH care through cost-effective interventions may strengthen health care services and can help interventions reach marginalized and disadvantaged people. Country leaders, stakeholders, and agencies need to undertake multidisciplinary collaborative actions by going beyond their commitment in allocating resources, implementing programs, and monitoring the progress and gaps in RMNCH care services toward achieving SDG target 3.8 by 2030.

## Supplementary Material

20-00097-Hasan-Supplement2.docx

20-00097-Hasan-Supplement1.docx

20-00097-Hasan-Supplement3.docx

## Notes

**Peer Reviewed**

**First published online:** October 8, 2020

**Funding:** MMH acknowledges the financial assistance from the University of Queensland and the Commonwealth Government of Australia to undertake his PhD.
