# Supplementary material for: Meeting the Global Target in Reproductive, Maternal, Newborn, and Child Health Care Services in Low- and Middle-Income Countries
Source: Glob Health Sci Pract. 2020 Dec 23;8(4):654–65. doi: 10.9745/GHSP-D-20-00097 (PMC7784071; doi:10.9745/GHSP-D-20-00097)
Supplement: 20-00097-Hasan-Supplement2.docx [file 20-00097-Hasan-Supplement2.docx]

# Supplement 2

## Modeling

We applied Bayesian linear regression models using normally distributed non-informative priors to calculate the estimates of intervention coverage during 1990-2018. This technique was previously used to examine trends for health indicators^3^. For analysis, we first draw the prevalence estimates in proportions for all intervention coverages for all survey years of each study countries. We performed a logit transformation of all proportions. All the calculations were done after this transformation and then transformed back to probabilities to ensure that the predicted probabilities lie between 0 and 1. We considered “survey year” as time as a covariate in each model.

We obtained 30000 samples from the posterior distribution by applying a Markov Chain Monte Carlo (MCMC) algorithm with two chains. MCMC is a general sampling method to draw values/samples from parameter distributions which are then used to estimate the posterior distribution of parameters given the data. We used both trace plots and Gelman-Rubin diagnostic statistics to check parameter convergence by using the value of potential scale reduction factor (PSRF)^4^. We consider that the convergence was achieved if the value of PSRF was close to 1, and a value greater than 1.10 was considered as convergence failure. We used rjags package^5^ in the R programming language with JAGS, a BUGS language, MCMC library for generating the estimates. For each model, the first 5000 iterations were discarded as burn-in and the number of iterations were increased until the output was diagnosed as convergent. During checking of the convergence, trace plots were generated each time to verify the mixing of iterations, and the iteration chains were always past in burn-in phase. These posterior predictive distributions were used to obtain estimates along with 95% credible intervals (CrI). We conducted subgroup analysis to examine the projections across subgroups such as socioeconomic status, place of residence, education, and age of women to understand the sociodemographic groups who are lagging.

## Model equation

We defined our indicators y_i_ as the logit transformed value of the proportion of the indicator and assumed the distribution as follows:

y_i ~_ N(µ_i_, σ_i_^2^) …….. (i)

Here, µ_i_ is the conditional mean of the indicator and has the following linear form:

µ_i_ = β_0_ + β_1_ x_i_ ……… (ii)

where, x_i_ is the time, β_0_ is the intercept and β_1_ is the regression coefficient. The prior distribution for β_0_ and β_1_ was assumed as follows:

β_0 ~_ Normal(0, 0.0001)

β_1 ~_ Normal(0, 0.0001)

and

σ^2^ _~_ Normal(0.0001, 100)

We fitted the model separately for each indicators of each countries at overall level. We repeat the procedure to make projections for each subgroups of each countries.

## Handling missing values

We omitted the countries for the indicators with missing values for trend analysis. We used information for women’s indicator whose age 15-49 years and child indicators whose age 0-59 months. To reduce missing values during logit transformation, we considered the proportion value as 0.9999 for 1. For example, the logit transformed value of 1 is undefined for BCG immunization in Albania in 2018.

## Cross-validation of estimates

We compared the estimates drawn from the Bayesian linear regression model with those drawn from original micro-data. We calculated the absolute differences in the estimates drawn from regression models and original data for all the indicators for several countries for the most recent time points. We then checked the differences between them to validate the results. This procedure depicts that the difference between original and estimated coverage of interventions were very little, mostly differed by 0.5 to 1.0 percentage points for all RMNCH services in most of the countries. See **Table S12** for details.
