# Supplementary material for: Meeting the Global Target in Reproductive, Maternal, Newborn, and Child Health Care Services in Low- and Middle-Income Countries
Source: Glob Health Sci Pract. 2020 Dec 23;8(4):654–65. doi: 10.9745/GHSP-D-20-00097 (PMC7784071; doi:10.9745/GHSP-D-20-00097)
Supplement: 20-00097-Hasan-Supplement1.docx [file 20-00097-Hasan-Supplement1.docx]

# Supplement 1

## Construction of composite coverage index

Composite coverage index (CCI) was constructed by using a total of eight indicators under the four potential intervention areas: family planning, maternal and newborn care, immunization, and treatment for sick children. One to three indicators from each of the intervention areas were selected to construct the coverage index. These coverage indicators were consistent with those used in the 2008 Countdown. However, there are exceptions. BCG has been added under the immunization area and indicator on “Demand for family planning satisfied with modern contraceptive methods” was used for creation of coverage index. We used the following formula developed by Boerma and colleagues to construct the coverage index^1^:

$CCI=\frac{mDFPS +\left( ANC+SBA \right)*0.5+\left( BCG + 2*DPT+Measles \right)*0.25+\left( ORT+ARI \right)*0.5}{4}$

Where,

mDFPS= Demand for family planning satisfied with modern contraceptive methods

ANC= Antenatal care visits of at least 4 times

SBA= Skilled birth attendance

BCG= BCG immunization

DPT= Three doses of DPT immunization

Measles= Measles immunization

ORT= Oral rehydration therapy for diarrhoea treatment

ARI= Care seeking for symptoms of acute respiratory infections

The CCI is a useful tool for measuring the levels and trends of coverage of health services. Additional information on CCI can be found in www.countdown2030.org

The CCI is the weighted mean of the above indicators. We used Cronbach’s α to check the internal reliability among the indicators before constructing the indices. Though the theoretical value of α varies from 0 to 1, the value exceeding of 0.7 is considered as acceptable for the use of index construction^2^. In our study, the coefficient of the Cronbach’s Alpha (α) was 0.8203 in LMICs.
