# Supplementary material for: Meeting the Global Target in Reproductive, Maternal, Newborn, and Child Health Care Services in Low- and Middle-Income Countries
Source: Glob Health Sci Pract. 2020 Dec 23;8(4):654–65. doi: 10.9745/GHSP-D-20-00097 (PMC7784071; doi:10.9745/GHSP-D-20-00097)
Supplement: 20-00097-Hasan-Supplement3.docx [file 20-00097-Hasan-Supplement3.docx]

**List of Tables**

[Table S1: Countries with survey year and sample size for all indicators 9](#_Toc46135102)

[Table S2: Potential scale reduction factor (PSRF) to diagnose convergence after Markov Chain Monte Carlo simulation in Bayesian regression model for Composite Coverage Index (CCI) in low- and midle-income countries 14](#_Toc46135103)

[Table S3: Potential scale reduction factor (PSRF) to diagnose convergence after Markov Chain Monte Carlo simulation in Bayesian regression model for demand for family planning satisfied with modern contraceptive method (mDFPS) among women 15-49 years of age in low- and midle-income countries^1^ 16](#_Toc46135104)

[Table S4: Potential scale reduction factor (PSRF) to diagnose convergence after Markov Chain Monte Carlo simulation in Bayesian regression model for demand for family planning satisfied with modern contraceptive method (mDFPS) among women 15-49 years of age in low- and midle-income countries^1^ 18](#_Toc46135105)

[Table S5: Potential scale reduction factor (PSRF) to diagnose convergence after Markov Chain Monte Carlo simulation in Bayesian regression model for antenatal care visits among women 15-49 years of age in low- and midle-income countries 20](#_Toc46135106)

[Table S6: Potential scale reduction factor (PSRF) to diagnose convergence after Markov Chain Monte Carlo simulation in Bayesian regression model for skilled birth attendance among women 15-49 years of age in low- and midle-income countries 22](#_Toc46135107)

[Table S7: Potential scale reduction factor (PSRF) to diagnose convergence after Markov Chain Monte Carlo simulation in Bayesian regression model for BCG immunization among children 12-23 months of age in low- and midle-income countries 24](#_Toc46135108)

[Table S8: Potential scale reduction factor (PSRF) to diagnose convergence after Markov Chain Monte Carlo simulation in Bayesian regression model for three doses of DPT immunization among children 12-23 months of age in low- and midle-income countries 26](#_Toc46135109)

[Table S9: Potential scale reduction factor (PSRF) to diagnose convergence after Markov Chain Monte Carlo simulation in Bayesian regression model for Measles immunization among children 12-23 months of age in low- and midle-income countries 28](#_Toc46135110)

[Table S10: Potential scale reduction factor (PSRF) to diagnose convergence after Markov Chain Monte Carlo simulation in Bayesian regression model for oral rehydration therapy among children 0-59 months of age in low- and midle-income countries 30](#_Toc46135111)

[Table S11: Potential scale reduction factor (PSRF) to diagnose convergence after Markov Chain Monte Carlo simulation in Bayesian regression model for care seeking for acute respiratory infections among children 0-59 months of age in low- and midle-income countries 32](#_Toc46135112)

[Table S12: Cross-validation of estimates of the coverage of interventions drawn from original micro data and regression models for some countries during the most recent survey years in low- and middle-income countries 34](#_Toc46135113)

[Table S13: Predictive probability of achieving universal coverage of maternal, newborn and child healthcare services in low- and middle-income countries in 2030 160](#_Toc46135114)

[Table S14: Predictive probability of achieving universal coverage of CCI in low- and middle-income countries in 2030 by socio-demographic factors 162](#_Toc46135115)

[Table S15: Predictive probability of achieving universal coverage of demand for family planning satisfied with modern contraceptive method among women 15-49 years of age in low- and middle-income countries in 2030 by socio-demographic factors 163](#_Toc46135116)

[Table S16: Predictive probability of achieving universal coverage of antenatal care visit among women 15-49 years of age in low- and middle-income countries in 2030 by socio-demographic factors 164](#_Toc46135117)

[Table S17: Predictive probability of achieving universal coverage of skilled birth attendance among women 15-49 years of age in low- and middle-income countries in 2030 by socio-demographic factors 165](#_Toc46135118)

[Table S18: Predictive probability of achieving universal coverage of BCG immunization among children 12-23 months of age in low- and middle-income countries in 2030 by socio-demographic factors 166](#_Toc46135119)

[Table S19: Predictive probability of achieving universal coverage of three doses of DPT immunization among children 12-23 months of age in low- and middle-income countries in 2030 by socio-demographic factors 167](#_Toc46135120)

[Table S20: Predictive probability of achieving universal coverage of Measles immunization among children 12-23 months of age in low- and middle-income countries in 2030 by socio-demographic factors 168](#_Toc46135121)

[Table S21: Predictive probability of achieving universal coverage of oral rehydration therapy for diarrhoea treatment among children 0-59 months of age in low- and middle-income countries in 2030 by socio-demographic factors 169](#_Toc46135122)

[Table S22: Predictive probability of achieving universal coverage of care seeking for acute respiratory infections among children 0-59 months of age in low- and middle-income countries in 2030 by socio-demographic factors 170](#_Toc46135123)

[Table S23: Predictive probability of achieving at least 75% coverage of demand for family planning satisfied with modern contraceptive method among women 15-49 years of age in low- and middle-income countries in 2030 by socio-demographic factors 171](#_Toc46135124)

[Table S24: Predicted average annual rate of increase (AARC) of composite coverage index (CCI) in low- and middle-income countries by wealth quintiles 172](#_Toc46135125)

[Table S25: Predicted average annual rate of increase (AARC) of composite coverage index in low- and middle-income countries by place of residence 173](#_Toc46135126)

[Table S26: Predicted average annual rate of increase (AARC) of composite coverage index in low- and middle-income countries by education of women 174](#_Toc46135127)

[Table S27: Predicted average annual rate of increase (AARC) of composite coverage index in low- and middle-income countries by age of women 175](#_Toc46135128)

[Table S28: Predicted average annual rate of increase (AARC) of demand for family planning satisfied with modern contraceptive method among women 15-49 years of age in low- and middle-income countries 176](#_Toc46135129)

[Table S29: Predicted average annual rate of increase (AARC) of demand for family planning satisfied with modern contraceptive method among women 15-49 years of age in low- and middle-income countries by wealth quintiles 177](#_Toc46135130)

[Table S30: Predicted average annual rate of increase (AARC) of demand for family planning satisfied with modern contraceptive method among women 15-49 years of age in low- and middle-income countries by place of residence 178](#_Toc46135131)

[Table S31: Predicted average annual rate of increase (AARC) of demand for family planning satisfied with modern contraceptive method among women 15-49 years of age in low- and middle-income countries by education 179](#_Toc46135132)

[Table S32: Predicted average annual rate of increase (AARC) of demand for family planning satisfied with modern contraceptive method among women 15-49 years of age in low- and middle-income countries by age 180](#_Toc46135133)

[Table S33: Predicted average annual rate of increase (AARC) of antenatal care visit among women 15-49 years of age in low- and middle-income countries 181](#_Toc46135134)

[Table S34: Predicted average annual rate of increase (AARC) of antenatal care visit among women 15-49 years of age in low- and middle-income countries by wealth quintiles 182](#_Toc46135135)

[Table S35: Predicted average annual rate of increase (AARC) of antenatal care visit among women 15-49 years of age in low- and middle-income countries by place of residence 184](#_Toc46135136)

[Table S36: Predicted average annual rate of increase (AARC) of antenatal care visit among women 15-49 years of age in low- and middle-income countries by education 185](#_Toc46135137)

[Table S37: Predicted average annual rate of increase (AARC) of antenatal care visit among women 15-49 years of age in low- and middle-income countries by age 186](#_Toc46135138)

[Table S38: Predicted average annual rate of increase (AARC) of skilled birth attendance among women 15-49 years of age in low- and middle-income countries 187](#_Toc46135139)

[Table S39: Predicted average annual rate of increase (AARC) of skilled birth attendance among women 15-49 years of age in low- and middle-income countries by wealth quintiles 188](#_Toc46135140)

[Table S40: Predicted average annual rate of increase (AARC) of skilled birth attendance among women 15-49 years of age in low- and middle-income countries by place of residence 190](#_Toc46135141)

[Table S41: Predicted average annual rate of increase (AARC) of skilled birth attendance among women 15-49 years of age in low- and middle-income countries by education 191](#_Toc46135142)

[Table S42: Predicted average annual rate of increase (AARC) of skilled birth attendance among women 15-49 years of age in low- and middle-income countries by age 192](#_Toc46135143)

[Table S43: Predicted average annual rate of increase (AARC) of BCG immunization among children 12-23 months of age in low- and middle-income countries 193](#_Toc46135144)

[Table S44: Predicted average annual rate of increase (AARC) of BCG immunization among children 12-23 months of age in low- and middle-income countries by wealth quintiles 195](#_Toc46135145)

[Table S45: Predicted average annual rate of increase (AARC) of BCG immunization among children 12-23 months of age in low- and middle-income countries by place of residence 196](#_Toc46135146)

[Table S46: Predicted average annual rate of increase (AARC) of BCG immunization among children 12-23 months of age in low- and middle-income countries by education of mother 197](#_Toc46135147)

[Table S47: Predicted average annual rate of increase (AARC) of BCG immunization among children 12-23 months of age in low- and middle-income countries by age of mother 198](#_Toc46135148)

[Table S48: Predicted average annual rate of increase (AARC) of BCG immunization among children 12-23 months of age in low- and middle-income countries by child sex 199](#_Toc46135149)

[Table S49: Predicted average annual rate of increase (AARC) of three doses of DPT immunization among children 12-23 months of age in low- and middle-income countries 201](#_Toc46135150)

[Table S50: Predicted average annual rate of increase (AARC) of three doses of DPT immunization among children 12-23 months of age in low- and middle-income countries by wealth quintiles 202](#_Toc46135151)

[Table S51: Predicted average annual rate of increase (AARC) of three doses of DPT immunization among children 12-23 months of age in low- and middle-income countries by place of residence 203](#_Toc46135152)

[Table S52: Predicted average annual rate of increase (AARC) of three doses of DPT immunization among children 12-23 months of age in low- and middle-income countries by education of mother 205](#_Toc46135153)

[Table S53: Predicted average annual rate of increase (AARC) of three doses of DPT immunization among children 12-23 months of age in low- and middle-income countries by age of mother 206](#_Toc46135154)

[Table S54: Predicted average annual rate of increase (AARC) of three doses of DPT immunization among children 12-23 months of age in low- and middle-income countries by child sex 207](#_Toc46135155)

[Table S55: Predicted average annual rate of increase (AARC) of Measles immunization among children 12-23 months of age in low- and middle-income countries 208](#_Toc46135156)

[Table S56: Predicted average annual rate of increase (AARC) of Measles immunization among children 12-23 months of age in low- and middle-income countries by wealth quintiles 209](#_Toc46135157)

[Table S57: Predicted average annual rate of increase (AARC) of Measles immunization among children 12-23 months of age in low- and middle-income countries by place of residence 210](#_Toc46135158)

[Table S58: Predicted average annual rate of increase (AARC) of Measles immunization among children 12-23 months of age in low- and middle-income countries by education of mother 212](#_Toc46135159)

[Table S59: Predicted average annual rate of increase (AARC) of Measles immunization among children 12-23 months of age in low- and middle-income countries by age of mother 213](#_Toc46135160)

[Table S60: Predicted average annual rate of increase (AARC) of Measles immunization among children 12-23 months of age in low- and middle-income countries by child sex 214](#_Toc46135161)

[Table S61: Predicted average annual rate of increase (AARC) of oral rehydration therapy for diarrhoea treatment among children 0-59 months of age in low- and middle-income countries 215](#_Toc46135162)

[Table S62: Predicted average annual rate of increase (AARC) of oral rehydration therapy for diarrhoea treatment among children 0-59 months of age in low- and middle-income countries by wealth quintiles 216](#_Toc46135163)

[Table S63: Predicted average annual rate of increase (AARC) of oral rehydration therapy for diarrhoea treatment among children 0-59 months of age in low- and middle-income countries by place of residence 217](#_Toc46135164)

[Table S64: Predicted average annual rate of increase (AARC) of oral rehydration therapy for diarrhoea treatment among children 0-59 months of age in low- and middle-income countries by education of mother 218](#_Toc46135165)

[Table S65: Predicted average annual rate of increase (AARC) of oral rehydration therapy for diarrhoea treatment among children 0-59 months of age in low- and middle-income countries by age of mother 219](#_Toc46135166)

[Table S66: Predicted average annual rate of increase (AARC) of oral rehydration therapy for diarrhoea treatment among children 0-59 months of age in low- and middle-income countries by child sex 220](#_Toc46135167)

[Table S67: Predicted average annual rate of increase (AARC) of care seeking for acute respiratory infections among children 0-59 months of age in low- and middle-income countries 221](#_Toc46135168)

[Table S68: Predicted average annual rate of increase (AARC) of care seeking for acute respiratory infections among children 0-59 months of age in low- and middle-income countries by wealth quintiles 223](#_Toc46135169)

[Table S69: Predicted average annual rate of increase (AARC) of care seeking for acute respiratory infections among children 0-59 months of age in low- and middle-income countries by place of residence 224](#_Toc46135170)

[Table S70: Predicted average annual rate of increase (AARC) of care seeking for acute respiratory infections among children 0-59 months of age in low- and middle-income countries by education of mother 225](#_Toc46135171)

[Table S71: Predicted average annual rate of increase (AARC) of care seeking for acute respiratory infections among children 0-59 months of age in low- and middle-income countries by age of mother 226](#_Toc46135172)

[Table S72: Predicted average annual rate of increase (AARC) of care seeking for acute respiratory infections among children 0-59 months of age in low- and middle-income countries by child sex 227](#_Toc46135173)

**List of Figures**

[Figure S1: Progress and projections of demand for family planning satisfied with modern contraceptive methods (mDFPS) among women 15-49 years of age in low- and middle-income countries 39](#_Toc45827949)

[Figure S2: Progress and projections of antenatal care (ANC) visits among women 15-49 years of age in low- and middle-income countries 40](#_Toc45827950)

[Figure S3: Progress and projections of skilled birth attendance (SBA) among women 15-49 years of age in low- and middle-income countries 41](#_Toc45827951)

[Figure S4: Progress and projections of BCG immunization (BCG) among children 12-23 months of age in low- and middle-income countries 42](#_Toc45827952)

[Figure S5: Progress and projections of three doses of DPT immunization (DPT) among children 12-23 months of age in low- and middle-income countries 43](#_Toc45827953)

[Figure S6: Progress and projections of Measles immunization (Measles) among children 12-23 months of age in low- and middle-income countries 44](#_Toc45827954)

[Figure S7: Progress and projections of oral rehydration therapy (ORT) for diarrhoea treatment among children 0-59 months of age in low- and middle-income countries 45](#_Toc45827955)

[Figure S8: Progress and projections of care seeking for acute respiratory infections (ARI care) among children 0-59 months of age in low- and middle-income countries 46](#_Toc45827956)

[Figure S9: Predicted coverage (in %) of demand for family planning satisfied with modern contraceptive method (mDFPS) among women 15-49 years of age in low- and middle-income countries in 2030 47](#_Toc45827957)

[Figure S10: Predicted coverage (in %) of antenatal care (ANC) visits among women 15-49 years of age in low- and middle-income countries in 2030 48](#_Toc45827958)

[Figure S11: Predicted coverage (in %) of skilled birth attendance (SBA) among women 15-49 years of age in low- and middle-income countries in 2030 49](#_Toc45827959)

[Figure S12: Predicted coverage (in %) of BCG immunization (BCG) among children 12-23 months of age in low- and middle-income countries in 2030 50](#_Toc45827960)

[Figure S13: Predicted coverage (in %) of three doses of DPT immunization (DPT) among children 12-23 months of age in low- and middle-income countries in 2030 51](#_Toc45827961)

[Figure S14: Predicted coverage (in %) of Measles immunization (Measles) among children 12-23 months of age in low- and middle-income countries in 2030 52](#_Toc45827962)

[Figure S15: Predicted coverage (in %) of oral rehydration therapy (ORT) for diarrhoea treatment among children 0-59 months of age in low- and middle-income countries in 2030 53](#_Toc45827963)

[Figure S16: Predicted coverage (in %) of care seeking for acute respiratory infections (ARI care) among children 0-59 months of age in low- and middle-income countries in 2030 54](#_Toc45827964)

[Figure S17: Trends in predicted rural-urban gaps in composite coverage index (CCI) in low- and middle-income countries 55](#_Toc45827965)

[Figure S18: Trends in predicted below secondary-secondary+ education gaps in composite coverage index (CCI) in low- and middle-income countries 56](#_Toc45827966)

[Figure S19: Trends in predicted adolescent-adult gaps in composite coverage index (CCI) in low- and middle-income countries 57](#_Toc45827967)

[Figure S20: Trends in predicted poorest-richest gaps in the coverage of demand for family planning satisfied with modern contraceptive method (mDFPS) among women 15-49 years of age in low- and middle-income countries 58](#_Toc45827968)

[Figure S21: Trends in predicted rural-urban gaps in the coverage of demand for family planning satisfied with modern contraceptive method (mDFPS) among women 15-49 years of age in low- and middle-income countries 59](#_Toc45827969)

[Figure S22: Trends in predicted below secondary-secondary+ education gaps in the coverage of demand for family planning satisfied with modern contraceptive method (mDFPS) among women 15-49 years of age in low- and middle-income countries 60](#_Toc45827970)

[Figure S23: Trends in predicted adolescent-adult gaps in the coverage of demand for family planning satisfied with modern contraceptive method (mDFPS) among women 15-49 years of age in low- and middle-income countries 61](#_Toc45827971)

[Figure S24: Trends in predicted poorest-richest gaps in the coverage of antenatal care (ANC) visits among women 15-49 years of age in low- and middle-income countries 62](#_Toc45827972)

[Figure S25: Trends in predicted rural-urban gaps in the coverage of antenatal care (ANC) visits among women 15-49 years of age in low- and middle-income countries 63](#_Toc45827973)

[Figure S26: Trends in predicted below secondary-secondary+ education gaps in the coverage of antenatal care (ANC) visits among women 15-49 years of age in low- and middle-income countries 64](#_Toc45827974)

[Figure S27: Trends in predicted adolescent-adult gaps in the coverage of antenatal care (ANC) visits among women 15-49 years of age in low- and middle-income countries 65](#_Toc45827975)

[Figure S28: Trends in predicted poorest-richest gaps in the coverage of skilled birth attendance (SBA) among women 15-49 years of age in low- and middle-income countries 66](#_Toc45827976)

[Figure S29: Trends in predicted rural-urban gaps in the coverage of skilled birth attendance (SBA) among women 15-49 years of age in low- and middle-income countries 67](#_Toc45827977)

[Figure S30: Trends in predicted below secondary-secondary+ education gaps in the coverage of skilled birth attendance (SBA) among women 15-49 years of age in low- and middle-income countries 68](#_Toc45827978)

[Figure S31: Trends in predicted adolescent-adult gaps in the coverage of skilled birth attendance (SBA) among women 15-49 years of age in low- and middle-income countries 69](#_Toc45827979)

[Figure S32: Trends in predicted poorest-richest gaps in the coverage of BCG immunization (BCG) among children 12-23 months of age in low- and middle-income countries 70](#_Toc45827980)

[Figure S33: Trends in predicted rural-urban gaps in the coverage of BCG immunization (BCG) among children 12-23 months of age in low- and middle-income countries 71](#_Toc45827981)

[Figure S34: Trends in predicted mother’s below secondary-secondary+ education gaps in the coverage of BCG immunization (BCG) among children 12-23 months of age in low- and middle-income countries 72](#_Toc45827982)

[Figure S35: Trends in predicted adolescent-adult motherhood gaps in the coverage of BCG immunization (BCG) among children 12-23 months of age in low- and middle-income countries 73](#_Toc45827983)

[Figure S36: Trends in predicted male-female gaps in the coverage of BCG immunization (BCG) among children 12-23 months of age in low- and middle-income countries 74](#_Toc45827984)

[Figure S37: Trends in predicted poorest-richest gaps in the coverage of three doses of DPT immunization (DPT) among children 12-23 months of age in low- and middle-income countries 75](#_Toc45827985)

[Figure S38: Trends in predicted rural-urban gaps in the coverage of three doses of DPT immunization (DPT) among children 12-23 months of age in low- and middle-income countries 76](#_Toc45827986)

[Figure S39: Trends in predicted mother’s below secondary-secondary+ education gaps in the coverage of three doses of DPT immunization (DPT) among children 12-23 months of age in low- and middle-income countries 77](#_Toc45827987)

[Figure S40: Trends in predicted adolescent-adult motherhood gaps in the coverage of three doses of DPT immunization (DPT) among children 12-23 months of age in low- and middle-income countries 78](#_Toc45827988)

[Figure S41: Trends in predicted male-female gaps in the coverage of three doses of DPT immunization (DPT) among children 12-23 months of age in low- and middle-income countries 79](#_Toc45827989)

[Figure S42: Trends in predicted poorest-richest gaps in the coverage of Measles immunization (Measles) among children 12-23 months of age in low- and middle-income countries 80](#_Toc45827990)

[Figure S43: Trends in predicted rural-urban gaps in the coverage of Measles immunization (Measles) among children 12-23 months of age in low- and middle-income countries 81](#_Toc45827991)

[Figure S44: Trends in predicted mother’s below secondary-secondary+ education gaps in the coverage of Measles immunization (Measles) among children 12-23 months of age in low- and middle-income countries 82](#_Toc45827992)

[Figure S45: Trends in predicted adolescent-adult motherhood gaps in the coverage of Measles immunization (Measles) among children 12-23 months of age in low- and middle-income countries 83](#_Toc45827993)

[Figure S46: Trends in predicted male-female gaps in the coverage of Measles immunization (Measles) among children 12-23 months of age in low- and middle-income countries 84](#_Toc45827994)

[Figure S47: Trends in predicted poorest-richest gaps in the coverage of oral rehydration therapy (ORT) for diarrhoea treatment among children 0-59 months of age in low- and middle-income countries 85](#_Toc45827995)

[Figure S48: Trends in predicted rural-urban gaps in the coverage of oral rehydration therapy (ORT) for diarrhoea treatment among children 0-59 months of age in low- and middle-income countries 86](#_Toc45827996)

[Figure S49: Trends in predicted mother’s below secondary-secondary+ education gaps in the coverage of oral rehydration therapy (ORT) for diarrhoea treatment among children 0-59 months of age in low- and middle-income countries 87](#_Toc45827997)

[Figure S50: Trends in predicted adolescent-adult motherhood gaps in the coverage of oral rehydration therapy (ORT) for diarrhoea treatment among children 0-59 months of age in low- and middle-income countries 88](#_Toc45827998)

[Figure S51: Trends in predicted male-female gaps in the coverage of oral rehydration therapy (ORT) for diarrhoea treatment among children 0-56 months of age in low- and middle-income countries 89](#_Toc45827999)

[Figure S52: Trends in predicted poorest-richest gaps in the coverage of care seeking for acute respiratory infections (ARI care) among children 0-59 months of age in low- and middle-income countries 90](#_Toc45828000)

[Figure S53: Trends in predicted rural-urban gaps in the coverage of care seeking for acute respiratory infections (ARI care) among children 0-59 months of age in low- and middle-income countries 91](#_Toc45828001)

[Figure S54: Trends in predicted mothers below secondary-secondary+ education gaps in the coverage of care seeking for acute respiratory infections (ARI care) among children 0-59 months of age in low- and middle-income countries 92](#_Toc45828002)

[Figure S55: Trends in predicted adolescent-adult motherhood gaps in the coverage of care seeking for acute respiratory infections (ARI care) among children 0-59 months of age in low- and middle-income countries 93](#_Toc45828003)

[Figure S56: Trends in predicted male-female gaps in the coverage of care seeking for acute respiratory infections (ARI care) among children 0-59 months of age in low- and middle-income countries 94](#_Toc45828004)

[Figure S57: Projected gaps in the coverage of demand for family planning satisfied with modern contraceptive method (mDFPS) among women 15-49 years of age in low- and middle-income countries in 2030 by wealth quintiles 95](#_Toc45828005)

[Figure S58: Projected gaps in the coverage of demand for family planning satisfied with modern contraceptive method (mDFPS) among women 15-49 years of age in low- and middle-income countries in 2030 by place of residence 96](#_Toc45828006)

[Figure S59: Projected gaps in the coverage of demand for family planning satisfied with modern contraceptive method (mDFPS) among women 15-49 years of age in low- and middle-income countries in 2030 by education 97](#_Toc45828007)

[Figure S60: Projected gaps in the coverage of demand for family planning satisfied with modern contraceptive method (mDFPS) among women 15-49 years of age in low- and middle-income countries in 2030 by age 98](#_Toc45828008)

[Figure S61: Projected gaps in the coverage of antenatal care (ANC) visits among women 15-49 years of age in low- and middle-income countries in 2030 by wealth quintiles 99](#_Toc45828009)

[Figure S62: Projected gaps in the coverage of antenatal care (ANC) visits among women 15-49 years of age in low- and middle-income countries in 2030 by place of residence 100](#_Toc45828010)

[Figure S63: Projected gaps in the coverage of antenatal care (ANC) visits among women 15-49 years of age in low- and middle-income countries in 2030 by education 101](#_Toc45828011)

[Figure S64: Projected gaps in the coverage of antenatal care (ANC) visits among women 15-49 years of age in low- and middle-income countries in 2030 by age 102](#_Toc45828012)

[Figure S65: Projected gaps in the coverage of skilled birth attendance (SBA) among women 15-49 years of age in low- and middle-income countries in 2030 by wealth quintiles 103](#_Toc45828013)

[Figure S66: Projected gaps in the coverage of skilled birth attendance (SBA) among women 15-49 years of age in low- and middle-income countries in 2030 by place of residence 104](#_Toc45828014)

[Figure S67: Projected gaps in the coverage of skilled birth attendance (SBA) among women 15-49 years of age in low- and middle-income countries in 2030 by education 105](#_Toc45828015)

[Figure S68: Projected gaps in the coverage of skilled birth attendance (SBA) among women 15-49 years of age in low- and middle-income countries in 2030 by age 106](#_Toc45828016)

[Figure S69: Projected gaps in the coverage of BCG immunization (BCG) among children 12-23 months of age in low- and middle-income countries in 2030 by wealth quintiles 107](#_Toc45828017)

[Figure S70: Projected gaps in the coverage of BCG immunization (BCG) among children 12-23 months of age in low- and middle-income countries in 2030 by place of residence 108](#_Toc45828018)

[Figure S71: Projected gaps in the coverage of BCG immunization (BCG) among children 12-23 months of age in low- and middle-income countries in 2030 by mother’s education 109](#_Toc45828019)

[Figure S72: Projected gaps in the coverage of BCG immunization (BCG) among children 12-23 months of age in low- and middle-income countries in 2030 by mother’s age 110](#_Toc45828020)

[Figure S73: Projected gaps in the coverage of three doses of DPT immunization (DPT) among children 12-23 months of age in low- and middle-income countries in 2030 by wealth quintiles 111](#_Toc45828021)

[Figure S74: Projected gaps in the coverage of three doses of DPT immunization (DPT) among children 12-23 months of age in low- and middle-income countries in 2030 by place of residence 112](#_Toc45828022)

[Figure S75: Projected gaps in the coverage of three doses of DPT immunization (DPT) among children 12-23 months of age in low- and middle-income countries in 2030 by mother’s education 113](#_Toc45828023)

[Figure S76: Projected gaps in the coverage of three doses of DPT immunization (DPT) among children 12-23 months of age in low- and middle-income countries in 2030 by mother’s age 114](#_Toc45828024)

[Figure S77: Projected gaps in the coverage of Measles immunization (Measles) among children 12-23 months of age in low- and middle-income countries in 2030 by wealth quintiles 115](#_Toc45828025)

[Figure S78: Projected gaps in the coverage of Measles immunization (Measles) among children 12-23 months of age in low- and middle-income countries in 2030 by place of residence 116](#_Toc45828026)

[Figure S79: Projected gaps in the coverage of Measles immunization (Measles) among children 12-23 months of age in low- and middle-income countries in 2030 by mother’s education 117](#_Toc45828027)

[Figure S80: Projected gaps in the coverage of Measles immunization (Measles) among children 12-23 months of age in low- and middle-income countries in 2030 by mother’s age 118](#_Toc45828028)

[Figure S81: Projected gaps in the coverage of oral rehydration therapy (ORT) for diarrhoea treatment among children 0-59 months of age in low- and middle-income countries in 2030 by wealth quintiles 119](#_Toc45828029)

[Figure S82: Projected gaps in the coverage of oral rehydration therapy (ORT) for diarrhoea treatment among children 0-59 months of age in low- and middle-income countries in 2030 by place of residence 120](#_Toc45828030)

[Figure S83: Projected gaps in the coverage of oral rehydration therapy (ORT) for diarrhoea treatment among children 0-59 months of age in low- and middle-income countries in 2030 by mother’s education 121](#_Toc45828031)

[Figure S84: Projected gaps in the coverage of oral rehydration therapy (ORT) for diarrhoea treatment among children 0-59 months of age in low- and middle-income countries in 2030 by mother’s age 122](#_Toc45828032)

[Figure S85: Projected gaps in the coverage of care seeking for acute respiratory infections (ARI care) among children 0-59 months of age in low- and middle-income countries in 2030 by wealth quintiles 123](#_Toc45828033)

[Figure S86: Projected gaps in the coverage of care seeking for acute respiratory infections (ARI care) among children 0-59 months of age in low- and middle-income countries in 2030 by place of residence 124](#_Toc45828034)

[Figure S87: Projected gaps in the coverage of care seeking for acute respiratory infections (ARI care) among children 0-59 months of age in low- and middle-income countries in 2030 by mother’s education 125](#_Toc45828035)

[Figure S88: Projected gaps in the coverage of care seeking for acute respiratory infections (ARI care) among children 0-59 months of age in low- and middle-income countries in 2030 by mother’s age 126](#_Toc45828036)

[Figure S89: Projected gaps in the coverage of BCG immunization (BCG) among children 12-23 months of age in low- and middle-income countries in 2030 by child sex 127](#_Toc45828037)

[Figure S90: Projected gaps in the coverage of three doses of DPT immunization (DPT) among children 12-23 months of age in low- and middle-income countries in 2030 by child sex 128](#_Toc45828038)

[Figure S91: Projected gaps in the coverage of Measles immunization (Measles) among children 12-23 months of age in low- and middle-income countries in 2030 by child sex 129](#_Toc45828039)

[Figure S92: Projected gaps in the coverage of oral rehydration therapy (ORT) for diarrhoea treatment among children 0-59 months of age in low- and middle-income countries in 2030 by child sex 130](#_Toc45828040)

[Figure S93: Projected gaps in the coverage of care seeking for acute respiratory infections (ARI care) among children 0-59 months of age in low- and middle-income countries in 2030 by child sex 131](#_Toc45828041)

# Supplementary

Table S1: Countries with survey year and sample size for all indicators

| **Country** | **Survey year** | **mDFPS** | **ANC** | **SBA** | **BCG** | **DPT** | **Measles** | **ORT** | **ARI care** |
| --- | --- | --- | --- | --- | --- | --- | --- | --- | --- |
| **South and Southeast Asia** |  |  |  |  |  |  |  |  |  |
| Afghanistan | 2010 | 39,855 | 2,766 | 13,017 | N/A | N/A | N/A | N/A | N/A |
| Afghanistan | 2015 | 13,144 | 19,801 | 32,557 | 5,818 | 5,818 | 5,816 | 7,838 | 3,923 |
| Bangladesh | 1994 | 5,955 | 3,569 | 3,857 | 1,145 | 1,144 | 1,142 | 444 | 847 |
| Bangladesh | 1997 | 5,740 | 3,289 | 3,610 | 1,080 | 1,080 | 1,077 | 402 | 687 |
| Bangladesh | 2000 | 6,935 | 3,745 | 4,127 | 1,303 | 1,303 | 1,303 | 393 | 1,072 |
| Bangladesh | 2004 | 7,642 | 3,735 | 4,093 | 1,247 | 1,247 | 1,247 | 483 | 1,255 |
| Bangladesh | 2007 | 7,317 | 3,383 | 3,643 | 1,161 | 1,161 | 1,161 | 559 | 768 |
| Bangladesh | 2011 | 12,399 | 4,661 | 4,964 | 1,546 | 1,546 | 1,546 | 395 | 1,376 |
| Bangladesh | 2014 | 12,436 | 4,494 | 4,723 | 1,557 | 1,557 | 1,556 | 368 | 1,065 |
| Cambodia | 2000 | 5,342 | 4,386 | 5,005 | 1,329 | 1,329 | 1,328 | 1,575 | 1,522 |
| Cambodia | 2005 | 6,708 | 4,495 | 5,013 | 1,585 | 1,585 | 1,585 | 1,449 | 1,265 |
| Cambodia | 2010 | 7,702 | 6,448 | 8,232 | 1,619 | 1,619 | 1,619 | 1,132 | 830 |
| Cambodia | 2014 | 8,019 | 4,077 | 4,391 | 1,441 | 1,441 | 1,441 | 841 | 560 |
| India | 1993 | 52,692 | 31,941 | 36,821 | 12,535 | 12,535 | 12,535 | 4,806 | 2,972 |
| India | 1999 | 55,344 | 28,862 | 32,927 | 10,207 | 10,208 | 10,209 | 6,083 | 5,936 |
| India | 2006 | 62,601 | 26,552 | 30,402 | 9,582 | 9,582 | 9,582 | 4,408 | 4,158 |
| India | 2016 | 332,076 | 135,357 | 153,388 | 49,284 | 49,284 | 49,284 | 22,486 | 13,555 |
| Indonesia | 1991 | 13,931 | 8,370 | 9,443 | 3,153 | 3,148 | 3,113 | 1,397 | 1,467 |
| Indonesia | 1994 | 17,673 | 9,724 | 10,771 | 3,237 | 3,236 | 3,230 | 1,780 | 1,631 |
| Indonesia | 1997 | 18,583 | 9,617 | 10,543 | 3,329 | 3,329 | 3,329 | 1,719 | 1,553 |
| Indonesia | 2003 | 20,198 | 9,230 | 9,940 | 3,097 | 3,097 | 3,097 | 1,516 | 1,340 |
| Indonesia | 2007 | 22,360 | 10,302 | 11,172 | 3,487 | 3,487 | 3,487 | 2,519 | 2,175 |
| Indonesia | 2012 | 23,799 | 10,168 | 10,912 | 3,502 | 3,502 | 3,502 | 2,483 | 1,919 |
| Indonesia | 2017 | 25,217 | 9,976 | 10,588 | 3,535 | 3,531 | 3,532 | 2,413 | 1,710 |
| Maldives | 2009 | 4,226 | 2,293 | 2,443 | 843 | 843 | 843 | 185 | 508 |
| Maldives | 2017 | 3,877 | 1,671 | 1,769 | 587 | 587 | 587 | 126 | 102 |
| Myanmar | 2016 | 5,249 | 2,628 | 2,867 | 915 | 915 | 915 | 548 | 372 |
| Nepal | 1996 | 4,850 | 3,775 | 4,412 | 1,402 | 1,402 | 1,402 | 1,176 | 1,412 |
| Nepal | 2001 | 5,626 | 3,553 | 4,050 | 1,299 | 1,299 | 1,295 | 1,285 | 1,475 |
| Nepal | 2006 | 5,934 | 3,016 | 3,396 | 1,063 | 1,063 | 1,063 | 659 | 474 |
| Nepal | 2011 | 7,365 | 2,795 | 3,102 | 945 | 945 | 945 | 677 | 487 |
| Nepal | 2016 | 7,655 | 2,727 | 2,946 | 1,009 | 1,009 | 1,009 | 336 | 252 |
| Pakistan | 1991 | 2,860 | 3,274 | 3,934 | 1,287 | 1,286 | 1,276 | 780 | 860 |
| Pakistan | 2007 | 5,226 | 4,457 | 5,453 | 1,541 | 1,541 | 1,541 | 1,873 | 1,590 |
| Pakistan | 2013 | 7,413 | 5,686 | 6,849 | 2,039 | 2,039 | 2,039 | 2,291 | 2,129 |
| Pakistan | 2018 | 7,461 | 6,232 | 7,478 | 2,331 | 2,331 | 2,331 | 2,084 | 2,079 |
| Philippines | 1993 | 6,479 | 4,471 | 5,579 | 1,879 | 1,879 | 1,860 | 895 | 773 |
| Philippines | 1998 | 6,299 | 3,958 | 4,851 | 1,554 | 1,554 | 1,554 | 604 | 1,065 |
| Philippines | 2003 | 6,352 | 3,587 | 4,237 | 1,368 | 1,368 | 1,367 | 748 | 728 |
| Philippines | 2008 | 6,425 | 3,422 | 3,945 | 1,320 | 1,320 | 1,320 | 571 | 654 |
| Philippines | 2013 | 7,377 | 3,767 | 4,257 | 1,423 | 1,423 | 1,423 | 550 | 733 |
| Philippines | 2017 | 11,202 | 5,374 | 6,026 | 1,996 | 1,996 | 1,996 | 651 | 295 |
| Timor-Leste | 2010 | 4,252 | 5,999 | 9,806 | 1,803 | 1,803 | 1,803 | 1,388 | 389 |
| Timor-Leste | 2016 | 4,783 | 3,725 | 4,355 | 1,443 | 1,443 | 1,443 | 694 | 201 |
| Vietnam | 1997 | 4,468 | 1,633 | 1,775 | 615 | 615 | 615 | 170 | 237 |
| Vietnam | 2002 | 4,557 | 1,220 | 1,316 | 467 | 466 | 467 | 138 | 255 |
| **Central Asia** |  |  |  |  |  |  |  |  |  |
| Kazakhstan | 1995 | 2,030 | 732 | 1,692 | 588 | 588 | 588 | 232 | 82 |
| Kazakhstan | 1999 | 2,796 | 682 | 1,530 | 506 | 506 | 506 | 332 | 78 |
| Kyrgyz Republic | 1997 | 1,991 | 984 | 2,252 | 742 | 742 | 742 | 370 | 74 |
| Kyrgyz Republic | 2012 | 3,395 | 2,365 | 5,496 | 1,758 | 1,758 | 1,754 | 432 | 188 |
| Tajikistan | 2012 | 3,937 | 2,697 | 6,348 | 2,046 | 2,048 | 2,042 | 1,436 | 208 |
| Tajikistan | 2017 | 4,603 | 3,137 | 7,436 | 2,540 | 2,540 | 2,540 | 1,596 | 198 |
| Uzbekistan | 1996 | 2,189 | 1,151 | 2,648 | 876 | 876 | 876 | 154 | 52 |
| **Sub-Saharan Africa** |  |  |  |  |  |  |  |  |  |
| Angola | 2007 | 2,972 | 1,381 | 665 | N/A | N/A | N/A | N/A | N/A |
| Angola | 2011 | 8,589 | N/A | N/A | N/A | N/A | N/A | N/A | N/A |
| Angola | 2016 | 5,694 | 7,413 | 8,869 | 2,842 | 2,842 | 2,842 | 1,884 | 605 |
| Benin | 1996 | 2,124 | 2,655 | 2,996 | 895 | 894 | 894 | 736 | 442 |
| Benin | 2001 | 2,519 | 2,851 | 3,232 | 939 | 939 | 935 | 644 | 568 |
| Benin | 2006 | 6,894 | 8,667 | 9,773 | 3,037 | 3,037 | 3,037 | 1,383 | 1,409 |
| Benin | 2012 | 6,631 | 7,042 | 7,872 | 2,532 | 2,532 | 2,532 | 808 | 421 |
| Benin | 2018 | 6,469 | 7,202 | 8,202 | 2,521 | 2,521 | 2,521 | 1,338 | 696 |
| Burkina Faso | 1993 | 2,826 | 3,095 | 3,423 | 1,122 | 1,122 | 1,119 | 1,009 | 571 |
| Burkina Faso | 1999 | 2,475 | 3,166 | 3,470 | 1,000 | 1,001 | 1,001 | 1,012 | 675 |
| Burkina Faso | 2003 | 4,747 | 5,738 | 6,206 | 1,823 | 1,823 | 1,817 | 1,873 | 819 |
| Burkina Faso | 2010 | 6,094 | 10,364 | 15,044 | 2,791 | 2,791 | 2,791 | 2,021 | 591 |
| Burkina Faso | 2014 | 8,111 | N/A | N/A | N/A | N/A | N/A | N/A | N/A |
| Burundi | 2010 | 3,126 | 4,916 | 7,742 | 1,524 | 1,524 | 1,524 | 1,784 | 2,087 |
| Burundi | 2012 | 5,149 | 2,230 | N/A | N/A | N/A | N/A | N/A | N/A |
| Burundi | 2017 | 6,130 | 6,951 | 7,922 | 2,645 | 2,645 | 2,645 | 2,659 | 1,543 |
| Cameroon | 1991 | 1,600 | 1,710 | 2,041 | 690 | 690 | 689 | 506 | 260 |
| Cameroon | 1998 | 2,370 | 2,005 | 2,295 | 703 | 703 | 702 | 390 | 418 |
| Cameroon | 2004 | 4,575 | 4,238 | 4,922 | 1,542 | 1,542 | 1,541 | 1,110 | 762 |
| Cameroon | 2011 | 6,489 | 6,181 | 7,185 | 2,286 | 2,284 | 2,279 | 2,052 | 1,657 |
| Central African Republic | 1995 | 1,702 | 2,422 | 2,814 | 805 | 805 | 805 | 580 | 702 |
| Chad | 1997 | 1,532 | 3,786 | 4,388 | 1,179 | 1,178 | 1,178 | 1,376 | 809 |
| Chad | 2004 | 1,618 | 2,881 | 3,316 | 901 | 901 | 901 | 1,185 | 208 |
| Chad | 2015 | 3,987 | 9,088 | 10,519 | 2,880 | 2,873 | 2,875 | 3,258 | 1,734 |
| Comoros | 1996 | 1,045 | 934 | 1,145 | 368 | 368 | 367 | 244 | 235 |
| Comoros | 2012 | 1,872 | 1,607 | 1,950 | 630 | 630 | 630 | 469 | 197 |
| Congo | 2005 | 4,254 | 2,745 | 3,065 | 901 | 901 | 901 | 624 | 322 |
| Congo | 2012 | 6,335 | 5,087 | 5,785 | 1,884 | 1,884 | 1,884 | 1,527 | 807 |
| Congo DR | 2007 | 4,122 | 4,544 | 5,519 | 1,632 | 1,632 | 1,632 | 1,277 | 1,224 |
| Congo DR | 2014 | 7,501 | 9,575 | 11,382 | 3,443 | 3,442 | 3,440 | 2,800 | 2,078 |
| Cote d'Ivoire | 1994 | 2,182 | 3,559 | 3,998 | 1,166 | 1,166 | 1,164 | 774 | 293 |
| Cote d'Ivoire | 1999 | 1,389 | 1,131 | 1,258 | 390 | 390 | 390 | 335 | 270 |
| Cote d'Ivoire | 2005 | 9,686 | 1,975 | 2,186 | N/A | N/A | N/A | N/A | N/A |
| Cote d'Ivoire | 2012 | 4,237 | 4,233 | 4,788 | 1,417 | 1,417 | 1,417 | 1,264 | 597 |
| Ethiopia | 2000 | 4,435 | 5,625 | 6,330 | 1,844 | 1,843 | 1,843 | 2,150 | 2,199 |
| Ethiopia | 2005 | 4,347 | 5,119 | 5,765 | 1,697 | 1,697 | 1,697 | 1,538 | 964 |
| Ethiopia | 2011 | 5,510 | 5,925 | 6,701 | 1,927 | 1,927 | 1,927 | 1,606 | 1,237 |
| Ethiopia | 2016 | 5,312 | 5,491 | 6,213 | 1,953 | 1,953 | 1,953 | 1,086 | 787 |
| Gabon | 2000 | 3,387 | 2,347 | 2,741 | 889 | 889 | 889 | 599 | 509 |
| Gabon | 2012 | 4,438 | 3,278 | 3,863 | 1,197 | 1,197 | 1,197 | 974 | 830 |
| Gambia | 2013 | 2,503 | 4,499 | 5,138 | 1,648 | 1,648 | 1,648 | 1,334 | 655 |
| Ghana | 1993 | 2,089 | 1,978 | 2,199 | 686 | 685 | 686 | 401 | 208 |
| Ghana | 1998 | 2,053 | 1,784 | 1,961 | 651 | 651 | 651 | 566 | 428 |
| Ghana | 2003 | 2,567 | 2,085 | 2,300 | 735 | 735 | 735 | 576 | 347 |
| Ghana | 2008 | 2,116 | 1,621 | 1,826 | 569 | 569 | 569 | 545 | 301 |
| Ghana | 2014 | 4,034 | 3,242 | 3,618 | 1,127 | 1,127 | 1,127 | 670 | 364 |
| Ghana | 2016 | 5,150 | 1,785 | N/A | N/A | N/A | N/A | N/A | N/A |
| Guinea | 1999 | 2,011 | 3,079 | 3,402 | 919 | 918 | 915 | 1,039 | 776 |
| Guinea | 2005 | 2,343 | 3,542 | 3,926 | 1,112 | 1,112 | 1,111 | 821 | 414 |
| Guinea | 2012 | 2,591 | 3,900 | 4,219 | 1,300 | 1,301 | 1,301 | 1,068 | 792 |
| Kenya | 1993 | 3,738 | 3,073 | 3,644 | 1,212 | 1,211 | 1,205 | 736 | 1,005 |
| Kenya | 1998 | 3,977 | 3,054 | 3,518 | 1,127 | 1,127 | 1,125 | 535 | 619 |
| Kenya | 2003 | 3,755 | 3,101 | 3,633 | 1,099 | 1,099 | 1,099 | 860 | 1,008 |
| Kenya | 2009 | 4,017 | 3,188 | 3,733 | 1,119 | 1,119 | 1,119 | 943 | 710 |
| Kenya | 2014 | 14,107 | 11,005 | 12,423 | 4,051 | 4,051 | 4,047 | 2,937 | 3,262 |
| Kenya | 2015 | 5,394 | 1,934 | N/A | N/A | N/A | N/A | N/A | N/A |
| Lesotho | 2004 | 3,522 | 2,132 | 2,284 | 673 | 672 | 673 | 472 | 663 |
| Lesotho | 2009 | 3,907 | 2,302 | 2,509 | 797 | 797 | 797 | 417 | 458 |
| Lesotho | 2014 | 4,042 | 1,924 | 2,027 | 655 | 655 | 655 | 327 | 260 |
| Liberia | 2007 | 3,234 | 3,049 | 3,476 | 996 | 996 | 996 | 1,062 | 695 |
| Liberia | 2009 | 4,397 | 2,174 | N/A | N/A | N/A | N/A | N/A | N/A |
| Liberia | 2011 | 3,939 | 1,783 | N/A | N/A | N/A | N/A | N/A | N/A |
| Liberia | 2013 | 4,576 | 4,065 | 4,595 | 1,433 | 1,433 | 1,433 | 1,666 | 904 |
| Liberia | 2016 | 1,401 | 1,622 | N/A | 573 | N/A | 573 | N/A | N/A |
| Madagascar | 1992 | 1,885 | 2,651 | 3,241 | 1,089 | 1,087 | 1,086 | 560 | 737 |
| Madagascar | 1997 | 2,699 | 3,116 | 3,656 | 1,124 | 1,124 | 1,119 | 882 | 752 |
| Madagascar | 2004 | 3,452 | 2,815 | 3,206 | 1,061 | 1,060 | 1,061 | 461 | 443 |
| Madagascar | 2009 | 8,083 | 6,455 | 7,415 | 2,183 | 2,183 | 2,183 | 988 | 604 |
| Madagascar | 2011 | 8,169 | 3,203 | N/A | N/A | N/A | N/A | N/A | N/A |
| Madagascar | 2013 | 8,045 | 2,939 | N/A | N/A | N/A | N/A | N/A | N/A |
| Madagascar | 2016 | 10,655 | 3,773 | N/A | N/A | N/A | N/A | N/A | N/A |
| Malawi | 1992 | 1,950 | 2,377 | 2,813 | 925 | 924 | 920 | 811 | 534 |
| Malawi | 2000 | 6,443 | 6,605 | 7,574 | 2,216 | 2,215 | 2,213 | 1,753 | 2,672 |
| Malawi | 2004 | 5,816 | 6,007 | 6,788 | 2,233 | 2,233 | 2,223 | 2,208 | 1,865 |
| Malawi | 2010 | 12,741 | 13,776 | 19,967 | 3,808 | 3,808 | 3,808 | 3,096 | 2,764 |
| Malawi | 2012 | 2,906 | 1,295 | N/A | N/A | N/A | N/A | N/A | N/A |
| Malawi | 2014 | 2,897 | 1,215 | N/A | N/A | N/A | N/A | N/A | N/A |
| Malawi | 2016 | 14,864 | 9,464 | 10,182 | 3,245 | 3,245 | 3,245 | 3,391 | 1,640 |
| Mali | 1996 | 3,199 | 5,161 | 6,021 | 1,583 | 1,581 | 1,579 | 1,374 | 804 |
| Mali | 2001 | 4,482 | 6,805 | 7,901 | 2,256 | 2,255 | 2,241 | 2,179 | 1,187 |
| Mali | 2006 | 4,560 | 7,458 | 8,537 | 2,558 | 2,558 | 2,557 | 1,439 | 699 |
| Mali | 2013 | 3,468 | 5,338 | 6,016 | 1,844 | 1,844 | 1,844 | 844 | 324 |
| Mali | 2015 | 7,758 | N/A | N/A | N/A | N/A | N/A | N/A | N/A |
| Mozambique | 1997 | 2,711 | 3,730 | 4,109 | 1,218 | 1,215 | 1,214 | 795 | 479 |
| Mozambique | 2003 | 5,317 | 5,534 | 6,164 | 1,875 | 1,875 | 1,875 | 1,197 | 925 |
| Mozambique | 2011 | 4,978 | 6,162 | 6,937 | 2,225 | 2,225 | 2,225 | 1,069 | 495 |
| Mozambique | 2015 | 3,398 | 2,910 | 2,838 | 1,029 | 1,029 | 1,029 | 547 | 759 |
| Namibia | 1992 | 1,933 | 2,200 | 2,531 | 842 | 841 | 837 | 838 | 736 |
| Namibia | 2000 | 3,890 | 2,226 | 2,466 | 805 | 804 | 803 | 477 | 606 |
| Namibia | 2007 | 5,794 | 2,945 | 3,240 | 1,020 | 1,018 | 1,020 | 572 | 385 |
| Namibia | 2013 | 5,765 | 2,876 | 3,152 | 991 | 989 | 991 | 801 | 592 |
| Niger | 1992 | 1,441 | 3,452 | 4,125 | 1,257 | 1,258 | 1,258 | 1,419 | 546 |
| Niger | 1998 | 1,867 | 4,082 | 4,791 | 1,385 | 1,386 | 1,381 | 1,543 | 594 |
| Niger | 2006 | 2,457 | 4,898 | 5,598 | 1,674 | 1,674 | 1,674 | 1,657 | 1,166 |
| Niger | 2012 | 3,167 | 6,427 | 7,515 | 2,151 | 2,151 | 2,151 | 1,589 | 823 |
| Nigeria | 1990 | 2,316 | 4,005 | 4,717 | 1,483 | 1,488 | 1,480 | 1,115 | 437 |
| Nigeria | 2003 | 2,082 | 3,149 | 3,646 | 1,013 | 1,012 | 1,007 | 924 | 503 |
| Nigeria | 2008 | 9,832 | 14,794 | 17,063 | 5,012 | 5,006 | 4,996 | 2,577 | 1,166 |
| Nigeria | 2010 | 6,344 | 3,602 | N/A | N/A | N/A | N/A | N/A | N/A |
| Nigeria | 2013 | 11,464 | 16,426 | 18,869 | 5,834 | 5,834 | 5,834 | 2,937 | 1,122 |
| Nigeria | 2015 | 8,034 | 3,374 | N/A | N/A | N/A | N/A | N/A | N/A |
| Rwanda | 1992 | 2,423 | 2,878 | 3,315 | 1,093 | 1,093 | 1,093 | 1,056 | 1,514 |
| Rwanda | 2000 | 2,803 | 3,937 | 4,674 | 1,309 | 1,308 | 1,306 | 1,138 | 1,430 |
| Rwanda | 2005 | 3,452 | 4,542 | 5,497 | 1,624 | 1,624 | 1,624 | 1,090 | 1,327 |
| Rwanda | 2008 | 3,011 | 2,864 | 3,302 | 1,174 | 1,174 | 1,174 | 680 | 873 |
| Rwanda | 2010 | 5,631 | 6,328 | 9,002 | 1,596 | 1,596 | 1,596 | 1,109 | 835 |
| Rwanda | 2013 | 5,135 | 1,675 | N/A | N/A | N/A | N/A | N/A | N/A |
| Rwanda | 2015 | 5,813 | 4,416 | 4,855 | 1,537 | 1,537 | 1,537 | 903 | 870 |
| Sao Tome and Principe | 2009 | 1,518 | 1,101 | 1,201 | 377 | 377 | 377 | 227 | 203 |
| Senegal | 1993 | 1,809 | 2,904 | 3,360 | 1,059 | 1,059 | 1,055 | 1,041 | 710 |
| Senegal | 1997 | 3,070 | 3,852 | 4,405 | N/A | N/A | N/A | 986 | N/A |
| Senegal | 2005 | 4,509 | 5,877 | 6,862 | 2,138 | 2,136 | 2,135 | 2,119 | 1,206 |
| Senegal | 2006 | 6,655 | 2,631 | N/A | N/A | N/A | N/A | N/A | N/A |
| Senegal | 2009 | 19,441 | 7,963 | N/A | N/A | N/A | N/A | N/A | N/A |
| Senegal | 2011 | 4,747 | 6,652 | 7,617 | 2,377 | 2,377 | 2,377 | 2,194 | 1,262 |
| Senegal | 2013 | 2,826 | 3,666 | 4,183 | 1,329 | 1,329 | 1,329 | 972 | 402 |
| Senegal | 2014 | 2,864 | 3,617 | 4,135 | 1,333 | 1,333 | 1,333 | 1,272 | 211 |
| Senegal | 2015 | 3,056 | 3,735 | 4,234 | 1,310 | 1,310 | 1,310 | 1,359 | 343 |
| Senegal | 2016 | 3,028 | 3,538 | 8,238 | 2,633 | 2,633 | 2,633 | 2,421 | 665 |
| Senegal | 2017 | 5,864 | 6,474 | 7,231 | 2,386 | 2,386 | 2,386 | 2,204 | 889 |
| Sierra Leone | 2008 | 2,738 | 3,167 | 3,533 | 1,032 | 1,032 | 1,032 | 579 | 482 |
| Sierra Leone | 2013 | 7,139 | 6,407 | 7,095 | 2,090 | 2,090 | 2,090 | 1,204 | 1,185 |
| Sierra Leone | 2016 | 8,501 | 3,451 | N/A | N/A | N/A | N/A | N/A | N/A |
| South Africa | 1998 | 7,286 | 2,884 | 3,091 | 971 | 971 | 968 | 631 | 855 |
| South Africa | 2016 | 5,229 | 1,951 | 2,073 | 664 | 664 | 664 | 349 | 149 |
| Sudan | 1990 | 1,399 | 3,301 | 3,940 | 896 | 888 | 832 | 1,808 | N/A |
| Swaziland | 2007 | 2,741 | 1,569 | 1,744 | 538 | 538 | 538 | 347 | 369 |
| Tanzania | 1992 | 2,673 | 4,411 | 5,094 | 1,772 | 1,772 | 1,764 | 934 | 575 |
| Tanzania | 1996 | 3,165 | 3,680 | 4,175 | 1,296 | 1,296 | 1,284 | 843 | 786 |
| Tanzania | 1999 | 1,593 | 1,710 | 1,963 | 561 | 560 | 559 | 348 | 412 |
| Tanzania | 2005 | 4,117 | 4,560 | 5,290 | 1,613 | 1,613 | 1,613 | 1,016 | 661 |
| Tanzania | 2008 | 16,318 | 3,969 | N/A | N/A | N/A | N/A | N/A | N/A |
| Tanzania | 2010 | 4,392 | 5,358 | 8,023 | 1,549 | 1,549 | 1,549 | 1,007 | 528 |
| Tanzania | 2012 | 19,319 | 4,627 | 4,627 | N/A | N/A | N/A | N/A | N/A |
| Tanzania | 2016 | 6,157 | 5,509 | 6,284 | 2,137 | 2,137 | 2,137 | 1,121 | 466 |
| Tanzania | 2017 | 10,018 | 4,097 | N/A | N/A | N/A | N/A | N/A | N/A |
| Togo | 1998 | 4,398 | 3,753 | 4,137 | 1,191 | 1,191 | 1,190 | 1,174 | 717 |
| Togo | 2014 | 4,237 | 3,844 | 4,239 | 1,404 | 1,404 | 1,403 | 1,038 | 909 |
| Togo | 2017 | 4,674 | 1,892 | N/A | N/A | N/A | N/A | N/A | N/A |
| Uganda | 1995 | 2,771 | 3,663 | 4,429 | 1,475 | 1,475 | 1,474 | 1,172 | 1,333 |
| Uganda | 2001 | 3,365 | 3,594 | 4,361 | 1,398 | 1,399 | 1,395 | 1,174 | 1,343 |
| Uganda | 2006 | 3,883 | 4,169 | 5,062 | 1,573 | 1,573 | 1,573 | 1,947 | 1,728 |
| Uganda | 2009 | 4,134 | 1,994 | N/A | N/A | N/A | N/A | N/A | N/A |
| Uganda | 2011 | 3,981 | 3,953 | 4,761 | 1,427 | 1,427 | 1,427 | 1,676 | 1,641 |
| Uganda | 2015 | 5,322 | 2,395 | N/A | N/A | N/A | N/A | N/A | N/A |
| Uganda | 2016 | 9,320 | 7,978 | 9,300 | 2,907 | 2,907 | 2,907 | 2,913 | 2,156 |
| Zambia | 1992 | 2,289 | 3,441 | 4,069 | 1,343 | 1,341 | 1,330 | 1,206 | 695 |
| Zambia | 1996 | 3,068 | 3,860 | 4,516 | 1,348 | 1,347 | 1,347 | 1,427 | 777 |
| Zambia | 2002 | 3,419 | 3,731 | 4,289 | 1,329 | 1,329 | 1,329 | 1,227 | 861 |
| Zambia | 2007 | 3,576 | 3,442 | 3,984 | 1,266 | 1,266 | 1,266 | 898 | 523 |
| Zambia | 2014 | 8,431 | 7,122 | 7,921 | 2,580 | 2,579 | 2,575 | 2,020 | 837 |
| Zimbabwe | 1994 | 3,080 | 2,215 | 2,435 | 709 | 709 | 708 | 523 | 562 |
| Zimbabwe | 1999 | 2,945 | 2,053 | 2,235 | 724 | 724 | 724 | 432 | 509 |
| Zimbabwe | 2006 | 4,472 | 2,962 | 3,209 | 988 | 988 | 988 | 606 | 530 |
| Zimbabwe | 2011 | 4,795 | 3,315 | 3,601 | 1,059 | 1,059 | 1,059 | 674 | 531 |
| Zimbabwe | 2015 | 5,664 | 3,381 | 3,623 | 1,137 | 1,137 | 1,137 | 926 | 441 |
| **North Africa- West Asia- Europe** |  |  |  |  |  |  |  |  |  |
| Albania | 2009 | 4,328 | 808 | 879 | 276 | 276 | 276 | 81 | 141 |
| Albania | 2018 | 6,208 | 1,535 | 1,631 | 489 | N/A | 413 | 146 | 161 |
| Armenia | 2000 | 3,315 | 839 | 950 | 305 | 305 | 305 | 129 | 186 |
| Armenia | 2005 | 2,968 | 798 | 901 | 278 | 278 | 278 | 224 | 104 |
| Armenia | 2010 | 2,485 | 1,163 | 1,473 | 324 | 324 | 324 | 127 | 106 |
| Armenia | 2016 | 2,778 | 943 | 1,034 | 350 | 350 | 350 | 68 | 54 |
| Azerbaijan | 2006 | 3,496 | 1,201 | 1,402 | 439 | 439 | 439 | 225 | 98 |
| Egypt | 1992 | 6,334 | 4,232 | 5,036 | 1,691 | 1,691 | 1,691 | 1,121 | 718 |
| Egypt | 1995 | 8,962 | 6,062 | 7,139 | 2,223 | 2,223 | 2,223 | 1,860 | 2,615 |
| Egypt | 2000 | 9,905 | 6,042 | 7,063 | 2,198 | 2,198 | 2,198 | 816 | 1,059 |
| Egypt | 2003 | 5,924 | 3,520 | 4,056 | 1,230 | 1,230 | 1,230 | 1,334 | 689 |
| Egypt | 2005 | 12,789 | 7,381 | 8,321 | 2,745 | 2,745 | 2,742 | 2,460 | 1,407 |
| Egypt | 2008 | 10,884 | 6,073 | 6,876 | 2,205 | 2,205 | 2,205 | 976 | 1,067 |
| Egypt | 2014 | 14,288 | 8,707 | 9,953 | 3,281 | 3,280 | 3,278 | 2,004 | 2,461 |
| Jordan | 1990 | 4,043 | 3,700 | 5,034 | 1,699 | 1,703 | 1,702 | 671 | N/A |
| Jordan | 1997 | 3,861 | 2,998 | 3,887 | 1,289 | 1,290 | 1,290 | 1,117 | 615 |
| Jordan | 2002 | 4,018 | 3,013 | 3,691 | 1,198 | 1,198 | 1,198 | 900 | 355 |
| Jordan | 2007 | 7,269 | 5,115 | 6,252 | 1,980 | 1,980 | 1,980 | 1,659 | 653 |
| Jordan | 2009 | 6,785 | N/A | N/A | N/A | N/A | N/A | N/A | N/A |
| Jordan | 2012 | 7,821 | 5,089 | 6,117 | 2,030 | 2,030 | 2,030 | 1,539 | 1,164 |
| Jordan | 2018 | 8,882 | 5,310 | 6,330 | 1,958 | 1,958 | 1,958 | 965 | 691 |
| Moldova | 2005 | 4,360 | 945 | 1,001 | 343 | 343 | 343 | 129 | 116 |
| Morocco | 1992 | 3,330 | 2,593 | 3,055 | 1,060 | 1,059 | 1,057 | 609 | 737 |
| Morocco | 2004 | 6,602 | 3,271 | 3,600 | 1,154 | 1,154 | 1,154 | 726 | 693 |
| Turkey | 1993 | 4,847 | 1,953 | 2,222 | 762 | 762 | 758 | 840 | 431 |
| Turkey | 1998 | 4,578 | 1,916 | 2,163 | 705 | 706 | 707 | 1,039 | N/A |
| Turkey | 2003 | 3,151 | 1,131 | 2,615 | 807 | 807 | 805 | N/A | 1,298 |
| Turkey | 2008 | 5,710 | 2,080 | 2,336 | 773 | 773 | 771 | 550 | N/A |
| Turkey | 2013 | 5,802 | 1,948 | 2,151 | 743 | 743 | 741 | N/A | N/A |
| Ukraine | 2007 | 3,903 | 674 | 716 | N/A | N/A | N/A | N/A | N/A |
| Yemen | 1992 | 2,652 | 3,402 | 4,325 | 1,214 | 1,218 | 1,198 | 2,009 | 1,414 |
| Yemen | 2013 | 18,407 | 8,203 | 9,691 | 3,048 | 3,049 | 3,047 | 4,654 | 2,642 |
| **Latin America & Caribbean** |  |  |  |  |  |  |  |  |  |
| Bolivia | 1994 | 4,219 | 3,075 | 3,643 | 1,105 | 1,103 | 1,105 | 1,021 | 641 |
| Bolivia | 1998 | 5,440 | 3,655 | 4,313 | 1,331 | 1,332 | 1,330 | 1,242 | 1,645 |
| Bolivia | 2003 | 9,821 | 5,183 | 5,953 | 1,889 | 1,889 | 1,889 | 2,268 | 2,228 |
| Bolivia | 2008 | 9,445 | 4,628 | 5,225 | 1,722 | 1,722 | 1,722 | 2,049 | 1,654 |
| Brazil | 1991 | 3,190 | 1,538 | 1,872 | 624 | 624 | 624 | 416 | 492 |
| Brazil | 1996 | 7,949 | 2,618 | 3,011 | 978 | 978 | 978 | 701 | 1,136 |
| Colombia | 1990 | 4,188 | 2,042 | 2,325 | 779 | 779 | 770 | 443 | N/A |
| Colombia | 1995 | 6,240 | 2,712 | 3,105 | 1,031 | 1,031 | 1,030 | 837 | 1,195 |
| Colombia | 2000 | 6,952 | 2,525 | 2,838 | 914 | 914 | 913 | 642 | N/A |
| Colombia | 2005 | 23,980 | 7,874 | 8,684 | 2,919 | 2,919 | 2,919 | 2,162 | N/A |
| Colombia | 2010 | 33,579 | 14,492 | 17,756 | 3,435 | 3,435 | 3,435 | 2,489 | 1,143 |
| Colombia | 2015 | 25,414 | 6,470 | 6,890 | N/A | N/A | N/A | N/A | N/A |
| Dominican Republic | 1991 | 3,646 | 2,111 | 2,616 | 907 | 907 | 902 | 668 | 887 |
| Dominican Republic | 1996 | 4,706 | 2,333 | 2,781 | 971 | 968 | 957 | 258 | 1,154 |
| Dominican Republic | 1999 | 748 | 290 | 327 | 75 | 75 | 75 | 96 | 165 |
| Dominican Republic | 2002 | 14,263 | 5,880 | 6,942 | 2,272 | 2,272 | 2,271 | 1,670 | 2,249 |
| Dominican Republic | 2007 | 17,127 | 5,761 | 6,484 | 2,077 | 2,077 | 2,077 | 1,738 | 1,493 |
| Dominican Republic | 2013 | 6,051 | 2,005 | 2,212 | 737 | 667 | 560 | 631 | 575 |
| Guatemala | 1995 | 4,622 | 5,003 | 6,139 | 1,960 | 1,960 | 1,956 | 748 | 1,952 |
| Guatemala | 1999 | 2,501 | 2,418 | 2,976 | 929 | 929 | 928 | 670 | 984 |
| Guatemala | 2015 | 12,487 | 6,747 | 7,472 | 2,408 | 2,408 | 2,408 | 2,238 | 2,181 |
| Guyana | 2005 | 2,534 | 459 | N/A | N/A | N/A | N/A | N/A | 55 |
| Guyana | 2009 | 2,701 | 1,217 | 1,407 | 426 | 426 | 426 | 208 | 210 |
| Haiti | 1995 | 1,937 | 1,794 | 2,107 | 654 | 654 | 651 | 875 | 645 |
| Haiti | 2000 | 4,611 | 3,436 | 4,074 | 1,268 | 1,268 | 1,267 | 1,534 | 2,398 |
| Haiti | 2006 | 5,120 | 3,254 | 3,700 | 1,186 | 1,186 | 1,186 | 1,212 | 1,507 |
| Haiti | 2012 | 6,844 | 4,043 | 4,540 | 1,370 | 1,370 | 1,370 | 1,411 | 2,435 |
| Haiti | 2017 | 7,133 | 3,527 | 3,904 | 1,206 | 1,206 | 1,206 | 1,230 | 1,911 |
| Honduras | 2006 | 10,715 | 5,757 | 6,432 | 2,103 | 2,103 | 2,103 | 1,794 | 1,891 |
| Honduras | 2012 | 12,836 | 6,191 | 6,724 | 2,277 | 2,277 | 2,277 | 1,909 | 2,097 |
| Nicaragua | 1998 | 7,117 | 4,174 | 4,863 | 1,563 | 1,560 | 1,543 | 1,074 | 2,044 |
| Nicaragua | 2001 | 7,341 | 3,677 | 4,176 | 1,452 | 1,452 | 1,451 | 841 | 1,992 |
| Paraguay | 1990 | 2,539 | 2,067 | 2,596 | 863 | 865 | 858 | 338 | N/A |
| Peru | 1992 | 7,968 | 4,662 | 5,433 | 1,752 | 1,750 | 1,740 | 1,691 | 2,142 |
| Peru | 1996 | 15,864 | 8,917 | 10,247 | 3,301 | 3,298 | 3,296 | 3,256 | 3,580 |
| Peru | 2000 | 15,497 | 7,106 | 7,859 | 2,593 | 2,593 | 2,592 | 2,192 | 2,534 |
| Peru | 2006 | 23,949 | 9,413 | 10,196 | 3,433 | 3,433 | 3,433 | 2,679 | 3,244 |
| Peru | 2008 | 23,949 | 9,413 | 10,196 | 3,433 | 3,433 | 3,433 | 2,679 | 3,244 |
| Peru | 2009 | 14,237 | 5,608 | 6,010 | 2,004 | 2,004 | 2,004 | 1,474 | 1,631 |
| Peru | 2010 | 13,439 | 7,665 | 9,281 | 1,860 | 1,860 | 1,860 | 1,398 | 1,530 |
| Peru | 2011 | 13,400 | 5,068 | 5,432 | 1,773 | 1,773 | 1,773 | 1,311 | 1,457 |
| Peru | 2012 | 14,252 | 5,333 | 5,708 | 1,861 | 1,861 | 1,861 | 1,253 | 1,343 |

**Note:** mDFPS= Demand for family planning satisfied with modern contraceptive methods, ANC= Antenatal care visits, SBA= Skilled birth attendance, BCG= BCG immunization, DPT= Three doses of DPT immunization, Measles= Measles immunization, ORT= Oral rehydration therapy for diarrhoea treatment, ARI Care= Care seeking for symptoms of acute respiratory infections. N/A represents no observation.

Table S2: Potential scale reduction factor (PSRF) to diagnose convergence after Markov Chain Monte Carlo simulation in Bayesian regression model for Composite Coverage Index (CCI) in low- and midle-income countries

| **Country** | **Overall** | **Poorest** | **Poorer** | **Middle** | **Richer** | **Richest** | **Urban** | **Rural** | **Below secondary** | **Secondary+** | **15-19 years** | **20-49 years** |
| --- | --- | --- | --- | --- | --- | --- | --- | --- | --- | --- | --- | --- |
| Armenia | 1.082279 | 1.000371 | 1.001318 | 1.01008 | 1.005122 | 1.012468 | 1.007551 | 1.059433 | N/A | 1.004314 | 1.001633 | 1.002564 |
| Bangladesh | 1.005743 | 1.008629 | 0.999365 | 1.00123 | 1.000217 | 1.004218 | 1.009514 | 1.026067 | 1.005178 | 0.9993096 | 1.024709 | 1.000364 |
| Benin | 1.00623 | 1.004668 | 1.001744 | 1.002817 | 1.005954 | 1.008851 | 1.012749 | 0.9994566 | 1.010389 | 0.9993799 | 1.010106 | 1.033769 |
| Bolivia | 1.05375 | 1.005813 | 1.003314 | 1.025968 | 1.043772 | 1.002015 | 1.033736 | 1.008758 | 1.000261 | 1.00005 | 1.002415 | 1.002723 |
| Brazil | 1.027799 | N/A | N/A | N/A | N/A | N/A | 1.004281 | 0.9994116 | 1.042138 | 1.022729 | 1.006507 | 1.005209 |
| Burkina Faso | 1.004281 | 0.9998707 | 1.00032 | 1.000039 | 1.001124 | 1.002228 | 1.003664 | 1.005708 | 1.001122 | 1.02139 | 1.033844 | 1.01166 |
| Burundi | 0.999665 | 1.009778 | 1.001569 | 1.023307 | 1.003808 | 1.012377 | 1.030984 | 1.008147 | 0.9991958 | 1.000028 | 1.001639 | 1.002219 |
| Cambodia | 1.023614 | 1.017835 | 1.037917 | 1.014865 | 1.033757 | 1.017446 | 1.002513 | 1.003971 | 1.011782 | 1.005817 | 1.00148 | 1.001054 |
| Cameroon | 1.005133 | 1.001052 | 1.000957 | 1.006821 | 1.000582 | 1.019947 | 1.006361 | 1.00404 | 1.003428 | 1.020961 | 1.003087 | 1.016648 |
| Chad | 0.999187 | 1.000593 | 1.015199 | 0.9995487 | 1.012876 | 0.9999555 | 1.017115 | 1.00161 | 1.000038 | 1.001796 | 1.023491 | 1.031508 |
| Colombia | 1.010153 | 1.002301 | 1.007251 | 1.044505 | 1.001804 | 1.005283 | 1.003022 | 1.004814 | 1.034979 | 1.004012 | 1.001424 | 0.9997961 |
| Comoros | 1.002826 | 1.000471 | 1.0002 | 1.031299 | 1.008222 | 1.003572 | 1.000186 | 1.003316 | 1.025391 | 1.004651 | 0.9999728 | 1.002281 |
| Congo | 1.000058 | 1.004337 | 1.001451 | 1.000629 | 1.000477 | 1.025004 | 1.005163 | 1.007237 | 1.000459 | 1.000037 | 1.001261 | 1.011357 |
| Congo DR | 0.999333 | 0.9992503 | 1.000864 | 1.032264 | 1.008237 | 0.9991869 | 1.002255 | 1.001087 | 1.007856 | 1.002239 | 1.000063 | 1.000697 |
| Cote d'Ivoire | 1.000565 | 1.007624 | 1.010423 | 1.003221 | 1.004592 | 0.999478 | 1.002796 | 0.9996693 | 1.00753 | 1.003046 | 1.008596 | 1.018322 |
| Dominican Republic | 1.000843 | 1.06709 | 1.001672 | 1.012179 | 1.028461 | 1.000357 | 1.027789 | 0.9999428 | 1.009277 | 1.004254 | 1.004685 | 1.005555 |
| Egypt | 0.9995822 | 1.003294 | 1.005983 | 1.012324 | 1.008765 | 1.017416 | 1.007325 | 1.009607 | 1.014811 | 1.018479 | 1.00651 | 1.005604 |
| Ethiopia | 1.011459 | 1.004256 | 1.028545 | 0.9999586 | 1.012641 | 1.009015 | 1.01791 | 1.006285 | 1.007919 | 1.000367 | 1.00841 | 1.003341 |
| Gabon | 1.029593 | 1.000224 | 1.001864 | 0.9996263 | 1.000813 | 1.022218 | 1.038962 | 1.004693 | 1.016456 | 1.003335 | 1.000671 | 1.002491 |
| Ghana | 1.033429 | 0.9991806 | 1.005124 | 1.011405 | 0.9998493 | 1.00375 | 1.006367 | 1.049444 | 1.00384 | 1.003427 | 1.036521 | 1.001611 |
| Guatemala | 1.000559 | 1.005362 | 1.002111 | 1.002221 | 1.002645 | 1.000101 | 1.05431 | 1.02494 | 1.029418 | 1.052965 | 1.002085 | 1.01265 |
| Guinea | 1.004251 | 1.004551 | 1.014053 | 1.016878 | 1.026048 | 1.014844 | 1.000943 | 1.024508 | 1.005205 | 1.000984 | 0.999763 | 1.004489 |
| Haiti | 1.010815 | 1.003395 | 1.000627 | 1.009627 | 1.007894 | 1.014747 | 1.005815 | 1.000109 | 1.009777 | 1.003948 | 1.005229 | 1.000802 |
| Honduras | 0.9993909 | 1.001084 | 1.012272 | 1.000295 | 1.001023 | 1.016485 | 1.020357 | 1.007565 | 1.003199 | 1.004446 | 0.9992248 | 1.034613 |
| India | 1.032664 | 1.007969 | 1.034974 | 1.011427 | 1.011471 | 1.004225 | 1.029453 | 1.009256 | 1.000931 | 1.010671 | 1.025639 | 1.008886 |
| Indonesia | 1.000859 | 1.006747 | 1.006297 | 1.011399 | 1.003932 | 0.9992329 | 1.004986 | 1.005898 | 1.007096 | 1.017168 | 1.008712 | 1.010803 |
| Jordan | 1.002463 | 1.021994 | 1.005732 | 0.9998265 | 1.002736 | 0.9991914 | 1.011272 | 1.012947 | 1.003009 | 1.01479 | 0.999444 | 1.008047 |
| Kazakhstan | 0.9993706 | 0.999505 | 1.008689 | 1.001214 | 1.006453 | 1.005222 | 1.00437 | 1.003603 | N/A | 0.9994702 | N/A | 1.020453 |
| Kenya | 1.0133 | 1.011995 | 1.000323 | 1.001704 | 1.031012 | 1.005065 | 1.004103 | 1.000701 | 1.00468 | 1.022708 | 0.9992592 | 1.00085 |
| Kyrgyz Republic | 1.003994 | 0.9996243 | 0.9996979 | 0.9996448 | 1.018178 | 1.006061 | 0.9992155 | 1.002176 | N/A | 1.008509 | 1.011638 | 1.006452 |
| Lesotho | 1.015513 | 1.007564 | 1.031725 | 1.006948 | 1.009082 | 1.025991 | 1.01006 | 1.001284 | 0.9993511 | 1.016686 | 1.004388 | 1.018935 |
| Liberia | 1.001219 | 1.002242 | 1.001788 | 1.000578 | 1.004883 | 1.002484 | 1.017607 | 1.006777 | 1.016881 | 1.001837 | 1.008177 | 1.018215 |
| Madagascar | 1.002054 | 1.002176 | 1.002453 | 1.012185 | 0.9998559 | 1.001957 | 1.00008 | 1.002423 | 1.004044 | 1.014317 | 1.010908 | 1.006773 |
| Malawi | 1.003825 | 1.005378 | 1.01205 | 1.005276 | 1.006258 | 1.012922 | 1.000509 | 1.024526 | 1.034633 | 1.000097 | 1.027336 | 1.013937 |
| Maldives | 1.001877 | 1.003945 | 1.001483 | 1.00913 | 1.009554 | 1.000777 | 1.029352 | 1.016135 | 1.000899 | 1.000346 | 1.027336 | 1.004582 |
| Mali | 1.001132 | 0.9992566 | 1.021482 | 1.023937 | 1.000178 | 1.008176 | 1.000894 | 1.008859 | 1.001661 | 1.002576 | 1.001675 | 0.9993742 |
| Morocco | 1.00112 | 1.001562 | 1.00051 | 1.032699 | 1.011755 | 1.000127 | 0.9995596 | 1.000404 | 0.9995653 | 1.000238 | 1.026876 | 1.003017 |
| Mozambique | 1.023182 | 1.00626 | 1.000796 | 0.9992504 | 1.009046 | 1.011427 | 1.001884 | 1.012251 | 1.005272 | 1.012357 | 0.9996677 | 1.005704 |
| Namibia | 1.00844 | 1.003167 | 1.026838 | 1.001752 | 1.006664 | 1.011069 | 0.9997951 | 1.008036 | 0.999709 | 0.9994417 | 1.018061 | 0.9994164 |
| Nepal | 1.027492 | 1.003996 | 1.00095 | 1.018897 | 1.004468 | 1.004539 | 1.005056 | 1.004161 | 1.002663 | 1.003642 | 1.009953 | 1.003142 |
| Nicaragua | 1.010066 | 1.008016 | 0.9991733 | 1.016861 | 0.9993788 | 0.99959 | 1.007728 | 0.9994661 | 1.02448 | 1.002478 | 0.9995465 | 0.9997333 |
| Niger | 1.009692 | 1.001353 | 1.033369 | 1.013851 | 1.002302 | 1.00627 | 0.9992871 | 1.003309 | 0.9992802 | 1.019534 | 1.000844 | 1.044164 |
| Nigeria | 1.0098 | 1.009278 | 1.003218 | 1.000769 | 1.002951 | 1.000354 | 1.006157 | 1.002694 | 1.013066 | 1.006964 | 0.9992286 | 1.000504 |
| Pakistan | 1.018041 | 1.007284 | 1.037675 | 1.006818 | 0.9992163 | 1.009814 | 1.009472 | 1.020485 | 1.009041 | 1.00345 | 1.025628 | 1.008312 |
| Peru | 1.012247 | 1.005205 | 1.000036 | 0.9998574 | 1.001085 | 0.9998924 | 1.008728 | 1.003463 | 1.022789 | 1.021617 | 1.004655 | 1.003879 |
| Philippines | 1.016314 | 1.000581 | 1.00201 | 1.001549 | 1.003155 | 1.005562 | 0.9994163 | 1.002608 | 1.001567 | 1.00595 | 1.010169 | 1.043234 |
| Rwanda | 1.010568 | 1.002183 | 1.008468 | 0.9993384 | 1.037646 | 1.000016 | 1.027369 | 1.002032 | 1.010781 | 1.004442 | 1.000775 | 1.001209 |
| Senegal | 1.004239 | 1.001638 | 1.015016 | 1.009216 | 1.003454 | 0.9991816 | 1.002487 | 0.9991744 | 1.004604 | 1.004187 | 1.011147 | 1.060842 |
| Sierra Leone | 1.00336 | 1.019634 | 1.004465 | 1.009986 | 1.001066 | 1.004214 | 1.002149 | 1.000737 | 1.015621 | 1.021329 | 1.003668 | 0.9993112 |
| South Africa | 1.007935 | 1.001835 | 1.015576 | 1.003507 | 1.003034 | 0.9999362 | 1.000521 | 1.000456 | 1.000561 | 1.0154 | 1.004936 | 1.001488 |
| Tajikistan | 0.9994388 | 1.005694 | 0.9998233 | 0.9993843 | 0.999413 | 1.028666 | 1.012031 | 0.9999216 | 1.006793 | 1.000959 | 1.004936 | 1.001941 |
| Tanzania | 1.019469 | 1.000325 | 0.9996957 | 1.005028 | 1.009265 | 1.00461 | 1.014794 | 1.004795 | 0.9995478 | 1.002805 | 1.000777 | 1.003607 |
| Timor-Leste | 1.017958 | 1.000948 | 0.9998833 | 1.003651 | 1.000829 | 1.004273 | 1.007924 | 1.012887 | 1.004835 | 1.014256 | 1.013976 | 1.000324 |
| Togo | 1.01022 | 1.017062 | 1.000409 | 1.004535 | 1.007595 | 1.000964 | 1.003373 | 1.000886 | 0.999902 | 1.000439 | 1.013502 | 1.002441 |
| Turkey | 1.002463 | 1.021994 | 1.005732 | 0.9998265 | 1.002736 | 0.9991914 | 1.011272 | 1.012947 | 1.003009 | 1.01479 | 1.013502 | 1.002441 |
| Uganda | 1.020618 | 1.003347 | 1.019398 | 1.01405 | 1.0094 | 1.003825 | 0.9993297 | 1.024783 | 1.012204 | 1.002685 | 1.002472 | 1.003705 |
| Vietnam | 0.9997655 | 0.9995807 | 1.005707 | 1.014685 | 1.0006 | 1.035482 | 1.002986 | 0.9992875 | 1.011626 | 1.018744 | 1.002472 | 0.9996966 |
| Yemen | 1.002138 | N/A | N/A | N/A | N/A | N/A | 0.9998903 | 1.00444 | 1.005689 | 1.007187 | 1.005445 | 1.040312 |
| Zambia | 1.001938 | 1.004322 | 1.022836 | 1.002092 | 1.004213 | 1.026209 | 1.000657 | 0.9997833 | 1.000556 | 1.030124 | 0.9997042 | 1.010482 |
| Zimbabwe | 1.003535 | 1.006337 | 0.9991679 | 0.9994668 | 1.002555 | 1.001137 | 1.001103 | 1.002197 | 1.011039 | 1.002455 | 1.015495 | 1.004399 |

**Note:** N/A denotes not application and refers that the indicator has no sufficient data to make projections

Table S3: Potential scale reduction factor (PSRF) to diagnose convergence after Markov Chain Monte Carlo simulation in Bayesian regression model for demand for family planning satisfied with modern contraceptive method (mDFPS) among women 15-49 years of age in low- and midle-income countries^1^

| **Country** | **Overall** | **Poorest** | **Poorer** | **Middle** | **Richer** | **Richest** | **Urban** | **Rural** | **Below secondary** | **Secondary+** | **15-19 years** | **20-49 years** |
| --- | --- | --- | --- | --- | --- | --- | --- | --- | --- | --- | --- | --- |
| Albania | 1.002602 | 1.046127 | 1.025066 | 1.008538 | 1.022346 | 1.040587 | 1.062268 | 1.01763 | 1.015431 | 1.000115 | 1.057264 | 1.007247 |
| Armenia | 1.000546 | 1.008543 | 1.006769 | 1.000064 | 1.019737 | 1.012423 | 1.011185 | 1.007863 | 0.9996975 | 1.007339 | 1.029605 | 1.00175 |
| Bangladesh | 1.003594 | 1.002911 | 1.00027 | 1.003439 | 1.004879 | 1.000818 | 1.012539 | 1.003012 | 0.9993852 | 1.01974 | 1.003421 | 0.9992995 |
| Benin | 1.003083 | 1.000701 | 0.9999637 | 0.9995313 | 1.008242 | 1.000382 | 1.029752 | 1.002379 | 1.011806 | 1.000943 | 1.016729 | 1.002492 |
| Bolivia | 0.999948 | 1.003458 | 1.002984 | 1.005545 | 1.000213 | 1.001355 | 1.00061 | 1.003429 | 1.01426 | 0.999616 | 1.010286 | 1.002082 |
| Brazil | 1.001893 | N/A | N/A | N/A | N/A | N/A | 1.00827 | 1.002755 | 0.999967 | 1.001444 | 0.9996628 | 0.9997334 |
| Burkina Faso | 1.034582 | 1.001025 | 1.004088 | 1.001146 | 1.019904 | 1.003245 | 1.004598 | 1.053439 | 1.001997 | 1.046434 | 1.053457 | 1.001839 |
| Burundi | 1.016391 | 1.00168 | 1.016713 | 1.004878 | 1.004366 | 1.011789 | 1.001405 | 0.9997025 | 1.016825 | 1.003224 | 0.9991745 | 1.012159 |
| Cambodia | 1.003566 | 1.030699 | 1.018465 | 0.9997129 | 1.005535 | 1.0478 | 1.000158 | 1.003335 | 1.000095 | 1.001772 | 1.020187 | 1.01287 |
| Cameroon | 1.010093 | 0.9993525 | 0.999543 | 0.9993306 | 1.004063 | 1.003813 | 1.007063 | 1.000882 | 1.008725 | 1.007458 | 1.00832 | 0.9994039 |
| Chad | 1.014071 | 1.018054 | 1.015566 | 1.020665 | 1.000868 | 1.007175 | 1.003938 | 1.037462 | 1.02589 | 1.002483 | 0.9996742 | 1.000794 |
| Colombia | 1.002504 | 1.00465 | 1.01127 | 1.000729 | 1.00035 | 1.001364 | 1.000543 | 1.002959 | 1.005385 | 1.000256 | 1.00596 | 1.009979 |
| Comoros | 1.008116 | 1.00002 | 1.004965 | 1.005188 | 1.00216 | 1.007843 | 1.000207 | 1.000601 | 1.001685 | 1.015305 | 1.004277 | 1.000654 |
| Congo | 1.002442 | 1.010723 | 1.00883 | 1.001058 | 0.9998926 | 1.002113 | 0.9992762 | 1.001628 | 1.027527 | 0.9994203 | 1.004966 | 1.002636 |
| Congo DR | 1.010937 | 1.031114 | 1.012681 | 0.999346 | 1.00249 | 1.010748 | 0.9992643 | 0.9994473 | 1.001913 | 1.034449 | 1.008064 | 1.004994 |
| Cote d'Ivoire | 1.00159 | 1.007667 | 1.03618 | 1.001768 | 1.006002 | 0.9995049 | 1.018053 | 1.003525 | 1.002824 | 1.0044 | 1.005415 | 1.001106 |
| Dominican Republic | 1.023561 | 1.006235 | 1.030056 | 1.002637 | 1.001275 | 1.014054 | 0.9999256 | 1.004425 | 1.015505 | 1.003885 | 1.024987 | 1.034822 |
| Egypt | 1.002921 | 0.9994051 | 0.999541 | 1.028676 | 1.007163 | 1.005685 | 1.011568 | 1.000537 | 1.007507 | 1.011773 | 1.003788 | 1.012265 |
| Ethiopia | 0.9995553 | 0.9991667 | 0.9992328 | 1.000037 | 1.000286 | 1.007264 | 1.008126 | 1.00323 | 1.04696 | 1.014102 | 0.9994534 | 1.036922 |
| Gabon | 1.009197 | 1.001678 | 1.000513 | 1.000003 | 1.004906 | 1.001842 | 0.9993378 | 1.002484 | 1.002429 | 1.007744 | 1.021399 | 1.010055 |
| Ghana | 1.000016 | 1.001574 | 1.002708 | 1.007982 | 0.9996344 | 1.002383 | 1.002161 | 1.000226 | 1.016286 | 1.011849 | 1.008298 | 1.005809 |
| Guatemala | 1.013668 | 0.9991974 | 1.02951 | 1.008095 | 0.9996174 | 0.9996994 | 1.031847 | 1.00472 | 1.001628 | 1.011306 | 1.00601 | 0.9994594 |
| Guinea | 1.001729 | 1.002832 | 1.019918 | 1.006761 | 1.007606 | 1.006957 | 1.003627 | 1.025281 | 1.005078 | 1.019143 | 1.008967 | 1.001293 |
| Guyana | 1.063783 | 1.062621 | 1.023655 | 1.035715 | 1.005952 | 1.096588 | 1.087029 | 1.088948 | 1.010596 | 1.008545 | 1.00617 | 1.074098 |
| Haiti | 1.001618 | 1.005936 | 1.004039 | 1.010084 | 1.007527 | 1.005312 | 1.002289 | 1.00641 | 0.9993667 | 1.003708 | 1.006626 | 1.012128 |
| Honduras | 1.051989 | 0.9992044 | 1.00494 | 1.003099 | 1.001659 | 1.019989 | 0.9993396 | 1.047973 | 1.002634 | 1.001706 | 1.017864 | 1.063941 |
| India | 1.009603 | 0.999261 | 1.000843 | 1.006093 | 1.005006 | 1.006481 | 1.003356 | 1.000267 | 1.012265 | 1.016692 | 1.003643 | 1.034623 |
| Indonesia | 1.006841 | 1.007438 | 1.000274 | 1.014077 | 1.017438 | 0.9998896 | 0.9997988 | 1.005871 | 0.9999933 | 1.002285 | 1.016951 | 1.013764 |
| Jordan | 1.028272 | 1.003869 | 1.000059 | 0.9993604 | 1.009753 | 1.000428 | 1.010095 | 1.001206 | 1.022844 | 1.003536 | 1.02451 | 1.000273 |
| Kazakhstan | 1.000201 | 1.004888 | 0.999473 | 1.00507 | 1.057991 | 1.008369 | 1.003407 | 1.005476 | N/A | 1.000092 | 1.001956 | 1.004942 |
| Kenya | 1.003172 | 1.00225 | 1.039845 | 1.007778 | 1.009435 | 1.00314 | 1.008356 | 1.005015 | 1.005583 | 1.009763 | 1.000608 | 0.9995195 |
| Kyrgyz Republic | 1.009661 | 1.002427 | 1.000597 | 1.006272 | 1.055965 | 1.001984 | 1.002514 | 0.9999691 | 1.016365 | 1.002656 | 1.002234 | 1.008426 |
| Lesotho | 1.015623 | 1.068955 | 1.000358 | 1.006366 | 1.06503 | 1.005066 | 1.009481 | 1.023652 | 1.002544 | 1.016474 | 1.0119 | 1.016464 |
| Liberia | 1.07637 | 1.083017 | 1.06182 | 1.069824 | 1.04877 | 1.071708 | 1.063009 | 0.9998111 | 0.9998503 | 1.033501 | 1.004691 | 1.068471 |
| Madagascar | 1.039288 | 1.029917 | 0.9996094 | 1.005484 | 0.9995529 | 1.052162 | 1.005485 | 1.014857 | 1.024782 | 0.9996648 | 1.018997 | 1.028558 |
| Malawi | 1.01708 | 1.012531 | 1.000699 | 1.004449 | 1.000205 | 1.014616 | 1.004171 | 1.016641 | 1.005007 | 1.029391 | 0.9994907 | 1.00531 |
| Maldives | 1.003379 | 1.037065 | 1.00024 | 1.005521 | 1.001598 | 1.06817 | 1.003615 | 1.038655 | 1.002681 | 1.002473 | 1.04778 | 1.029644 |
| Mali | 0.9995116 | 1.012617 | 1.000028 | 1.017294 | 1.001058 | 1.000398 | 1.006156 | 1.033134 | 1.006003 | 1.001704 | 1.000466 | 1.004675 |
| Morocco | 0.999527 | 1.08161 | 1.008577 | 1.005419 | 1.003932 | 1.014669 | 1.013119 | 1.029448 | 1.074771 | 1.008222 | 1.020056 | 0.9999138 |
| Mozambique | 1.001051 | 1.002078 | 1.006171 | 1.01922 | 1.0017 | 1.001301 | 1.001068 | 0.9994485 | 1.007666 | 1.02048 | 0.9996632 | 1.002902 |
| Namibia | 0.999813 | 1.008184 | 1.003139 | 1.009721 | 1.019887 | 0.9993833 | 1.01691 | 1.005032 | 1.000669 | 1.012008 | 1.00109 | 1.0482 |
| Nepal | 1.002052 | 0.9997994 | 1.007558 | 1.002431 | 0.9996468 | 1.001041 | 0.9995079 | 1.011805 | 1.000191 | 1.005973 | 1.013358 | 0.9996986 |
| Nicaragua | 0.9996779 | 1.007215 | 1.004803 | 1.010609 | 1.000051 | 1.004162 | 0.999285 | 0.999378 | 1.030121 | 1.035742 | 0.9993834 | 1.01021 |
| Niger | 1.000031 | 1.001284 | 1.016542 | 1.004869 | 0.999225 | 1.002698 | 1.001506 | 1.009497 | 1.002963 | 1.009758 | 1.007558 | 1.000347 |
| Nigeria | 1.003934 | 1.001557 | 1.002695 | 1.002997 | 1.007602 | 1.000259 | 1.004061 | 1.007131 | 1.001047 | 1.012075 | 0.9994051 | 1.025094 |
| Pakistan | 1.003664 | 1.000355 | 1.00242 | 0.9995096 | 1.00054 | 1.010261 | 1.025802 | 1.000597 | 1.016184 | 1.001538 | 1.004682 | 1.014453 |
| Peru | 1.005908 | 1.023951 | 1.01014 | 1.001943 | 1.002538 | 1.002078 | 0.9993212 | 1.012838 | 1.011888 | 1.005976 | 1.004021 | 1.000835 |
| Philippines | 1.000296 | 1.025562 | 1.000633 | 1.003094 | 1.016626 | 1.011901 | 1.003556 | 1.007378 | 1.002266 | 1.016945 | 1.000633 | 1.020299 |
| Rwanda | 0.9997003 | 1.009025 | 1.034497 | 1.006835 | 1.000156 | 1.000862 | 1.014656 | 1.005464 | 1.011463 | 1.008361 | 1.008392 | 1.002489 |
| Senegal | 1.006694 | 1.014695 | 0.9996505 | 1.002883 | 1.002012 | 1.015557 | 1.022872 | 1.016316 | 1.01399 | 1.001486 | 1.006738 | 1.014961 |
| Sierra Leone | 1.038532 | 1.016119 | 1.029223 | 1.020179 | 1.022127 | 1.021419 | 1.000384 | 1.016487 | 1.016723 | 1.009203 | 1.029514 | 1.010981 |
| South Africa | 1.001492 | 1.010486 | 1.019052 | 1.009676 | 1.010001 | 1.002064 | 1.001419 | 1.003921 | 1.016783 | 1.000431 | 1.010049 | 0.9994439 |
| Tajikistan | 1.001954 | 1.005342 | 1.002308 | 1.038638 | 0.9991702 | 1.002196 | 0.9997966 | 1.001058 | 1.001481 | 0.9994629 | 1.000628 | 1.002849 |
| Tanzania | 1.008581 | 1.001258 | 1.001611 | 1.00639 | 1.004239 | 1.001845 | 1.000128 | 1.037542 | 1.010144 | 1.000231 | 1.003541 | 1.004211 |
| Timor-Leste | 0.9995726 | 1.007391 | 0.9997873 | 1.057657 | 1.00848 | 1.001235 | 1.001396 | 1.002543 | 1.00017 | 1.003995 | 1.002245 | 1.005617 |
| Togo | 1.003829 | 1.00105 | 1.006813 | 1.001363 | 1.000105 | 1.009398 | 1.022387 | 1.006275 | 0.9998936 | 1.004473 | 0.9991941 | 0.9993048 |
| Turkey | 1.023759 | 1.000733 | 1.008358 | 1.010915 | 1.0443 | 0.9999198 | 1.00805 | 1.000434 | 1.000051 | 1.005498 | 1.008495 | 1.006424 |
| Uganda | 1.021106 | 0.999231 | 1.009563 | 1.002148 | 1.053761 | 1.006539 | 1.004629 | 1.002643 | 1.000786 | 1.004459 | 1.003365 | 1.009552 |
| Vietnam | 1.005248 | 0.99961 | 1.011393 | 1.005664 | 1.016114 | 0.999912 | 1.017461 | 1.021757 | 1.007648 | 1.025457 | 1.018374 | 1.015666 |
| Yemen | 1.002293 | N/A | N/A | N/A | N/A | N/A | 1.00329 | 1.000325 | 1.007929 | 1.017811 | 1.011528 | 0.9997647 |
| Zambia | 1.013266 | 1.050527 | 1.000871 | 1.000216 | 1.005463 | 1.023364 | 1.003882 | 1.005786 | 1.02647 | 1.000352 | 1.018633 | 0.9998743 |
| Zimbabwe | 1.001761 | 0.9994726 | 1.000782 | 1.000164 | 1.000472 | 1.000901 | 1.001832 | 1.014954 | 1.031788 | 1.007563 | 1.014622 | 1.005614 |

^1^The PSRF values were drawn from models when fitted for calculating the probability of attaining ≥99% coverage for mDFPS. N/A denotes not application and refers that the indicator has no sufficient data to make projections

Table S4: Potential scale reduction factor (PSRF) to diagnose convergence after Markov Chain Monte Carlo simulation in Bayesian regression model for demand for family planning satisfied with modern contraceptive method (mDFPS) among women 15-49 years of age in low- and midle-income countries^1^

| **Country** | **Overall** | **Poorest** | **Poorer** | **Middle** | **Richer** | **Richest** | **Urban** | **Rural** | **Below secondary** | **Secondary+** | **15-19 years** | **20-49 years** |
| --- | --- | --- | --- | --- | --- | --- | --- | --- | --- | --- | --- | --- |
| Albania | 1.012114 | 1.046802 | 1.000041 | 1.005234 | 1.007746 | 1.024992 | 1.01024 | 1.043805 | 1.007635 | 1.085974 | 1.011506 | 1.02616 |
| Armenia | 1.000796 | 1.01378 | 1.000068 | 1.00159 | 1.00224 | 1.000177 | 0.9993688 | 1.010859 | 0.9998416 | 1.004796 | 1.020371 | 1.001556 |
| Bangladesh | 1.002042 | 1.015984 | 1.037559 | 1.000213 | 1.005924 | 1.039262 | 1.005732 | 1.001737 | 0.9997811 | 1.002626 | 1.011803 | 0.9998313 |
| Benin | 1.02234 | 1.011944 | 1.005796 | 0.9998531 | 1.005411 | 1.002829 | 1.00537 | 0.9998059 | 0.9995064 | 1.00022 | 0.9994816 | 1.045125 |
| Bolivia | 1.003043 | 1.00927 | 1.034419 | 1.009399 | 1.001021 | 1.004226 | 1.001926 | 1.005833 | 1.016536 | 0.9997312 | 1.041699 | 1.001279 |
| Brazil | 1.058068 | N/A | N/A | N/A | N/A | N/A | 1.005207 | 1.013327 | 0.9999804 | 1.074511 | 1.001324 | 1.011901 |
| Burkina Faso | 1.003107 | 1.001883 | 1.007839 | 0.9996642 | 1.026989 | 1.003801 | 1.012682 | 0.999806 | 1.015405 | 1.008298 | 1.00829 | 1.001323 |
| Burundi | 1.000872 | 1.003038 | 1.010135 | 1.009049 | 1.001071 | 0.9996521 | 1.002904 | 1.007129 | 1.00211 | 1.002482 | 1.002149 | 1.001362 |
| Cambodia | 1.018798 | 1.014848 | 1.002446 | 1.049062 | 1.010321 | 1.003598 | 1.010728 | 1.008177 | 0.9995461 | 1.002351 | 1.024609 | 0.9998276 |
| Cameroon | 1.009426 | 0.9993176 | 1.002665 | 1.002127 | 1.00293 | 1.034065 | 1.000374 | 1.006137 | 1.018009 | 1.027602 | 0.9995296 | 1.015086 |
| Chad | 1.005052 | 1.008221 | 1.001525 | 1.002567 | 1.001638 | 1.006855 | 0.9997852 | 1.000495 | 1.012217 | 1.004764 | 1.001366 | 1.000111 |
| Colombia | 1.001754 | 1.004695 | 1.02504 | 1.008577 | 1.00917 | 1.008909 | 1.004599 | 1.007737 | 1.000199 | 1.019728 | 1.002961 | 1.001353 |
| Comoros | 0.9996421 | 1.003767 | 1.000846 | 1.002751 | 0.9999735 | 1.001219 | 1.003787 | 1.008927 | 1.003114 | 1.000857 | 0.9998894 | 1.00973 |
| Congo | 1.00299 | 1.005376 | 1.000513 | 1.000424 | 1.00963 | 1.081801 | 1.000192 | 0.9993035 | 1.000309 | 1.016587 | 1.010997 | 1.00403 |
| Congo DR | 1.001146 | 1.005816 | 0.9997776 | 1.030916 | 0.9998105 | 0.9992314 | 1.000304 | 1.007506 | 1.00376 | 0.9991816 | 1.003354 | 1.001754 |
| Cote d'Ivoire | 1.004823 | 1.001535 | 0.9996978 | 1.004207 | 1.014603 | 0.9995021 | 1.004007 | 1.000888 | 0.9998419 | 1.001552 | 1.000049 | 1.002693 |
| Dominican Republic | 1.0299 | 1.046758 | 0.9998548 | 1.003733 | 1.002129 | 1.001419 | 0.9999778 | 1.004287 | 1.012806 | 1.028196 | 1.006537 | 1.007242 |
| Egypt | 1.003909 | 1.003168 | 1.035358 | 1.007202 | 1.028158 | 1.002857 | 1.000871 | 1.000222 | 1.078444 | 1.003177 | 1.003133 | 0.999608 |
| Ethiopia | 1.003778 | 1.004829 | 1.004748 | 1.000576 | 1.001207 | 1.00061 | 1.014046 | 1.024531 | 1.015966 | 1.003483 | 1.00173 | 1.001477 |
| Gabon | 1.005732 | 1.003314 | 1.003799 | 1.001099 | 1.011928 | 1.000924 | 1.000171 | 1.000366 | 1.000819 | 1.018509 | 1.000372 | 0.9996241 |
| Ghana | 1.010909 | 1.0027 | 1.004784 | 1.004986 | 1.002073 | 1.000749 | 0.9998719 | 1.001144 | 1.00103 | 1.002475 | 1.003531 | 1.000549 |
| Guatemala | 1.008002 | 1.002974 | 0.9993206 | 1.008077 | 0.9998564 | 1.000957 | 1.003866 | 1.026803 | 1.000094 | 1.001087 | 1.000942 | 1.004682 |
| Guinea | 0.9998403 | 1.006698 | 1.009849 | 1.014303 | 0.9999116 | 1.004909 | 1.001936 | 1.0004 | 1.024707 | 1.032312 | 1.001686 | 1.011138 |
| Guyana | 1.096824 | 1.010915 | 1.00257 | 1.000469 | 1.003342 | 1.094789 | 1.001742 | 1.006189 | 1.033255 | 1.094637 | 1.098024 | 1.000192 |
| Haiti | 1.004216 | 1.04265 | 1.021504 | 1.001547 | 1.018819 | 1.01487 | 1.011004 | 1.004647 | 1.010005 | 0.9997344 | 1.001783 | 0.9992174 |
| Honduras | 1.007041 | 1.000885 | 1.00889 | 1.001715 | 1.001789 | 1.008643 | 1.002065 | 1.004309 | 1.001753 | 1.000986 | 1.001608 | 1.005949 |
| India | 1.019592 | 1.021219 | 1.021693 | 1.004073 | 1.001544 | 1.004169 | 1.004107 | 1.002646 | 1.002895 | 1.017357 | 1.000952 | 1.017352 |
| Indonesia | 1.014606 | 1.005406 | 1.005815 | 1.011053 | 1.013747 | 1.009365 | 1.027587 | 1.008416 | 1.024265 | 1.002684 | 0.9992579 | 1.002843 |
| Jordan | 1.000392 | 1.016535 | 1.01852 | 0.9996692 | 0.9999899 | 1.004014 | 1.001057 | 0.9997964 | 1.001864 | 1.017057 | 1.001636 | 1.000473 |
| Kazakhstan | 0.9993178 | 0.9996323 | 1.022697 | 1.001236 | 1.008683 | 1.003817 | 0.9994943 | 1.006849 | N/A | 1.002205 | 1.000353 | 1.011603 |
| Kenya | 1.003073 | 1.001038 | 1.00794 | 1.005129 | 1.00083 | 1.00032 | 1.00678 | 1.003872 | 1.000389 | 1.053068 | 1.006757 | 1.001188 |
| Kyrgyz Republic | 1.018695 | 0.9995868 | 1.006614 | 1.018887 | 1.004151 | 0.9993606 | 1.001279 | 1.005861 | 1.050365 | 1.00305 | 1.001719 | 0.9993349 |
| Lesotho | 1.044803 | 1.01316 | 1.003032 | 1.037879 | 1.005306 | 1.002973 | 1.001258 | 0.9994156 | 1.000404 | 1.000774 | 1.00915 | 1.007256 |
| Liberia | 1.015835 | 1.004358 | 1.007506 | 1.040306 | 1.003768 | 1.042042 | 1.083435 | 1.000305 | 1.005369 | 1.085661 | 1.018908 | 1.050286 |
| Madagascar | 1.044799 | 1.067115 | 1.011937 | 1.000334 | 1.011578 | 1.011201 | 1.003184 | 1.03589 | 1.036776 | 0.9996378 | 1.012228 | 1.061591 |
| Malawi | 1.000549 | 1.006463 | 1.001417 | 1.004796 | 1.002702 | 1.005699 | 1.003999 | 1.009007 | 1.003233 | 1.000138 | 1.003784 | 0.9995241 |
| Maldives | 1.013536 | 1.069525 | 1.026736 | 1.00761 | 1.001569 | 1.028627 | 0.9999424 | 1.039415 | 1.011982 | 1.00122 | 1.097989 | 1.004014 |
| Mali | 1.001301 | 1.000155 | 1.001387 | 0.9995229 | 1.002348 | 1.024874 | 1.005683 | 0.9992549 | 1.022671 | 0.9995713 | 1.000224 | 1.001994 |
| Morocco | 1.018893 | 1.01919 | 1.00902 | 1.00072 | 1.010505 | 0.9992163 | 0.999284 | 0.9995944 | 1.02246 | 1.001292 | 1.007462 | 1.001246 |
| Mozambique | 1.001473 | 1.032816 | 1.002978 | 0.9997033 | 1.006832 | 1.008132 | 0.9995453 | 1.014605 | 1.00033 | 1.001889 | 1.014305 | 0.9999185 |
| Namibia | 1.002057 | 1.013667 | 1.039158 | 0.9998687 | 1.007157 | 1.00719 | 1.00828 | 0.9996603 | 0.9999754 | 1.005728 | 1.002802 | 0.9993286 |
| Nepal | 1.025672 | 1.000863 | 0.9994687 | 1.003515 | 1.000656 | 1.008066 | 1.002533 | 1.011574 | 1.023242 | 1.003199 | 1.000199 | 0.9995322 |
| Nicaragua | 1.01676 | 0.9993919 | 0.999462 | 1.000894 | 1.014488 | 1.001425 | 1.000272 | 1.005747 | 1.002912 | 1.00566 | 1.002528 | 1.000305 |
| Niger | 1.007266 | 1.000793 | 1.005835 | 1.00234 | 0.9992958 | 1.004142 | 1.003116 | 1.001797 | 1.01573 | 0.9995949 | 1.013659 | 1.001321 |
| Nigeria | 1.002963 | 1.001956 | 1.015413 | 1.006084 | 1.009554 | 1.009774 | 1.00041 | 1.006203 | 0.9993117 | 1.004239 | 1.01524 | 1.001765 |
| Pakistan | 1.039564 | 1.00402 | 1.004773 | 1.012092 | 1.004078 | 1.000624 | 1.009344 | 1.014581 | 1.000606 | 1.003698 | 1.000962 | 0.9993654 |
| Peru | 1.010927 | 1.005867 | 1.005054 | 1.006599 | 1.005315 | 1.000675 | 1.009526 | 1.019481 | 1.00183 | 1.003261 | 1.009761 | 1.002421 |
| Philippines | 1.004697 | 1.008193 | 1.002087 | 1.026173 | 1.006485 | 1.004056 | 0.9994135 | 1.010616 | 1.000566 | 1.052944 | 1.00573 | 1.021301 |
| Rwanda | 1.005423 | 0.9994322 | 1.023225 | 1.002284 | 1.005406 | 0.9991889 | 1.001031 | 1.003233 | 0.9997191 | 1.002386 | 1.002389 | 1.025187 |
| Senegal | 1.009382 | 1.00005 | 1.002429 | 1.0061 | 1.005871 | 1.000578 | 0.9991702 | 1.000303 | 0.9995469 | 1.002607 | 1.013105 | 1.000173 |
| Sierra Leone | 1.002669 | 0.999952 | 1.000856 | 1.003068 | 0.9992709 | 1.001555 | 1.000471 | 1.003731 | 0.9995269 | 1.030853 | 1.005956 | 1.002988 |
| South Africa | 1.009093 | 1.001544 | 1.000655 | 1.001073 | 1.011101 | 1.002835 | 1.003069 | 0.9992995 | 1.002674 | 1.024476 | 0.9995735 | 1.000173 |
| Tajikistan | 0.9991766 | 1.011961 | 1.029845 | 1.019451 | 0.9994829 | 0.9995942 | 1.013431 | 1.000608 | 1.000312 | 1.001518 | 0.9994757 | 1.002657 |
| Tanzania | 1.004211 | 1.035478 | 0.9995115 | 1.010666 | 1.003154 | 0.9997061 | 1.002888 | 1.014499 | 1.005241 | 1.006157 | 1.027583 | 1.004735 |
| Timor-Leste | 1.016825 | 1.00182 | 1.003103 | 1.003631 | 1.009795 | 0.9999752 | 1.037407 | 1.002752 | 0.9994946 | 1.007245 | 1.001376 | 1.000652 |
| Togo | 1.003697 | 1.00122 | 0.9992966 | 1.000192 | 1.013902 | 1.02087 | 1.013402 | 1.016646 | 1.015746 | 1.009495 | 1.000309 | 1.001228 |
| Turkey | 1.008066 | 1.020561 | 1.007315 | 1.006949 | 1.017563 | 1.017038 | 0.9993733 | 1.000662 | 1.009353 | 1.000557 | 1.002176 | 1.00058 |
| Uganda | 1.00112 | 1.003043 | 1.006441 | 1.003671 | 1.000118 | 1.001159 | 1.002452 | 1.002159 | 1.000619 | 1.00756 | 0.9999751 | 1.000273 |
| Vietnam | 0.99953 | 1.027786 | 1.00117 | 1.010856 | 0.9997353 | 0.9994851 | 1.00723 | 1.006016 | 1.019044 | 1.00661 | 1.002337 | 1.006649 |
| Yemen | 1.003018 | N/A | N/A | N/A | N/A | N/A | 0.9996176 | 1.008255 | 1.018592 | 1.004283 | 1.001779 | 1.001568 |
| Zambia | 0.9991952 | 1.003921 | 1.000344 | 0.9995033 | 1.006317 | 0.9998224 | 1.000761 | 1.011927 | 1.008537 | 1.017541 | 1.012908 | 1.000641 |
| Zimbabwe | 1.007812 | 1.011959 | 1.000693 | 1.010681 | 1.000095 | 1.013534 | 1.012956 | 1.005732 | 1.001354 | 1.004287 | 1.03077 | 0.9996242 |

^1^The PSRF values were drawn from models when fitted for calculating the probability of attaining ≥75% coverage for mDFPS. N/A denotes not application and refers that the indicator has no sufficient data to make projections

Table S5: Potential scale reduction factor (PSRF) to diagnose convergence after Markov Chain Monte Carlo simulation in Bayesian regression model for antenatal care visits among women 15-49 years of age in low- and midle-income countries

| **Country** | **Overall** | **Poorest** | **Poorer** | **Middle** | **Richer** | **Richest** | **Urban** | **Rural** | **Below secondary** | **Secondary+** | **15-19 years** | **20-49 years** |
| --- | --- | --- | --- | --- | --- | --- | --- | --- | --- | --- | --- | --- |
| Afghanistan | 1.000805 | 1.001448 | 1.030994 | 1.001789 | 1.000834 | 1.011886 | 1.001276 | 1.019303 | 1.009026 | 0.9994851 | 1.003926 | 1.002627 |
| Albania | 1.006406 | 1.00714 | 1.000994 | 1.004682 | 1.003176 | 1.019407 | 1.003275 | 1.003477 | 0.9994832 | 1.014799 | 1.005991 | 1.003697 |
| Angola | 1.028885 | 1.019911 | 1.001714 | 1.003999 | 1.016468 | 1.010699 | 1.014573 | 1.002799 | 1.010434 | 1.086731 | 1.042451 | 1.004917 |
| Armenia | 1.00441 | 1.004743 | 1.03712 | 1.004427 | 1.000226 | 1.003661 | 1.058936 | 1.012226 | 0.9992062 | 1.000624 | 1.060446 | 1.032941 |
| Bangladesh | 1.005548 | 1.024462 | 1.011809 | 0.999846 | 1.004805 | 1.006587 | 1.00107 | 1.016399 | 1.001134 | 1.000882 | 1.029062 | 1.026309 |
| Benin | 1.002449 | 1.009576 | 1.002512 | 1.029311 | 1.015592 | 1.000187 | 1.01016 | 1.000751 | 1.008378 | 1.009326 | 1.007635 | 1.024787 |
| Bolivia | 1.003011 | 1.007114 | 1.007254 | 1.018833 | 1.036712 | 1.010858 | 1.07255 | 1.097542 | 1.010095 | 1.041831 | 1.039516 | 1.017256 |
| Brazil | 1.062975 | N/A | N/A | N/A | N/A | N/A | 1.036105 | 1.000327 | 1.000849 | 1.024664 | 1.051697 | 1.000284 |
| Burkina Faso | 1.01843 | 1.000195 | 1.004098 | 1.002831 | 1.000205 | 0.9999066 | 1.004902 | 1.018187 | 0.9993346 | 1.000903 | 1.00302 | 0.9998819 |
| Burundi | 1.012938 | 1.084612 | 1.048712 | 1.014467 | 1.002038 | 1.051308 | 0.9994192 | 1.030452 | 1.058432 | 1.010579 | 1.066812 | 1.000039 |
| Cambodia | 1.020923 | 1.002641 | 1.001717 | 1.014557 | 1.010047 | 1.005199 | 1.003937 | 1.05754 | 1.03018 | 1.065063 | 1.015852 | 1.012171 |
| Cameroon | 0.999714 | 0.999364 | 1.008716 | 1.023295 | 1.033758 | 1.011395 | 1.014013 | 0.9994506 | 1.013409 | 1.015475 | 1.000795 | 1.010978 |
| Chad | 0.9996603 | 1.012595 | 1.02132 | 1.048817 | 1.001326 | 1.00855 | 1.003289 | 0.9999445 | 1.024902 | 1.021274 | 1.002369 | 1.022936 |
| Colombia | 1.000708 | 1.002377 | 0.9996564 | 1.00338 | 1.001027 | 0.9993283 | 1.000644 | 1.015114 | 1.004464 | 1.05143 | 1.001426 | 1.013128 |
| Comoros | 1.014465 | 1.000384 | 1.031047 | 1.028215 | 0.9994344 | 1.012691 | 1.004986 | 1.001393 | 1.042419 | 1.029131 | 1.006905 | 1.005535 |
| Congo | 1.007939 | 1.001682 | 1.018637 | 1.017256 | 1.001198 | 1.00994 | 1.023459 | 1.057448 | 1.030844 | 1.00231 | 1.005986 | 1.022969 |
| Congo DR | 1.002872 | 1.001435 | 1.00564 | 1.002094 | 1.021438 | 1.011437 | 1.023628 | 1.003057 | 1.002064 | 1.008868 | 0.9993429 | 1.015452 |
| Cote d'Ivoire | 1.002 | 1.014286 | 1.012323 | 1.004343 | 1.094188 | 1.021123 | 1.027172 | 1.013081 | 1.048914 | 1.000113 | 1.004942 | 1.007887 |
| Dominican Republic | 1.005342 | 1.000783 | 1.001107 | 1.005129 | 1.000763 | 1.013548 | 1.011984 | 1.009419 | 1.01372 | 1.002594 | 1.002044 | 1.000018 |
| Egypt | 1.013796 | 1.008028 | 1.021899 | 1.011897 | 1.001433 | 1.01281 | 1.001733 | 1.003067 | 1.028494 | 1.02924 | 1.013571 | 1.002292 |
| Ethiopia | 1.005742 | 1.001645 | 1.011578 | 1.019697 | 1.004495 | 1.017712 | 1.011336 | 1.039043 | 1.004553 | 1.007352 | 1.008044 | 0.999461 |
| Gabon | 0.999214 | 1.000142 | 1.06481 | 1.012721 | 1.087819 | 1.024955 | 1.047755 | 1.00303 | 1.021543 | 1.013697 | 1.000716 | 1.034493 |
| Ghana | 1.00071 | 1.002665 | 1.00254 | 1.031985 | 1.00164 | 1.002534 | 1.006966 | 1.054867 | 1.001023 | 1.00337 | 1.029123 | 1.002116 |
| Guatemala | 1.036774 | 1.012987 | 1.062272 | 1.016387 | 1.004966 | 1.030277 | 1.021511 | 1.006985 | 0.9996622 | 1.009454 | 1.000719 | 1.003413 |
| Guinea | 1.007202 | 1.049424 | 1.007515 | 1.005333 | 1.028275 | 1.014435 | 1.001205 | 1.000926 | 1.001515 | 1.000321 | 1.000548 | 1.011184 |
| Guyana | 1.002212 | 1.072039 | 1.030799 | 1.040933 | 1.001287 | 1.079169 | 1.000599 | 1.002779 | 1.000865 | 0.9999581 | 0.9997108 | 1.016585 |
| Haiti | 1.024311 | 1.007698 | 1.00655 | 1.000331 | 1.000321 | 1.00003 | 1.011774 | 1.009262 | 1.034181 | 1.004098 | 1.095806 | 1.00289 |
| Honduras | 1.001306 | 1.003939 | 1.036481 | 1.03004 | 1.082242 | 1.015416 | 1.002009 | 1.023435 | 1.018295 | 1.005007 | 1.024757 | 0.9998338 |
| India | 1.005213 | 1.013233 | 1.000216 | 1.020441 | 1.005805 | 0.9993201 | 1.010729 | 1.001716 | 1.008781 | 1.001672 | 1.007145 | 1.013498 |
| Indonesia | 0.9994232 | 1.010406 | 1.004393 | 1.022243 | 1.002323 | 1.015921 | 1.009548 | 1.00692 | 0.9999339 | 1.006623 | 1.033365 | 1.002049 |
| Jordan | 1.024652 | 1.000698 | 1.036582 | 1.025525 | 0.9993562 | 1.000352 | 1.000069 | 1.00368 | 1.04732 | 1.002569 | 1.021323 | 1.005374 |
| Kazakhstan | 1.000338 | 1.024065 | 1.000406 | 1.006528 | 1.001034 | 1.010201 | 0.9992539 | 1.007779 | N/A | 1.003867 | N/A | 1.009143 |
| Kenya | 1.003301 | 1.004349 | 1.002917 | 1.005013 | 1.005 | 1.004503 | 1.004353 | 1.010148 | 1.000575 | 1.004348 | 1.001639 | 1.0022 |
| Kyrgyz Republic | 1.038976 | 0.9998324 | 1.010719 | 1.019501 | 0.9996053 | 1.053565 | 1.044275 | 1.004068 | 1.072071 | 1.000348 | 1.007672 | 1.007472 |
| Lesotho | 1.000479 | 1.006428 | 1.011787 | 1.035128 | 1.01126 | 1.075618 | 1.004812 | 1.039299 | 1.002665 | 0.9998716 | 1.006733 | 1.002463 |
| Liberia | 1.03758 | 1.012788 | 1.010642 | 1.004499 | 1.020104 | 1.010209 | 1.008201 | 1.027998 | 1.005406 | 1.040186 | 1.019792 | 1.019822 |
| Madagascar | 1.00203 | 1.000478 | 1.09979 | 1.00297 | 1.002831 | 1.00153 | 1.00767 | 1.001835 | 1.004808 | 1.002701 | 1.002513 | 1.016514 |
| Malawi | 1.017269 | 1.019299 | 0.9992331 | 1.003928 | 0.9992898 | 1.003039 | 1.043161 | 1.028537 | 1.009641 | 1.003961 | 1.007142 | 1.018051 |
| Maldives | 1.072895 | 1.006697 | 1.002175 | 1.028956 | 1.057844 | 1.083569 | 1.009394 | 1.005078 | 1.071744 | 1.008702 | 1.003857 | 1.049862 |
| Mali | 1.014522 | 0.9997703 | 1.012879 | 1.009867 | 1.002344 | 1.096426 | 1.005306 | 1.014251 | 1.002866 | 0.9992359 | 1.005886 | 1.01276 |
| Morocco | 1.005296 | 1.033234 | 1.000731 | 1.031447 | 1.010212 | 1.020156 | 1.0087 | 1.004567 | 1.051829 | 1.002797 | 1.039583 | 1.027122 |
| Mozambique | 1.010048 | 1.002825 | 1.00157 | 1.000256 | 1.001211 | 1.003036 | 1.000854 | 1.023555 | 1.002339 | 1.020465 | 1.001338 | 1.005371 |
| Namibia | 1.048826 | 0.9994788 | 1.003711 | 1.015876 | 1.014932 | 1.004704 | 1.04512 | 1.005366 | 1.023434 | 1.002229 | 1.026702 | 1.007827 |
| Nepal | 1.005625 | 1.004759 | 1.046113 | 1.000919 | 1.009059 | 1.002443 | 1.012135 | 1.014497 | 1.007705 | 1.036751 | 1.018507 | 1.007926 |
| Nicaragua | 1.000484 | 0.9996693 | 1.009223 | 1.02422 | 1.003728 | 0.9995508 | 1.000681 | 1.013091 | 1.006271 | 0.9998788 | 1.002692 | 1.001146 |
| Niger | 1.076159 | 1.007296 | 1.000375 | 1.002568 | 1.004209 | 1.001779 | 1.009768 | 1.016605 | 1.033244 | 1.002509 | 1.006044 | 1.00075 |
| Nigeria | 1.005813 | 1.020404 | 1.014519 | 1.00772 | 1.024105 | 0.9999961 | 1.009945 | 1.003575 | 1.007824 | 1.008331 | 1.038651 | 1.00003 |
| Pakistan | 1.03683 | 1.011098 | 1.022161 | 1.008098 | 1.003992 | 1.001702 | 1.00011 | 1.000005 | 1.005226 | 1.010141 | 1.013138 | 1.00503 |
| Peru | 1.000039 | 1.00261 | 1.004005 | 1.001038 | 1.002094 | 1.021638 | 1.002763 | 1.004236 | 1.014508 | 1.008091 | 1.00546 | 1.000675 |
| Philippines | 1.015838 | 1.011174 | 1.000175 | 1.000623 | 1.004221 | 1.012079 | 1.000948 | 1.00482 | 1.00064 | 1.001349 | 1.008235 | 1.019862 |
| Rwanda | 1.019119 | 1.016316 | 1.048386 | 1.008057 | 1.001631 | 1.00814 | 1.010517 | 1.025542 | 1.00102 | 1.000778 | 1.01714 | 1.001058 |
| Senegal | 1.011174 | 1.001739 | 1.020207 | 1.020895 | 1.000624 | 1.019096 | 1.015422 | 1.017844 | 1.03565 | 1.007721 | 1.007289 | 1.00873 |
| Sierra Leone | 0.9999927 | 0.9993066 | 1.000836 | 0.9997138 | 1.006884 | 1.002287 | 1.005234 | 1.008031 | 0.9992197 | 1.013928 | 1.001595 | 0.999924 |
| South Africa | 1.000385 | 1.000732 | 1.011387 | 1.017909 | 1.028803 | 1.077707 | 1.002302 | 1.009668 | 1.00974 | 1.008174 | 1.017787 | 1.020859 |
| Tajikistan | 1.00608 | 1.012874 | 1.002644 | 0.9998493 | 1.02661 | 1.004227 | 1.071399 | 1.015328 | 0.9998392 | 1.019315 | 0.9993224 | 0.9998002 |
| Tanzania | 1.002856 | 1.002484 | 1.001843 | 1.002426 | 1.003496 | 1.015531 | 1.001938 | 1.000082 | 1.014653 | 1.002048 | 1.004054 | 1.026232 |
| Timor-Leste | 1.001703 | 1.00038 | 1.009373 | 1.005376 | 1.001832 | 1.021008 | 1.010295 | 1.003621 | 1.001945 | 0.9992638 | 1.0001 | 0.9999554 |
| Togo | 1.00021 | 1.022501 | 1.011715 | 1.019326 | 1.006863 | 1.000641 | 1.055538 | 1.001214 | 1.021727 | 1.012005 | 1.038477 | 1.001649 |
| Turkey | 1.03959 | 1.001651 | 1.014817 | 1.053515 | 0.9992698 | 1.006526 | 1.034751 | 1.002846 | 0.9998668 | 1.000171 | 1.013961 | 1.038868 |
| Uganda | 1.018245 | 1.004205 | 1.002132 | 1.023646 | 1.009779 | 1.002563 | 1.01048 | 1.001041 | 1.013605 | 1.006582 | 1.003536 | 0.9994961 |
| Vietnam | 1.035515 | 1.001568 | 1.085911 | 1.071839 | 1.036296 | 1.063625 | 1.021913 | 0.999746 | 1.01985 | 1.034919 | 1.006275 | 1.00051 |
| Yemen | 1.016323 | N/A | N/A | N/A | N/A | N/A | 1.004386 | 1.008202 | 0.9992613 | 0.9993464 | 1.002628 | 1.001264 |
| Zambia | 1.014259 | 1.009236 | 1.001557 | 0.9992146 | 1.009026 | 0.9998764 | 1.014423 | 1.033142 | 1.000423 | 1.02226 | 1.010384 | 1.004117 |
| Zimbabwe | 1.002363 | 1.000741 | 1.001315 | 1.00862 | 0.9995705 | 1.00043 | 1.015745 | 1.001567 | 1.006663 | 1.000155 | 1.001299 | 1.008115 |

**Note:** N/A denotes not application and refers that the indicator has no sufficient data to make projections

Table S6: Potential scale reduction factor (PSRF) to diagnose convergence after Markov Chain Monte Carlo simulation in Bayesian regression model for skilled birth attendance among women 15-49 years of age in low- and midle-income countries

| **Country** | **Overall** | **Poorest** | **Poorer** | **Middle** | **Richer** | **Richest** | **Urban** | **Rural** | **Below secondary** | **Secondary+** | **15-19 years** | **20-49 years** |
| --- | --- | --- | --- | --- | --- | --- | --- | --- | --- | --- | --- | --- |
| Afghanistan | 1.003603 | 0.9996147 | 1.017878 | 1.000743 | 1.001225 | 0.9999297 | 1.000691 | 1.000471 | 1.014081 | 1.001698 | 1.018146 | 1.003174 |
| Albania | 1.001576 | 1.003888 | 0.9991862 | 0.9999032 | 1.011421 | 1.044755 | 1.000711 | 1.00412 | 1.003394 | 0.9999431 | 1.000911 | 1.00264 |
| Angola | 1.002152 | 1.001177 | 1.024577 | 1.028046 | 1.034017 | 1.047396 | 1.000788 | 1.009654 | 1.076615 | 1.084095 | 1.012128 | 1.000951 |
| Armenia | 1.016327 | 0.9993234 | 1.05263 | 1.011152 | 1.007904 | 1.047396 | 1.015872 | 1.005865 | 1.0566 | 1.008211 | 1.000257 | 1.030301 |
| Bangladesh | 1.010645 | 1.005154 | 1.001758 | 1.017266 | 1.019763 | 0.9994376 | 1.001591 | 1.004292 | 1.007052 | 1.00439 | 1.001494 | 1.012068 |
| Benin | 1.000327 | 1.001594 | 1.027385 | 1.001757 | 1.053648 | 1.089128 | 1.003457 | 1.008393 | 1.010893 | 1.063615 | 0.9993317 | 1.002069 |
| Bolivia | 1.00203 | 1.009344 | 0.9999005 | 1.081828 | 1.004683 | 1.007737 | 1.003891 | 1.09596 | 1.001631 | 1.003921 | 1.007892 | 1.000863 |
| Brazil | 1.044513 | N/A | N/A | N/A | N/A | N/A | 1.041918 | 1.004181 | 1.013432 | 1.007406 | 1.037494 | 1.087936 |
| Burkina Faso | 1.005271 | 1.004384 | 1.000165 | 1.003529 | 1.007967 | 1.025926 | 1.025182 | 1.000373 | 1.000498 | 1.001804 | 1.003624 | 1.03331 |
| Burundi | 1.011973 | 1.013823 | 1.065997 | 0.9999072 | 1.026681 | 1.067825 | 1.075195 | 1.004732 | 1.000383 | 1.074568 | 1.019609 | 1.093618 |
| Cambodia | 1.025739 | 1.007459 | 1.008166 | 1.000414 | 1.022279 | 1.022452 | 1.006017 | 1.007565 | 1.012349 | 1.039214 | 1.000639 | 1.075107 |
| Cameroon | 1.007886 | 1.025464 | 1.00669 | 1.032166 | 0.9993678 | 0.9995532 | 1.003899 | 1.001523 | 1.004226 | 1.002851 | 1.000518 | 1.003674 |
| Chad | 1.000819 | 1.0057 | 1.005485 | 1.002295 | 1.006488 | 1.003086 | 1.038388 | 1.011443 | 1.014011 | 1.017235 | 0.9997184 | 1.000753 |
| Colombia | 1.003132 | 1.001632 | 1.040648 | 1.001498 | 1.000307 | 1.006529 | 1.002881 | 1.006815 | 1.000646 | 1.002474 | 1.005709 | 1.024716 |
| Comoros | 1.004266 | 1.01063 | 1.009038 | 1.017976 | 1.009205 | 1.025315 | 1.002185 | 1.036946 | 1.024649 | 1.00404 | 1.044532 | 0.999512 |
| Congo | 1.000392 | 1.002541 | 1.0097 | 0.9994535 | 1.007756 | 1.005526 | 1.02517 | 1.002404 | 1.001588 | 1.063901 | 1.003692 | 1.005898 |
| Congo DR | 0.9998116 | 1.027358 | 1.007086 | 1.002145 | 1.000138 | 1.005201 | 1.017123 | 0.9993498 | 1.017217 | 1.006456 | 1.000373 | 1.001369 |
| Cote d'Ivoire | 1.000998 | 1.003447 | 1.001405 | 0.9997513 | 1.06154 | 0.9993275 | 1.019689 | 0.9998526 | 1.000618 | 1.015587 | 1.004879 | 1.002703 |
| Dominican Republic | 1.018209 | 1.004962 | 1.002602 | 1.0113 | 1.010082 | 1.008147 | 1.013476 | 1.000193 | 1.056604 | 1.002612 | 1.017127 | 1.013991 |
| Egypt | 1.007277 | 1.010407 | 1.015864 | 1.004007 | 1.019932 | 1.035196 | 1.001305 | 1.010453 | 1.008707 | 1.001187 | 1.001268 | 0.99997 |
| Ethiopia | 0.9996221 | 1.003579 | 1.000725 | 0.9996732 | 1.003829 | 1.019901 | 1.004328 | 0.9995841 | 1.001077 | 1.012926 | 1.000129 | 1.003752 |
| Gabon | 1.0007 | 1.000171 | 1.036593 | 1.009655 | 1.002739 | 0.9999396 | 1.000358 | 1.004641 | 1.022458 | 1.000715 | 1.006936 | 0.9992404 |
| Ghana | 1.001379 | 1.004264 | 1.035109 | 1.002854 | 1.004716 | 1.000117 | 1.018084 | 1.001275 | 0.9997378 | 1.00896 | 0.9992827 | 1.000074 |
| Guatemala | 1.00733 | 1.000077 | 1.018386 | 0.9996839 | 1.003423 | 1.006364 | 1.008775 | 1.009758 | 1.03009 | 1.012233 | 1.085527 | 1.087086 |
| Guinea | 1.002945 | 1.006538 | 1.005129 | 0.999767 | 1.008138 | 1.039723 | 1.017955 | 0.9993711 | 1.00239 | 1.001387 | 1.000965 | 1.035732 |
| Haiti | 1.011591 | 1.00156 | 1.003791 | 1.016611 | 1.000737 | 1.009037 | 0.9993824 | 1.003572 | 1.044139 | 1.003302 | 1.011646 | 1.002744 |
| Honduras | 1.041846 | 1.000754 | 1.018859 | 1.031478 | 1.079159 | 1.026066 | 1.053199 | 1.007202 | 1.009315 | 1.001902 | 1.073521 | 1.095795 |
| India | 1.002104 | 1.017342 | 1.00424 | 1.007016 | 1.001876 | 1.001368 | 1.019505 | 1.000612 | 1.002916 | 1.002246 | 1.003991 | 0.9992175 |
| Indonesia | 1.002882 | 0.9995309 | 0.9998189 | 1.00139 | 1.002209 | 1.003286 | 0.9999627 | 1.000198 | 0.9996879 | 0.9996369 | 1.011025 | 1.004137 |
| Jordan | 1.003048 | 1.001876 | 1.083079 | 1.027213 | 1.033931 | 1.083854 | 1.009502 | 1.03173 | 1.02164 | 1.012125 | 1.009557 | 1.032162 |
| Kazakhstan | 1.006159 | 1.000117 | 1.010147 | 1.043301 | 1.036661 | 1.063165 | 1.062458 | 1.004289 | 1.003463 | 1.004289 | 1.001562 | 1.005479 |
| Kenya | 0.9993819 | 1.005109 | 0.999378 | 1.002765 | 1.001547 | 1.011356 | 0.9998245 | 0.9997567 | 1.006329 | 1.000766 | 1.00345 | 1.000795 |
| Kyrgyz Republic | 1.078124 | 1.028853 | 1.001952 | 1.002576 | 1.033981 | 1.019269 | 1.034348 | 1.004561 | 1.005906 | 1.022748 | 0.9996236 | 1.003366 |
| Lesotho | 1.040217 | 1.016322 | 1.000399 | 1.012041 | 1.025324 | 1.019587 | 1.002244 | 1.00306 | 0.9998888 | 1.000633 | 1.028899 | 1.012386 |
| Liberia | 1.009559 | 1.003973 | 1.001291 | 1.022654 | 1.007489 | 1.004521 | 1.011172 | 1.002336 | 1.003906 | 1.003671 | 1.00523 | 1.005706 |
| Madagascar | 1.043375 | 0.9992638 | 1.013356 | 0.9999008 | 0.9994124 | 1.003321 | 0.9998138 | 1.001186 | 1.040462 | 1.016732 | 0.9994153 | 0.9997374 |
| Malawi | 1.013399 | 1.003058 | 1.003785 | 1.000166 | 1.006011 | 1.000269 | 1.001105 | 1.001068 | 1.017264 | 1.011881 | 0.9994954 | 1.01536 |
| Maldives | 1.012926 | 1.030336 | 1.007785 | 1.000156 | 1.005441 | 1.000253 | 1.005196 | 1.010283 | 0.9996254 | 1.001787 | 1.00917 | 0.9999028 |
| Mali | 1.012167 | 1.016078 | 1.00237 | 0.9992144 | 1.001489 | 1.002219 | 1.007931 | 1.013991 | 1.000509 | 1.030757 | 1.009475 | 0.9992986 |
| Morocco | 1.010327 | 1.014838 | 1.014305 | 1.024232 | 1.090145 | 1.042926 | 1.049115 | 1.009155 | 0.9996741 | 1.041412 | 1.003487 | 1.004762 |
| Mozambique | 1.02185 | 1.012712 | 1.027138 | 1.016307 | 0.9997163 | 0.9993505 | 1.014299 | 1.007565 | 0.9998498 | 0.9994685 | 1.001389 | 1.001076 |
| Namibia | 1.000664 | 1.034072 | 1.00375 | 1.002016 | 1.048279 | 1.00601 | 1.043659 | 1.006145 | 1.004261 | 1.010345 | 1.01055 | 0.9998718 |
| Nepal | 1.002397 | 1.00068 | 1.005216 | 1.009606 | 1.001187 | 1.008827 | 1.013593 | 1.003159 | 1.016309 | 1.000235 | 1.004553 | 1.009744 |
| Nicaragua | 0.9993919 | 1.014986 | 1.039852 | 1.006639 | 1.007118 | 1.029518 | 1.002152 | 1.002043 | 1.00002 | 1.02176 | 1.01628 | 1.004202 |
| Niger | 1.002706 | 1.012636 | 1.003883 | 1.00359 | 1.001442 | 1.012049 | 1.008062 | 1.009653 | 1.008761 | 1.04261 | 1.006058 | 1.007803 |
| Nigeria | 1.002303 | 1.031582 | 1.005307 | 1.000794 | 1.000197 | 1.019415 | 1.007893 | 0.9998105 | 1.045107 | 1.011268 | 1.006082 | 1.002659 |
| Pakistan | 1.01341 | 1.000813 | 1.01824 | 0.9993221 | 1.015368 | 1.003251 | 0.9999166 | 1.010936 | 1.014104 | 1.012218 | 1.0002 | 1.004362 |
| Peru | 1.004726 | 1.000945 | 1.013538 | 1.007428 | 1.03806 | 1.02345 | 1.009909 | 1.012286 | 1.013879 | 1.030815 | 1.031588 | 1.006537 |
| Philippines | 0.9997876 | 0.9997261 | 1.017418 | 0.9996538 | 1.000568 | 1.009317 | 1.007728 | 1.016491 | 1.020437 | 1.00122 | 1.01198 | 1.018825 |
| Rwanda | 1.000867 | 1.02303 | 1.009137 | 1.020003 | 0.9992597 | 1.015325 | 0.9993774 | 1.000212 | 1.004019 | 0.999831 | 1.000734 | 1.003616 |
| Senegal | 1.0046 | 1.002598 | 1.01026 | 1.00499 | 1.011423 | 1.024055 | 1.001363 | 1.002981 | 1.020582 | 1.007289 | 1.001026 | 1.00144 |
| Sierra Leone | 1.006112 | 1.012491 | 1.003305 | 1.004363 | 1.001824 | 0.9997267 | 1.000793 | 1.013163 | 1.029054 | 1.028115 | 1.002188 | 1.002968 |
| South Africa | 1.02318 | 0.9999482 | 1.014961 | 1.003998 | 0.9996167 | 1.007627 | 1.023632 | 1.026806 | 1.039235 | 1.012776 | 1.010976 | 1.004599 |
| Tajikistan | 1.000352 | 1.071311 | 1.032417 | 1.008938 | 1.090042 | 1.004553 | 1.021797 | 1.005078 | 1.029044 | 1.000879 | 0.9992132 | 1.003739 |
| Tanzania | 0.9999332 | 1.003818 | 1.002152 | 0.9999914 | 1.015801 | 1.011404 | 1.014819 | 1.001857 | 1.003743 | 0.9992714 | 1.007576 | 0.9998071 |
| Timor-Leste | 1.020653 | 1.041423 | 1.000909 | 1.063491 | 1.008373 | 1.013778 | 1.001002 | 0.9993026 | 1.008862 | 1.001713 | 1.003229 | 1.003674 |
| Togo | 1.000952 | 1.002569 | 1.037714 | 1.005631 | 1.009343 | 1.001918 | 1.00104 | 1.01093 | 1.002464 | 1.041685 | 1.028543 | 1.001015 |
| Turkey | 1.00752 | 1.015568 | 1.002595 | 1.023443 | 1.028726 | 1.039694 | 1.00488 | 1.01029 | 1.013629 | 1.017385 | 1.008446 | 1.00142 |
| Uganda | 1.019689 | 1.004413 | 0.9999918 | 1.00953 | 1.072812 | 1.014681 | 1.009186 | 1.014387 | 1.000293 | 1.028853 | 1.004556 | 1.008441 |
| Vietnam | 1.025038 | 1.003083 | 1.076676 | 1.043865 | 1.033832 | 1.046128 | 1.042185 | 1.065146 | 1.008021 | 1.088223 | 1.08427 | 1.013475 |
| Yemen | 1.006435 | N/A | N/A | N/A | N/A | N/A | 0.9994706 | 0.9992995 | 1.027789 | 1.002646 | 1.001294 | 1.007929 |
| Zambia | 1.005414 | 1.000575 | 1.005738 | 0.9997761 | 1.061207 | 1.019853 | 1.014455 | 0.999436 | 1.019388 | 1.015939 | 1.00113 | 0.9994918 |
| Zimbabwe | 1.0053 | 1.000699 | 1.000256 | 1.0054 | 1.006539 | 1.021158 | 1.003661 | 1.000115 | 1.001269 | 1.013641 | 1.00364 | 1.010698 |

**Note:** N/A denotes not application and refers that the indicator has no sufficient data to make projections

Table S7: Potential scale reduction factor (PSRF) to diagnose convergence after Markov Chain Monte Carlo simulation in Bayesian regression model for BCG immunization among children 12-23 months of age in low- and midle-income countries

| **Country** | **Overall** | **Poorest** | **Poorer** | **Middle** | **Richer** | **Richest** | **Urban** | **Rural** | **Below secondary** | **Secondary+** | **15-19 years** | **20-49 years** | **Male** | **Female** |
| --- | --- | --- | --- | --- | --- | --- | --- | --- | --- | --- | --- | --- | --- | --- |
| Albania | 1.090837 | 1.069262 | 1.024895 | 1.002206 | 1.022722 | 1.027801 | 1.070382 | 1.020212 | 1.004027 | 1.004614 | 0.988054 | 1.05238 | 1.005451 | 1.029415 |
| Armenia | 1.024524 | 1.011829 | 1.058875 | 1.018428 | 1.040968 | 1.0592 | 1.002373 | 1.002727 | 0.9992333 | 1.021163 | 1.002327 | 1.058194 | 0.9994622 | 1.025363 |
| Bangladesh | 1.002019 | 1.01657 | 1.01185 | 1.011938 | 1.000117 | 1.007136 | 1.001878 | 1.032906 | 1.015185 | 1.004391 | 1.001761 | 1.026344 | 1.006376 | 1.010915 |
| Benin | 1.024513 | 1.002497 | 1.002586 | 1.000266 | 1.008959 | 0.9994162 | 1.0034 | 1.002784 | 1.004035 | 1.007692 | 1.018859 | 0.9994534 | 1.009175 | 1.000531 |
| Bolivia | 1.007149 | 1.045034 | 1.01303 | 1.00197 | 1.033793 | 1.075827 | 1.011505 | 1.0018 | 1.021727 | 1.03451 | 1.002895 | 1.004428 | 1.059638 | 1.068528 |
| Brazil | 1.019519 | N/A | N/A | N/A | N/A | N/A | 1.016051 | 1.094112 | 1.013766 | 1.030874 | 1.008833 | 1.049371 | 1.037899 | 1.051184 |
| Burkina Faso | 1.035146 | 0.9996479 | 1.005553 | 1.008114 | 1.002534 | 1.002854 | 1.065186 | 0.9998344 | 1.001568 | 1.0031 | 1.026327 | 1.005093 | 1.014977 | 0.9997628 |
| Burundi | 1.000549 | 1.009849 | 1.000577 | 1.000143 | 1.002226 | 1.003626 | 1.00351 | 1.002432 | 1.003559 | 1.035567 | 1.096403 | 0.9992652 | 1.011213 | 0.9999117 |
| Cambodia | 1.002286 | 1.022736 | 1.016801 | 1.042346 | 1.007045 | 1.023946 | 1.029796 | 1.011832 | 1.00133 | 1.008279 | 0.9997709 | 1.006964 | 1.076697 | 1.015886 |
| Cameroon | 1.001876 | 1.006285 | 1.008432 | 1.009986 | 1.00399 | 1.004355 | 1.055165 | 1.010477 | 1.006903 | 1.025582 | 1.000859 | 1.000599 | 1.011225 | 1.042976 |
| Chad | 1.000478 | 0.9997894 | 1.004743 | 1.00228 | 1.022367 | 1.00668 | 0.9996515 | 0.9997848 | 1.002819 | 1.002512 | 1.001347 | 1.006973 | 1.000074 | 1.037856 |
| Colombia | 1.035089 | 1.016579 | 1.008019 | 1.010783 | 1.02677 | 1.022559 | 1.019158 | 1.007602 | 1.007272 | 1.012554 | 1.029589 | 1.001566 | 1.028883 | 1.001501 |
| Comoros | 1.067841 | 1.000627 | 1.004797 | 0.9995676 | 1.004891 | 1.00264 | 1.003726 | 1.003792 | 1.000912 | 1.016909 | 1.017287 | 1.009697 | 1.004834 | 0.9996017 |
| Congo | 1.007719 | 1.000791 | 1.047326 | 1.020407 | 1.011487 | 1.006086 | 1.035485 | 1.012554 | 1.007757 | 1.001488 | 1.005633 | 1.002786 | 0.9999586 | 1.011681 |
| Congo DR | 1.007875 | 0.9997094 | 1.00026 | 1.026333 | 1.001867 | 1.026167 | 1.079511 | 1.001504 | 1.025876 | 0.9999338 | 1.018261 | 1.004978 | 1.004905 | 1.020878 |
| Cote d'Ivoire | 1.003603 | 0.9992469 | 0.9996523 | 1.039182 | 1.020574 | 0.9998945 | 1.001636 | 1.000229 | 1.00749 | 0.9995644 | 1.015053 | 1.000495 | 1.001939 | 1 |
| Dominican Republic | 1.019637 | 1.002907 | 1.031974 | 1.003955 | 0.9998796 | 1.001962 | 1.008824 | 1.019417 | 0.9994084 | 1.002285 | 1.008491 | 1.001404 | 1.005486 | 1.009502 |
| Egypt | 1.001231 | 1.071244 | 1.001043 | 0.9996321 | 1.023525 | 1.014139 | 1.022715 | 1.00248 | 0.9994189 | 1.02085 | 1.014492 | 1.003475 | 1.000565 | 1.042428 |
| Ethiopia | 0.999186 | 1.001199 | 1.007217 | 1.002402 | 1.028706 | 1.002033 | 0.9996616 | 1.003975 | 1.000368 | 1.007899 | 1.005353 | 1.003768 | 1.015893 | 1.004692 |
| Gabon | 1.00006 | 1.027284 | 1.024924 | 1.000209 | 1.017186 | 1.004596 | 1.03654 | 1.001693 | 1.006875 | 1.01316 | 1.001195 | 1.012397 | 1.005046 | 1.00465 |
| Ghana | 1.000489 | 1.0176 | 1.000893 | 1.001608 | 1.047989 | 1.010479 | 1.013566 | 1.001575 | 1.000764 | 1.006773 | 1.000682 | 1.055852 | 1.007969 | 1.014377 |
| Guatemala | 1.00776 | 1.015666 | 1.001586 | 1.013105 | 1.004828 | 0.9996727 | 1.003309 | 1.006047 | 1.057918 | 1.032494 | 0.9997189 | 1.000991 | 1.00227 | 1.004461 |
| Guinea | 1.003957 | 1.001453 | 1.000687 | 1.000978 | 1.014467 | 1.001019 | 1.000233 | 1.010076 | 0.9992982 | 1.005251 | 1.015716 | 1.010077 | 1.027809 | 1.013758 |
| Haiti | 1.002081 | 1.004297 | 1.009758 | 1.005187 | 1.018398 | 1.004739 | 1.041224 | 1.0216 | 1.003751 | 1.015702 | 1.003226 | 1.00746 | 1.008734 | 1.010352 |
| Honduras | 1.08246 | 1.02216 | 1.082375 | 1.002906 | 1.01317 | 1.030062 | 1.00164 | 1.030149 | 1.092817 | 1.090884 | 1.076901 | 1.08929 | 1.090322 | 1.04082 |
| India | 1.005156 | 1.016368 | 1.00819 | 1.001268 | 1.002092 | 1.013907 | 1.02735 | 1.000321 | 1.001417 | 1.028474 | 1.003834 | 1.07011 | 1.020412 | 1.000331 |
| Indonesia | 1.010027 | 1.000756 | 1.005418 | 1.022203 | 1.003659 | 1.013798 | 1.022106 | 1.009015 | 1.007202 | 1.035 | 1.018697 | 1.035491 | 1.000771 | 1.031091 |
| Jordan | 1.002046 | 1.003767 | 1.008263 | 1.047908 | 1.004967 | 1.013478 | 1.020624 | 1.000784 | 0.9998591 | 1.001523 | 1.033575 | 1.020539 | 0.9995778 | 1.001324 |
| Kazakhstan | 1.06735 | 1.004519 | 1.001578 | 1.000459 | 1.000904 | 0.9999279 | 1.038811 | 1.006663 | N/A | 1.067947 | 1.000006 | 1.007863 | 1.014076 | 1.001869 |
| Kenya | 1.001054 | 1.018403 | 1.023575 | 1.024754 | 1.007621 | 1.064923 | 1.000894 | 1.010637 | 1.002699 | 1.007358 | 1.071414 | 1.000571 | 1.008924 | 1.012217 |
| Kyrgyz Republic | 1.037901 | 1.059861 | 1.020404 | 1.003192 | 1.010061 | 1.00142 | 1.002823 | 1.001219 | 1.009614 | 0.99949 | 1.030138 | 1.027849 | 1.0033 | 1.015287 |
| Lesotho | 1.047136 | 1.001692 | 0.9992012 | 1.003604 | 1.005246 | 1.002559 | 1.031485 | 1.004232 | 1.00484 | 1.094781 | 1.011233 | 1.018707 | 1.00123 | 1.024557 |
| Liberia | 1.006966 | 1.043383 | 1.090108 | 1.065301 | 1.000775 | 1.001806 | 1.002249 | 0.999312 | 1.000564 | 1.002912 | 1.02737 | 1.067558 | 0.9998224 | 1.003888 |
| Madagascar | 1.002077 | 1.001201 | 1.000757 | 1.006226 | 1.088849 | 1.00449 | 1.03806 | 1.014685 | 1.000186 | 1.072019 | 1.001454 | 1.02233 | 1.000892 | 1.012517 |
| Malawi | 1.017547 | 1.030125 | 0.99951 | 0.9993765 | 1.005677 | 1.004786 | 0.9994774 | 1.017319 | 1.004503 | 1.006814 | 1.003639 | 1.001233 | 0.9998448 | 1.004929 |
| Maldives | 1.00805 | 0.99932 | 1.001438 | 0.9992273 | 1.001015 | 1.020196 | 1.004559 | 0.9991692 | 1.001478 | 1.002122 | 1.00009 | 1.008673 | 1.02116 | 1.00346 |
| Mali | 1.011719 | 1.000262 | 1.001626 | 0.9997094 | 1.034834 | 1.001536 | 0.9996343 | 1.010628 | 1.015763 | 1.015047 | 1.007151 | 1.005352 | 0.9999495 | 1.001862 |
| Morocco | 1.063267 | 1.076106 | 1.027226 | 1.033546 | 1.009757 | 1.008466 | 0.9994105 | 1.004539 | 1.022378 | 1.001562 | 1.000933 | 1.078637 | 1.002827 | 1.083898 |
| Mozambique | 1.001185 | 1.020746 | 1.053493 | 1.001735 | 1.056853 | 0.9997333 | 1.003376 | 1.000701 | 1.019939 | 1.029279 | 1.005358 | 1.008425 | 0.9998725 | 1.002501 |
| Namibia | 1.013462 | 1.003056 | 1.01582 | 0.9992523 | 1.036278 | 1.058227 | 1.004843 | 1.003073 | 1.032595 | 1.01402 | 1.015211 | 1.002465 | 1.000385 | 1.03782 |
| Nepal | 1.018726 | 1.003537 | 0.9995167 | 1.026271 | 1.009225 | 1.050681 | 1.043987 | 1.014347 | 1.026888 | 1.035912 | 1.011976 | 1.017701 | 1.01196 | 1.010324 |
| Nicaragua | 1.006738 | 0.9993314 | 1.013908 | 1.028407 | 1.016681 | 1.002901 | 1.032415 | 1.012493 | 0.999633 | 1.000256 | 1.001847 | 1.028641 | 1.001309 | 1.000379 |
| Niger | 1.0199 | 1.027797 | 1.015846 | 1.043876 | 1.012065 | 1.06139 | 1.001023 | 1.001221 | 1.004779 | 1.009898 | 1.052253 | 1.009335 | 1.004041 | 1.020089 |
| Nigeria | 1.000567 | 1.010251 | 0.9996439 | 1.007759 | 1.004526 | 1.004758 | 1.014075 | 1.001101 | 0.9992439 | 1.005439 | 0.999349 | 0.9996578 | 1.000485 | 1.000553 |
| Pakistan | 1.00747 | 1.000855 | 1.000201 | 1.014547 | 0.9992437 | 1.00307 | 1.00374 | 1.037903 | 1.000662 | 1.002703 | 1.002682 | 1.007592 | 1.006036 | 1.000639 |
| Peru | 1.005155 | 1.010947 | 1.01085 | 1.007954 | 1.004432 | 1.000687 | 1.007102 | 1.030882 | 1.00044 | 1.000436 | 1.072237 | 1.007542 | 1.004213 | 1.004384 |
| Philippines | 1.007723 | 0.99942 | 1.000496 | 1.001395 | 1.004594 | 1.003026 | 0.9999111 | 1.000505 | 1.000194 | 1.003721 | 1.001973 | 1.000857 | 1.03317 | 1.011223 |
| Rwanda | 1.003458 | 0.9996353 | 1.045878 | 1.01921 | 0.9999812 | 1.000535 | 0.9999988 | 1.003198 | 1.007968 | 1.002754 | 1.00367 | 1.015054 | 1.032541 | 1.00453 |
| Senegal | 1.001045 | 1.004359 | 1.005488 | 1.016486 | 1.007066 | 1.010737 | 1.013529 | 1.000986 | 1.004764 | 1.001764 | 1.027707 | 1.002597 | 1.036 | 1.000183 |
| Sierra Leone | 1.081967 | 1.007723 | 0.999762 | 1.013336 | 1.003476 | 1.008214 | 1.086154 | 1.093727 | 1.000651 | 1.004283 | 1.075622 | 1.030132 | 1.020177 | 1.095157 |
| South Africa | 1.019836 | 1.000382 | 1.006986 | 1.00335 | 0.999349 | 1.016256 | 1.002128 | 1.003445 | 1.002685 | 1.004069 | 1.0024 | 0.9998588 | 1.002833 | 1.015698 |
| Tajikistan | 1.005593 | 1.030354 | 1.000728 | 1.002137 | 0.9994019 | 1.00929 | 1.004849 | 1.038978 | 1.016919 | 1.01158 | 1.012905 | 1.005354 | 1.001827 | 1.004727 |
| Tanzania | 1.006365 | 1.002368 | 1.009854 | 1.000652 | 1.030175 | 1.000071 | 1.005844 | 1.009178 | 1.012984 | 1.004946 | 1.000383 | 1.001749 | 1.024888 | 1.001442 |
| Timor-Leste | 1.001187 | 0.9997619 | 1.006266 | 1.00291 | 1.019891 | 1.027627 | 1.052519 | 1.016375 | 1.005098 | 1.000737 | 1.007343 | 1.005269 | 1.005869 | 1.004461 |
| Togo | 1.024184 | 1.012529 | 1.031518 | 1.000911 | 1.005363 | 1.084473 | 1.006561 | 1.016125 | 1.052783 | 1.033548 | 1.086643 | 1.001776 | 1.013281 | 1.003176 |
| Turkey | 1.012962 | 1.061903 | 0.9991936 | 1.023279 | 1.017412 | 1.000042 | 1.015272 | 1.006411 | 1.00054 | 1.009716 | 1.002507 | 1.002302 | 1.005805 | 1.006316 |
| Uganda | 1.000525 | 1.015226 | 1.002865 | 1.017879 | 1.026302 | 1.043498 | 1.004674 | 0.9992871 | 1.029198 | 1.001166 | 0.9991984 | 1.001382 | 1.010122 | 1.011007 |
| Vietnam | 1.001593 | 1.008426 | 1.01243 | 1.000726 | 1.00169 | 1.092734 | 1.005769 | 1.049722 | 0.999402 | 1.000096 | 1.090709 | 1.000522 | 1.001723 | 1.008367 |
| Yemen | 1.010924 | N/A | N/A | N/A | N/A | N/A | 1.001143 | 1.005861 | 1.005873 | 1.008697 | 1.000954 | 1.014465 | 1.004614 | 1.005969 |
| Zambia | 1.003048 | 1.000982 | 1.052297 | 1.010547 | 1.000338 | 1.006156 | 1.007724 | 1.002043 | 0.9994003 | 0.9992048 | 1.001978 | 1.005811 | 1.008654 | 1.008386 |
| Zimbabwe | 1.037161 | 1.004023 | 0.9994441 | 1.005584 | 1.003621 | 1.001392 | 0.9999118 | 1.008607 | 1.001864 | 0.999623 | 1.006535 | 1.00168 | 1.000508 | 0.9998454 |

**Note:** N/A denotes not application and refers that the indicator has no sufficient data to make projections

Table S8: Potential scale reduction factor (PSRF) to diagnose convergence after Markov Chain Monte Carlo simulation in Bayesian regression model for three doses of DPT immunization among children 12-23 months of age in low- and midle-income countries

| **Country** | **Overall** | **Poorest** | **Poorer** | **Middle** | **Richer** | **Richest** | **Urban** | **Rural** | **Below secondary** | **Secondary+** | **15-19 years** | **20-49 years** | **Male** | **Female** |
| --- | --- | --- | --- | --- | --- | --- | --- | --- | --- | --- | --- | --- | --- | --- |
| Armenia | 1.015053 | 1.00351 | 1.005399 | 1.017108 | 1.000858 | 0.9993337 | 1.019566 | 1.005265 | 1.027935 | 1.019425 | 1.030462 | 1.011133 | 1.003409 | 1.000993 |
| Bangladesh | 1.005432 | 1.000186 | 0.9996669 | 1.025742 | 1.001987 | 1.003507 | 1.001696 | 1.003079 | 1.009765 | 1.000978 | 1.0159 | 1.014775 | 1.005257 | 1.001418 |
| Benin | 1.002658 | 1.011021 | 1.001511 | 1.013336 | 1.001249 | 0.9995567 | 1.007437 | 1.009011 | 1.009565 | 0.9994683 | 0.9996784 | 1.009117 | 0.9998075 | 0.9996543 |
| Bolivia | 1.034141 | 1.032676 | 1.006392 | 1.085066 | 1.011483 | 1.030892 | 1.029487 | 1.019077 | 1.042308 | 1.043311 | 1.044267 | 1.053711 | 1.050787 | 1.003683 |
| Brazil | 1.007989 | N/A | N/A | N/A | N/A | N/A | 1.08477 | 1.026643 | 1.0148 | 1.000223 | 1.012905 | 1.010385 | 1.053072 | 1.006534 |
| Burkina Faso | 1.003676 | 1.018511 | 1.026103 | 1.021658 | 1.000276 | 1.018772 | 1.003275 | 1.041243 | 1.017325 | 1.028823 | 1.005651 | 1.015123 | 1.009522 | 1.00029 |
| Burundi | 1.045315 | 1.08339 | 1.017177 | 1.008905 | 1.00059 | 1.001454 | 1.002681 | 1.016197 | 1.047085 | 1.062497 | 0.9995555 | 1.039945 | 1.003542 | 1.037473 |
| Cambodia | 1.002635 | 1.025238 | 1.003284 | 1.001556 | 1.002009 | 1.018796 | 1.006917 | 1.084834 | 0.9998885 | 1.032406 | 1.037653 | 1.01441 | 0.9994232 | 1.007546 |
| Cameroon | 1.005159 | 0.9993818 | 1.002558 | 1.041688 | 1.028148 | 1.003888 | 1.031705 | 1.005978 | 1.00078 | 1.0019 | 1.004864 | 1.010622 | 1.001086 | 1.002225 |
| Chad | 1.010463 | 1.000027 | 1.004646 | 1.058 | 0.9995402 | 1.001173 | 1.011634 | 1.006716 | 1.011473 | 1.000406 | 1.006299 | 1.002722 | 1.013006 | 1.001081 |
| Colombia | 1.003568 | 1.001433 | 1.001198 | 1.008963 | 1.001145 | 1.000609 | 0.9999027 | 1.005261 | 1.001369 | 1.001386 | 0.9995939 | 0.9992378 | 1.005763 | 1.016613 |
| Comoros | 1.014969 | 1.030538 | 1.011049 | 1.028321 | 1.000404 | 1.000566 | 1.028725 | 1.010603 | 1.073404 | 1.000502 | 1.000082 | 1.002239 | 1.018057 | 1.020671 |
| Congo | 0.999929 | 0.9999365 | 1.005686 | 1.000017 | 1.003774 | 1.011591 | 1.016379 | 1.000022 | 0.9996008 | 1.002458 | 1.007055 | 1.000831 | 1.001397 | 1.001178 |
| Congo DR | 0.9999406 | 1.00388 | 1.017736 | 0.9994428 | 1.001279 | 1.002482 | 1.001755 | 1.02606 | 1.001742 | 0.9999095 | 1.005818 | 0.9996001 | 1.001292 | 1.021952 |
| Cote d'Ivoire | 1.002169 | 1.001016 | 1.00405 | 1.027651 | 1.006983 | 1.05009 | 1.0144 | 1.027967 | 1.000948 | 1.004608 | 1.001138 | 1.033345 | 1.005051 | 1.007367 |
| Dominican Republic | 1.000466 | 1.021703 | 1.006622 | 0.9996512 | 1.002694 | 1.001446 | 1.001367 | 1.007022 | 1.002793 | 1.002585 | 1.000475 | 1.000458 | 1.014937 | 1.008238 |
| Egypt | 1.048049 | 0.9999714 | 1.014558 | 1.001587 | 1.002658 | 1.003034 | 1.00458 | 1.002401 | 1.004697 | 1.000004 | 0.9996068 | 1.010315 | 1.003505 | 1.016141 |
| Ethiopia | 0.9993027 | 1.012998 | 0.9999198 | 1.019151 | 1.001559 | 1.001694 | 1.000999 | 1.021944 | 1.017545 | 1.027733 | 1.00024 | 1.004554 | 1.0137 | 1.023777 |
| Gabon | 1.009047 | 0.9992164 | 1.017407 | 1.00585 | 0.9998863 | 1.001878 | 1.044455 | 1.000715 | 1.000425 | 0.9996593 | 1.004568 | 1.015529 | 1.012159 | 1.017592 |
| Ghana | 1.008373 | 1.004107 | 1.002677 | 1.053384 | 1.00157 | 1.004213 | 0.9997556 | 1.001511 | 1.036421 | 1.0008 | 0.999241 | 1.019735 | 1.012071 | 1.044001 |
| Guatemala | 1.011604 | 0.999997 | 1.021299 | 0.9994857 | 1.050379 | 1.00543 | 1.006339 | 1.00402 | 1.002253 | 1.005502 | 1.008381 | 1.009081 | 1.001905 | 1.003721 |
| Guinea | 1.031871 | 1.011204 | 1.006884 | 1.001034 | 1.012393 | 1.00055 | 1.04046 | 1.00037 | 1.022139 | 1.018996 | 1.003496 | 1.023921 | 1.016227 | 1.000137 |
| Haiti | 1.001243 | 1.000849 | 0.9998857 | 1.000323 | 1.013626 | 1.015519 | 1.022187 | 1.008698 | 1.004481 | 1.002831 | 1.006771 | 1.004749 | 1.019752 | 1.001131 |
| Honduras | 1.060866 | 1.00607 | 1.039228 | 1.009681 | 1.004641 | 1.012317 | 1.036015 | 1.001033 | 1.013035 | 1.030257 | 1.04487 | 1.003556 | 1.008874 | 1.01571 |
| India | 1.003669 | 0.9997652 | 1.013913 | 1.000149 | 1.018863 | 1.009565 | 1.009724 | 1.002714 | 1.005754 | 1.03048 | 1.004962 | 1.011456 | 1.003069 | 1.014026 |
| Indonesia | 1.007305 | 1.001947 | 0.9995939 | 1.000742 | 1.002952 | 1.004329 | 1.008705 | 1.007082 | 1.006197 | 1.008417 | 1.008064 | 0.9995275 | 1.004338 | 1.00799 |
| Jordan | 1.005431 | 1.000998 | 0.9997482 | 1.007555 | 1.028102 | 1.000966 | 1.002351 | 1.001079 | 1.014832 | 0.9998109 | 1.024337 | 0.9995026 | 1.004853 | 1.006833 |
| Kazakhstan | 1.048114 | 1.003767 | 1.040077 | 1.009085 | 1.0747 | 0.9999961 | 1.001626 | 1.003549 | N/A | 1.008555 | 1.015737 | 1.05902 | 1.097439 | 1.007601 |
| Kenya | 1.000763 | 1.017028 | 1.001855 | 1.005951 | 1.016707 | 1.003066 | 1.016333 | 1.003685 | 1.001187 | 1.000022 | 1.029373 | 1.000976 | 1.002087 | 1.026288 |
| Kyrgyz Republic | 1.002245 | 1.002279 | 0.999193 | 1.021614 | 1.028867 | 1.015384 | 1.000321 | 0.9991817 | N/A | 1.00098 | 1.033136 | 1.015036 | 1.015877 | 1.035959 |
| Lesotho | 1.004651 | 0.9999426 | 1.023829 | 1.000782 | 1.000993 | 1.008576 | 1.001526 | 1.001127 | 1.000735 | 1.012188 | 1.002681 | 1.017248 | 1.041202 | 1.00009 |
| Liberia | 1.004963 | 0.9994003 | 1.041156 | 1.006789 | 1.001572 | 1.00052 | 1.002358 | 1.050494 | 0.99948 | 0.9996226 | 1.004666 | 1.025627 | 1.000846 | 1.011816 |
| Madagascar | 1.016654 | 0.9997138 | 1.007063 | 1.004136 | 1.022076 | 1.00896 | 1.00081 | 1.011381 | 0.9994944 | 1.019127 | 0.999653 | 0.9996777 | 1.006685 | 1.022304 |
| Malawi | 1.001992 | 1.00646 | 1.00239 | 0.9996623 | 1.005962 | 1.001216 | 1.018951 | 1.01003 | 1.00267 | 1.003926 | 1.005861 | 1.005213 | 1.008819 | 1.003996 |
| Maldives | 1.016608 | 1.001687 | 1.008428 | 1.003413 | 1.000442 | 1.029226 | 0.9991778 | 1.002588 | 1.001619 | 1.003398 | 1.045197 | 1.006286 | 1.001152 | 1.000762 |
| Mali | 1.011649 | 1.000951 | 0.9999645 | 1.044579 | 0.9991684 | 1.000619 | 1.003775 | 1.006804 | 1.002995 | 1.00978 | 1.001293 | 1.016658 | 1.016948 | 1.003531 |
| Morocco | 1.008462 | 1.019372 | 1.090296 | 1.007722 | 1.007698 | 1.070938 | 1.025205 | 1.090322 | 1.037666 | 0.9997587 | 1.003643 | 1.003294 | 1.009192 | 1.016536 |
| Mozambique | 1.001748 | 0.9992608 | 1.005444 | 0.9995953 | 1.008487 | 1.001047 | 0.999582 | 1.009419 | 1.005619 | 1.001623 | 1.008089 | 1.013279 | 1.009808 | 1.015235 |
| Namibia | 1.005121 | 1.00603 | 0.9992118 | 1.003657 | 1.010103 | 0.9993923 | 1.002485 | 1.009707 | 1.004158 | 1.003431 | 1.007786 | 1.005643 | 1.011697 | 1.072213 |
| Nepal | 0.999513 | 1.004999 | 1.012029 | 1.005383 | 1.034392 | 0.9992095 | 1.002844 | 1.009494 | 1.007225 | 1.027743 | 1.043991 | 1.000867 | 1.06367 | 1.028351 |
| Nicaragua | 1.013337 | 1.001581 | 1.007385 | 1.000837 | 1.00695 | 1.026468 | 0.99932 | 1.054056 | 1.002315 | 1.004379 | 1.00058 | 1.017455 | 1.025767 | 1.004703 |
| Niger | 1.006194 | 1.03214 | 1.071225 | 1.022524 | 1.012159 | 1.00023 | 1.003787 | 1.021194 | 1.016454 | 1.001702 | 1.007201 | 1.027171 | 1.021779 | 1.000018 |
| Nigeria | 1.004216 | 1.002319 | 1.00174 | 1.008574 | 1.007766 | 1.00047 | 0.9992816 | 1.010737 | 1.001678 | 1.000051 | 1.021917 | 1.006405 | 1.009715 | 1.005207 |
| Pakistan | 1.010062 | 1.002717 | 1.003271 | 1.008016 | 1.007393 | 1.00782 | 1.023068 | 1.006691 | 1.011531 | 1.026784 | 1.00045 | 1.005966 | 1.004748 | 1.003952 |
| Peru | 1.007698 | 1.001055 | 1.002739 | 1.002854 | 1.008734 | 1.009028 | 1.009203 | 1.002317 | 1.020752 | 1.000535 | 1.007595 | 1.000856 | 1.005243 | 1.004322 |
| Philippines | 1.007442 | 1.001832 | 1.010761 | 1.004498 | 1.006245 | 1.002975 | 1.009093 | 1.005678 | 0.9992369 | 1.004241 | 1.022063 | 1.003202 | 1.007221 | 1.024635 |
| Rwanda | 1.009793 | 1.019769 | 1.016956 | 1.021987 | 1.014007 | 1.000758 | 1.003176 | 1.001684 | 1.011979 | 1.003959 | 1.009563 | 1.0015 | 1.008473 | 1.02048 |
| Senegal | 1.004168 | 1.001162 | 1.02287 | 1.025721 | 1.000269 | 1.010135 | 0.9994795 | 0.9998103 | 1.008742 | 1.016763 | 1.027582 | 1.001156 | 1.000369 | 1.001372 |
| Sierra Leone | 1.004199 | 1.029801 | 1.01029 | 1.038912 | 1.010571 | 1.004321 | 1.000354 | 0.9993213 | 1.002299 | 1.000381 | 1.025055 | 1.022059 | 1.02533 | 1.000407 |
| South Africa | 1.003399 | 1.008694 | 0.999788 | 1.014392 | 1.001304 | 0.9998197 | 1.000515 | 1.006246 | 1.007116 | 1.001907 | 0.9992021 | 1.013242 | 1.007861 | 1.024468 |
| Tajikistan | 1.026351 | 0.9993113 | 1.012116 | 1.010235 | 1.007194 | 1.00451 | 0.9995387 | 1.02063 | 1.003071 | 1.000591 | 1.043929 | 1.006251 | 1.012441 | 1.00859 |
| Tanzania | 1.005863 | 1.000815 | 1.020282 | 1.015852 | 1.025361 | 0.9994535 | 1.001742 | 1.005813 | 1.006214 | 1.000143 | 0.9992909 | 1.010999 | 0.9996969 | 1.013314 |
| Timor-Leste | 1.023866 | 1.012968 | 0.9999567 | 1.003301 | 1.00292 | 1.003755 | 1.000289 | 1.000831 | 0.9997271 | 1.005796 | 1.001937 | 1.006635 | 1.000001 | 1.006448 |
| Togo | 1.001296 | 1.022446 | 1.001176 | 1.009252 | 1.035218 | 1.000658 | 1.008388 | 1.012959 | 1.018031 | 0.9997258 | 1.069771 | 1.023283 | 1.000535 | 1.00348 |
| Turkey | 1.016433 | 1.00307 | 0.9994077 | 1.003964 | 1.002647 | 1.042129 | 1.027626 | 1.003765 | 0.9993319 | 1.009948 | 1.015409 | 1.004429 | 0.999975 | 1.001777 |
| Uganda | 0.9993745 | 1.00636 | 1.001248 | 1.000061 | 1.003178 | 1.002222 | 1.01168 | 1.000788 | 0.9991759 | 1.028006 | 1.010215 | 1.007168 | 1.008228 | 1.002697 |
| Vietnam | 0.999495 | 0.9996192 | 1.000135 | 1.002119 | 1.025409 | 1.025964 | 1.039974 | 1.001299 | 1.004574 | 1.007408 | 1.005686 | 1.017933 | 1.003127 | 0.9996085 |
| Yemen | 1.03374 | N/A | N/A | N/A | N/A | N/A | 1.009739 | 1.029412 | 1.010253 | 0.9999297 | 0.9992728 | 1.000845 | 1.005903 | 1.035682 |
| Zambia | 1.007547 | 0.9997746 | 1.006439 | 0.9993712 | 1.020333 | 1.006341 | 1.004721 | 1.001993 | 1.007318 | 1.00189 | 1.007522 | 1.004498 | 1.00875 | 1.008714 |
| Zimbabwe | 1.004714 | 1.001151 | 1.020771 | 0.9997609 | 1.019333 | 1.017312 | 1.030602 | 0.9991689 | 1.00066 | 1.00717 | 1.000688 | 1.003513 | 1.032538 | 1.000584 |

**Note:** N/A denotes not application and refers that the indicator has no sufficient data to make projections

Table S9: Potential scale reduction factor (PSRF) to diagnose convergence after Markov Chain Monte Carlo simulation in Bayesian regression model for Measles immunization among children 12-23 months of age in low- and midle-income countries

| **Country** | **Overall** | **Poorest** | **Poorer** | **Middle** | **Richer** | **Richest** | **Urban** | **Rural** | **Below secondary** | **Secondary+** | **15-19 years** | **20-49 years** | **Male** | **Female** |
| --- | --- | --- | --- | --- | --- | --- | --- | --- | --- | --- | --- | --- | --- | --- |
| Albania | 1.002986 | 1.029118 | 1.028866 | 1.009136 | 1.085571 | 1.020816 | 1.053393 | 1.015653 | 1.002943 | 1.038991 | 1.060421 | 1.015165 | 1.011597 | 1.026233 |
| Armenia | 1.01244 | 1.01777 | 1.018884 | 1.017169 | 1.003743 | 1.012086 | 1.02246 | 1.040097 | 1.001063 | 1.006072 | 1.010263 | 0.9995728 | 1.044065 | 1.024628 |
| Bangladesh | 1.008403 | 1.002386 | 0.999382 | 1.005711 | 1.040416 | 1.033116 | 1.002194 | 1.033663 | 1.004173 | 1.000588 | 1.002317 | 1.014901 | 1.007711 | 1.016137 |
| Benin | 1.002786 | 1.000477 | 0.9997731 | 1.002312 | 1.000509 | 1.00091 | 1.004098 | 0.9993576 | 1.00058 | 0.999673 | 1.000379 | 1.006425 | 1.001584 | 0.9997056 |
| Bolivia | 1.000955 | 1.010163 | 1.004549 | 1.003051 | 1.002445 | 1.003551 | 1.003127 | 1.071727 | 0.9999663 | 0.9997371 | 1.002553 | 1.019761 | 1.00479 | 1.010638 |
| Brazil | 1.056127 | N/A | N/A | N/A | N/A | N/A | 1.014447 | 1.000896 | 1.009656 | 1.004262 | 1.000456 | 1.035484 | 1.026541 | 0.9997076 |
| Burkina Faso | 1.030288 | 1.016018 | 1.002116 | 1.046961 | 0.9992703 | 1.011423 | 1.007872 | 1.003746 | 1.00291 | 1.000923 | 1.012376 | 1.001533 | 1.000232 | 1.008392 |
| Burundi | 1.000726 | 1.005891 | 0.9991703 | 1.001739 | 1.005689 | 1.007926 | 1.01255 | 1.00404 | 1.002973 | 1.007847 | 1.008057 | 1.008452 | 1.002998 | 1.028879 |
| Cambodia | 0.9994656 | 1.075456 | 0.9997771 | 1.001761 | 0.9994898 | 0.9992313 | 1.040466 | 1.012073 | 1.000767 | 1.010577 | 1.076145 | 1.017935 | 1.001915 | 1.021397 |
| Cameroon | 1.018284 | 0.9992334 | 1.000108 | 1.008221 | 1.002112 | 1.001712 | 1.001129 | 1.027034 | 1.000421 | 1.000244 | 0.9992762 | 0.9993958 | 1.001343 | 1.023942 |
| Chad | 1.014982 | 0.9998481 | 1.002821 | 1.000792 | 1.02633 | 1.019181 | 1.030961 | 1.003223 | 1.045482 | 1.00548 | 1.037967 | 1.014492 | 1.008403 | 1.010286 |
| Colombia | 1.003094 | 0.9999236 | 0.9995056 | 1.002964 | 0.9996178 | 1.010116 | 1.003529 | 1.000387 | 1.00094 | 1.011207 | 1.003872 | 1.026355 | 1.030421 | 0.999408 |
| Comoros | 1.007026 | 1.003438 | 1.003368 | 1.007766 | 0.9992333 | 1.020174 | 1.045526 | 1.002426 | 1.019625 | 1.013353 | 0.9994149 | 1.003434 | 1.000008 | 1.01217 |
| Congo | 1.006691 | 1.017148 | 0.9994123 | 1.025962 | 1.010023 | 1.007989 | 1.004791 | 1.007898 | 1.000248 | 1.00733 | 1.000532 | 1.000327 | 1.005112 | 1.001294 |
| Congo DR | 0.9993559 | 1.00119 | 1.000379 | 1.005963 | 1.007554 | 1.001682 | 1.000898 | 1.002736 | 1.008814 | 1.001265 | 1.015726 | 1.001895 | 1.004975 | 1.026453 |
| Cote d'Ivoire | 1.000224 | 1.013738 | 1.002492 | 1.006326 | 1.006731 | 1.015812 | 1.006568 | 1.003098 | 1.0006 | 1.000652 | 1.00551 | 1.021482 | 1.000632 | 1.006296 |
| Dominican Republic | 1.006881 | 1.027564 | 1.007046 | 1.001099 | 1.004122 | 1.001956 | 0.9997751 | 1.006445 | 1.009095 | 1.006263 | 1.002595 | 1.005609 | 0.9996478 | 0.9995611 |
| Egypt | 1.000528 | 1.017227 | 1.00301 | 0.9996338 | 1.00309 | 0.9993304 | 1.002236 | 1.001432 | 1.011347 | 1.003272 | 1.0055 | 1.003041 | 1.010438 | 1.005026 |
| Ethiopia | 1.007392 | 1.004849 | 1.007904 | 1.000973 | 1.010358 | 1.017495 | 0.9997727 | 1.00482 | 1.012213 | 1.000154 | 1.045624 | 1.000962 | 1.012109 | 1.004368 |
| Gabon | 0.9996899 | 1.001447 | 1.002583 | 1.007285 | 1.000922 | 1.048426 | 1.008644 | 1.033353 | 1.009892 | 1.043845 | 1.021909 | 1.001557 | 1.029134 | 0.9992608 |
| Ghana | 1.000372 | 1.009636 | 1.029837 | 1.004797 | 0.9994108 | 1.040272 | 1.003162 | 0.9993855 | 1.029258 | 1.037737 | 0.9996197 | 1.00618 | 1.001181 | 0.9991842 |
| Guatemala | 0.9993855 | 1.00572 | 1.013093 | 0.9996182 | 1.007642 | 1.022912 | 1.002082 | 1.018377 | 1.004446 | 1.011675 | 1.002922 | 1.002273 | 1.032426 | 0.9995227 |
| Guinea | 1.001245 | 1.008463 | 1.008048 | 1.000256 | 1.002499 | 1.001975 | 1.008548 | 1.006262 | 1.006494 | 1.006856 | 1.000299 | 1.001258 | 1.013154 | 1.002674 |
| Haiti | 1.000154 | 1.001899 | 1.00513 | 1.033279 | 0.9996899 | 1.014906 | 1.022399 | 1.013826 | 1.024918 | 1.008348 | 1.012905 | 0.9996943 | 1.002997 | 1.008036 |
| Honduras | 1.001286 | 1.026788 | 0.9998031 | 1.007942 | 1.000375 | 1.004116 | 1.001524 | 0.9992556 | 0.9996465 | 1.005302 | 1.001236 | 1.003915 | 0.999293 | 1.032115 |
| India | 1.004208 | 1.004018 | 1.006616 | 1.018975 | 1.004713 | 1.00148 | 1.000925 | 1.004125 | 1.010013 | 1.007249 | 0.9991937 | 1.00084 | 1.001346 | 1.006991 |
| Indonesia | 1.004689 | 1.006204 | 1.00166 | 1.008365 | 1.003937 | 1.04122 | 1.033557 | 1.023394 | 1.002282 | 1.022821 | 1.000969 | 1.018168 | 0.9998583 | 1.000343 |
| Jordan | 1.000494 | 0.9993977 | 1.000319 | 1.000282 | 0.9995751 | 1.017395 | 0.9998475 | 1.003044 | 1.009913 | 1.006983 | 1.001021 | 0.9992354 | 1.004815 | 1.079417 |
| Kazakhstan | 1.030124 | 1.009478 | 1.032703 | 1.000868 | 1.032476 | 1.00257 | 0.9999347 | 1.019984 | N/A | 0.9995663 | 1.08495 | 1.049619 | 1.017217 | 0.9992989 |
| Kenya | 0.9997787 | 1.035506 | 0.9997687 | 1.008571 | 1.004613 | 1.002026 | 1.002444 | 1.000567 | 1.009274 | 1.010617 | 1.008203 | 1.008124 | 1.009453 | 1.001732 |
| Kyrgyz Republic | 1.000038 | 1.014572 | 1.000782 | 1.019146 | 0.9994879 | 1.003266 | 1.011496 | 1.000079 | 1.005381 | 1.004692 | 1.002819 | 0.9996673 | 1.01754 | 0.9995797 |
| Lesotho | 1.005544 | 1.007345 | 1.011279 | 1.003808 | 1.001326 | 1.007973 | 1.003636 | 1.000682 | 1.001276 | 1.018659 | 1.001896 | 1.002239 | 1.005618 | 1.003668 |
| Liberia | 1.006197 | 1.010813 | 1.005451 | 1.017717 | 1.007835 | 1.007182 | 1.000716 | 1.006379 | 1.007791 | 1.007936 | 1.002589 | 1.02483 | 0.9992611 | 1.004527 |
| Madagascar | 1.001757 | 1.000217 | 1.033893 | 1.003544 | 1.012617 | 1.029483 | 1.003641 | 1.032459 | 1.000837 | 1.004962 | 1.003466 | 1.002103 | 1.000142 | 1.011471 |
| Malawi | 1.000004 | 1.001891 | 1.006124 | 1.016141 | 1.002156 | 1.001341 | 1.002382 | 1.018046 | 1.007494 | 1.001696 | 1.00284 | 1.021275 | 1.025419 | 1.007141 |
| Maldives | 1.00048 | 1.00864 | 1.0014 | 1.018497 | 1.000417 | 1.028355 | 1.022575 | 1.004567 | 0.9995158 | 1.011041 | 1.022366 | 1.001848 | 1.00373 | 1.0005 |
| Mali | 1.027341 | 1.004058 | 1.010962 | 1.013515 | 1.03737 | 1.029274 | 1.010255 | 1.009722 | 1.016033 | 1.084239 | 1.006518 | 1.000089 | 1.004808 | 1.001644 |
| Morocco | 1.034961 | 1.013301 | 1.002308 | 1.022194 | 1.029168 | 1.079144 | 1.008617 | 1.087412 | 1.035056 | 1.000755 | 1.02906 | 1.008081 | 1.008274 | 1.006649 |
| Mozambique | 1.001345 | 1.003509 | 1.015949 | 1.014731 | 0.9996433 | 1.019113 | 1.029701 | 1.036277 | 1.015355 | 1.001054 | 1.005858 | 1.007405 | 1.004534 | 1.011407 |
| Namibia | 1.006893 | 1.002692 | 1.013255 | 0.9995082 | 0.9998504 | 1.033491 | 1.022308 | 1.007037 | 1.003023 | 1.068374 | 1.002559 | 1.003347 | 1.008633 | 1.003432 |
| Nepal | 1.027987 | 1.036198 | 1.003132 | 1.015431 | 1.001647 | 1.062977 | 1.020759 | 1.002688 | 1.015411 | 1.015885 | 1.009459 | 1.037294 | 1.001238 | 1.008884 |
| Nicaragua | 0.999468 | 1.001026 | 1.021267 | 1.004579 | 1.022285 | 1.080139 | 1.010467 | 0.9995226 | 1.011441 | 1.004935 | 1.003699 | 1.00323 | 1.019598 | 1.06075 |
| Niger | 1.000383 | 0.9993035 | 1.005171 | 1.005012 | 1.011109 | 1.00147 | 0.9998284 | 1.012166 | 1.010631 | 1.011559 | 1.037172 | 1.005635 | 1.013196 | 1.008437 |
| Nigeria | 1.018823 | 1.008541 | 1.001063 | 0.9996398 | 0.9995152 | 1.002666 | 1.001181 | 1.016647 | 0.9992491 | 1.012509 | 1.028287 | 0.9998294 | 1.002003 | 1.015492 |
| Pakistan | 1.001294 | 1.017559 | 1.010893 | 1.001002 | 1.001114 | 1.003179 | 1.004679 | 1.004719 | 1.005771 | 0.9994186 | 1.010599 | 1.013475 | 1.013586 | 1.004277 |
| Peru | 1.02005 | 1.003193 | 1.004688 | 1.003378 | 1.0298 | 1.028453 | 0.9995158 | 1.021705 | 1.01473 | 1.001605 | 1.001403 | 0.9998208 | 1.014615 | 1.001806 |
| Philippines | 1.010757 | 1.005006 | 1.005982 | 1.004901 | 1.004398 | 0.9997036 | 1.002102 | 1.013705 | 1.004492 | 0.9995767 | 1.008315 | 1.00091 | 1.019922 | 1.000124 |
| Rwanda | 0.9992833 | 1.028474 | 1.000764 | 0.9997516 | 1.010191 | 1.00213 | 1.015247 | 1.002281 | 1.003196 | 0.9997054 | 0.9992291 | 1.009255 | 1.010649 | 1.005023 |
| Senegal | 1.001115 | 1.001878 | 1.007667 | 0.9999006 | 1.000488 | 1.066652 | 1.026546 | 1.003156 | 1.002098 | 1.002184 | 1.004283 | 1.002606 | 1.003916 | 1.000715 |
| Sierra Leone | 1.036317 | 1.062863 | 1.004256 | 1.025573 | 1.009401 | 1.032859 | 1.006839 | 1.036017 | 1.032216 | 1.002958 | 1.058934 | 1.091494 | 1.069029 | 1.010133 |
| South Africa | 1.004486 | 1.027022 | 1.009311 | 1.004787 | 1.005713 | 1.000321 | 1.002852 | 1.012808 | 1.016352 | 1.01102 | 1.001923 | 1.00595 | 0.9993028 | 1.006947 |
| Tajikistan | 0.9998549 | 0.9991819 | 1.003996 | 1.000111 | 1.002719 | 1.006857 | 1.001305 | 1.012228 | 1.001271 | 1.009272 | 1.004908 | 1.000267 | 0.999688 | 1.002269 |
| Tanzania | 1.016544 | 1.011075 | 1.008038 | 1.005042 | 1.049963 | 1.032147 | 1.020493 | 1.003718 | 1.002477 | 1.018116 | 1.002003 | 0.9995737 | 1.007356 | 1.029503 |
| Timor-Leste | 1.012966 | 1.002825 | 1.000228 | 1.00806 | 1.011683 | 1.00345 | 1.001818 | 1.006009 | 1.038381 | 0.9993813 | 1.002493 | 1.001419 | 1.002999 | 1.013145 |
| Togo | 1.028944 | 1.026371 | 1.00352 | 1.002213 | 0.9991831 | 1.005762 | 1.012665 | 1.011721 | 1.000196 | 1.000528 | 1.040094 | 1.007371 | 1.000551 | 1.007532 |
| Turkey | 1.007407 | 1.007091 | 1.020621 | 1.000945 | 1.000338 | 1.01524 | 1.007944 | 1.002025 | 1.005605 | 1.002082 | 1.002227 | 1.005232 | 1.040852 | 1.005795 |
| Uganda | 1.050437 | 1.028801 | 1.002215 | 1.000715 | 1.00131 | 1.04449 | 1.027341 | 0.999384 | 1.020453 | 1.013242 | 1.020713 | 1.0097 | 1.00029 | 1.004556 |
| Vietnam | 1.011224 | 1.002431 | 0.9999202 | 1.013403 | 0.9997423 | 1.007483 | 1.001859 | 0.9992074 | 1.006753 | 1.004675 | 1.004016 | 1.006208 | 1.000813 | 1.01068 |
| Yemen | 1.000566 | N/A | N/A | N/A | N/A | N/A | 0.999641 | 1.017685 | 1.005442 | 1.003838 | 1.000498 | 1.002156 | 1.003342 | 1.01618 |
| Zambia | 1.001501 | 1.006337 | 0.9999847 | 1.006676 | 1.001689 | 1.007253 | 1.000095 | 1.003846 | 1.027635 | 1.002757 | 1.008498 | 0.9992522 | 1.00795 | 1.005133 |
| Zimbabwe | 1.003881 | 1.000558 | 1.006723 | 0.9991898 | 1.015518 | 1.005183 | 0.9993367 | 0.9998871 | 0.9999453 | 0.9992213 | 1.005396 | 1.012167 | 1.003321 | 1.005152 |

**Note:** N/A denotes not application and refers that the indicator has no sufficient data to make projections

Table S10: Potential scale reduction factor (PSRF) to diagnose convergence after Markov Chain Monte Carlo simulation in Bayesian regression model for oral rehydration therapy among children 0-59 months of age in low- and midle-income countries

| **Country** | **Overall** | **Poorest** | **Poorer** | **Middle** | **Richer** | **Richest** | **Urban** | **Rural** | **Below secondary** | **Secondary+** | **15-19 years** | **20-49 years** | **Male** | **Female** |
| --- | --- | --- | --- | --- | --- | --- | --- | --- | --- | --- | --- | --- | --- | --- |
| Albania | 1.005368 | 1.007195 | 0.9997125 | 1.01641 | 1.006287 | 1.02169 | 1.002055 | 0.9993088 | 1.001916 | 1.023256 | 1.016648 | 0.9993529 | 1.00939 | 1.006238 |
| Armenia | 1.014403 | 1.017853 | 0.9992353 | 1.004084 | 0.9999912 | 1.000283 | 1.007375 | 1.012302 | 1.003567 | 1.010518 | 1.025388 | 1.00813 | 1.008582 | 1.001155 |
| Bangladesh | 1.001632 | 1.015964 | 1.003875 | 1.00765 | 1.005414 | 1.005897 | 1.013338 | 1.001362 | 1.010337 | 1.011308 | 1.020939 | 1.018095 | 1.013471 | 1.000772 |
| Benin | 1.016213 | 1.024301 | 1.029741 | 1.023204 | 1.008491 | 1.003579 | 0.9996316 | 1.001474 | 0.9995532 | 1.003135 | 1.039952 | 1.000341 | 1.022624 | 1.014609 |
| Bolivia | 1.013303 | 1.014695 | 1.007517 | 1.001112 | 0.999405 | 1.012975 | 1.001755 | 1.000479 | 1.019577 | 1.000011 | 1.013509 | 1.000181 | 1.01693 | 1.032647 |
| Brazil | 1.000701 | N/A | N/A | N/A | N/A | N/A | 0.9995853 | 1.005842 | 1.009717 | 1.005798 | 1.000337 | 1.00485 | 1.012072 | 1.001352 |
| Burkina Faso | 0.9992271 | 1.002497 | 1.007954 | 1.048916 | 1.004519 | 1.018771 | 1.00735 | 1.021712 | 1.001231 | 1.004179 | 0.9996793 | 1.000186 | 1.005656 | 1.003999 |
| Burundi | 1.001586 | 1.040519 | 1.00147 | 0.9993511 | 1.002001 | 1.004734 | 1.028367 | 0.9994379 | 1.003504 | 1.004118 | 1.02181 | 0.9999554 | 1.042501 | 1.003907 |
| Cambodia | 1.000772 | 1.001253 | 1.009345 | 1.000129 | 1.001516 | 1.006716 | 0.9999851 | 1.016771 | 1.001212 | 1.00093 | 1.004862 | 1.007278 | 1.002751 | 1.008032 |
| Cameroon | 1.008942 | 0.9994404 | 0.9996974 | 1.006291 | 1.00236 | 0.9994321 | 1.003937 | 1.00739 | 1.004811 | 1.000864 | 1.002827 | 1.001107 | 1.014678 | 1.003778 |
| Chad | 1.002256 | 1.012401 | 0.9998522 | 1.003801 | 1.004071 | 1.001368 | 1.000107 | 0.9997146 | 1.006041 | 1.0037 | 0.9992554 | 1.010853 | 1.007834 | 1.000307 |
| Colombia | 1.020223 | 1.000938 | 1.015881 | 1.017015 | 1.001437 | 1.000224 | 1.000242 | 1.006156 | 1.00059 | 1.000124 | 1.00265 | 1.006235 | 0.9999129 | 0.9999669 |
| Comoros | 1.025561 | 1.00453 | 0.9994842 | 1.01159 | 1.00304 | 0.9997796 | 1.026497 | 0.9993277 | 0.9994486 | 1.004405 | 1.000263 | 1.006338 | 1.001268 | 1.003674 |
| Congo | 1.002503 | 1.000084 | 1.000466 | 1.007402 | 0.9997485 | 0.9999098 | 1.040519 | 1.00724 | 1.003728 | 1.00101 | 1.00566 | 0.9999055 | 0.9994256 | 1.004615 |
| Congo DR | 1.000863 | 1.000685 | 1.004917 | 1.011361 | 1.030039 | 1.002334 | 0.9998652 | 1.000584 | 1.004961 | 1.01456 | 1.005086 | 1.013133 | 1.017067 | 1.002534 |
| Cote d'Ivoire | 1.001233 | 1.004436 | 1.010163 | 0.9999489 | 1.002181 | 1.001853 | 1.001654 | 1.013964 | 1.014132 | 1.006596 | 1.064487 | 1.008416 | 1.001047 | 1.008034 |
| Dominican Republic | 1.00366 | 1.000418 | 1.005312 | 1.058883 | 1.016449 | 1.009381 | 0.9994892 | 1.00555 | 1.000628 | 1.00329 | 1.001242 | 1.003613 | 1.007402 | 1.000524 |
| Egypt | 0.9999819 | 1.004081 | 1.005214 | 1.007813 | 1.000555 | 1.015189 | 0.9992887 | 1.002061 | 1.001915 | 1.002244 | 1.001528 | 1.000297 | 1.012818 | 1.010999 |
| Ethiopia | 1.001037 | 1.00339 | 1.004005 | 1.010373 | 1.002674 | 1.003853 | 1.003452 | 0.9996253 | 1.018288 | 1.00224 | 1.000653 | 1.004863 | 1.002121 | 0.9999693 |
| Gabon | 1.006467 | 1.010101 | 1.000105 | 1.00474 | 1.00683 | 0.9995269 | 1.013999 | 1.021101 | 1.000883 | 1.003717 | 1.000177 | 1.003386 | 1.004191 | 1.004778 |
| Ghana | 0.9994969 | 1.004226 | 1.004961 | 1.002221 | 1.007088 | 1.02979 | 1.001357 | 1.000354 | 0.9992802 | 1.007138 | 1.020826 | 1.007064 | 1.00256 | 1.000841 |
| Guatemala | 1.022232 | 1.001226 | 1.001917 | 1.000796 | 1.005183 | 1.012582 | 0.9991851 | 1.009729 | 0.9992879 | 1.028331 | 1.004313 | 1.018364 | 1.003578 | 1.001606 |
| Guinea | 1.022555 | 1.002447 | 1.001123 | 1.001834 | 0.9997253 | 1.002808 | 1.003455 | 1.024403 | 1.009107 | 1.002081 | 1.008495 | 1.017366 | 1.002758 | 1.002276 |
| Haiti | 1.006129 | 1.026401 | 1.009416 | 1.002079 | 1.000256 | 1.000245 | 0.9998161 | 1.002599 | 1.001031 | 1.005194 | 1.003789 | 0.9995809 | 1.017363 | 0.999462 |
| Honduras | 1.005686 | 1.000366 | 1.00698 | 1.012159 | 1.000707 | 1.010939 | 1.007037 | 1.027855 | 0.9992275 | 1.005183 | 1.000929 | 1.006752 | 1.007373 | 1.003431 |
| India | 1.007131 | 1.000815 | 1.004979 | 1.000587 | 1.016582 | 1.006953 | 1.002806 | 1.000312 | 1.000129 | 1.009119 | 1.014818 | 1.001309 | 1.001543 | 0.9992769 |
| Indonesia | 1.008717 | 1.015278 | 1.000263 | 1.000213 | 1.002811 | 0.9997401 | 1.018574 | 0.9993601 | 1.005589 | 1.008625 | 1.004945 | 1.000402 | 1.008868 | 1.00907 |
| Jordan | 1.007532 | 1.012169 | 0.9999408 | 1.009264 | 1.002539 | 1.001154 | 1.018425 | 1.00269 | 1.018972 | 1.002205 | 1.01027 | 0.9992686 | 1.016032 | 1.0006 |
| Kazakhstan | 1.011952 | 1.048416 | 1.015031 | 0.99924 | 1.024864 | 1.001864 | 1.015448 | 1.003012 | N/A | 1.000084 | 1.001386 | 1.003089 | 1.008482 | 1.007652 |
| Kenya | 0.9997595 | 1.001914 | 1.002648 | 1.002416 | 1.006954 | 1.00037 | 1.001126 | 1.006687 | 1.001045 | 1.028481 | 1.011357 | 1.034783 | 1.043996 | 1.003377 |
| Kyrgyz Republic | 1.003866 | 1.001557 | 0.9998829 | 1.018848 | 1.008352 | 1.00444 | 1.006075 | 1.000703 | N/A | 1.00389 | 1.011772 | 1.045528 | 1.004701 | 1.001779 |
| Lesotho | 1.003251 | 1.016342 | 1.006502 | 1.016851 | 1.0001 | 1.001375 | 1.007545 | 1.032274 | 1.000103 | 1.000305 | 1.001161 | 1.004496 | 0.9998516 | 1.001303 |
| Liberia | 1.01324 | 1.007313 | 1.00687 | 0.9998009 | 1.001846 | 1.029305 | 1.0018 | 1.039741 | 0.9991928 | 1.008192 | 1.073575 | 1.00347 | 0.9995822 | 1.024678 |
| Madagascar | 1.021449 | 1.003354 | 1.000178 | 1.001595 | 1.000617 | 1.022965 | 1.000592 | 1.007341 | 0.9991801 | 0.9991707 | 1.000615 | 1.003745 | 0.9996274 | 0.9995634 |
| Malawi | 1.011242 | 1.00533 | 1.006357 | 1.005417 | 1.061969 | 0.9996847 | 1.000252 | 1.010635 | 1.006135 | 0.9998944 | 1.001911 | 0.9998805 | 1.033676 | 1.005558 |
| Maldives | 1.003972 | 1.007478 | 1.012432 | 1.017097 | 1.010478 | 1.012404 | 1.007581 | 1.020844 | 1.050751 | 1.021982 | 1.001911 | 1.01568 | 1.051266 | 1.08242 |
| Mali | 1.016549 | 1.005806 | 0.999265 | 1.006038 | 1.002326 | 1.010169 | 1.003176 | 1.006887 | 0.999188 | 1.000823 | 0.9991761 | 1.017351 | 1.001474 | 1.001177 |
| Morocco | 1.024187 | 1.003199 | 1.003182 | 1.011911 | 1.00198 | 1.012371 | 1.017698 | 1.011758 | 1.00482 | 1.000807 | 1.089674 | 1.00004 | 0.999523 | 1.000485 |
| Mozambique | 1.000777 | 1.006821 | 1.00666 | 1.003047 | 1.003601 | 1.005432 | 1.001617 | 1.042312 | 1.001681 | 1.000288 | 1.017818 | 1.012075 | 1.000988 | 1.006418 |
| Namibia | 1.000951 | 1.012466 | 1.007943 | 1.011384 | 1.011463 | 1.014902 | 1.02358 | 1.001369 | 1.002083 | 1.008993 | 1.010236 | 0.9992539 | 1.00111 | 1.001577 |
| Nepal | 1.015823 | 0.9993652 | 1.000533 | 1.014655 | 1.015282 | 1.007359 | 1.010476 | 1.006423 | 1.003696 | 1.002549 | 0.9992372 | 1.002373 | 0.9993072 | 1.0168 |
| Nicaragua | 1.000086 | 1.00014 | 0.9997572 | 0.9991974 | 1.004752 | 1.007679 | 0.9996382 | 1.006574 | 1.000955 | 1.012166 | 1.002557 | 0.9995676 | 1.006427 | 1.016459 |
| Niger | 1.001413 | 0.9998589 | 1.00137 | 1.006066 | 1.00151 | 1.002947 | 1.00988 | 0.9993281 | 1.030946 | 1.001808 | 1.006857 | 0.9993945 | 1.026858 | 0.9999716 |
| Nigeria | 1.006491 | 1.007634 | 1.00162 | 0.9995819 | 1.000124 | 1.029118 | 1.022371 | 1.005446 | 1.004023 | 1.002316 | 0.9997181 | 1.00567 | 1.001548 | 1.001784 |
| Pakistan | 1.019752 | 1.007337 | 1.003719 | 1.033489 | 1.001637 | 1.001723 | 1.007965 | 1.000278 | 1.004936 | 1.006385 | 1.004862 | 0.9995617 | 1.001009 | 1.022514 |
| Peru | 1.003537 | 1.009737 | 1.00384 | 0.9998443 | 1.004522 | 1.034528 | 1.001244 | 1.002895 | 1.000521 | 1.004105 | 0.9999433 | 1.00101 | 1.00706 | 0.9992101 |
| Philippines | 1.001346 | 1.02082 | 1.022071 | 0.9997696 | 1.014376 | 1.010259 | 1.016623 | 1.003352 | 1.000809 | 1.004172 | 1.003116 | 1.001126 | 1.002459 | 0.9996173 |
| Rwanda | 1.023414 | 1.006354 | 1.00245 | 1.003647 | 1.006405 | 1.001521 | 1.000466 | 1.000029 | 1.000243 | 1.000182 | 1.002644 | 1.002174 | 1.015009 | 1.003082 |
| Senegal | 0.9992143 | 1.003602 | 1.000973 | 1.007976 | 1.002295 | 1.003221 | 0.9995947 | 1.001073 | 1.001915 | 1.001073 | 0.9995158 | 1.00663 | 1.002193 | 1.003288 |
| Sierra Leone | 1.005051 | 1.007181 | 1.025473 | 1.010299 | 1.010948 | 1.009889 | 1.032092 | 1.056498 | 1.003454 | 1.010853 | 1.055791 | 0.9998986 | 1.019683 | 1.000086 |
| South Africa | 1.003629 | 1.0014 | 1.000628 | 1.003263 | 1.026712 | 1.011652 | 1.013589 | 1.003075 | 1.015387 | 1.004508 | 1.001857 | 1.00562 | 1.030205 | 0.9992223 |
| Tajikistan | 1.000346 | 1.014464 | 1.001221 | 0.9992768 | 1.027247 | 1.006304 | 1.006078 | 1.001255 | 1.005963 | 1.015043 | 1.001743 | 1.002694 | 0.9992845 | 1.02006 |
| Tanzania | 0.9995587 | 1.023228 | 1.018081 | 1.008128 | 1.010455 | 0.9998088 | 0.9999866 | 1.002796 | 1.015381 | 1.009872 | 1.02206 | 1.010614 | 0.9992654 | 1.001008 |
| Timor-Leste | 1.007998 | 1.006338 | 1.012455 | 0.9998759 | 1.00033 | 1.009614 | 1.003878 | 0.9998214 | 1.044653 | 1.001919 | 1.011413 | 1.010053 | 1.000825 | 1.010004 |
| Togo | 1.000247 | 0.9997928 | 1.00158 | 0.9992258 | 1.00744 | 1.00531 | 1.024308 | 0.999888 | 0.999224 | 1.021506 | 1.001176 | 1.016946 | 1.009184 | 1.002753 |
| Turkey | 1.023786 | 0.9991804 | 1.010757 | 1.011057 | 0.9997427 | 1.008945 | 0.9993129 | 1.003816 | 1.002866 | 1.001299 | 1.007635 | 1.003323 | 1.011216 | 1.000066 |
| Uganda | 1.015237 | 1.016232 | 1.00309 | 1.003428 | 1.002664 | 1.000087 | 0.9999959 | 1.003842 | 0.9994018 | 1.00005 | 0.9994051 | 1.001727 | 1.001506 | 0.9995916 |
| Vietnam | 1.010578 | 1.007697 | 1.061142 | 1.039828 | 1.001989 | 1.000754 | 1.012092 | 1.006951 | 1.000039 | 1.013746 | 0.9994051 | 1.005484 | 0.9998075 | 1.005943 |
| Yemen | 1.006221 | N/A | N/A | N/A | N/A | N/A | 1.020183 | 1.002386 | 1.008295 | 1.021276 | 1.014243 | 1.001275 | 1.009406 | 1.003352 |
| Zambia | 1.014885 | 1.001948 | 1.023007 | 1.004482 | 1.015894 | 1.002617 | 1.000149 | 1.001951 | 0.9993799 | 1.007631 | 1.004006 | 1.003999 | 0.9995014 | 1.000484 |
| Zimbabwe | 1.002785 | 1.010176 | 0.9993253 | 1.020728 | 1.002583 | 1.002061 | 1.002255 | 0.9994735 | 1.034039 | 1.001828 | 1.001155 | 1.035524 | 1.000828 | 1.011191 |

**Note:** N/A denotes not application and refers that the indicator has no sufficient data to make projections

Table S11: Potential scale reduction factor (PSRF) to diagnose convergence after Markov Chain Monte Carlo simulation in Bayesian regression model for care seeking for acute respiratory infections among children 0-59 months of age in low- and midle-income countries

| **Country** | **Overall** | **Poorest** | **Poorer** | **Middle** | **Richer** | **Richest** | **Urban** | **Rural** | **Below secondary** | **Secondary+** | **15-19 years** | **20-49 years** | **Male** | **Female** |
| --- | --- | --- | --- | --- | --- | --- | --- | --- | --- | --- | --- | --- | --- | --- |
| Albania | 1.002667 | 1.003855 | 1.037824 | 1.000676 | 1.026738 | 1.017792 | 1.004708 | 1.005606 | 1.006894 | 1.024601 | 0.9992406 | 1.00055 | 1.004873 | 1.017509 |
| Armenia | 1.001732 | 1.023988 | 0.9994063 | 1.021688 | 1.001608 | 1.020736 | 1.01384 | 1.0022 | N/A | 0.9998388 | 1.095156 | 1.025527 | 1.005178 | 1.016593 |
| Bangladesh | 1.011958 | 0.9992849 | 1.013422 | 1.003467 | 1.000092 | 0.9999547 | 1.004337 | 1.002826 | 0.9993314 | 1.005317 | 1.000253 | 1.002409 | 1.000171 | 0.9994854 |
| Benin | 1.003323 | 1.007048 | 1.001453 | 1.004111 | 0.9999928 | 1.000776 | 1.019278 | 0.999359 | 1.023961 | 1.001885 | 0.9993812 | 1.002524 | 1.006053 | 1.009029 |
| Bolivia | 1.006541 | 1.003975 | 0.9999273 | 0.9998803 | 1.007046 | 1.005119 | 1.029373 | 1.008777 | 1.014488 | 1.003571 | 1.004514 | 1.008001 | 1.000598 | 0.9994442 |
| Brazil | 1.002922 | N/A | N/A | N/A | N/A | N/A | 1.000364 | 1.005811 | 1.010448 | 1.004652 | 1.016363 | 1.002001 | 1.000934 | 1.051462 |
| Burkina Faso | 1.006426 | 1.01199 | 1.01704 | 0.999735 | 1.011822 | 1.004661 | 1.010258 | 1.071286 | 1.00683 | 1.002632 | 1.022563 | 1.001781 | 1.000558 | 1.004508 |
| Burundi | 1.003231 | 1.008261 | 1.018609 | 1.00402 | 0.9992595 | 1.000409 | 1.007926 | 1.004222 | 1.000172 | 1.004467 | 1.001048 | 0.9996578 | 1.006799 | 1.035021 |
| Cambodia | 1.001631 | 1.013304 | 1.006637 | 1.001005 | 1.003003 | 1.017852 | 1.006912 | 1.003392 | 1.009348 | 1.000256 | 1.034503 | 1.016464 | 1.002189 | 1.013312 |
| Cameroon | 1.036449 | 1.00418 | 1.005274 | 1.012187 | 1.010446 | 1.000458 | 0.9994261 | 1.005791 | 1.002193 | 0.9995136 | 0.9995762 | 1.008548 | 1.003904 | 1.000051 |
| Chad | 1.00545 | 1.000258 | 0.9992996 | 0.9992309 | 1.000753 | 1.0041 | 0.9991748 | 1.000828 | 0.9991737 | 1.007074 | 1.002084 | 1.000375 | 0.9992657 | 1.012197 |
| Colombia | 1.000144 | 0.9994251 | 1.000943 | 1.017739 | 1.006491 | 1.001583 | 1.010393 | 1.002933 | 0.9993084 | 1.007215 | 1.006727 | 1.008026 | 1.012192 | 1.015363 |
| Comoros | 1.010905 | 1.000455 | 0.9998193 | 1.016587 | 1.001681 | 1.028241 | 1.001485 | 1.006096 | 1.000015 | 1.010268 | 1.011003 | 1.000952 | 1.001026 | 1.011525 |
| Congo | 1.009574 | 1.008747 | 0.9998879 | 1.000714 | 1.01309 | 1.012554 | 1.007127 | 1.003271 | 0.9994009 | 1.009766 | 1.022133 | 1.000879 | 1.012367 | 1.000792 |
| Congo DR | 1.026235 | 1.006983 | 0.9994606 | 1.016241 | 1.006148 | 0.9994822 | 1.004357 | 1.000043 | 1.006996 | 0.9996845 | 1.004192 | 1.008506 | 1.000989 | 1.019788 |
| Cote d'Ivoire | 0.9992861 | 1.001943 | 1.013036 | 1.00496 | 1.007152 | 1.024374 | 1.000985 | 0.9992105 | 1.003823 | 1.001378 | 1.000656 | 1.011535 | 1.016455 | 1.00239 |
| Dominican Republic | 1.006576 | 1.00032 | 0.9999506 | 1.016457 | 1.000335 | 1.003224 | 1.002289 | 1.006925 | 1.015949 | 1.001426 | 1.006188 | 1.000679 | 1.000511 | 1.055073 |
| Egypt | 0.9995865 | 1.008865 | 1.013673 | 1.019108 | 1.005303 | 1.005789 | 1.006448 | 1.000394 | 1.001043 | 1.005276 | 0.9993384 | 1.004641 | 1.024231 | 0.9999349 |
| Ethiopia | 0.999284 | 1.004533 | 1.002851 | 1.000412 | 0.9994454 | 1.001895 | 0.9992797 | 1.009108 | 1.000875 | 1.010742 | 1.000755 | 1.000392 | 1.017995 | 1.045545 |
| Gabon | 1.023438 | 1.033271 | 1.008179 | 1.005979 | 1.002011 | 1.006418 | 1.00265 | 1.009565 | 1.000605 | 1.009029 | 1.006683 | 1.012573 | 1.014747 | 1.01325 |
| Ghana | 1.002185 | 1.003687 | 0.9997559 | 1.020041 | 1.003214 | 0.9995165 | 0.9994569 | 0.9994452 | 1.000014 | 1.004537 | 1.003126 | 1.003395 | 1.002727 | 1.004931 |
| Guatemala | 0.9998828 | 1.02808 | 1.000484 | 1.014736 | 1.000422 | 1.002118 | 1.060637 | 0.9994314 | 1.005244 | 1.000174 | 1.014514 | 1.009029 | 1.023991 | 0.9992911 |
| Guinea | 1.018117 | 1.000207 | 1.003003 | 1.015591 | 1.010245 | 1.007385 | 1.002047 | 1.009718 | 1.002392 | 1.002505 | 1.011686 | 1.022027 | 1.008924 | 1.006807 |
| Guyana | 1.008163 | 1.002712 | 0.9996351 | 1.010091 | 1.006073 | 1.080968 | 1.000266 | 1.010747 | 1.005433 | 0.9998882 | 1.004076 | 1.00417 | 1.010393 | 0.999983 |
| Haiti | 1.000866 | 1.004485 | 1.002356 | 1.001994 | 1.000123 | 0.9994846 | 1.004198 | 1.022764 | 0.999828 | 1.004966 | 1.000224 | 1.002667 | 1.025912 | 0.9994361 |
| Honduras | 0.9995549 | 1.007003 | 0.9992148 | 1.036145 | 1.005404 | 0.9994079 | 1.006241 | 1.000327 | 1.0022 | 1.032168 | 1.080523 | 1.020648 | 1.001334 | 1.015072 |
| India | 1.005627 | 1.002971 | 1.001801 | 0.9996831 | 1.001535 | 1.001256 | 1.062491 | 1.003439 | 1.001411 | 1.001366 | 1.014702 | 1.002327 | 1.012714 | 1.008267 |
| Indonesia | 1.005065 | 1.009496 | 1.003476 | 1.033066 | 1.010537 | 1.000655 | 1.004136 | 0.9996412 | 1.025355 | 1.005677 | 1.016028 | 1.025154 | 1.010421 | 1.000352 |
| Jordan | 1.007706 | 1.019589 | 1.008355 | 1.002011 | 1.020199 | 1.009049 | 1.003696 | 1.017833 | 1.003056 | 1.007028 | 1.017853 | 1.007614 | 1.031914 | 1.001852 |
| Kazakhstan | 1.000978 | 1.005621 | 1.00081 | 1.054299 | 1.035825 | 1.020995 | 1.003835 | 1.038747 | N/A | 0.9996519 | N/A | 1.005866 | N/A | 0.9999197 |
| Kenya | 0.9995114 | 1.01632 | 1.000301 | 1.013137 | 1.009872 | 1.000118 | 1.019596 | 1.037846 | 1.00158 | 1.002821 | 1.022224 | 1.000961 | 1.001903 | 1.000491 |
| Kyrgyz Republic | 1.000714 | 1.001479 | 1.000278 | 1.00374 | 1.001376 | 0.9994287 | 1.080424 | 1.001951 | N/A | 1.007617 | 1.002982 | 1.019097 | 1.009189 | 0.9997287 |
| Lesotho | 1.016575 | 1.008929 | 1.001172 | 1.000575 | 1.002345 | 1.004365 | 1.002388 | 1.005858 | 1.002564 | 1.000569 | 1.012346 | 1.016685 | 1.002382 | 1.00122 |
| Liberia | 1.001176 | 0.9997194 | 0.9995625 | 1.002067 | 1.030167 | 1.000005 | 1.051808 | 1.00289 | 1.006702 | 1.004278 | 1.000997 | 1.001199 | 1.00773 | 1.007343 |
| Madagascar | 1.002111 | 1.001958 | 1.001109 | 0.9994652 | 0.9997159 | 1.001756 | 1.000709 | 1.00146 | 1.00187 | 1.001565 | 1.001181 | 1.010722 | 0.9996676 | 1.000695 |
| Malawi | 1.000839 | 1.002054 | 1.001908 | 1.001004 | 0.9994057 | 1.000931 | 0.9995335 | 1.036338 | 1.003039 | 0.999765 | 1.000266 | 0.9998617 | 1.000026 | 1.0079 |
| Maldives | 1.000563 | 1.069479 | 1.001524 | 1.002339 | 1.000349 | 1.002641 | 1.015299 | 1.010391 | 1.020058 | 1.034934 | 1.000266 | 0.9994263 | 0.9995606 | 1.0285 |
| Mali | 1.001618 | 1.002 | 0.9995323 | 1.000323 | 0.9993391 | 1.003263 | 1.022163 | 1.023838 | 1.007608 | 1.004604 | 1.002271 | 1.002564 | 1.003108 | 1.010588 |
| Morocco | 1.000619 | 0.9994991 | 1.033671 | 1.096875 | 1.000259 | 1.07974 | 0.999581 | 1.022253 | 1.002598 | 1.012788 | 1.002553 | 1.007208 | 1.086417 | 1.000416 |
| Mozambique | 1.003853 | 0.9994719 | 1.013658 | 1.000102 | 1.001007 | 1.019674 | 0.999517 | 1.00006 | 0.9998502 | 1.022109 | 1.020367 | 1.001028 | 1.003744 | 0.9993051 |
| Namibia | 0.9994454 | 1.005458 | 1.000543 | 1.008444 | 1.001991 | 1.008149 | 1.008784 | 1.005209 | 1.000389 | 1.001676 | 1.000018 | 1.000026 | 1.002706 | 1.013839 |
| Nepal | 1.036021 | 0.9998121 | 1.00739 | 1.001048 | 0.9993076 | 1.000301 | 1.018567 | 1.00209 | 1.003798 | 1.021619 | 1.005175 | 1.000576 | 1.008698 | 0.999221 |
| Nicaragua | 1.013587 | 1.004547 | 1.005998 | 1.001619 | 1.004476 | 0.9997552 | 1.013295 | 1.004196 | 1.001742 | 1.001193 | 1.000075 | 1.007911 | 1.002558 | 1.008234 |
| Niger | 0.9993776 | 1.004759 | 0.999947 | 1.000673 | 1.000387 | 1.000453 | 1.010375 | 1.004843 | 1.000658 | 1.001214 | 1.013259 | 1.009207 | 1.008398 | 1.010915 |
| Nigeria | 1.022282 | 1.005645 | 1.008093 | 0.9997436 | 0.9998209 | 0.9992535 | 1.015293 | 1.023402 | 0.9992217 | 1.005281 | 1.00133 | 1.003072 | 1.005757 | 1.002137 |
| Pakistan | 0.9993682 | 1.003909 | 1.006664 | 1.019471 | 1.000501 | 1.008476 | 1.001847 | 1.010013 | 1.011282 | 0.9996445 | 1.040586 | 1.002295 | 1.003562 | 1.009303 |
| Peru | 1.01639 | 1.000683 | 0.9998736 | 1.035343 | 1.077541 | 0.9992602 | 1.007105 | 1.002769 | 0.9999869 | 1.011887 | 1.014933 | 1.002725 | 1.000861 | 1.000869 |
| Philippines | 1.000157 | 1.004041 | 1.001945 | 1.010854 | 1.005312 | 1.000323 | 1.004355 | 0.9993192 | 1.026278 | 1.009099 | 0.9995971 | 1.016006 | 0.9993904 | 1.004577 |
| Rwanda | 1.00005 | 1.012321 | 1.01454 | 1.002881 | 0.9993422 | 0.9992818 | 0.9996535 | 1.004546 | 1.030316 | 1.00927 | 0.9995492 | 1.007913 | 1.012726 | 1.010182 |
| Senegal | 1.001048 | 1.010366 | 1.001141 | 1.009227 | 1.002725 | 1.004654 | 1.012008 | 0.9995415 | 1.00178 | 1.004542 | 1.006036 | 1.000528 | 1.023487 | 1.008952 |
| Sierra Leone | 1.000158 | 1.000414 | 0.9998751 | 1.015799 | 1.037173 | 1.006102 | 0.9993376 | 1.008022 | 1.004475 | 0.9998798 | 1.016668 | 1.003162 | 1.001622 | 1.001526 |
| South Africa | 1.005667 | 0.9996474 | 1.009986 | 1.016579 | 1.004122 | 1.0033 | 1.012847 | 1.0005 | 0.9994645 | 1.010337 | 1.015663 | 1.017619 | 1.002241 | 1.019266 |
| Tajikistan | 1.003758 | 1.060466 | 1.003503 | 1.000893 | 1.003853 | 0.9993315 | 1.012836 | 1.002427 | 1.036469 | 1.015817 | 1.015663 | 0.9998886 | 1.000884 | 1.030324 |
| Tanzania | 1.004558 | 1.006661 | 1.002624 | 1.013936 | 1.00003 | 1.003228 | 1.005317 | 1.01377 | 0.9992189 | 1.019144 | 0.9992442 | 1.003308 | 1.014809 | 0.9999209 |
| Timor-Leste | 1.019128 | 1.000697 | 1.020345 | 1.00168 | 1.005109 | 1.005177 | 1.00412 | 1.000383 | 1.014709 | 1.001193 | 1.001145 | 0.9998825 | 1.026195 | 1.007129 |
| Togo | 1.000708 | 1.03018 | 1.001838 | 0.9994397 | 1.013061 | 1.012298 | 1.000772 | 0.9999945 | 1.001724 | 0.9992712 | 1.015794 | 1.014579 | 1.003184 | 1.011413 |
| Turkey | 0.9997431 | 1.002228 | 0.9998825 | 1.002665 | 0.9994884 | 1.001728 | 1.006686 | 1.000782 | 0.9994107 | 1.006938 | 1.001079 | 1.003189 | 1.004391 | 1.001192 |
| Uganda | 1.024807 | 0.9997107 | 1.011395 | 1.002242 | 1.000079 | 1.001367 | 1.024159 | 1.010154 | 1.000012 | 1.001414 | 1.002271 | 1.004415 | 1.015225 | 1.009358 |
| Vietnam | 1.03448 | 1.008104 | 1.056456 | 1.005158 | 1.000026 | 1.006652 | 0.9994352 | 1.002208 | 1.003892 | 1.011017 | 1.012337 | 0.9992733 | 1.00273 | 1.000302 |
| Yemen | 1.006335 | N/A | N/A | N/A | N/A | N/A | 1.002619 | 1.002298 | 1.003635 | 1.001852 | 1.011775 | 1.017115 | 1.002942 | 1.002601 |
| Zambia | 1.003019 | 1.002795 | 1.003995 | 1.004875 | 1.012384 | 1.000871 | 1.002981 | 1.000537 | 1.002601 | 1.000866 | 1.003023 | 0.9993257 | 1.010675 | 0.9991958 |
| Zimbabwe | 1.004683 | 1.004597 | 1.002752 | 0.9993612 | 1.000094 | 0.9993122 | 1.00453 | 1.001615 | 1.032171 | 0.9995941 | 1.000141 | 1.008766 | 1.014084 | 0.9993985 |

**Note:** N/A denotes not application and refers that the indicator has no sufficient data to make projections

Table S12: Cross-validation of estimates of the coverage of interventions drawn from original micro data and regression models for some countries during the most recent survey years in low- and middle-income countries

| **Country** | **Survey year** | **mDFPS** | | | **ANC** | | | **SBA** | | | **BCG** | | | **DPT** | | | **Measles** | | | **ORT** | | | **ARI care** | | |
| --- | --- | --- | --- | --- | --- | --- | --- | --- | --- | --- | --- | --- | --- | --- | --- | --- | --- | --- | --- | --- | --- | --- | --- | --- | --- |
|  |  | **O** | **E** | **D** | **O** | **E** | **D** | **O** | **E** | **D** | **O** | **E** | **D** | **O** | **E** | **D** | **O** | **E** | **D** | **O** | **E** | **D** | **O** | **E** | **D** |
| Bangladesh | 2014 | 72.7 | 72.0 | 0.7 | 68.6 | 66.7 | 1.9 | 41.8 | 37.7 | 4.0 | 97.9 | 98.2 | -0.3 | 91.3 | 93.5 | -2.2 | 86.2 | 87.3 | -1.1 | 85.0 | 85.4 | -0.4 | 42.5 | 40.9 | 1.6 |
| Bolivia | 2008 | 43.4 | 46.6 | -3.2 | 88.3 | 88.0 | 0.3 | 71.4 | 71.2 | 0.2 | 98.5 | 98.1 | 0.3 | 85.2 | 84.1 | 1.2 | 72.1 | 70.2 | 1.9 | 43.8 | 43.3 | 0.5 | 50.9 | 51.4 | -0.5 |
| Brazil | 1996 | 79.8 | 79.5 | 0.4 | 89.2 | 88.7 | 0.4 | 88.8 | 88.4 | 0.4 | 92.7 | 92.4 | 0.3 | 80.8 | 80.2 | 0.5 | 87.2 | 86.8 | 0.4 | 53.6 | 53.2 | 0.5 | 46.2 | 46.5 | -0.3 |
| Burundi | 2017 | 40.3 | 40.1 | 0.2 | 54.0 | 58.3 | -4.3 | 86.4 | 86.0 | 0.4 | 97.6 | 97.5 | 0.1 | 96.6 | 96.5 | 0.1 | 93.7 | 93.3 | 0.3 | 37.8 | 38.0 | -0.2 | 55.2 | 55.0 | 0.1 |
| Cameroon | 2011 | 39.2 | 42.7 | -3.5 | 82.8 | 83.3 | -0.6 | 57.8 | 58.6 | -0.7 | 87.1 | 87.6 | -0.5 | 68.4 | 69.9 | -1.5 | 70.9 | 70.3 | 0.5 | 22.4 | 22.7 | -0.3 | 24.2 | 27.2 | -3.1 |
| Comoros | 2012 | 28.8 | 29.2 | -0.4 | 92.5 | 92.3 | 0.2 | 83.8 | 83.6 | 0.2 | 85.4 | 85.3 | 0.1 | 72.7 | 72.6 | 0.1 | 75.3 | 75.0 | 0.3 | 61.3 | 61.4 | -0.1 | 37.0 | 37.1 | -0.1 |
| Congo | 2012 | 38.0 | 38.5 | -0.5 | 92.0 | 91.6 | 0.3 | 90.9 | 90.6 | 0.3 | 93.9 | 93.7 | 0.2 | 55.9 | 55.7 | 0.1 | 74.9 | 74.3 | 0.6 | 37.0 | 36.9 | 0.1 | 50.0 | 50.2 | -0.2 |
| Congo DR | 2014 | 19.5 | 19.9 | -0.4 | 77.1 | 76.4 | 0.7 | 50.4 | 50.6 | -0.1 | 83.4 | 82.9 | 0.5 | 60.6 | 60.1 | 0.4 | 71.6 | 71.6 | 0.0 | 42.1 | 42.4 | -0.3 | 35.3 | 35.4 | -0.1 |
| Cote d'Ivoire | 2012 | 32.2 | 34.7 | -2.6 | 90.1 | 88.0 | 2.0 | 58.8 | 54.8 | 4.1 | 83.4 | 84.1 | -0.7 | 63.8 | 65.3 | -1.5 | 64.5 | 65.9 | -1.4 | 22.5 | 24.9 | -2.4 | 32.9 | 30.8 | 2.1 |
| Dominican Republic | 2013 | 82.7 | 84.4 | -1.7 | 95.9 | 96.5 | -0.6 | 98.9 | 99.0 | -0.1 | 92.6 | 95.2 | -2.5 | 82.4 | 81.6 | 0.7 | 88.6 | 86.8 | 1.7 | 53.3 | 51.8 | 1.5 | 65.4 | 69.0 | -3.6 |
| Gabon | 2012 | 44.1 | 44.6 | -0.6 | 94.8 | 94.7 | 0.1 | 86.0 | 85.8 | 0.2 | 91.5 | 91.2 | 0.3 | 22.2 | 22.4 | -0.2 | 74.3 | 73.8 | 0.6 | 37.7 | 38.1 | -0.4 | 47.4 | 47.2 | 0.1 |
| Guatemala | 2015 | 66.2 | 66.1 | 0.1 | 96.4 | 96.2 | 0.2 | 94.9 | 94.4 | 0.5 | 98.0 | 98.0 | 0.0 | 84.6 | 84.5 | 0.1 | 63.2 | 64.1 | -0.9 | 52.7 | 50.7 | 2.1 | 48.6 | 47.9 | 0.6 |
| Guinea | 2012 | 24.6 | 25.8 | -1.2 | 77.8 | 74.9 | 2.9 | 40.8 | 38.1 | 2.8 | 82.5 | 82.4 | 0.1 | 49.8 | 50.2 | -0.5 | 61.8 | 59.7 | 2.0 | 36.6 | 35.8 | 0.8 | 33.0 | 32.9 | 0.0 |
| Honduras | 2012 | 76.0 | 75.7 | 0.3 | 96.5 | 96.3 | 0.1 | 97.0 | 96.8 | 0.1 | 99.1 | 99.1 | 0.0 | 95.2 | 95.1 | 0.1 | 87.7 | 87.2 | 0.4 | 60.1 | 59.4 | 0.7 | 63.7 | 63.3 | 0.3 |
| Kazakhstan | 1999 | 67.7 | 67.4 | 0.3 | 94.1 | 93.9 | 0.2 | 99.0 | 99.0 | 0.0 | 89.9 | 89.7 | 0.2 | 88.7 | 88.4 | 0.3 | 78.5 | 78.0 | 0.4 | 32.0 | 32.4 | -0.4 | 49.2 | 49.0 | 0.2 |
| Kyrgyz Republic | 2012 | 57.2 | 56.9 | 0.4 | 96.1 | 96.0 | 0.1 | 99.3 | 99.2 | 0.0 | 99.1 | 99.0 | 0.0 | 85.3 | 84.9 | 0.4 | 89.3 | 89.1 | 0.2 | 69.8 | 69.5 | 0.2 | 34.7 | 35.1 | -0.5 |
| Lesotho | 2014 | 78.9 | 78.7 | 0.2 | 95.3 | 94.7 | 0.5 | 80.4 | 78.3 | 2.1 | 98.0 | 97.4 | 0.6 | 85.4 | 84.9 | 0.5 | 90.1 | 88.0 | 2.1 | 74.9 | 73.5 | 1.4 | 65.6 | 65.0 | 0.6 |
| Maldives | 2017 | 22.6 | 22.9 | -0.3 | 98.8 | 98.8 | 0.0 | 40.0 | 40.1 | -0.1 | 91.8 | 91.5 | 0.3 | 84.5 | 84.4 | 0.2 | 88.1 | 87.6 | 0.5 | 84.0 | 83.6 | 0.4 | 82.7 | 82.3 | 0.4 |
| Morocco | 2004 | 73.2 | 73.0 | 0.1 | 67.6 | 67.1 | 0.5 | 65.1 | 64.7 | 0.4 | 98.4 | 98.3 | 0.1 | 94.9 | 94.6 | 0.2 | 90.4 | 90.0 | 0.4 | 28.0 | 28.3 | -0.3 | 37.8 | 38.0 | -0.2 |
| Namibia | 2013 | 80.4 | 81.2 | -0.8 | 96.6 | 96.5 | 0.1 | 89.2 | 87.9 | 1.3 | 94.2 | 94.8 | -0.6 | 83.9 | 85.5 | -1.6 | 89.5 | 88.7 | 0.7 | 78.2 | 75.3 | 2.9 | 59.8 | 60.5 | -0.7 |
| Nicaragua | 2001 | 79.0 | 78.4 | 0.6 | 84.4 | 84.0 | 0.4 | 70.0 | 69.8 | 0.2 | 95.3 | 95.1 | 0.2 | 83.4 | 83.0 | 0.4 | 75.6 | 75.2 | 0.4 | 54.3 | 54.2 | 0.0 | 57.9 | 58.0 | -0.1 |
| Pakistan | 2018 | 48.6 | 51.0 | -2.4 | 87.3 | 85.3 | 1.9 | 69.7 | 67.2 | 2.5 | 87.8 | 87.9 | -0.1 | 75.1 | 72.6 | 2.4 | 72.4 | 69.4 | 3.0 | 39.4 | 41.9 | -2.5 | 76.9 | 77.3 | -0.4 |
| Philippines | 2017 | 56.1 | 55.5 | 0.6 | 96.3 | 96.2 | 0.0 | 86.8 | 82.2 | 4.6 | 89.8 | 93.3 | -3.5 | 79.3 | 83.6 | -4.3 | 79.5 | 82.4 | -2.8 | 51.0 | 55.9 | -4.9 | 63.3 | 59.4 | 3.9 |
| South Africa | 2016 | 76.7 | 76.7 | 0.0 | 94.4 | 94.2 | 0.2 | 96.6 | 96.5 | 0.1 | 91.9 | 91.6 | 0.3 | 63.8 | 63.6 | 0.2 | 85.1 | 84.9 | 0.2 | 80.6 | 80.2 | 0.4 | 63.3 | 62.7 | 0.6 |
| Tajikistan | 2017 | 44.9 | 45.1 | -0.2 | 86.6 | 86.1 | 0.4 | 95.2 | 94.9 | 0.2 | 95.2 | 95.1 | 0.1 | 86.5 | 86.2 | 0.3 | 82.0 | 81.5 | 0.5 | 66.9 | 66.4 | 0.5 | 56.8 | 56.6 | 0.2 |
| Timor-Leste | 2016 | 38.0 | 38.0 | 0.1 | 85.6 | 85.0 | 0.6 | 57.5 | 57.3 | 0.2 | 80.7 | 80.3 | 0.4 | 61.9 | 61.8 | 0.1 | 69.3 | 69.3 | 0.1 | 77.3 | 77.0 | 0.3 | 72.1 | 71.8 | 0.3 |
| Vietnam | 2002 | 66.6 | 66.3 | 0.2 | 74.8 | 74.5 | 0.4 | 77.4 | 77.0 | 0.4 | 93.4 | 93.1 | 0.2 | 72.4 | 71.7 | 0.7 | 83.2 | 82.8 | 0.4 | 41.9 | 41.8 | 0.0 | 71.3 | 70.6 | 0.7 |
| Yemen | 2013 | 24.5 | 24.8 | -0.3 | 60.1 | 59.5 | 0.5 | 45.2 | 45.3 | -0.1 | 67.7 | 67.8 | -0.1 | 59.8 | 59.4 | 0.4 | 63.5 | 63.2 | 0.3 | 28.3 | 28.4 | -0.1 | 33.3 | 33.5 | -0.2 |

**Note:** mDFPS= Demand for family planning satisfied with modern contraceptive methods, ANC= Antenatal care visits, SBA= Skilled birth attendance, BCG= BCG immunization, DPT= Three doses of DPT immunization, Measles= Measles immunization, ORT= Oral rehydration therapy for diarrhoea treatment, ARI Care= Care seeking for symptoms of acute respiratory infections, O= Observed value from the original data, E= Estimated value drawn from the regression model, D= Absolute difference between observed and estimated value.s

Figure S1: Progress and projections of demand for family planning satisfied with modern contraceptive methods (mDFPS) among women 15-49 years of age in low- and middle-income countries

Figure S2: Progress and projections of antenatal care (ANC) visits among women 15-49 years of age in low- and middle-income countries

Figure S3: Progress and projections of skilled birth attendance (SBA) among women 15-49 years of age in low- and middle-income countries

Figure S4: Progress and projections of BCG immunization (BCG) among children 12-23 months of age in low- and middle-income countries

Figure S5: Progress and projections of three doses of DPT immunization (DPT) among children 12-23 months of age in low- and middle-income countries

Figure S6: Progress and projections of Measles immunization (Measles) among children 12-23 months of age in low- and middle-income countries

Figure S7: Progress and projections of oral rehydration therapy (ORT) for diarrhoea treatment among children 0-59 months of age in low- and middle-income countries

Figure S8: Progress and projections of care seeking for acute respiratory infections (ARI care) among children 0-59 months of age in low- and middle-income countries

Figure S9: Predicted coverage (in %) of demand for family planning satisfied with modern contraceptive method (mDFPS) among women 15-49 years of age in low- and middle-income countries in 2030

Figure S10: Predicted coverage (in %) of antenatal care (ANC) visits among women 15-49 years of age in low- and middle-income countries in 2030

Figure S11: Predicted coverage (in %) of skilled birth attendance (SBA) among women 15-49 years of age in low- and middle-income countries in 2030

Figure S12: Predicted coverage (in %) of BCG immunization (BCG) among children 12-23 months of age in low- and middle-income countries in 2030

Figure S13: Predicted coverage (in %) of three doses of DPT immunization (DPT) among children 12-23 months of age in low- and middle-income countries in 2030

Figure S14: Predicted coverage (in %) of Measles immunization (Measles) among children 12-23 months of age in low- and middle-income countries in 2030

Figure S15: Predicted coverage (in %) of oral rehydration therapy (ORT) for diarrhoea treatment among children 0-59 months of age in low- and middle-income countries in 2030

Figure S16: Predicted coverage (in %) of care seeking for acute respiratory infections (ARI care) among children 0-59 months of age in low- and middle-income countries in 2030

Figure S17: Trends in predicted rural-urban gaps in composite coverage index (CCI) in low- and middle-income countries

Figure S18: Trends in predicted below secondary-secondary+ education gaps in composite coverage index (CCI) in low- and middle-income countries

Figure S19: Trends in predicted adolescent-adult gaps in composite coverage index (CCI) in low- and middle-income countries

Figure S20: Trends in predicted poorest-richest gaps in the coverage of demand for family planning satisfied with modern contraceptive method (mDFPS) among women 15-49 years of age in low- and middle-income countries

Figure S21: Trends in predicted rural-urban gaps in the coverage of demand for family planning satisfied with modern contraceptive method (mDFPS) among women 15-49 years of age in low- and middle-income countries

Figure S22: Trends in predicted below secondary-secondary+ education gaps in the coverage of demand for family planning satisfied with modern contraceptive method (mDFPS) among women 15-49 years of age in low- and middle-income countries

Figure S23: Trends in predicted adolescent-adult gaps in the coverage of demand for family planning satisfied with modern contraceptive method (mDFPS) among women 15-49 years of age in low- and middle-income countries

Figure S24: Trends in predicted poorest-richest gaps in the coverage of antenatal care (ANC) visits among women 15-49 years of age in low- and middle-income countries

Figure S25: Trends in predicted rural-urban gaps in the coverage of antenatal care (ANC) visits among women 15-49 years of age in low- and middle-income countries

Figure S26: Trends in predicted below secondary-secondary+ education gaps in the coverage of antenatal care (ANC) visits among women 15-49 years of age in low- and middle-income countries

Figure S27: Trends in predicted adolescent-adult gaps in the coverage of antenatal care (ANC) visits among women 15-49 years of age in low- and middle-income countries

Figure S28: Trends in predicted poorest-richest gaps in the coverage of skilled birth attendance (SBA) among women 15-49 years of age in low- and middle-income countries

Figure S29: Trends in predicted rural-urban gaps in the coverage of skilled birth attendance (SBA) among women 15-49 years of age in low- and middle-income countries

Figure S30: Trends in predicted below secondary-secondary+ education gaps in the coverage of skilled birth attendance (SBA) among women 15-49 years of age in low- and middle-income countries

Figure S31: Trends in predicted adolescent-adult gaps in the coverage of skilled birth attendance (SBA) among women 15-49 years of age in low- and middle-income countries

Figure S32: Trends in predicted poorest-richest gaps in the coverage of BCG immunization (BCG) among children 12-23 months of age in low- and middle-income countries

Figure S33: Trends in predicted rural-urban gaps in the coverage of BCG immunization (BCG) among children 12-23 months of age in low- and middle-income countries

Figure S34: Trends in predicted mother’s below secondary-secondary+ education gaps in the coverage of BCG immunization (BCG) among children 12-23 months of age in low- and middle-income countries

Figure S35: Trends in predicted adolescent-adult motherhood gaps in the coverage of BCG immunization (BCG) among children 12-23 months of age in low- and middle-income countries

Figure S36: Trends in predicted male-female gaps in the coverage of BCG immunization (BCG) among children 12-23 months of age in low- and middle-income countries

Figure S37: Trends in predicted poorest-richest gaps in the coverage of three doses of DPT immunization (DPT) among children 12-23 months of age in low- and middle-income countries

Figure S38: Trends in predicted rural-urban gaps in the coverage of three doses of DPT immunization (DPT) among children 12-23 months of age in low- and middle-income countries

Figure S39: Trends in predicted mother’s below secondary-secondary+ education gaps in the coverage of three doses of DPT immunization (DPT) among children 12-23 months of age in low- and middle-income countries

Figure S40: Trends in predicted adolescent-adult motherhood gaps in the coverage of three doses of DPT immunization (DPT) among children 12-23 months of age in low- and middle-income countries

Figure S41: Trends in predicted male-female gaps in the coverage of three doses of DPT immunization (DPT) among children 12-23 months of age in low- and middle-income countries

Figure S42: Trends in predicted poorest-richest gaps in the coverage of Measles immunization (Measles) among children 12-23 months of age in low- and middle-income countries

Figure S43: Trends in predicted rural-urban gaps in the coverage of Measles immunization (Measles) among children 12-23 months of age in low- and middle-income countries

Figure S44: Trends in predicted mother’s below secondary-secondary+ education gaps in the coverage of Measles immunization (Measles) among children 12-23 months of age in low- and middle-income countries

Figure S45: Trends in predicted adolescent-adult motherhood gaps in the coverage of Measles immunization (Measles) among children 12-23 months of age in low- and middle-income countries

Figure S46: Trends in predicted male-female gaps in the coverage of Measles immunization (Measles) among children 12-23 months of age in low- and middle-income countries

Figure S47: Trends in predicted poorest-richest gaps in the coverage of oral rehydration therapy (ORT) for diarrhoea treatment among children 0-59 months of age in low- and middle-income countries

Figure S48: Trends in predicted rural-urban gaps in the coverage of oral rehydration therapy (ORT) for diarrhoea treatment among children 0-59 months of age in low- and middle-income countries

Figure S49: Trends in predicted mother’s below secondary-secondary+ education gaps in the coverage of oral rehydration therapy (ORT) for diarrhoea treatment among children 0-59 months of age in low- and middle-income countries

Figure S50: Trends in predicted adolescent-adult motherhood gaps in the coverage of oral rehydration therapy (ORT) for diarrhoea treatment among children 0-59 months of age in low- and middle-income countries

Figure S51: Trends in predicted male-female gaps in the coverage of oral rehydration therapy (ORT) for diarrhoea treatment among children 0-56 months of age in low- and middle-income countries

Figure S52: Trends in predicted poorest-richest gaps in the coverage of care seeking for acute respiratory infections (ARI care) among children 0-59 months of age in low- and middle-income countries

Figure S53: Trends in predicted rural-urban gaps in the coverage of care seeking for acute respiratory infections (ARI care) among children 0-59 months of age in low- and middle-income countries

Figure S54: Trends in predicted mothers below secondary-secondary+ education gaps in the coverage of care seeking for acute respiratory infections (ARI care) among children 0-59 months of age in low- and middle-income countries

Figure S55: Trends in predicted adolescent-adult motherhood gaps in the coverage of care seeking for acute respiratory infections (ARI care) among children 0-59 months of age in low- and middle-income countries

Figure S56: Trends in predicted male-female gaps in the coverage of care seeking for acute respiratory infections (ARI care) among children 0-59 months of age in low- and middle-income countries

Figure S57: Projected gaps in the coverage of demand for family planning satisfied with modern contraceptive method (mDFPS) among women 15-49 years of age in low- and middle-income countries in 2030 by wealth quintiles

Figure S58: Projected gaps in the coverage of demand for family planning satisfied with modern contraceptive method (mDFPS) among women 15-49 years of age in low- and middle-income countries in 2030 by place of residence

Figure S59: Projected gaps in the coverage of demand for family planning satisfied with modern contraceptive method (mDFPS) among women 15-49 years of age in low- and middle-income countries in 2030 by education

Figure S60: Projected gaps in the coverage of demand for family planning satisfied with modern contraceptive method (mDFPS) among women 15-49 years of age in low- and middle-income countries in 2030 by age

Figure S61: Projected gaps in the coverage of antenatal care (ANC) visits among women 15-49 years of age in low- and middle-income countries in 2030 by wealth quintiles

Figure S62: Projected gaps in the coverage of antenatal care (ANC) visits among women 15-49 years of age in low- and middle-income countries in 2030 by place of residence

Figure S63: Projected gaps in the coverage of antenatal care (ANC) visits among women 15-49 years of age in low- and middle-income countries in 2030 by education

Figure S64: Projected gaps in the coverage of antenatal care (ANC) visits among women 15-49 years of age in low- and middle-income countries in 2030 by age

Figure S65: Projected gaps in the coverage of skilled birth attendance (SBA) among women 15-49 years of age in low- and middle-income countries in 2030 by wealth quintiles

Figure S66: Projected gaps in the coverage of skilled birth attendance (SBA) among women 15-49 years of age in low- and middle-income countries in 2030 by place of residence

Figure S67: Projected gaps in the coverage of skilled birth attendance (SBA) among women 15-49 years of age in low- and middle-income countries in 2030 by education

Figure S68: Projected gaps in the coverage of skilled birth attendance (SBA) among women 15-49 years of age in low- and middle-income countries in 2030 by age

Figure S69: Projected gaps in the coverage of BCG immunization (BCG) among children 12-23 months of age in low- and middle-income countries in 2030 by wealth quintiles

Figure S70: Projected gaps in the coverage of BCG immunization (BCG) among children 12-23 months of age in low- and middle-income countries in 2030 by place of residence

Figure S71: Projected gaps in the coverage of BCG immunization (BCG) among children 12-23 months of age in low- and middle-income countries in 2030 by mother’s education

Figure S72: Projected gaps in the coverage of BCG immunization (BCG) among children 12-23 months of age in low- and middle-income countries in 2030 by mother’s age

Figure S73: Projected gaps in the coverage of three doses of DPT immunization (DPT) among children 12-23 months of age in low- and middle-income countries in 2030 by wealth quintiles

Figure S74: Projected gaps in the coverage of three doses of DPT immunization (DPT) among children 12-23 months of age in low- and middle-income countries in 2030 by place of residence

Figure S75: Projected gaps in the coverage of three doses of DPT immunization (DPT) among children 12-23 months of age in low- and middle-income countries in 2030 by mother’s education

Figure S76: Projected gaps in the coverage of three doses of DPT immunization (DPT) among children 12-23 months of age in low- and middle-income countries in 2030 by mother’s age

Figure S77: Projected gaps in the coverage of Measles immunization (Measles) among children 12-23 months of age in low- and middle-income countries in 2030 by wealth quintiles

Figure S78: Projected gaps in the coverage of Measles immunization (Measles) among children 12-23 months of age in low- and middle-income countries in 2030 by place of residence

Figure S79: Projected gaps in the coverage of Measles immunization (Measles) among children 12-23 months of age in low- and middle-income countries in 2030 by mother’s education

Figure S80: Projected gaps in the coverage of Measles immunization (Measles) among children 12-23 months of age in low- and middle-income countries in 2030 by mother’s age

Figure S81: Projected gaps in the coverage of oral rehydration therapy (ORT) for diarrhoea treatment among children 0-59 months of age in low- and middle-income countries in 2030 by wealth quintiles

Figure S82: Projected gaps in the coverage of oral rehydration therapy (ORT) for diarrhoea treatment among children 0-59 months of age in low- and middle-income countries in 2030 by place of residence

Figure S83: Projected gaps in the coverage of oral rehydration therapy (ORT) for diarrhoea treatment among children 0-59 months of age in low- and middle-income countries in 2030 by mother’s education

Figure S84: Projected gaps in the coverage of oral rehydration therapy (ORT) for diarrhoea treatment among children 0-59 months of age in low- and middle-income countries in 2030 by mother’s age

Figure S85: Projected gaps in the coverage of care seeking for acute respiratory infections (ARI care) among children 0-59 months of age in low- and middle-income countries in 2030 by wealth quintiles

Figure S86: Projected gaps in the coverage of care seeking for acute respiratory infections (ARI care) among children 0-59 months of age in low- and middle-income countries in 2030 by place of residence

Figure S87: Projected gaps in the coverage of care seeking for acute respiratory infections (ARI care) among children 0-59 months of age in low- and middle-income countries in 2030 by mother’s education

Figure S88: Projected gaps in the coverage of care seeking for acute respiratory infections (ARI care) among children 0-59 months of age in low- and middle-income countries in 2030 by mother’s age

Figure S89: Projected gaps in the coverage of BCG immunization (BCG) among children 12-23 months of age in low- and middle-income countries in 2030 by child sex

Figure S90: Projected gaps in the coverage of three doses of DPT immunization (DPT) among children 12-23 months of age in low- and middle-income countries in 2030 by child sex

Figure S91: Projected gaps in the coverage of Measles immunization (Measles) among children 12-23 months of age in low- and middle-income countries in 2030 by child sex

Figure S92: Projected gaps in the coverage of oral rehydration therapy (ORT) for diarrhoea treatment among children 0-59 months of age in low- and middle-income countries in 2030 by child sex

Figure S93: Projected gaps in the coverage of care seeking for acute respiratory infections (ARI care) among children 0-59 months of age in low- and middle-income countries in 2030 by child sex

Table S13: Predictive probability of achieving universal coverage of maternal, newborn and child healthcare services in low- and middle-income countries in 2030

| **Country** | **CCI** | **mDFPS** | **ANC** | **SBA** | **BCG** | **DPT** | **Measles** | **ORT** | **ARI care** |
| --- | --- | --- | --- | --- | --- | --- | --- | --- | --- |
| Afghanistan | N/A | N/A | 0% | 1% | N/A | N/A | N/A | N/A | N/A |
| Albania | N/A | 0% | 0% | 0% | 100% | N/A | 100% | 0% | 0% |
| Angola | N/A | N/A | 0% | 0% | N/A | N/A | N/A | N/A | N/A |
| Armenia | 0% | 0% | 100% | 100% | 100% | 4% | 1% | 0% | 0% |
| Bangladesh | 0% | 0% | 0% | 0% | 99% | 30% | 0% | 0% | 0% |
| Benin | 0% | 0% | 0% | 0% | 0% | 0% | 0% | 0% | 0% |
| Bolivia | 0% | 0% | 70% | 0% | 100% | 73% | 0% | 0% | 0% |
| Brazil | 72% | 65% | 98% | 96% | 99% | 89% | 85% | 13% | 39% |
| Burkina Faso | 0% | 0% | 0% | 0% | 68% | 67% | 14% | 0% | 0% |
| Burundi | 0% | 0% | 0% | 43% | 1% | 22% | 2% | 0% | 0% |
| Cambodia | 1% | 0% | 100% | 92% | 97% | 19% | 2% | 0% | 1% |
| Cameroon | 0% | 0% | 0% | 0% | 1% | 0% | 0% | 0% | 0% |
| Chad | 0% | 0% | 0% | 0% | 0% | 0% | 0% | 0% | 0% |
| Colombia | 1% | 0% | 85% | 5% | 48% | 0% | 0% | 0% | 0% |
| Comoros | 0% | 0% | 13% | 10% | 0% | 0% | 0% | 0% | 0% |
| Congo | 1% | 1% | 76% | 27% | 31% | 0% | 3% | 0% | 0% |
| Congo DR | 0% | 0% | 5% | 0% | 9% | 1% | 2% | 0% | 0% |
| Cote d'Ivoire | 0% | 0% | 11% | 1% | 0% | 0% | 0% | 0% | 0% |
| Dominican Republic | 0% | 0% | 11% | 99% | 36% | 0% | 0% | 0% | 0% |
| Egypt | 0% | 0% | 4% | 22% | 100% | 99% | 0% | 0% | 0% |
| Ethiopia | 0% | 0% | 0% | 0% | 0% | 0% | 0% | 0% | 0% |
| Gabon | 0% | 0% | 5% | 1% | 3% | 0% | 1% | 0% | 0% |
| Ghana | 0% | 0% | 2% | 0% | 91% | 2% | 5% | 0% | 0% |
| Guatemala | 0% | 0% | 75% | 97% | 97% | 0% | 0% | 0% | 0% |
| Guinea | 0% | 0% | 0% | 0% | 0% | 0% | 0% | 0% | 0% |
| Guyana | N/A | 95% | 89% | N/A | N/A | N/A | N/A | N/A | 4% |
| Haiti | 0% | 0% | 0% | 0% | 0% | 0% | 0% | 0% | 0% |
| Honduras | 12% | 3% | 91% | 100% | 93% | 39% | 7% | 1% | 4% |
| India | 0% | 0% | 0% | 0% | 1% | 0% | 0% | 0% | 0% |
| Indonesia | 0% | 0% | 0% | 0% | 0% | 0% | 0% | 0% | 0% |
| Jordan | 0% | 0% | 99% | 100% | 99% | 4% | 0% | 0% | 0% |
| Kazakhstan | 40% | 26% | 42% | 3% | 0% | 100% | 69% | 6% | 9% |
| Kenya | 0% | 0% | 0% | 0% | 5% | 0% | 0% | 0% | 0% |
| Kyrgyz Republic | 0% | 0% | 1% | 97% | 92% | 0% | 2% | 0% | 0% |
| Lesotho | 1% | 2% | 23% | 6% | 53% | 0% | 4% | 0% | 0% |
| Liberia | 3% | 100% | 98% | 6% | 75% | 16% | 0% | 1% | 0% |
| Madagascar | 0% | 0% | 0% | 0% | 0% | 0% | 0% | 0% | 0% |
| Malawi | 0% | 0% | 8% | 1% | 36% | 1% | 1% | 0% | 0% |
| Maldives | 0% | 0% | 100% | 0% | 0% | 0% | 0% | 7% | 1% |
| Mali | 0% | 0% | 0% | 0% | 0% | 0% | 0% | 0% | 0% |
| Morocco | 5% | 6% | 16% | 49% | 100% | 98% | 51% | 0% | 1% |
| Mozambique | 0% | 0% | 0% | 0% | 2% | 0% | 0% | 0% | 0% |
| Namibia | 0% | 0% | 20% | 0% | 4% | 0% | 0% | 0% | 0% |
| Nepal | 0% | 0% | 45% | 0% | 100% | 10% | 4% | 0% | 0% |
| Nicaragua | 19% | 42% | 32% | 22% | 33% | 38% | 1% | 6% | 13% |
| Niger | 0% | 0% | 3% | 0% | 1% | 0% | 0% | 0% | 0% |
| Nigeria | 0% | 0% | 0% | 0% | 0% | 0% | 0% | 0% | 0% |
| Pakistan | 0% | 0% | 0% | 0% | 0% | 0% | 0% | 0% | 0% |
| Peru | 0% | 0% | 100% | 2% | 0% | 0% | 0% | 0% | 0% |
| Philippines | 0% | 0% | 29% | 0% | 0% | 0% | 0% | 0% | 0% |
| Rwanda | 0% | 0% | 100% | 8% | 97% | 72% | 0% | 0% | 0% |
| Senegal | 0% | 0% | 45% | 0% | 3% | 0% | 0% | 0% | 0% |
| Sierra Leone | 20% | 32% | 1% | 9% | 99% | 35% | 41% | 57% | 15% |
| South Africa | 0% | 0% | 0% | 45% | 0% | 0% | 0% | 0% | 0% |
| Tajikistan | 1% | 0% | 32% | 81% | 0% | 1% | 0% | 0% | 0% |
| Tanzania | 0% | 0% | 0% | 0% | 1% | 0% | 0% | 0% | 0% |
| Timor-Leste | 0% | 0% | 1% | 8% | 2% | 0% | 1% | 1% | 0% |
| Togo | 0% | 0% | 0% | 0% | 71% | 4% | 0% | 0% | 0% |
| Turkey | 0% | 0% | 99% | 7% | 22% | 1% | 0% | 0% | 1% |
| Uganda | 0% | 0% | 10% | 0% | 32% | 0% | 0% | 0% | 0% |
| Vietnam | 16% | 6% | 94% | 84% | 68% | 16% | 34% | 1% | 9% |
| Yemen | 0% | 0% | 0% | 0% | 0% | 0% | 0% | 0% | 0% |
| Zambia | 0% | 0% | 3% | 0% | 1% | 0% | 0% | 0% | 0% |
| Zimbabwe | 0% | 0% | 0% | 0% | 0% | 0% | 0% | 0% | 0% |

**Note:** N/A denotes not application and refers that the indicator has no sufficient data to make projections

Table S14: Predictive probability of achieving universal coverage of CCI in low- and middle-income countries in 2030 by socio-demographic factors

| **Country** | **Wealth quintiles** | | | | | **Place of residence** | | **Education of women/mother** | | **Age of women/mother** | |
| --- | --- | --- | --- | --- | --- | --- | --- | --- | --- | --- | --- |
|  | **Poorest** | **Poorer** | **Middle** | **Richer** | **Richest** | **Urban** | **Rural** | **Below secondary** | **Secondary+** | **Adolescent** | **Adult** |
| Armenia | 0% | 0% | 0% | 0% | 0% | 0% | 0% | N/A | 0% | 3% | 0% |
| Bangladesh | 0% | 0% | 0% | 0% | 0% | 0% | 0% | 0% | 0% | 0% | 0% |
| Benin | 0% | 0% | 0% | 0% | 0% | 0% | 0% | 0% | 0% | 0% | 0% |
| Bolivia | 0% | 0% | 0% | 0% | 0% | 0% | 0% | 0% | 0% | 0% | 0% |
| Brazil | N/A | N/A | N/A | N/A | N/A | 63% | 38% | 46% | 22% | 36% | 74% |
| Burkina Faso | 0% | 0% | 0% | 0% | 0% | 0% | 0% | 0% | 0% | 0% | 0% |
| Burundi | 0% | 0% | 0% | 0% | 0% | 0% | 0% | 0% | 0% | 0% | 0% |
| Cambodia | 0% | 1% | 1% | 1% | 0% | 0% | 1% | 0% | 0% | 0% | 1% |
| Cameroon | 0% | 0% | 0% | 0% | 0% | 0% | 0% | 0% | 0% | 0% | 0% |
| Chad | 0% | 0% | 0% | 0% | 0% | 0% | 0% | 0% | 0% | 0% | 0% |
| Colombia | 1% | 1% | 1% | 0% | 1% | 1% | 1% | 1% | 0% | 1% | 1% |
| Comoros | 0% | 0% | 0% | 0% | 0% | 0% | 0% | 0% | 0% | 0% | 0% |
| Congo | 0% | 1% | 1% | 1% | 1% | 1% | 1% | 0% | 2% | 1% | 1% |
| Congo DR | 0% | 0% | 0% | 0% | 0% | 0% | 0% | 0% | 0% | 0% | 0% |
| Cote d'Ivoire | 0% | 0% | 0% | 0% | 0% | 0% | 0% | 0% | 0% | 0% | 0% |
| Dominican Republic | 0% | 0% | 0% | 0% | 0% | 0% | 0% | 0% | 0% | 0% | 0% |
| Egypt | 0% | 0% | 0% | 0% | 0% | 0% | 0% | 0% | 0% | 0% | 0% |
| Ethiopia | 0% | 0% | 0% | 0% | 0% | 0% | 0% | 0% | 0% | 0% | 0% |
| Gabon | 0% | 0% | 0% | 0% | 0% | 0% | 0% | 0% | 0% | 0% | 0% |
| Ghana | 0% | 0% | 0% | 0% | 0% | 0% | 0% | 0% | 0% | 0% | 0% |
| Guatemala | 0% | 0% | 0% | 0% | 0% | 0% | 0% | 0% | 0% | 0% | 0% |
| Guinea | 0% | 0% | 0% | 0% | 0% | 0% | 0% | 0% | 0% | 0% | 0% |
| Haiti | 0% | 0% | 0% | 0% | 0% | 0% | 0% | 0% | 0% | 0% | 0% |
| Honduras | 22% | 16% | 7% | 7% | 4% | 6% | 15% | 12% | 5% | 7% | 12% |
| India | 0% | 0% | 0% | 0% | 0% | 0% | 0% | 0% | 0% | 0% | 0% |
| Indonesia | 0% | 0% | 0% | 0% | 0% | 0% | 0% | 0% | 0% | 0% | 0% |
| Jordan | 0% | 0% | 0% | 0% | 0% | 0% | 0% | 0% | 0% | 0% | 0% |
| Kazakhstan | 12% | 47% | 73% | 73% | 28% | 28% | 52% | N/A | 36% | N/A | 41% |
| Kenya | 0% | 0% | 0% | 0% | 0% | 0% | 0% | 0% | 0% | 0% | 0% |
| Kyrgyz Republic | 0% | 0% | 0% | 0% | 0% | 0% | 0% | N/A | 0% | 2% | 0% |
| Lesotho | 2% | 0% | 2% | 2% | 0% | 0% | 1% | 1% | 1% | 1% | 1% |
| Liberia | 3% | 10% | 3% | 1% | 0% | 1% | 4% | 2% | 2% | 2% | 3% |
| Madagascar | 0% | 0% | 0% | 0% | 0% | 0% | 0% | 0% | 0% | 0% | 0% |
| Malawi | 0% | 0% | 0% | 0% | 0% | 0% | 0% | 0% | 0% | 0% | 0% |
| Maldives | 0% | 0% | 0% | 0% | 0% | 0% | 0% | 0% | 0% | 0% | 0% |
| Mali | 0% | 0% | 0% | 0% | 0% | 0% | 0% | 0% | 0% | 0% | 0% |
| Morocco | 5% | 4% | 6% | 7% | 4% | 4% | 2% | 3% | 1% | 9% | 4% |
| Mozambique | 0% | 0% | 0% | 0% | 0% | 0% | 0% | 0% | 0% | 0% | 0% |
| Namibia | 0% | 0% | 0% | 0% | 0% | 0% | 0% | 0% | 0% | 0% | 0% |
| Nepal | 0% | 0% | 0% | 0% | 0% | 0% | 0% | 0% | 0% | 0% | 0% |
| Nicaragua | 12% | 29% | 35% | 11% | 17% | 20% | 19% | 20% | 19% | 30% | 14% |
| Niger | 0% | 0% | 0% | 0% | 0% | 0% | 0% | 0% | 0% | 0% | 0% |
| Nigeria | 0% | 0% | 0% | 0% | 0% | 0% | 0% | 0% | 0% | 0% | 0% |
| Pakistan | 0% | 0% | 0% | 0% | 0% | 0% | 0% | 0% | 0% | 0% | 0% |
| Peru | 0% | 0% | 0% | 0% | 0% | 0% | 0% | 0% | 0% | 0% | 0% |
| Philippines | 0% | 0% | 0% | 0% | 0% | 0% | 0% | 0% | 0% | 0% | 0% |
| Rwanda | 0% | 0% | 0% | 0% | 0% | 0% | 0% | 0% | 0% | 0% | 0% |
| Senegal | 0% | 0% | 0% | 0% | 0% | 0% | 0% | 0% | 0% | 0% | 0% |
| Sierra Leone | 28% | 17% | 15% | 25% | 17% | 19% | 20% | 14% | 23% | 48% | 19% |
| South Africa | 0% | 0% | 0% | 0% | 0% | 0% | 0% | 0% | 0% | 0% | 0% |
| Tajikistan | 0% | 1% | 1% | 2% | 1% | 1% | 0% | 1% | 0% | 0% | 1% |
| Tanzania | 0% | 0% | 0% | 0% | 0% | 0% | 0% | 0% | 0% | 0% | 0% |
| Timor-Leste | 0% | 0% | 0% | 1% | 0% | 1% | 0% | 0% | 0% | 0% | 0% |
| Togo | 0% | 0% | 0% | 0% | 0% | 0% | 0% | 0% | 0% | 0% | 0% |
| Turkey | 0% | 0% | 0% | 0% | 0% | 0% | 0% | 0% | 0% | 0% | 0% |
| Uganda | 0% | 0% | 0% | 0% | 0% | 0% | 0% | 0% | 0% | 0% | 0% |
| Vietnam | 14% | 12% | 25% | 26% | 17% | 10% | 17% | 13% | 20% | 0% | 19% |
| Yemen | N/A | N/A | N/A | N/A | N/A | 0% | 0% | 0% | 0% | 0% | 0% |
| Zambia | 0% | 0% | 0% | 0% | 0% | 0% | 0% | 0% | 0% | 0% | 0% |
| Zimbabwe | 0% | 0% | 0% | 0% | 0% | 0% | 0% | 0% | 0% | 0% | 0% |

**Note:** N/A denotes not application and refers that the indicator has no sufficient data to make projections

Table S15: Predictive probability of achieving universal coverage of demand for family planning satisfied with modern contraceptive method among women 15-49 years of age in low- and middle-income countries in 2030 by socio-demographic factors

| **Country** | **Wealth quintiles** | | | | | **Place of residence** | | **Education of women** | | **Age of women** | |
| --- | --- | --- | --- | --- | --- | --- | --- | --- | --- | --- | --- |
|  | **Poorest** | **Poorer** | **Middle** | **Richer** | **Richest** | **Urban** | **Rural** | **Below secondary** | **Secondary+** | **Adolescent** | **Adult** |
| Albania | 0% | 0% | 0% | 0% | 0% | 0% | 0% | 0% | 0% | 0% | 0% |
| Armenia | 0% | 0% | 0% | 0% | 0% | 0% | 0% | 0% | 0% | 0% | 0% |
| Bangladesh | 0% | 0% | 0% | 0% | 0% | 0% | 0% | 0% | 0% | 0% | 0% |
| Benin | 0% | 0% | 0% | 0% | 0% | 0% | 0% | 0% | 0% | 0% | 0% |
| Bolivia | 18% | 0% | 0% | 0% | 0% | 0% | 0% | 0% | 0% | 0% | 0% |
| Brazil | N/A | N/A | N/A | N/A | N/A | 51% | 44% | 45% | 18% | 12% | 72% |
| Burkina Faso | 0% | 3% | 0% | 2% | 0% | 0% | 0% | 0% | 1% | 1% | 0% |
| Burundi | 0% | 0% | 0% | 0% | 0% | 0% | 0% | 0% | 0% | 0% | 0% |
| Cambodia | 0% | 0% | 0% | 0% | 0% | 0% | 0% | 0% | 0% | 0% | 0% |
| Cameroon | 0% | 0% | 0% | 0% | 0% | 0% | 0% | 0% | 0% | 5% | 0% |
| Chad | 0% | 0% | 0% | 0% | 0% | 0% | 0% | 0% | 0% | 0% | 0% |
| Colombia | 0% | 0% | 0% | 0% | 0% | 0% | 0% | 0% | 0% | 0% | 0% |
| Comoros | 0% | 0% | 0% | 0% | 0% | 0% | 0% | 0% | 0% | 0% | 0% |
| Congo | 0% | 1% | 4% | 3% | 3% | 2% | 0% | 1% | 2% | 7% | 1% |
| Congo DR | 0% | 0% | 0% | 0% | 0% | 0% | 0% | 0% | 0% | 0% | 0% |
| Cote d'Ivoire | 0% | 0% | 0% | 0% | 0% | 0% | 0% | 0% | 0% | 0% | 0% |
| Dominican Republic | 0% | 0% | 0% | 0% | 0% | 0% | 0% | 0% | 0% | 0% | 0% |
| Egypt | 0% | 0% | 0% | 0% | 0% | 0% | 0% | 0% | 0% | 0% | 0% |
| Ethiopia | 0% | 1% | 5% | 6% | 0% | 0% | 0% | 0% | 0% | 0% | 0% |
| Gabon | 0% | 0% | 0% | 0% | 0% | 0% | 0% | 0% | 0% | 0% | 0% |
| Ghana | 0% | 0% | 0% | 0% | 0% | 0% | 0% | 0% | 0% | 0% | 0% |
| Guatemala | 0% | 0% | 0% | 0% | 0% | 0% | 0% | 0% | 0% | 0% | 0% |
| Guinea | 0% | 0% | 0% | 0% | 0% | 0% | 0% | 0% | 0% | 0% | 0% |
| Guyana | 74% | 93% | 98% | 97% | 98% | 97% | 95% | 91% | 96% | 100% | 93% |
| Haiti | 0% | 0% | 0% | 0% | 0% | 0% | 0% | 0% | 0% | 0% | 0% |
| Honduras | 7% | 6% | 4% | 4% | 3% | 4% | 4% | 4% | 3% | 3% | 6% |
| India | 0% | 0% | 0% | 0% | 0% | 0% | 0% | 0% | 0% | 0% | 0% |
| Indonesia | 0% | 0% | 0% | 0% | 0% | 0% | 0% | 0% | 0% | 0% | 0% |
| Jordan | 0% | 0% | 0% | 0% | 0% | 0% | 0% | 0% | 0% | 0% | 0% |
| Kazakhstan | 13% | 7% | 61% | 34% | 37% | 33% | 22% | N/A | 25% | 6% | 30% |
| Kenya | 0% | 0% | 0% | 0% | 0% | 0% | 0% | 0% | 0% | 0% | 0% |
| Kyrgyz Republic | 0% | 0% | 0% | 0% | 0% | 0% | 0% | 100% | 0% | 0% | 0% |
| Lesotho | 10% | 3% | 8% | 2% | 1% | 1% | 3% | 1% | 2% | 2% | 3% |
| Liberia | 100% | 100% | 100% | 100% | 100% | 100% | 100% | 100% | 100% | 100% | 100% |
| Madagascar | 12% | 7% | 3% | 2% | 0% | 0% | 1% | 1% | 0% | 6% | 0% |
| Malawi | 0% | 1% | 0% | 0% | 0% | 0% | 0% | 0% | 0% | 0% | 0% |
| Maldives | 0% | 0% | 0% | 0% | 0% | 0% | 0% | 0% | 0% | 0% | 0% |
| Mali | 0% | 0% | 0% | 0% | 0% | 0% | 0% | 0% | 0% | 0% | 0% |
| Morocco | 54% | 13% | 4% | 2% | 1% | 1% | 16% | 8% | 1% | 6% | 5% |
| Mozambique | 0% | 0% | 0% | 0% | 0% | 0% | 0% | 0% | 0% | 0% | 0% |
| Namibia | 0% | 1% | 0% | 0% | 0% | 0% | 0% | 0% | 0% | 0% | 0% |
| Nepal | 0% | 0% | 0% | 0% | 0% | 0% | 0% | 0% | 0% | 0% | 0% |
| Nicaragua | 36% | 54% | 66% | 28% | 37% | 43% | 48% | 52% | 26% | 56% | 38% |
| Niger | 1% | 0% | 0% | 0% | 0% | 0% | 0% | 0% | 0% | 0% | 0% |
| Nigeria | 0% | 0% | 0% | 0% | 0% | 0% | 0% | 0% | 0% | 0% | 0% |
| Pakistan | 0% | 0% | 0% | 0% | 0% | 0% | 0% | 0% | 0% | 0% | 0% |
| Peru | 0% | 0% | 0% | 0% | 0% | 0% | 0% | 0% | 0% | 0% | 0% |
| Philippines | 0% | 0% | 0% | 0% | 0% | 0% | 0% | 0% | 0% | 0% | 0% |
| Rwanda | 0% | 0% | 0% | 0% | 0% | 0% | 0% | 0% | 0% | 0% | 0% |
| Senegal | 0% | 0% | 0% | 0% | 0% | 0% | 0% | 0% | 0% | 0% | 0% |
| Sierra Leone | 71% | 68% | 67% | 46% | 20% | 30% | 60% | 25% | 41% | 70% | 25% |
| South Africa | 0% | 0% | 0% | 0% | 0% | 0% | 0% | 0% | 0% | 0% | 0% |
| Tajikistan | 0% | 0% | 0% | 0% | 0% | 0% | 0% | 0% | 0% | 0% | 0% |
| Tanzania | 0% | 0% | 0% | 0% | 0% | 0% | 0% | 0% | 0% | 0% | 0% |
| Timor-Leste | 0% | 0% | 0% | 0% | 0% | 0% | 0% | 0% | 0% | 0% | 0% |
| Togo | 0% | 0% | 0% | 0% | 0% | 0% | 0% | 0% | 0% | 0% | 0% |
| Turkey | 0% | 0% | 0% | 0% | 0% | 0% | 0% | 0% | 0% | 0% | 0% |
| Uganda | 0% | 0% | 0% | 0% | 0% | 0% | 0% | 0% | 0% | 0% | 0% |
| Vietnam | 18% | 6% | 2% | 4% | 4% | 8% | 4% | 16% | 4% | 0% | 6% |
| Yemen | N/A | N/A | N/A | N/A | N/A | 0% | 0% | 0% | 0% | 0% | 0% |
| Zambia | 0% | 0% | 0% | 0% | 0% | 0% | 0% | 0% | 0% | 0% | 0% |
| Zimbabwe | 0% | 0% | 0% | 0% | 0% | 0% | 0% | 0% | 0% | 0% | 0% |

**Note:** N/A denotes not application and refers that the indicator has no sufficient data to make projections

Table S16: Predictive probability of achieving universal coverage of antenatal care visit among women 15-49 years of age in low- and middle-income countries in 2030 by socio-demographic factors

| **Country** | **Wealth quintiles** | | | | | **Place of residence** | | **Education of women** | | **Age of women** | |
| --- | --- | --- | --- | --- | --- | --- | --- | --- | --- | --- | --- |
|  | **Poorest** | **Poorer** | **Middle** | **Richer** | **Richest** | **Urban** | **Rural** | **Below secondary** | **Secondary+** | **Adolescent** | **Adult** |
| Afghanistan | 1% | 0% | 0% | 0% | 1% | 0% | 0% | 0% | 0% | 0% | 0% |
| Albania | 0% | 0% | 0% | 0% | 3% | 0% | 0% | 0% | 0% | 0% | 0% |
| Angola | 0% | 0% | 0% | 0% | 0% | 0% | 0% | 0% | 0% | 0% | 0% |
| Armenia | 99% | 100% | 99% | 100% | 100% | 100% | 100% | 95% | 100% | 100% | 100% |
| Bangladesh | 0% | 0% | 0% | 0% | 42% | 0% | 0% | 0% | 0% | 0% | 0% |
| Benin | 0% | 0% | 0% | 0% | 0% | 0% | 0% | 0% | 0% | 0% | 0% |
| Bolivia | 52% | 80% | 96% | 96% | 86% | 95% | 49% | 69% | 96% | 73% | 66% |
| Brazil | N/A | N/A | N/A | N/A | N/A | 98% | 87% | 81% | 4% | 97% | 99% |
| Burkina Faso | 0% | 0% | 0% | 0% | 0% | 0% | 0% | 0% | 25% | 0% | 0% |
| Burundi | 0% | 0% | 0% | 0% | 0% | 0% | 0% | 0% | 0% | 0% | 0% |
| Cambodia | 100% | 100% | 100% | 100% | 100% | 100% | 100% | 100% | 100% | 100% | 100% |
| Cameroon | 0% | 0% | 1% | 39% | 49% | 1% | 0% | 0% | 29% | 0% | 0% |
| Chad | 0% | 0% | 0% | 0% | 1% | 0% | 0% | 0% | 0% | 0% | 0% |
| Colombia | 4% | 99% | 100% | 100% | 100% | 100% | 4% | 0% | 100% | 32% | 91% |
| Comoros | 12% | 3% | 56% | 27% | 1% | 15% | 12% | 5% | 24% | 12% | 10% |
| Congo | 12% | 50% | 97% | 100% | 100% | 97% | 17% | 14% | 99% | 37% | 83% |
| Congo DR | 1% | 4% | 3% | 15% | 55% | 18% | 3% | 4% | 8% | 15% | 5% |
| Cote d'Ivoire | 3% | 2% | 44% | 61% | 67% | 56% | 4% | 9% | 34% | 25% | 12% |
| Dominican Republic | 0% | 1% | 27% | 1% | 0% | 5% | 23% | 8% | 8% | 13% | 11% |
| Egypt | 18% | 41% | 54% | 69% | 65% | 6% | 6% | 0% | 2% | 14% | 4% |
| Ethiopia | 0% | 1% | 1% | 2% | 17% | 37% | 0% | 0% | 87% | 1% | 0% |
| Gabon | 2% | 5% | 1% | 41% | 2% | 2% | 0% | 1% | 2% | 1% | 5% |
| Ghana | 0% | 17% | 61% | 100% | 95% | 37% | 0% | 0% | 4% | 4% | 1% |
| Guatemala | 53% | 63% | 70% | 92% | 99% | 86% | 67% | 50% | 79% | 63% | 77% |
| Guinea | 0% | 0% | 0% | 1% | 1% | 7% | 0% | 0% | 2% | 0% | 0% |
| Guyana | 91% | 64% | 92% | 25% | 100% | 100% | 84% | 44% | 97% | 3% | 92% |
| Haiti | 0% | 0% | 1% | 1% | 91% | 0% | 0% | 0% | 6% | 0% | 0% |
| Honduras | 86% | 85% | 96% | 90% | 98% | 87% | 92% | 80% | 94% | 97% | 90% |
| India | 0% | 0% | 0% | 2% | 4% | 0% | 0% | 0% | 0% | 0% | 0% |
| Indonesia | 0% | 0% | 0% | 0% | 0% | 0% | 0% | 0% | 0% | 0% | 0% |
| Jordan | 84% | 100% | 100% | 100% | 100% | 97% | 100% | 57% | 98% | 98% | 98% |
| Kazakhstan | 92% | 25% | 83% | 4% | 0% | 18% | 75% | N/A | 37% | 100% | 34% |
| Kenya | 0% | 0% | 0% | 0% | 0% | 0% | 0% | 0% | 0% | 0% | 0% |
| Kyrgyz Republic | 0% | 1% | 0% | 2% | 41% | 34% | 0% | 100% | 1% | 23% | 1% |
| Lesotho | 13% | 3% | 8% | 98% | 55% | 10% | 19% | 5% | 63% | 52% | 22% |
| Liberia | 70% | 100% | 99% | 71% | 100% | 31% | 96% | 97% | 91% | 99% | 98% |
| Madagascar | 0% | 0% | 3% | 43% | 75% | 56% | 0% | 0% | 76% | 0% | 0% |
| Malawi | 4% | 58% | 25% | 0% | 46% | 16% | 6% | 6% | 30% | 37% | 11% |
| Maldives | 100% | 100% | 100% | 99% | 100% | 100% | 100% | 100% | 100% | 100% | 100% |
| Mali | 0% | 0% | 0% | 0% | 10% | 4% | 0% | 0% | 0% | 0% | 0% |
| Morocco | 17% | 26% | 30% | 82% | 89% | 31% | 5% | 12% | 13% | 8% | 13% |
| Mozambique | 0% | 0% | 0% | 0% | 0% | 0% | 0% | 0% | 0% | 0% | 0% |
| Namibia | 9% | 8% | 87% | 84% | 66% | 54% | 18% | 0% | 78% | 7% | 34% |
| Nepal | 9% | 49% | 80% | 97% | 99% | 22% | 27% | 11% | 47% | 76% | 42% |
| Nicaragua | 11% | 38% | 72% | 52% | 43% | 57% | 25% | 25% | 60% | 62% | 27% |
| Niger | 13% | 38% | 54% | 24% | 62% | 35% | 7% | 4% | 97% | 1% | 5% |
| Nigeria | 0% | 0% | 0% | 0% | 0% | 0% | 0% | 0% | 0% | 0% | 0% |
| Pakistan | 0% | 0% | 13% | 55% | 99% | 4% | 0% | 0% | 47% | 0% | 0% |
| Peru | 98% | 100% | 100% | 100% | 100% | 100% | 100% | 99% | 100% | 63% | 100% |
| Philippines | 1% | 84% | 86% | 100% | 99% | 25% | 34% | 0% | 75% | 35% | 29% |
| Rwanda | 90% | 100% | 100% | 100% | 100% | 100% | 100% | 100% | 93% | 100% | 99% |
| Senegal | 28% | 87% | 99% | 100% | 100% | 97% | 22% | 36% | 67% | 29% | 46% |
| Sierra Leone | 1% | 0% | 0% | 2% | 5% | 2% | 0% | 0% | 0% | 0% | 1% |
| South Africa | 0% | 0% | 0% | 1% | 0% | 0% | 2% | 0% | 0% | 0% | 0% |
| Tajikistan | 35% | 31% | 30% | 18% | 72% | 91% | 23% | 4% | 43% | 0% | 36% |
| Tanzania | 0% | 0% | 0% | 0% | 0% | 0% | 0% | 0% | 0% | 0% | 0% |
| Timor-Leste | 1% | 1% | 0% | 6% | 2% | 6% | 1% | 1% | 0% | 0% | 2% |
| Togo | 0% | 0% | 0% | 0% | 1% | 0% | 0% | 0% | 0% | 0% | 0% |
| Turkey | 75% | 100% | 100% | 100% | 100% | 100% | 61% | 92% | 99% | 97% | 99% |
| Uganda | 4% | 22% | 34% | 4% | 9% | 37% | 6% | 3% | 6% | 2% | 12% |
| Vietnam | 77% | 88% | 98% | 97% | 100% | 99% | 95% | 73% | 98% | 9% | 94% |
| Yemen | N/A | N/A | N/A | N/A | N/A | 0% | 0% | 0% | 0% | 0% | 0% |
| Zambia | 1% | 2% | 9% | 28% | 47% | 26% | 1% | 1% | 2% | 18% | 2% |
| Zimbabwe | 0% | 0% | 0% | 0% | 2% | 0% | 0% | 0% | 0% | 0% | 0% |

**Note:** N/A denotes not application and refers that the indicator has no sufficient data to make projections

Table S17: Predictive probability of achieving universal coverage of skilled birth attendance among women 15-49 years of age in low- and middle-income countries in 2030 by socio-demographic factors

| **Country** | **Wealth quintiles** | | | | | **Place of residence** | | **Education of women** | | **Age of women** | |
| --- | --- | --- | --- | --- | --- | --- | --- | --- | --- | --- | --- |
|  | **Poorest** | **Poorer** | **Middle** | **Richer** | **Richest** | **Urban** | **Rural** | **Below secondary** | **Secondary+** | **Adolescent** | **Adult** |
| Afghanistan | 1% | 1% | 0% | 5% | 15% | 2% | 1% | 0% | 2% | 0% | 1% |
| Albania | 0% | 0% | 0% | 0% | 0% | 0% | 0% | 0% | 0% | 0% | 0% |
| Angola | 0% | 0% | 0% | 0% | 0% | 0% | 0% | 0% | 0% | 0% | 0% |
| Armenia | 100% | 100% | 100% | 100% | 0% | 100% | 100% | 100% | 100% | 100% | 100% |
| Bangladesh | 0% | 0% | 0% | 0% | 0% | 0% | 0% | 0% | 0% | 0% | 0% |
| Benin | 0% | 0% | 0% | 19% | 25% | 2% | 0% | 0% | 34% | 0% | 0% |
| Bolivia | 0% | 1% | 24% | 61% | 98% | 29% | 0% | 0% | 25% | 2% | 1% |
| Brazil | N/A | N/A | N/A | N/A | N/A | 96% | 82% | 80% | 82% | 76% | 98% |
| Burkina Faso | 0% | 0% | 0% | 0% | 0% | 0% | 0% | 0% | 0% | 0% | 0% |
| Burundi | 15% | 56% | 62% | 54% | 97% | 93% | 41% | 36% | 67% | 54% | 46% |
| Cambodia | 51% | 95% | 99% | 100% | 100% | 100% | 86% | 77% | 100% | 94% | 91% |
| Cameroon | 0% | 0% | 0% | 0% | 0% | 0% | 0% | 0% | 0% | 0% | 0% |
| Chad | 0% | 0% | 0% | 0% | 0% | 0% | 0% | 0% | 0% | 0% | 0% |
| Colombia | 0% | 100% | 100% | 68% | 94% | 100% | 0% | 0% | 38% | 18% | 4% |
| Comoros | 1% | 13% | 80% | 96% | 80% | 85% | 6% | 4% | 83% | 14% | 11% |
| Congo | 1% | 64% | 98% | 56% | 81% | 30% | 5% | 6% | 74% | 15% | 29% |
| Congo DR | 1% | 0% | 0% | 0% | 1% | 0% | 0% | 0% | 0% | 1% | 0% |
| Cote d'Ivoire | 0% | 0% | 2% | 21% | 30% | 11% | 0% | 0% | 1% | 0% | 0% |
| Dominican Republic | 45% | 95% | 7% | 100% | 100% | 92% | 95% | 84% | 92% | 100% | 98% |
| Egypt | 7% | 2% | 97% | 100% | 100% | 98% | 21% | 0% | 14% | 31% | 21% |
| Ethiopia | 0% | 0% | 0% | 0% | 0% | 3% | 0% | 0% | 0% | 0% | 0% |
| Gabon | 0% | 1% | 0% | 3% | 0% | 0% | 0% | 0% | 0% | 1% | 0% |
| Ghana | 0% | 0% | 0% | 5% | 11% | 0% | 0% | 0% | 0% | 0% | 0% |
| Guatemala | 90% | 98% | 100% | 100% | 100% | 100% | 95% | 92% | 98% | 98% | 95% |
| Guinea | 0% | 0% | 0% | 0% | 4% | 0% | 0% | 0% | 0% | 0% | 0% |
| Haiti | 0% | 0% | 0% | 0% | 0% | 0% | 0% | 0% | 0% | 0% | 0% |
| Honduras | 100% | 100% | 100% | 100% | 98% | 100% | 100% | 100% | 99% | 100% | 100% |
| India | 0% | 0% | 2% | 8% | 10% | 1% | 0% | 0% | 0% | 1% | 0% |
| Indonesia | 0% | 0% | 0% | 0% | 0% | 0% | 0% | 0% | 0% | 0% | 0% |
| Jordan | 100% | 100% | 100% | 100% | 100% | 100% | 100% | 100% | 100% | 100% | 100% |
| Kazakhstan | 1% | 0% | 43% | 93% | 0% | 0% | 14% | 92% | 2% | 93% | 3% |
| Kenya | 0% | 0% | 0% | 0% | 1% | 0% | 0% | 0% | 0% | 0% | 0% |
| Kyrgyz Republic | 95% | 100% | 95% | 1% | 8% | 99% | 95% | 100% | 97% | 100% | 98% |
| Lesotho | 3% | 5% | 11% | 32% | 70% | 0% | 6% | 1% | 17% | 23% | 6% |
| Liberia | 3% | 13% | 7% | 4% | 28% | 0% | 6% | 1% | 3% | 15% | 4% |
| Madagascar | 0% | 0% | 0% | 0% | 1% | 60% | 0% | 0% | 39% | 0% | 0% |
| Malawi | 1% | 0% | 0% | 2% | 5% | 1% | 0% | 0% | 0% | 4% | 0% |
| Maldives | 0% | 0% | 0% | 0% | 3% | 2% | 0% | 0% | 0% | 0% | 0% |
| Mali | 0% | 0% | 0% | 0% | 0% | 1% | 0% | 0% | 0% | 0% | 0% |
| Morocco | 58% | 44% | 94% | 98% | 100% | 94% | 10% | 32% | 99% | 26% | 51% |
| Mozambique | 0% | 0% | 0% | 0% | 0% | 0% | 0% | 0% | 0% | 0% | 0% |
| Namibia | 0% | 2% | 0% | 69% | 100% | 13% | 0% | 0% | 2% | 0% | 0% |
| Nepal | 0% | 0% | 1% | 2% | 44% | 0% | 0% | 0% | 0% | 0% | 0% |
| Nicaragua | 5% | 23% | 66% | 39% | 100% | 76% | 16% | 12% | 76% | 16% | 24% |
| Niger | 0% | 0% | 0% | 0% | 0% | 0% | 0% | 0% | 0% | 0% | 0% |
| Nigeria | 0% | 0% | 0% | 0% | 0% | 0% | 0% | 0% | 0% | 0% | 0% |
| Pakistan | 0% | 0% | 0% | 0% | 0% | 0% | 0% | 0% | 0% | 0% | 0% |
| Peru | 0% | 8% | 100% | 100% | 100% | 98% | 0% | 0% | 57% | 4% | 2% |
| Philippines | 0% | 0% | 6% | 85% | 79% | 1% | 0% | 0% | 0% | 1% | 0% |
| Rwanda | 1% | 10% | 12% | 20% | 79% | 14% | 8% | 8% | 57% | 85% | 7% |
| Senegal | 0% | 0% | 0% | 0% | 0% | 0% | 0% | 0% | 0% | 0% | 0% |
| Sierra Leone | 11% | 7% | 5% | 14% | 35% | 16% | 12% | 7% | 5% | 24% | 7% |
| South Africa | 5% | 78% | 86% | 38% | 10% | 12% | 33% | 3% | 22% | 12% | 48% |
| Tajikistan | 82% | 53% | 77% | 94% | 85% | 92% | 81% | 14% | 88% | 9% | 82% |
| Tanzania | 0% | 0% | 0% | 0% | 1% | 0% | 0% | 0% | 0% | 0% | 0% |
| Timor-Leste | 2% | 6% | 26% | 56% | 88% | 81% | 5% | 1% | 10% | 19% | 8% |
| Togo | 0% | 0% | 0% | 0% | 0% | 0% | 0% | 0% | 0% | 0% | 0% |
| Turkey | 3% | 4% | 27% | 45% | 99% | 6% | 5% | 4% | 22% | 1% | 6% |
| Uganda | 0% | 0% | 0% | 0% | 35% | 5% | 0% | 0% | 1% | 0% | 0% |
| Vietnam | 27% | 80% | 97% | 100% | 100% | 100% | 82% | 50% | 96% | 95% | 84% |
| Yemen | N/A | N/A | N/A | N/A | N/A | 0% | 0% | 0% | 0% | 0% | 0% |
| Zambia | 0% | 0% | 0% | 0% | 1% | 0% | 0% | 0% | 0% | 0% | 0% |
| Zimbabwe | 0% | 0% | 0% | 0% | 4% | 0% | 0% | 0% | 0% | 0% | 0% |

**Note:** N/A denotes not application and refers that the indicator has no sufficient data to make projections

Table S18: Predictive probability of achieving universal coverage of BCG immunization among children 12-23 months of age in low- and middle-income countries in 2030 by socio-demographic factors

| **Country** | **Wealth quintiles** | | | | | **Place of residence** | | **Education of mother** | | **Age of mother** | | **Sex of child** | |
| --- | --- | --- | --- | --- | --- | --- | --- | --- | --- | --- | --- | --- | --- |
|  | **Poorest** | **Poorer** | **Middle** | **Richer** | **Richest** | **Urban** | **Rural** | **Below secondary** | **Secondary+** | **Adolescent** | **Adult** | **Male** | **Female** |
| Albania | 100% | 100% | 100% | 100% | 100% | 100% | 100% | 100% | 100% | 100% | 100% | 100% | 100% |
| Armenia | 100% | 100% | 91% | 100% | 100% | 100% | 98% | 100% | 99% | 100% | 99% | 100% | 100% |
| Bangladesh | 88% | 98% | 100% | 98% | 100% | 100% | 99% | 74% | 100% | 100% | 100% | 100% | 100% |
| Benin | 0% | 0% | 0% | 0% | 1% | 0% | 0% | 0% | 0% | 0% | 0% | 0% | 0% |
| Bolivia | 100% | 100% | 100% | 100% | 95% | 100% | 100% | 100% | 100% | 100% | 100% | 100% | 100% |
| Brazil | N/A | N/A | N/A | N/A | N/A | 100% | 95% | 98% | 55% | 97% | 100% | 100% | 99% |
| Burkina Faso | 18% | 46% | 95% | 100% | 71% | 96% | 65% | 65% | 0% | 23% | 70% | 83% | 50% |
| Burundi | 0% | 5% | 22% | 0% | 1% | 0% | 2% | 1% | 0% | 100% | 2% | 1% | 2% |
| Cambodia | 68% | 64% | 100% | 100% | 99% | 100% | 93% | 91% | 98% | 100% | 95% | 95% | 97% |
| Cameroon | 0% | 6% | 33% | 4% | 99% | 17% | 0% | 0% | 84% | 1% | 0% | 0% | 1% |
| Chad | 0% | 0% | 0% | 0% | 0% | 0% | 0% | 0% | 0% | 0% | 0% | 0% | 0% |
| Colombia | 13% | 69% | 86% | 67% | 82% | 74% | 11% | 13% | 76% | 0% | 71% | 45% | 51% |
| Comoros | 0% | 0% | 0% | 0% | 0% | 0% | 0% | 0% | 0% | 0% | 0% | 0% | 0% |
| Congo | 25% | 47% | 14% | 89% | 0% | 15% | 12% | 11% | 72% | 0% | 39% | 62% | 16% |
| Congo DR | 2% | 15% | 17% | 8% | 17% | 29% | 10% | 8% | 13% | 58% | 9% | 7% | 11% |
| Cote d'Ivoire | 0% | 0% | 0% | 35% | 39% | 2% | 0% | 0% | 4% | 0% | 0% | 0% | 0% |
| Dominican Republic | 1% | 92% | 17% | 0% | 18% | 18% | 62% | 3% | 89% | 91% | 25% | 39% | 29% |
| Egypt | 68% | 100% | 98% | 88% | 18% | 100% | 100% | 100% | 99% | 77% | 100% | 100% | 100% |
| Ethiopia | 0% | 0% | 0% | 0% | 0% | 2% | 0% | 0% | 74% | 0% | 0% | 0% | 0% |
| Gabon | 8% | 1% | 0% | 1% | 13% | 1% | 1% | 1% | 2% | 0% | 14% | 2% | 2% |
| Ghana | 67% | 78% | 97% | 100% | 97% | 70% | 88% | 28% | 0% | 77% | 89% | 86% | 89% |
| Guatemala | 100% | 85% | 100% | 100% | 39% | 96% | 98% | 100% | 60% | 100% | 96% | 90% | 99% |
| Guinea | 0% | 0% | 0% | 0% | 1% | 2% | 0% | 0% | 0% | 1% | 0% | 1% | 0% |
| Haiti | 0% | 0% | 0% | 0% | 1% | 0% | 0% | 0% | 0% | 0% | 0% | 0% | 0% |
| Honduras | 95% | 86% | 90% | 61% | 96% | 68% | 98% | 88% | 95% | 100% | 87% | 48% | 99% |
| India | 0% | 2% | 6% | 12% | 13% | 2% | 2% | 0% | 1% | 2% | 1% | 1% | 2% |
| Indonesia | 0% | 0% | 0% | 1% | 0% | 0% | 0% | 0% | 0% | 0% | 0% | 0% | 0% |
| Jordan | 98% | 100% | 100% | 99% | 96% | 99% | 100% | 33% | 100% | 100% | 99% | 97% | 100% |
| Kazakhstan | 0% | 5% | 1% | 0% | 0% | 0% | 3% | N/A | 0% | 0% | 0% | 0% | 1% |
| Kenya | 0% | 41% | 28% | 8% | 26% | 13% | 3% | 2% | 43% | 14% | 6% | 5% | 7% |
| Kyrgyz Republic | 100% | 100% | 72% | 52% | 49% | 97% | 89% | 100% | 92% | 100% | 92% | 98% | 81% |
| Lesotho | 48% | 0% | 81% | 99% | 100% | 95% | 40% | 15% | 77% | 89% | 46% | 98% | 4% |
| Liberia | 32% | 100% | 94% | 11% | 94% | 48% | 58% | 70% | 73% | 99% | 73% | 74% | 79% |
| Madagascar | 0% | 5% | 62% | 60% | 56% | 33% | 0% | 0% | 93% | 0% | 1% | 0% | 1% |
| Malawi | 22% | 25% | 48% | 70% | 46% | 88% | 28% | 21% | 85% | 59% | 33% | 25% | 47% |
| Maldives | 0% | 0% | 0% | 26% | 0% | 0% | 0% | 0% | 0% | 0% | 0% | 0% | 0% |
| Mali | 0% | 0% | 0% | 3% | 1% | 4% | 0% | 0% | 0% | 0% | 0% | 0% | 0% |
| Morocco | 100% | 93% | 100% | 100% | 85% | 99% | 100% | 100% | 0% | 100% | 100% | 99% | 100% |
| Mozambique | 1% | 1% | 32% | 1% | 2% | 0% | 1% | 1% | 1% | 0% | 2% | 1% | 8% |
| Namibia | 22% | 10% | 0% | 28% | 39% | 3% | 33% | 2% | 8% | 87% | 3% | 25% | 1% |
| Nepal | 100% | 94% | 88% | 100% | 100% | 99% | 96% | 95% | 75% | 100% | 99% | 100% | 93% |
| Nicaragua | 14% | 77% | 60% | 1% | 81% | 90% | 14% | 32% | 21% | 73% | 26% | 22% | 46% |
| Niger | 6% | 24% | 53% | 6% | 5% | 6% | 3% | 1% | 86% | 8% | 2% | 3% | 2% |
| Nigeria | 0% | 0% | 0% | 1% | 2% | 0% | 0% | 0% | 0% | 0% | 0% | 0% | 0% |
| Pakistan | 0% | 0% | 0% | 2% | 58% | 0% | 0% | 0% | 45% | 0% | 0% | 0% | 0% |
| Peru | 0% | 0% | 1% | 0% | 34% | 1% | 0% | 0% | 0% | 2% | 0% | 1% | 0% |
| Philippines | 0% | 0% | 0% | 3% | 20% | 0% | 0% | 0% | 1% | 0% | 0% | 0% | 0% |
| Rwanda | 49% | 99% | 93% | 100% | 100% | 100% | 95% | 95% | 100% | 100% | 97% | 99% | 90% |
| Senegal | 0% | 5% | 56% | 98% | 100% | 97% | 1% | 1% | 100% | 0% | 10% | 3% | 6% |
| Sierra Leone | 99% | 95% | 100% | 100% | 74% | 78% | 99% | 98% | 96% | 100% | 98% | 95% | 99% |
| South Africa | 0% | 0% | 0% | 0% | 0% | 0% | 0% | 0% | 0% | 0% | 0% | 0% | 0% |
| Tajikistan | 0% | 2% | 0% | 1% | 0% | 0% | 0% | 2% | 1% | 0% | 1% | 0% | 0% |
| Tanzania | 1% | 0% | 13% | 3% | 0% | 1% | 0% | 1% | 0% | 2% | 1% | 6% | 0% |
| Timor-Leste | 1% | 1% | 0% | 3% | 10% | 2% | 2% | 1% | 1% | 0% | 2% | 2% | 2% |
| Togo | 74% | 11% | 17% | 100% | 96% | 95% | 38% | 42% | 94% | 88% | 71% | 39% | 88% |
| Turkey | 43% | 3% | 15% | 99% | 1% | 9% | 48% | 6% | 1% | 99% | 25% | 58% | 6% |
| Uganda | 57% | 80% | 26% | 4% | 25% | 21% | 30% | 24% | 14% | 47% | 34% | 28% | 42% |
| Vietnam | 18% | 99% | 4% | 87% | 100% | 100% | 55% | 14% | 97% | 0% | 74% | 90% | 23% |
| Yemen | N/A | N/A | N/A | N/A | N/A | 0% | 0% | 0% | 0% | 0% | 0% | 0% | 0% |
| Zambia | 0% | 0% | 0% | 0% | 0% | 2% | 1% | 1% | 0% | 15% | 0% | 1% | 1% |
| Zimbabwe | 0% | 0% | 0% | 0% | 0% | 0% | 0% | 0% | 0% | 0% | 0% | 0% | 0% |

**Note:** N/A denotes not application and refers that the indicator has no sufficient data to make projections

Table S19: Predictive probability of achieving universal coverage of three doses of DPT immunization among children 12-23 months of age in low- and middle-income countries in 2030 by socio-demographic factors

| **Country** | **Wealth quintiles** | | | | | **Place of residence** | | **Education of mother** | | **Age of mother** | | **Sex of child** | |
| --- | --- | --- | --- | --- | --- | --- | --- | --- | --- | --- | --- | --- | --- |
|  | **Poorest** | **Poorer** | **Middle** | **Richer** | **Richest** | **Urban** | **Rural** | **Below secondary** | **Secondary+** | **Adolescent** | **Adult** | **Male** | **Female** |
| Armenia | 57% | 1% | 3% | 0% | 39% | 4% | 4% | 0% | 4% | 100% | 3% | 6% | 2% |
| Bangladesh | 8% | 30% | 32% | 79% | 99% | 19% | 25% | 1% | 64% | 15% | 35% | 28% | 43% |
| Benin | 0% | 0% | 0% | 0% | 0% | 0% | 0% | 0% | 0% | 0% | 0% | 0% | 0% |
| Bolivia | 90% | 86% | 85% | 57% | 8% | 33% | 94% | 72% | 83% | 91% | 73% | 65% | 84% |
| Brazil | N/A | N/A | N/A | N/A | N/A | 95% | 20% | 84% | 19% | 30% | 92% | 94% | 78% |
| Burkina Faso | 66% | 62% | 91% | 83% | 52% | 16% | 75% | 69% | 1% | 59% | 68% | 75% | 57% |
| Burundi | 19% | 62% | 48% | 7% | 9% | 11% | 23% | 20% | 42% | 2% | 25% | 19% | 27% |
| Cambodia | 1% | 12% | 22% | 94% | 98% | 94% | 11% | 7% | 68% | 8% | 18% | 15% | 24% |
| Cameroon | 0% | 0% | 0% | 0% | 0% | 0% | 0% | 0% | 0% | 0% | 0% | 0% | 0% |
| Chad | 0% | 0% | 0% | 0% | 0% | 0% | 0% | 0% | 0% | 0% | 0% | 0% | 0% |
| Colombia | 0% | 2% | 3% | 0% | 0% | 1% | 0% | 0% | 0% | 1% | 1% | 1% | 0% |
| Comoros | 0% | 0% | 0% | 0% | 0% | 0% | 0% | 0% | 0% | 0% | 0% | 0% | 0% |
| Congo | 0% | 0% | 0% | 0% | 0% | 0% | 0% | 0% | 0% | 0% | 0% | 0% | 0% |
| Congo DR | 2% | 1% | 0% | 3% | 7% | 4% | 1% | 1% | 1% | 3% | 2% | 1% | 2% |
| Cote d'Ivoire | 0% | 0% | 0% | 0% | 1% | 0% | 0% | 0% | 1% | 0% | 0% | 0% | 0% |
| Dominican Republic | 1% | 14% | 0% | 0% | 53% | 0% | 3% | 0% | 0% | 0% | 0% | 0% | 0% |
| Egypt | 70% | 84% | 94% | 82% | 98% | 100% | 98% | 81% | 99% | 94% | 99% | 98% | 99% |
| Ethiopia | 0% | 0% | 0% | 0% | 0% | 0% | 0% | 0% | 0% | 0% | 0% | 0% | 0% |
| Gabon | 0% | 0% | 0% | 0% | 0% | 0% | 0% | 0% | 0% | 0% | 0% | 0% | 0% |
| Ghana | 14% | 1% | 3% | 28% | 1% | 0% | 10% | 0% | 0% | 1% | 4% | 2% | 3% |
| Guatemala | 0% | 0% | 1% | 1% | 1% | 1% | 0% | 0% | 2% | 0% | 0% | 0% | 0% |
| Guinea | 0% | 0% | 0% | 0% | 0% | 0% | 0% | 0% | 0% | 0% | 0% | 0% | 0% |
| Haiti | 0% | 0% | 0% | 0% | 0% | 0% | 0% | 0% | 0% | 0% | 0% | 0% | 0% |
| Honduras | 32% | 31% | 6% | 32% | 100% | 23% | 68% | 28% | 67% | 30% | 40% | 52% | 29% |
| India | 0% | 0% | 0% | 0% | 0% | 0% | 0% | 0% | 0% | 0% | 0% | 0% | 0% |
| Indonesia | 0% | 0% | 0% | 0% | 0% | 0% | 0% | 0% | 0% | 0% | 0% | 0% | 0% |
| Jordan | 0% | 14% | 23% | 8% | 18% | 4% | 1% | 0% | 13% | 100% | 2% | 2% | 4% |
| Kazakhstan | 100% | 100% | 100% | 100% | 98% | 100% | 100% | N/A | 100% | 92% | 100% | 100% | 100% |
| Kenya | 0% | 0% | 0% | 1% | 0% | 0% | 0% | 0% | 0% | 1% | 0% | 0% | 0% |
| Kyrgyz Republic | 11% | 4% | 1% | 0% | 0% | 0% | 1% | N/A | 0% | 91% | 0% | 0% | 0% |
| Lesotho | 0% | 0% | 29% | 5% | 0% | 1% | 1% | 0% | 0% | 0% | 0% | 0% | 0% |
| Liberia | 21% | 65% | 15% | 12% | 6% | 4% | 21% | 13% | 12% | 6% | 20% | 12% | 25% |
| Madagascar | 0% | 2% | 16% | 19% | 40% | 16% | 0% | 0% | 41% | 0% | 0% | 0% | 1% |
| Malawi | 1% | 0% | 1% | 3% | 0% | 0% | 1% | 1% | 0% | 1% | 1% | 0% | 2% |
| Maldives | 0% | 0% | 0% | 0% | 0% | 0% | 0% | 0% | 0% | 0% | 0% | 0% | 0% |
| Mali | 0% | 1% | 1% | 0% | 0% | 0% | 0% | 0% | 0% | 0% | 0% | 0% | 0% |
| Morocco | 93% | 99% | 98% | 96% | 86% | 88% | 96% | 97% | 70% | 100% | 98% | 96% | 98% |
| Mozambique | 0% | 0% | 1% | 0% | 0% | 0% | 0% | 0% | 0% | 0% | 0% | 0% | 0% |
| Namibia | 0% | 0% | 0% | 2% | 1% | 0% | 0% | 0% | 0% | 0% | 0% | 0% | 0% |
| Nepal | 9% | 10% | 10% | 51% | 36% | 3% | 11% | 3% | 20% | 12% | 12% | 10% | 12% |
| Nicaragua | 22% | 82% | 41% | 4% | 82% | 47% | 34% | 40% | 37% | 84% | 30% | 29% | 44% |
| Niger | 3% | 7% | 21% | 4% | 2% | 0% | 0% | 0% | 0% | 0% | 0% | 0% | 0% |
| Nigeria | 0% | 0% | 1% | 10% | 5% | 0% | 0% | 0% | 0% | 0% | 0% | 0% | 0% |
| Pakistan | 0% | 0% | 0% | 0% | 0% | 0% | 0% | 0% | 0% | 0% | 0% | 0% | 0% |
| Peru | 0% | 0% | 0% | 0% | 0% | 0% | 0% | 0% | 0% | 0% | 0% | 0% | 0% |
| Philippines | 0% | 0% | 0% | 0% | 0% | 0% | 0% | 0% | 0% | 0% | 0% | 0% | 0% |
| Rwanda | 11% | 75% | 80% | 100% | 90% | 66% | 73% | 69% | 92% | 100% | 71% | 81% | 59% |
| Senegal | 0% | 1% | 3% | 58% | 100% | 1% | 0% | 0% | 75% | 0% | 0% | 0% | 0% |
| Sierra Leone | 66% | 34% | 36% | 68% | 2% | 9% | 50% | 34% | 11% | 32% | 35% | 32% | 37% |
| South Africa | 0% | 0% | 0% | 0% | 0% | 0% | 0% | 0% | 0% | 0% | 0% | 0% | 0% |
| Tajikistan | 1% | 1% | 4% | 2% | 0% | 0% | 3% | 16% | 1% | 0% | 2% | 0% | 3% |
| Tanzania | 0% | 0% | 1% | 0% | 6% | 5% | 0% | 0% | 1% | 0% | 0% | 0% | 0% |
| Timor-Leste | 0% | 0% | 0% | 0% | 1% | 0% | 0% | 0% | 0% | 0% | 0% | 0% | 0% |
| Togo | 24% | 1% | 1% | 3% | 11% | 2% | 4% | 3% | 3% | 8% | 3% | 7% | 3% |
| Turkey | 0% | 2% | 3% | 11% | 1% | 1% | 0% | 0% | 0% | 1% | 2% | 3% | 0% |
| Uganda | 0% | 0% | 0% | 0% | 0% | 0% | 0% | 0% | 0% | 0% | 0% | 0% | 0% |
| Vietnam | 3% | 4% | 66% | 77% | 98% | 88% | 13% | 5% | 30% | 20% | 19% | 23% | 13% |
| Yemen | N/A | N/A | N/A | N/A | N/A | 0% | 0% | 0% | 0% | 0% | 0% | 0% | 0% |
| Zambia | 0% | 0% | 0% | 0% | 0% | 0% | 0% | 0% | 0% | 0% | 0% | 0% | 0% |
| Zimbabwe | 0% | 0% | 0% | 0% | 0% | 0% | 0% | 0% | 0% | 0% | 0% | 0% | 0% |

**Note:** N/A denotes not application and refers that the indicator has no sufficient data to make projections

Table S20: Predictive probability of achieving universal coverage of Measles immunization among children 12-23 months of age in low- and middle-income countries in 2030 by socio-demographic factors

| **Country** | **Wealth quintiles** | | | | | **Place of residence** | | **Education of mother** | | **Age of mother** | | **Sex of child** | |
| --- | --- | --- | --- | --- | --- | --- | --- | --- | --- | --- | --- | --- | --- |
|  | **Poorest** | **Poorer** | **Middle** | **Richer** | **Richest** | **Urban** | **Rural** | **Below secondary** | **Secondary+** | **Adolescent** | **Adult** | **Male** | **Female** |
| Albania | 100% | 100% | 100% | 100% | 100% | 100% | 100% | 100% | 100% | 100% | 100% | 100% | 100% |
| Armenia | 14% | 0% | 1% | 1% | 0% | 2% | 0% | 0% | 0% | 100% | 0% | 0% | 2% |
| Bangladesh | 0% | 0% | 0% | 0% | 1% | 0% | 0% | 0% | 0% | 0% | 0% | 0% | 0% |
| Benin | 0% | 0% | 0% | 0% | 0% | 0% | 0% | 0% | 0% | 0% | 0% | 0% | 0% |
| Bolivia | 4% | 2% | 0% | 0% | 0% | 0% | 4% | 2% | 0% | 2% | 0% | 0% | 1% |
| Brazil | N/A | N/A | N/A | N/A | N/A | 91% | 22% | 66% | 12% | 11% | 88% | 93% | 48% |
| Burkina Faso | 4% | 8% | 35% | 42% | 23% | 8% | 14% | 13% | 11% | 26% | 13% | 16% | 14% |
| Burundi | 2% | 5% | 3% | 1% | 0% | 1% | 3% | 3% | 0% | 1% | 2% | 1% | 5% |
| Cambodia | 0% | 0% | 3% | 20% | 34% | 36% | 1% | 0% | 9% | 0% | 2% | 1% | 3% |
| Cameroon | 0% | 0% | 0% | 0% | 0% | 0% | 0% | 0% | 0% | 0% | 0% | 0% | 0% |
| Chad | 0% | 0% | 0% | 0% | 0% | 0% | 0% | 0% | 0% | 0% | 0% | 0% | 0% |
| Colombia | 0% | 0% | 0% | 1% | 0% | 0% | 0% | 0% | 0% | 0% | 0% | 0% | 0% |
| Comoros | 0% | 0% | 1% | 0% | 0% | 0% | 0% | 0% | 0% | 0% | 0% | 0% | 0% |
| Congo | 1% | 2% | 10% | 9% | 5% | 8% | 0% | 1% | 7% | 2% | 3% | 5% | 3% |
| Congo DR | 1% | 2% | 3% | 2% | 1% | 1% | 2% | 1% | 2% | 1% | 2% | 1% | 1% |
| Cote d'Ivoire | 0% | 0% | 0% | 0% | 3% | 0% | 0% | 0% | 1% | 0% | 0% | 0% | 0% |
| Dominican Republic | 3% | 2% | 0% | 0% | 1% | 0% | 0% | 0% | 0% | 0% | 0% | 2% | 0% |
| Egypt | 0% | 0% | 0% | 0% | 0% | 0% | 0% | 0% | 0% | 1% | 0% | 0% | 0% |
| Ethiopia | 0% | 0% | 0% | 0% | 0% | 0% | 0% | 0% | 2% | 0% | 0% | 0% | 0% |
| Gabon | 7% | 1% | 2% | 8% | 0% | 0% | 13% | 3% | 0% | 0% | 2% | 1% | 1% |
| Ghana | 9% | 4% | 11% | 24% | 1% | 2% | 10% | 2% | 0% | 0% | 10% | 2% | 11% |
| Guatemala | 0% | 0% | 0% | 0% | 0% | 0% | 0% | 0% | 0% | 0% | 0% | 0% | 0% |
| Guinea | 0% | 0% | 0% | 0% | 0% | 0% | 0% | 0% | 0% | 0% | 0% | 0% | 0% |
| Haiti | 0% | 0% | 0% | 0% | 0% | 0% | 0% | 0% | 0% | 0% | 0% | 0% | 0% |
| Honduras | 21% | 5% | 4% | 4% | 17% | 6% | 9% | 10% | 4% | 6% | 8% | 6% | 8% |
| India | 0% | 0% | 0% | 0% | 0% | 0% | 0% | 0% | 0% | 0% | 0% | 0% | 0% |
| Indonesia | 0% | 0% | 0% | 0% | 0% | 0% | 0% | 0% | 0% | 0% | 0% | 0% | 0% |
| Jordan | 0% | 0% | 1% | 0% | 0% | 0% | 0% | 0% | 0% | 0% | 0% | 0% | 0% |
| Kazakhstan | 65% | 96% | 16% | 84% | 34% | 75% | 64% | N/A | 67% | 0% | 83% | 84% | 44% |
| Kenya | 0% | 0% | 0% | 3% | 0% | 0% | 0% | 0% | 0% | 0% | 0% | 0% | 0% |
| Kyrgyz Republic | 22% | 0% | 1% | 0% | 4% | 1% | 1% | 0% | 1% | 88% | 1% | 2% | 0% |
| Lesotho | 4% | 0% | 89% | 5% | 7% | 2% | 4% | 1% | 13% | 2% | 4% | 1% | 21% |
| Liberia | 1% | 1% | 0% | 0% | 0% | 0% | 0% | 0% | 0% | 0% | 0% | 0% | 0% |
| Madagascar | 0% | 1% | 9% | 19% | 16% | 5% | 0% | 0% | 15% | 0% | 0% | 0% | 0% |
| Malawi | 1% | 2% | 0% | 3% | 2% | 1% | 1% | 0% | 0% | 0% | 1% | 0% | 1% |
| Maldives | 0% | 0% | 0% | 1% | 0% | 0% | 0% | 0% | 0% | 0% | 0% | 0% | 0% |
| Mali | 0% | 0% | 0% | 0% | 0% | 0% | 0% | 0% | 0% | 0% | 0% | 0% | 0% |
| Morocco | 36% | 27% | 19% | 91% | 96% | 33% | 25% | 39% | 68% | 62% | 50% | 24% | 81% |
| Mozambique | 0% | 0% | 2% | 9% | 4% | 0% | 0% | 0% | 1% | 0% | 0% | 0% | 1% |
| Namibia | 6% | 1% | 0% | 2% | 6% | 0% | 5% | 0% | 1% | 7% | 0% | 1% | 0% |
| Nepal | 47% | 5% | 0% | 45% | 14% | 1% | 2% | 0% | 11% | 10% | 4% | 9% | 2% |
| Nicaragua | 2% | 8% | 2% | 0% | 0% | 0% | 2% | 4% | 0% | 2% | 1% | 1% | 0% |
| Niger | 0% | 1% | 2% | 2% | 0% | 0% | 0% | 0% | 0% | 0% | 0% | 0% | 0% |
| Nigeria | 0% | 0% | 0% | 1% | 0% | 0% | 0% | 0% | 0% | 0% | 0% | 0% | 0% |
| Pakistan | 0% | 0% | 0% | 0% | 0% | 0% | 0% | 0% | 0% | 0% | 0% | 0% | 0% |
| Peru | 0% | 0% | 0% | 0% | 0% | 0% | 0% | 0% | 0% | 0% | 0% | 0% | 0% |
| Philippines | 0% | 0% | 0% | 0% | 0% | 0% | 0% | 0% | 0% | 0% | 0% | 0% | 0% |
| Rwanda | 0% | 0% | 0% | 0% | 0% | 0% | 0% | 0% | 0% | 0% | 0% | 0% | 0% |
| Senegal | 0% | 0% | 0% | 0% | 38% | 0% | 0% | 0% | 0% | 0% | 0% | 0% | 0% |
| Sierra Leone | 79% | 27% | 31% | 52% | 14% | 25% | 51% | 39% | 28% | 84% | 38% | 42% | 41% |
| South Africa | 0% | 0% | 0% | 0% | 0% | 0% | 0% | 0% | 0% | 0% | 0% | 0% | 0% |
| Tajikistan | 2% | 2% | 1% | 0% | 0% | 0% | 2% | 20% | 0% | 0% | 1% | 1% | 1% |
| Tanzania | 0% | 0% | 0% | 0% | 0% | 0% | 0% | 0% | 0% | 0% | 0% | 0% | 0% |
| Timor-Leste | 0% | 0% | 1% | 1% | 1% | 0% | 0% | 0% | 0% | 0% | 1% | 0% | 1% |
| Togo | 0% | 0% | 0% | 1% | 1% | 0% | 0% | 0% | 1% | 3% | 0% | 0% | 0% |
| Turkey | 0% | 0% | 0% | 0% | 2% | 0% | 0% | 0% | 0% | 0% | 0% | 0% | 0% |
| Uganda | 0% | 0% | 0% | 0% | 0% | 0% | 0% | 0% | 0% | 0% | 0% | 0% | 0% |
| Vietnam | 6% | 42% | 61% | 46% | 100% | 95% | 24% | 3% | 92% | 0% | 38% | 49% | 22% |
| Yemen | N/A | N/A | N/A | N/A | N/A | 0% | 0% | 0% | 0% | 0% | 0% | 0% | 0% |
| Zambia | 0% | 0% | 0% | 0% | 1% | 0% | 0% | 0% | 0% | 1% | 0% | 0% | 0% |
| Zimbabwe | 0% | 0% | 0% | 0% | 0% | 0% | 0% | 0% | 0% | 0% | 0% | 0% | 0% |

**Note:** N/A denotes not application and refers that the indicator has no sufficient data to make projections

Table S21: Predictive probability of achieving universal coverage of oral rehydration therapy for diarrhoea treatment among children 0-59 months of age in low- and middle-income countries in 2030 by socio-demographic factors

| **Country** | **Wealth quintiles** | | | | | **Place of residence** | | **Education of mother** | | **Age of mother** | | **Sex of child** | |
| --- | --- | --- | --- | --- | --- | --- | --- | --- | --- | --- | --- | --- | --- |
|  | **Poorest** | **Poorer** | **Middle** | **Richer** | **Richest** | **Urban** | **Rural** | **Below secondary** | **Secondary+** | **Adolescent** | **Adult** | **Male** | **Female** |
| Albania | 0% | 0% | 0% | 0% | 1% | 0% | 0% | 0% | 0% | 0% | 0% | 0% | 0% |
| Armenia | 0% | 0% | 6% | 0% | 4% | 1% | 0% | 37% | 0% | 100% | 0% | 0% | 0% |
| Bangladesh | 0% | 1% | 0% | 2% | 0% | 0% | 0% | 0% | 0% | 0% | 0% | 0% | 0% |
| Benin | 0% | 0% | 0% | 0% | 0% | 0% | 0% | 0% | 0% | 0% | 0% | 0% | 0% |
| Bolivia | 0% | 0% | 0% | 0% | 0% | 0% | 0% | 0% | 0% | 0% | 0% | 0% | 0% |
| Brazil | N/A | N/A | N/A | N/A | N/A | 20% | 5% | 10% | 25% | 45% | 9% | 8% | 23% |
| Burkina Faso | 0% | 0% | 0% | 0% | 0% | 0% | 0% | 0% | 0% | 0% | 0% | 0% | 0% |
| Burundi | 0% | 0% | 0% | 0% | 0% | 0% | 0% | 0% | 0% | 0% | 0% | 0% | 0% |
| Cambodia | 0% | 0% | 0% | 0% | 0% | 0% | 0% | 0% | 0% | 0% | 0% | 0% | 0% |
| Cameroon | 0% | 0% | 0% | 0% | 0% | 0% | 0% | 0% | 0% | 0% | 0% | 0% | 0% |
| Chad | 0% | 0% | 0% | 0% | 0% | 0% | 0% | 0% | 0% | 0% | 0% | 0% | 0% |
| Colombia | 0% | 0% | 0% | 0% | 0% | 0% | 0% | 0% | 0% | 0% | 0% | 0% | 0% |
| Comoros | 0% | 0% | 1% | 0% | 0% | 0% | 0% | 0% | 0% | 0% | 0% | 0% | 0% |
| Congo | 0% | 1% | 0% | 0% | 4% | 0% | 0% | 0% | 0% | 1% | 1% | 1% | 1% |
| Congo DR | 0% | 0% | 0% | 0% | 0% | 0% | 0% | 0% | 0% | 0% | 0% | 0% | 0% |
| Cote d'Ivoire | 0% | 0% | 0% | 0% | 0% | 0% | 0% | 0% | 0% | 0% | 0% | 0% | 0% |
| Dominican Republic | 0% | 0% | 0% | 0% | 0% | 0% | 0% | 0% | 0% | 0% | 0% | 0% | 0% |
| Egypt | 0% | 0% | 0% | 0% | 0% | 0% | 0% | 0% | 0% | 0% | 0% | 0% | 0% |
| Ethiopia | 0% | 0% | 0% | 0% | 0% | 0% | 0% | 0% | 0% | 0% | 0% | 0% | 0% |
| Gabon | 0% | 0% | 0% | 0% | 0% | 0% | 0% | 0% | 0% | 0% | 0% | 0% | 0% |
| Ghana | 0% | 0% | 0% | 0% | 0% | 0% | 0% | 0% | 0% | 0% | 0% | 0% | 0% |
| Guatemala | 0% | 0% | 0% | 0% | 0% | 0% | 0% | 0% | 0% | 0% | 0% | 0% | 0% |
| Guinea | 0% | 0% | 0% | 0% | 0% | 0% | 0% | 0% | 0% | 0% | 0% | 0% | 0% |
| Haiti | 0% | 0% | 0% | 0% | 0% | 0% | 0% | 0% | 0% | 0% | 0% | 0% | 0% |
| Honduras | 1% | 2% | 0% | 0% | 1% | 1% | 1% | 2% | 1% | 1% | 1% | 1% | 2% |
| India | 0% | 0% | 0% | 0% | 0% | 0% | 0% | 0% | 0% | 0% | 0% | 0% | 0% |
| Indonesia | 0% | 0% | 0% | 0% | 0% | 0% | 0% | 0% | 0% | 0% | 0% | 0% | 0% |
| Jordan | 0% | 0% | 0% | 0% | 0% | 0% | 0% | 0% | 0% | 0% | 0% | 0% | 0% |
| Kazakhstan | 0% | 3% | 5% | 91% | 67% | 30% | 1% | N/A | 7% | 1% | 6% | 7% | 5% |
| Kenya | 0% | 0% | 0% | 0% | 0% | 0% | 0% | 0% | 0% | 0% | 0% | 0% | 0% |
| Kyrgyz Republic | 1% | 0% | 1% | 0% | 0% | 1% | 0% | N/A | 0% | 100% | 0% | 0% | 1% |
| Lesotho | 0% | 0% | 0% | 1% | 0% | 0% | 0% | 0% | 0% | 1% | 0% | 0% | 0% |
| Liberia | 1% | 6% | 4% | 1% | 0% | 0% | 2% | 1% | 0% | 1% | 1% | 0% | 2% |
| Madagascar | 0% | 0% | 0% | 0% | 0% | 0% | 0% | 0% | 0% | 0% | 0% | 0% | 0% |
| Malawi | 0% | 0% | 0% | 0% | 0% | 0% | 0% | 0% | 0% | 0% | 0% | 0% | 0% |
| Maldives | 0% | 2% | 61% | 12% | 100% | 100% | 1% | 26% | 10% | 0% | 8% | 2% | 27% |
| Mali | 0% | 0% | 0% | 0% | 0% | 0% | 0% | 0% | 0% | 0% | 0% | 0% | 0% |
| Morocco | 0% | 0% | 1% | 0% | 0% | 1% | 0% | 0% | 0% | 7% | 0% | 0% | 0% |
| Mozambique | 0% | 0% | 0% | 0% | 0% | 0% | 0% | 0% | 0% | 0% | 0% | 0% | 0% |
| Namibia | 0% | 0% | 0% | 0% | 0% | 0% | 0% | 0% | 0% | 0% | 0% | 0% | 0% |
| Nepal | 0% | 0% | 0% | 0% | 0% | 0% | 0% | 0% | 0% | 0% | 0% | 0% | 0% |
| Nicaragua | 2% | 7% | 21% | 10% | 2% | 6% | 8% | 6% | 6% | 10% | 4% | 10% | 4% |
| Niger | 0% | 0% | 0% | 0% | 0% | 0% | 0% | 0% | 0% | 0% | 0% | 0% | 0% |
| Nigeria | 0% | 0% | 0% | 0% | 0% | 0% | 0% | 0% | 0% | 0% | 0% | 0% | 0% |
| Pakistan | 0% | 0% | 0% | 0% | 0% | 0% | 0% | 0% | 0% | 0% | 0% | 0% | 0% |
| Peru | 0% | 0% | 0% | 0% | 0% | 0% | 0% | 0% | 0% | 0% | 0% | 0% | 0% |
| Philippines | 0% | 0% | 0% | 0% | 0% | 0% | 0% | 0% | 0% | 0% | 0% | 0% | 0% |
| Rwanda | 0% | 0% | 0% | 0% | 0% | 0% | 0% | 0% | 0% | 0% | 0% | 0% | 0% |
| Senegal | 0% | 0% | 0% | 0% | 0% | 0% | 0% | 0% | 0% | 0% | 0% | 0% | 0% |
| Sierra Leone | 94% | 68% | 17% | 11% | 37% | 37% | 63% | 55% | 54% | 88% | 50% | 66% | 41% |
| South Africa | 0% | 0% | 0% | 0% | 0% | 0% | 0% | 0% | 0% | 0% | 0% | 0% | 0% |
| Tajikistan | 0% | 0% | 0% | 1% | 1% | 1% | 0% | 0% | 0% | 0% | 0% | 0% | 0% |
| Tanzania | 0% | 0% | 0% | 0% | 0% | 0% | 0% | 0% | 0% | 0% | 0% | 0% | 0% |
| Timor-Leste | 0% | 6% | 0% | 1% | 0% | 1% | 1% | 1% | 0% | 0% | 1% | 1% | 0% |
| Togo | 0% | 0% | 0% | 0% | 0% | 0% | 0% | 0% | 0% | 0% | 0% | 0% | 0% |
| Turkey | 0% | 0% | 0% | 0% | 0% | 0% | 0% | 0% | 0% | 0% | 0% | 0% | 0% |
| Uganda | 0% | 0% | 0% | 0% | 0% | 0% | 0% | 0% | 0% | 0% | 0% | 0% | 0% |
| Vietnam | 2% | 0% | 12% | 3% | 0% | 0% | 0% | 0% | 2% | 0% | 1% | 0% | 4% |
| Yemen | N/A | N/A | N/A | N/A | N/A | 0% | 0% | 0% | 0% | 0% | 0% | 0% | 0% |
| Zambia | 0% | 0% | 0% | 0% | 0% | 0% | 0% | 0% | 0% | 0% | 0% | 0% | 0% |
| Zimbabwe | 0% | 0% | 0% | 0% | 0% | 0% | 0% | 0% | 0% | 0% | 0% | 0% | 0% |

**Note:** N/A denotes not application and refers that the indicator has no sufficient data to make projections

Table S22: Predictive probability of achieving universal coverage of care seeking for acute respiratory infections among children 0-59 months of age in low- and middle-income countries in 2030 by socio-demographic factors

| **Country** | **Wealth quintiles** | | | | | **Place of residence** | | **Education of mother** | | **Age of mother** | | **Sex of child** | |
| --- | --- | --- | --- | --- | --- | --- | --- | --- | --- | --- | --- | --- | --- |
|  | **Poorest** | **Poorer** | **Middle** | **Richer** | **Richest** | **Urban** | **Rural** | **Below secondary** | **Secondary+** | **Adolescent** | **Adult** | **Male** | **Female** |
| Albania | 0% | 0% | 0% | 0% | 98% | 0% | 0% | 0% | 0% | 0% | 0% | 0% | 0% |
| Armenia | 10% | 0% | 0% | 0% | 64% | 0% | 3% | N/A | 0% | 100% | 0% | 0% | 2% |
| Bangladesh | 0% | 0% | 0% | 0% | 0% | 0% | 0% | 0% | 0% | 0% | 0% | 0% | 0% |
| Benin | 0% | 0% | 0% | 0% | 0% | 0% | 0% | 0% | 0% | 0% | 0% | 0% | 0% |
| Bolivia | 0% | 0% | 0% | 0% | 0% | 0% | 0% | 0% | 0% | 0% | 0% | 0% | 0% |
| Brazil | N/A | N/A | N/A | N/A | N/A | 23% | 16% | 12% | 14% | 40% | 38% | 55% | 26% |
| Burkina Faso | 0% | 0% | 1% | 0% | 1% | 0% | 0% | 0% | 0% | 1% | 0% | 0% | 0% |
| Burundi | 0% | 0% | 0% | 0% | 0% | 0% | 0% | 0% | 0% | 0% | 0% | 0% | 0% |
| Cambodia | 2% | 4% | 8% | 0% | 2% | 4% | 2% | 2% | 1% | 20% | 2% | 0% | 7% |
| Cameroon | 0% | 0% | 0% | 0% | 0% | 0% | 0% | 0% | 0% | 0% | 0% | 0% | 0% |
| Chad | 0% | 0% | 0% | 0% | 0% | 0% | 0% | 0% | 0% | 0% | 0% | 0% | 0% |
| Colombia | 0% | 0% | 0% | 1% | 0% | 0% | 1% | 0% | 0% | 0% | 0% | 0% | 0% |
| Comoros | 0% | 0% | 0% | 0% | 0% | 0% | 0% | 0% | 0% | 0% | 0% | 0% | 0% |
| Congo | 0% | 0% | 0% | 0% | 2% | 0% | 0% | 0% | 0% | 0% | 0% | 0% | 1% |
| Congo DR | 0% | 0% | 0% | 0% | 0% | 0% | 0% | 0% | 0% | 0% | 0% | 0% | 0% |
| Cote d'Ivoire | 0% | 0% | 0% | 0% | 0% | 0% | 0% | 0% | 0% | 0% | 0% | 0% | 0% |
| Dominican Republic | 0% | 0% | 0% | 0% | 0% | 0% | 0% | 0% | 0% | 0% | 0% | 0% | 0% |
| Egypt | 0% | 0% | 0% | 0% | 0% | 0% | 0% | 0% | 0% | 0% | 0% | 0% | 0% |
| Ethiopia | 0% | 0% | 0% | 0% | 0% | 0% | 0% | 0% | 0% | 0% | 0% | 0% | 0% |
| Gabon | 0% | 0% | 0% | 0% | 0% | 0% | 0% | 0% | 0% | 0% | 0% | 0% | 0% |
| Ghana | 0% | 0% | 0% | 0% | 0% | 0% | 0% | 0% | 0% | 0% | 0% | 0% | 0% |
| Guatemala | 0% | 0% | 0% | 0% | 0% | 0% | 0% | 0% | 0% | 0% | 0% | 0% | 0% |
| Guinea | 0% | 0% | 0% | 0% | 0% | 0% | 0% | 0% | 0% | 0% | 0% | 0% | 0% |
| Guyana | 6% | 3% | 8% | 98% | 0% | 28% | 2% | 95% | 1% | 3% | 4% | 8% | 2% |
| Haiti | 0% | 0% | 0% | 0% | 0% | 0% | 0% | 0% | 0% | 0% | 0% | 0% | 0% |
| Honduras | 9% | 4% | 6% | 2% | 0% | 2% | 4% | 3% | 1% | 8% | 2% | 3% | 3% |
| India | 0% | 0% | 0% | 0% | 0% | 0% | 0% | 0% | 0% | 0% | 0% | 0% | 0% |
| Indonesia | 0% | 0% | 0% | 0% | 0% | 0% | 0% | 0% | 0% | 0% | 0% | 0% | 0% |
| Jordan | 0% | 0% | 0% | 0% | 0% | 0% | 0% | 0% | 0% | 0% | 0% | 0% | 0% |
| Kazakhstan | 0% | 73% | 99% | 86% | 1% | 1% | 86% | N/A | 9% | N/A | 8% | N/A | 1% |
| Kenya | 0% | 0% | 0% | 0% | 0% | 0% | 0% | 0% | 0% | 0% | 0% | 0% | 0% |
| Kyrgyz Republic | 0% | 0% | 0% | 0% | 0% | 0% | 0% | N/A | 0% | 100% | 0% | 0% | 0% |
| Lesotho | 0% | 0% | 0% | 0% | 0% | 0% | 0% | 0% | 0% | 0% | 0% | 0% | 0% |
| Liberia | 1% | 0% | 0% | 0% | 0% | 0% | 0% | 0% | 0% | 0% | 0% | 0% | 0% |
| Madagascar | 0% | 0% | 0% | 0% | 0% | 0% | 0% | 0% | 0% | 0% | 0% | 0% | 0% |
| Malawi | 0% | 0% | 0% | 0% | 0% | 0% | 0% | 0% | 0% | 0% | 0% | 0% | 0% |
| Maldives | 83% | 0% | 100% | 0% | 0% | 0% | 38% | 100% | 0% | 0% | 0% | 0% | 5% |
| Mali | 0% | 0% | 0% | 0% | 0% | 0% | 0% | 0% | 0% | 0% | 0% | 0% | 0% |
| Morocco | 0% | 2% | 1% | 3% | 2% | 2% | 0% | 1% | 0% | 3% | 2% | 1% | 0% |
| Mozambique | 0% | 0% | 0% | 0% | 0% | 0% | 0% | 0% | 0% | 0% | 0% | 0% | 0% |
| Namibia | 0% | 0% | 0% | 0% | 0% | 0% | 0% | 0% | 0% | 0% | 0% | 0% | 0% |
| Nepal | 0% | 0% | 0% | 0% | 0% | 0% | 0% | 0% | 0% | 0% | 0% | 0% | 0% |
| Nicaragua | 11% | 24% | 12% | 4% | 19% | 17% | 12% | 10% | 15% | 26% | 12% | 16% | 9% |
| Niger | 0% | 1% | 0% | 0% | 0% | 0% | 0% | 0% | 0% | 0% | 0% | 0% | 0% |
| Nigeria | 0% | 0% | 0% | 0% | 0% | 0% | 0% | 0% | 0% | 0% | 0% | 0% | 0% |
| Pakistan | 0% | 0% | 0% | 0% | 0% | 0% | 0% | 0% | 0% | 0% | 0% | 0% | 0% |
| Peru | 0% | 0% | 0% | 0% | 0% | 0% | 0% | 0% | 0% | 0% | 0% | 0% | 0% |
| Philippines | 0% | 0% | 0% | 0% | 0% | 0% | 0% | 0% | 0% | 0% | 0% | 0% | 0% |
| Rwanda | 0% | 0% | 0% | 0% | 0% | 0% | 0% | 0% | 0% | 0% | 0% | 0% | 0% |
| Senegal | 0% | 0% | 0% | 0% | 0% | 0% | 0% | 0% | 0% | 0% | 0% | 0% | 0% |
| Sierra Leone | 18% | 10% | 28% | 22% | 11% | 16% | 17% | 13% | 30% | 84% | 14% | 21% | 14% |
| South Africa | 0% | 0% | 0% | 0% | 0% | 0% | 0% | 0% | 0% | 0% | 0% | 0% | 0% |
| Tajikistan | 0% | 0% | 0% | 1% | 3% | 2% | 0% | 0% | 0% | 0% | 0% | 0% | 0% |
| Tanzania | 0% | 0% | 0% | 0% | 0% | 0% | 0% | 0% | 0% | 0% | 0% | 0% | 0% |
| Timor-Leste | 1% | 1% | 0% | 1% | 85% | 2% | 0% | 1% | 0% | 0% | 1% | 1% | 0% |
| Togo | 0% | 0% | 0% | 0% | 0% | 0% | 0% | 0% | 0% | 0% | 0% | 0% | 0% |
| Turkey | 0% | 1% | 2% | 0% | 1% | 0% | 0% | 1% | 1% | 0% | 1% | 1% | 0% |
| Uganda | 0% | 0% | 0% | 0% | 0% | 0% | 0% | 0% | 0% | 0% | 0% | 0% | 0% |
| Vietnam | 20% | 30% | 3% | 1% | 4% | 7% | 10% | 32% | 3% | 0% | 13% | 15% | 5% |
| Yemen | N/A | N/A | N/A | N/A | N/A | 0% | 0% | 0% | 0% | 0% | 0% | 0% | 0% |
| Zambia | 0% | 0% | 0% | 0% | 0% | 0% | 0% | 0% | 0% | 0% | 0% | 0% | 0% |
| Zimbabwe | 0% | 0% | 0% | 0% | 0% | 0% | 0% | 0% | 0% | 0% | 0% | 0% | 0% |

**Note:** N/A denotes not application and refers that the indicator has no sufficient data to make projections

Table S23: Predictive probability of achieving at least 75% coverage of demand for family planning satisfied with modern contraceptive method among women 15-49 years of age in low- and middle-income countries in 2030 by socio-demographic factors

| **Country** | **Overall** | **Wealth quintiles** | | | | | **Place of residence** | | **Education of women** | | **Age of women** | |
| --- | --- | --- | --- | --- | --- | --- | --- | --- | --- | --- | --- | --- |
|  |  | **Poorest** | **Poorer** | **Middle** | **Richer** | **Richest** | **Urban** | **Rural** | **Below secondary** | **Secondary+** | **Adolescent** | **Adult** |
| Albania | 0% | 0% | 0% | 0% | 0% | 0% | 0% | 0% | 0% | 0% | 0% | 0% |
| Armenia | 5% | 2% | 1% | 4% | 24% | 17% | 18% | 1% | 1% | 7% | 8% | 6% |
| Bangladesh | 99% | 95% | 99% | 93% | 100% | 78% | 90% | 99% | 98% | 97% | 99% | 98% |
| Benin | 0% | 0% | 1% | 2% | 0% | 1% | 0% | 0% | 0% | 2% | 2% | 1% |
| Bolivia | 63% | 99% | 81% | 71% | 74% | 33% | 59% | 82% | 80% | 48% | 75% | 61% |
| Brazil | 95% | N/A | N/A | N/A | N/A | N/A | 92% | 89% | 90% | 68% | 57% | 95% |
| Burkina Faso | 94% | 92% | 97% | 96% | 98% | 95% | 93% | 99% | 92% | 97% | 79% | 92% |
| Burundi | 15% | 22% | 30% | 15% | 5% | 14% | 14% | 14% | 15% | 11% | 65% | 15% |
| Cambodia | 86% | 97% | 99% | 84% | 67% | 1% | 1% | 93% | 91% | 18% | 86% | 86% |
| Cameroon | 67% | 0% | 47% | 57% | 92% | 93% | 85% | 9% | 5% | 95% | 100% | 35% |
| Chad | 2% | 65% | 9% | 24% | 0% | 3% | 2% | 6% | 1% | 5% | 0% | 3% |
| Colombia | 100% | 100% | 100% | 100% | 100% | 100% | 100% | 100% | 100% | 100% | 99% | 100% |
| Comoros | 1% | 1% | 1% | 2% | 7% | 0% | 7% | 0% | 1% | 1% | 1% | 2% |
| Congo | 59% | 4% | 45% | 87% | 79% | 75% | 77% | 8% | 40% | 63% | 92% | 49% |
| Congo DR | 5% | 1% | 1% | 2% | 13% | 9% | 12% | 2% | 1% | 7% | 3% | 6% |
| Cote d'Ivoire | 26% | 17% | 15% | 53% | 36% | 35% | 33% | 16% | 16% | 46% | 58% | 22% |
| Dominican Republic | 100% | 99% | 99% | 91% | 86% | 99% | 100% | 100% | 100% | 99% | 97% | 100% |
| Egypt | 100% | 99% | 97% | 100% | 98% | 95% | 100% | 100% | 100% | 99% | 94% | 100% |
| Ethiopia | 100% | 96% | 100% | 100% | 100% | 100% | 100% | 100% | 100% | 99% | 100% | 100% |
| Gabon | 33% | 14% | 31% | 16% | 74% | 65% | 38% | 11% | 8% | 40% | 81% | 27% |
| Ghana | 4% | 15% | 19% | 15% | 2% | 2% | 2% | 10% | 4% | 1% | 0% | 5% |
| Guatemala | 75% | 77% | 88% | 88% | 87% | 77% | 80% | 73% | 73% | 46% | 17% | 80% |
| Guinea | 2% | 6% | 1% | 2% | 0% | 26% | 9% | 1% | 1% | 8% | 2% | 2% |
| Guyana | 99% | 97% | 100% | 100% | 100% | 100% | 100% | 100% | 99% | 100% | 100% | 100% |
| Haiti | 9% | 24% | 20% | 28% | 1% | 3% | 3% | 13% | 7% | 4% | 17% | 11% |
| Honduras | 81% | 89% | 88% | 82% | 80% | 72% | 77% | 85% | 83% | 74% | 68% | 85% |
| India | 83% | 20% | 86% | 91% | 89% | 83% | 85% | 80% | 96% | 45% | 0% | 78% |
| Indonesia | 96% | 94% | 97% | 86% | 73% | 7% | 75% | 99% | 99% | 81% | 99% | 96% |
| Jordan | 9% | 28% | 37% | 10% | 13% | 6% | 5% | 20% | 1% | 12% | 25% | 9% |
| Kazakhstan | 74% | 54% | 32% | 91% | 82% | 83% | 81% | 68% | N/A | 78% | 26% | 78% |
| Kenya | 96% | 75% | 97% | 98% | 99% | 94% | 94% | 95% | 95% | 99% | 95% | 97% |
| Kyrgyz Republic | 3% | 13% | 6% | 1% | 1% | 4% | 2% | 3% | 100% | 3% | 1% | 4% |
| Lesotho | 99% | 99% | 100% | 100% | 99% | 98% | 96% | 99% | 98% | 99% | 96% | 99% |
| Liberia | 100% | 100% | 100% | 100% | 100% | 100% | 100% | 100% | 100% | 100% | 100% | 100% |
| Madagascar | 100% | 100% | 99% | 98% | 97% | 36% | 71% | 100% | 100% | 81% | 99% | 100% |
| Malawi | 100% | 100% | 100% | 100% | 100% | 99% | 99% | 100% | 100% | 73% | 96% | 100% |
| Maldives | 0% | 0% | 0% | 0% | 0% | 0% | 0% | 0% | 0% | 0% | 0% | 0% |
| Mali | 1% | 7% | 2% | 0% | 5% | 15% | 15% | 3% | 0% | 5% | 2% | 1% |
| Morocco | 94% | 100% | 98% | 91% | 86% | 73% | 81% | 99% | 96% | 69% | 96% | 95% |
| Mozambique | 52% | 52% | 19% | 21% | 79% | 85% | 61% | 53% | 30% | 42% | 73% | 52% |
| Namibia | 100% | 97% | 100% | 100% | 99% | 100% | 98% | 99% | 95% | 99% | 93% | 100% |
| Nepal | 20% | 41% | 47% | 41% | 24% | 1% | 2% | 19% | 73% | 0% | 2% | 20% |
| Nicaragua | 83% | 77% | 86% | 89% | 70% | 79% | 79% | 84% | 85% | 69% | 84% | 79% |
| Niger | 34% | 94% | 47% | 28% | 70% | 32% | 48% | 66% | 31% | 25% | 19% | 35% |
| Nigeria | 8% | 0% | 2% | 16% | 33% | 27% | 6% | 7% | 0% | 5% | 12% | 6% |
| Pakistan | 5% | 64% | 18% | 18% | 16% | 2% | 2% | 15% | 7% | 0% | 0% | 5% |
| Peru | 57% | 66% | 32% | 48% | 62% | 98% | 71% | 65% | 37% | 48% | 92% | 58% |
| Philippines | 2% | 30% | 22% | 4% | 1% | 0% | 0% | 12% | 5% | 1% | 1% | 2% |
| Rwanda | 85% | 80% | 81% | 91% | 82% | 88% | 73% | 84% | 85% | 74% | 5% | 86% |
| Senegal | 9% | 53% | 76% | 83% | 67% | 26% | 44% | 20% | 13% | 1% | 0% | 13% |
| Sierra Leone | 95% | 99% | 99% | 99% | 98% | 94% | 96% | 99% | 95% | 96% | 99% | 95% |
| South Africa | 46% | 87% | 72% | 67% | 42% | 2% | 25% | 79% | 19% | 36% | 45% | 46% |
| Tajikistan | 14% | 16% | 10% | 14% | 29% | 15% | 27% | 14% | 7% | 16% | 11% | 14% |
| Tanzania | 61% | 45% | 25% | 81% | 75% | 6% | 31% | 85% | 68% | 8% | 59% | 63% |
| Timor-Leste | 7% | 25% | 9% | 17% | 3% | 2% | 2% | 12% | 7% | 5% | 4% | 7% |
| Togo | 22% | 49% | 33% | 16% | 23% | 25% | 17% | 22% | 15% | 21% | 21% | 25% |
| Turkey | 9% | 14% | 5% | 6% | 24% | 19% | 6% | 5% | 5% | 3% | 0% | 8% |
| Uganda | 43% | 19% | 83% | 79% | 93% | 39% | 23% | 57% | 38% | 29% | 16% | 49% |
| Vietnam | 41% | 78% | 42% | 24% | 30% | 31% | 52% | 39% | 70% | 28% | 2% | 41% |
| Yemen | 1% | N/A | N/A | N/A | N/A | N/A | 0% | 1% | 1% | 0% | 0% | 7% |
| Zambia | 99% | 93% | 84% | 97% | 97% | 97% | 99% | 99% | 99% | 93% | 61% | 100% |
| Zimbabwe | 100% | 100% | 100% | 100% | 100% | 100% | 99% | 100% | 100% | 99% | 62% | 100% |

**Note:** N/A denotes not application and refers that the indicator has no sufficient data to make projections

Table S24: Predicted average annual rate of increase (AARC) of composite coverage index (CCI) in low- and middle-income countries by wealth quintiles

| **Country** | **Poorest** | | | **Poorer** | | | **Middle** | | | **Richer** | | | **Richest** | | |
| --- | --- | --- | --- | --- | --- | --- | --- | --- | --- | --- | --- | --- | --- | --- | --- |
|  | **Predicted** | | **Required** | **Predicted** | | **Required** | **Predicted** | | **Required** | **Predicted** | | **Required** | **Predicted** | | **Required** |
|  | **1990-2018** | **2019-2020** | **1990-2018** | **2019-2020** | **2019-2030** | **2019-2030** | **2019-2020** | **2019-2030** | **2019-2030** | **2019-2020** | **2019-2030** | **2019-2030** | **2019-2020** | **2019-2030** | **2019-2030** |
| Armenia | 2.1 | 1.0 | 2.7 | 1.3 | 0.7 | 2.9 | 1.7 | 0.8 | 2.3 | 1.2 | 0.6 | 2.5 | 2.4 | 0.7 | 1.5 |
| Bangladesh | 2.1 | 1.2 | 3.7 | 2.5 | 1.2 | 2.9 | 2.2 | 1.0 | 2.7 | 2.2 | 0.9 | 2.1 | 1.5 | 0.6 | 1.5 |
| Benin | 0.7 | 0.7 | 7.9 | 0.9 | 0.7 | 6.3 | 0.3 | 0.3 | 5.8 | 0.2 | 0.2 | 5.1 | -0.2 | -0.2 | 4.3 |
| Bolivia | 5.3 | 1.4 | 2.3 | 3.7 | 1.1 | 2.1 | 2.8 | 0.9 | 2.0 | 1.5 | 0.7 | 2.0 | 0.2 | 0.1 | 2.6 |
| Burkina Faso | 4.2 | 2.3 | 5.9 | 4.2 | 2.1 | 4.8 | 4.4 | 1.8 | 3.9 | 3.4 | 1.5 | 3.7 | 1.4 | 0.6 | 2.3 |
| Burundi | 0.3 | 0.0 | 4.5 | 0.6 | 0.2 | 4.1 | -0.1 | -0.5 | 4.3 | -0.2 | -0.5 | 4.4 | -0.1 | -0.7 | 3.6 |
| Cambodia | 6.8 | 1.3 | 1.9 | 6.4 | 1.1 | 1.6 | 5.4 | 1.0 | 1.5 | 4.0 | 0.9 | 1.6 | 1.2 | 0.6 | 2.3 |
| Cameroon | 0.5 | 0.6 | 10.3 | 1.9 | 1.3 | 6.0 | 1.5 | 1.0 | 4.2 | 0.8 | 0.6 | 3.6 | 0.8 | 0.5 | 2.6 |
| Chad | 8.3 | 5.3 | 10.7 | 5.4 | 3.8 | 10.2 | 4.9 | 3.5 | 9.8 | 2.0 | 1.9 | 10.7 | 1.7 | 1.2 | 6.1 |
| Colombia | 1.9 | 0.6 | 1.6 | 0.9 | 0.3 | 1.2 | 0.9 | 0.3 | 1.1 | 0.5 | 0.2 | 1.2 | 0.3 | 0.1 | 1.3 |
| Comoros | 2.2 | 1.3 | 4.9 | 1.4 | 0.9 | 4.4 | 1.3 | 0.8 | 3.7 | 1.3 | 0.7 | 2.9 | 0.0 | -0.1 | 3.9 |
| Congo | 1.4 | 1.0 | 6.1 | 2.2 | 1.0 | 3.9 | 1.8 | 0.8 | 3.5 | 1.4 | 0.5 | 3.1 | 1.3 | 0.4 | 2.7 |
| Congo DR | 1.8 | 1.6 | 7.5 | 1.8 | 1.5 | 6.8 | 1.3 | 1.1 | 6.6 | 1.4 | 0.9 | 5.3 | 0.7 | 0.3 | 4.4 |
| Cote d'Ivoire | 2.6 | 1.9 | 7.9 | 1.1 | 0.9 | 7.1 | 1.8 | 1.2 | 5.6 | 1.2 | 0.7 | 4.2 | 0.6 | 0.4 | 3.6 |
| Dominican Republic | 1.1 | 0.5 | 1.6 | 0.9 | 0.4 | 1.3 | 0.3 | 0.2 | 1.9 | 0.3 | 0.2 | 1.9 | 0.7 | 0.4 | 1.6 |
| Egypt | 1.8 | 0.8 | 2.2 | 1.2 | 0.6 | 2.0 | 0.9 | 0.5 | 1.8 | 0.5 | 0.3 | 1.8 | 0.0 | 0.0 | 2.1 |
| Ethiopia | 8.0 | 4.3 | 7.9 | 9.4 | 3.7 | 5.7 | 8.6 | 3.3 | 5.2 | 8.4 | 2.9 | 4.4 | 4.3 | 1.4 | 2.6 |
| Gabon | 1.5 | 1.0 | 5.8 | 0.7 | 0.4 | 4.9 | -0.4 | -0.5 | 5.7 | 0.6 | 0.3 | 3.7 | -0.2 | -0.3 | 4.6 |
| Ghana | 2.8 | 1.4 | 3.7 | 2.3 | 1.2 | 3.2 | 1.9 | 1.0 | 2.7 | 1.1 | 0.7 | 2.7 | 0.5 | 0.3 | 2.7 |
| Guatemala | 3.1 | 1.3 | 3.1 | 2.7 | 1.1 | 2.7 | 1.8 | 0.8 | 2.4 | 1.2 | 0.6 | 2.0 | 0.3 | 0.2 | 1.9 |
| Guinea | -0.1 | 0.2 | 11.2 | 1.3 | 1.1 | 8.1 | 0.1 | 0.2 | 7.8 | -0.6 | -0.6 | 6.7 | 0.4 | 0.2 | 3.5 |
| Haiti | 3.0 | 2.0 | 6.4 | 2.4 | 1.6 | 5.6 | 2.0 | 1.3 | 4.5 | 1.3 | 0.9 | 4.2 | 1.0 | 0.7 | 3.3 |
| Honduras | 4.8 | 0.6 | 1.0 | 3.4 | 0.5 | 1.1 | 1.7 | 0.3 | 1.3 | 1.1 | 0.1 | 1.4 | 0.7 | -0.1 | 1.6 |
| India | 2.8 | 1.4 | 3.9 | 2.9 | 1.2 | 2.6 | 2.0 | 0.8 | 2.0 | 1.4 | 0.6 | 1.7 | 0.6 | 0.3 | 1.5 |
| Indonesia | 0.9 | 0.6 | 3.9 | 0.8 | 0.5 | 2.8 | 0.5 | 0.3 | 2.5 | 0.2 | 0.1 | 2.5 | -0.6 | -0.9 | 3.3 |
| Jordan | 0.9 | 0.5 | 2.2 | 0.7 | 0.4 | 2.2 | 0.8 | 0.4 | 2.0 | 0.7 | 0.4 | 2.0 | 0.9 | 0.4 | 1.8 |
| Kazakhstan | -0.1 | -0.3 | 4.2 | 2.0 | 0.1 | 1.2 | 3.0 | 0.1 | 0.6 | 2.7 | 0.1 | 0.5 | 1.0 | 0.0 | 1.8 |
| Kenya | 1.4 | 0.9 | 4.1 | 1.2 | 0.7 | 3.1 | 1.2 | 0.6 | 2.4 | 0.8 | 0.4 | 2.2 | 0.6 | 0.3 | 1.7 |
| Kyrgyz Republic | 0.3 | 0.1 | 2.5 | -0.1 | -0.3 | 2.8 | -0.2 | -0.3 | 2.9 | -0.3 | -0.4 | 3.5 | -0.4 | -0.7 | 2.8 |
| Lesotho | 3.7 | 0.9 | 1.9 | 2.2 | 0.7 | 1.9 | 2.3 | 0.6 | 1.4 | 1.8 | 0.5 | 1.1 | 0.1 | -0.1 | 1.6 |
| Liberia | 5.2 | 1.7 | 3.5 | 6.8 | 1.4 | 2.3 | 3.7 | 1.0 | 2.5 | 1.2 | 0.3 | 3.2 | -0.4 | -1.0 | 4.0 |
| Madagascar | 4.0 | 1.8 | 4.6 | 3.9 | 1.5 | 3.6 | 4.2 | 1.3 | 2.6 | 2.6 | 0.9 | 2.3 | 1.3 | 0.5 | 2.0 |
| Malawi | 2.4 | 0.9 | 1.9 | 2.1 | 0.8 | 1.7 | 2.0 | 0.7 | 1.7 | 1.7 | 0.6 | 1.5 | 0.8 | 0.4 | 1.5 |
| Maldives | -0.8 | -1.6 | 4.4 | -0.6 | -1.3 | 4.2 | 0.2 | -0.2 | 3.7 | -0.1 | -0.5 | 3.8 | 1.5 | 0.7 | 3.2 |
| Mali | 4.1 | 2.7 | 8.0 | 3.4 | 2.3 | 7.2 | 2.9 | 2.0 | 7.3 | 2.8 | 1.6 | 4.7 | 0.6 | 0.4 | 3.4 |
| Morocco | 4.3 | 1.0 | 1.9 | 2.9 | 0.7 | 1.7 | 2.4 | 0.6 | 1.3 | 1.9 | 0.5 | 1.1 | 1.2 | 0.4 | 1.3 |
| Mozambique | 3.5 | 1.9 | 4.9 | 1.7 | 1.1 | 5.2 | 1.8 | 1.1 | 4.1 | 1.0 | 0.6 | 3.0 | 0.3 | 0.2 | 2.4 |
| Namibia | 1.4 | 0.7 | 2.1 | 1.5 | 0.5 | 1.3 | 1.2 | 0.5 | 1.2 | 0.8 | 0.3 | 1.1 | 0.3 | 0.2 | 1.2 |
| Nepal | 5.0 | 1.9 | 3.4 | 4.1 | 1.6 | 3.0 | 3.7 | 1.4 | 2.7 | 3.3 | 1.1 | 2.2 | 1.5 | 0.7 | 2.0 |
| Nicaragua | -0.3 | -0.3 | 5.7 | 1.0 | -0.1 | 2.2 | 1.0 | -0.2 | 1.9 | -1.1 | -1.2 | 4.9 | -0.5 | -0.7 | 3.6 |
| Niger | 7.9 | 2.8 | 4.6 | 7.5 | 2.3 | 3.9 | 7.6 | 2.3 | 3.7 | 5.3 | 1.9 | 3.7 | 1.2 | 0.6 | 2.9 |
| Nigeria | -2.4 | -1.5 | 18.3 | 1.8 | 1.9 | 10.4 | 2.6 | 1.7 | 6.1 | 2.4 | 1.2 | 4.0 | 0.6 | 0.3 | 2.9 |
| Pakistan | 3.7 | 2.2 | 5.7 | 2.9 | 1.6 | 4.3 | 3.1 | 1.4 | 3.2 | 2.0 | 1.0 | 2.7 | 0.9 | 0.5 | 2.4 |
| Peru | 2.9 | 1.3 | 3.0 | 1.8 | 0.9 | 2.4 | 1.0 | 0.6 | 2.3 | 0.5 | 0.4 | 2.2 | 0.4 | 0.3 | 1.9 |
| Philippines | 1.8 | 1.1 | 3.7 | 1.4 | 0.8 | 2.7 | 0.7 | 0.5 | 2.5 | 0.5 | 0.4 | 2.4 | 0.2 | 0.2 | 2.5 |
| Rwanda | 2.0 | 1.1 | 3.7 | 2.3 | 1.1 | 3.1 | 2.0 | 1.0 | 3.0 | 2.2 | 1.0 | 2.7 | 1.5 | 0.7 | 2.3 |
| Senegal | 4.0 | 2.2 | 5.1 | 4.3 | 1.9 | 3.9 | 3.4 | 1.5 | 3.3 | 1.6 | 0.9 | 3.1 | 1.3 | 0.7 | 2.6 |
| Sierra Leone | 9.3 | 1.2 | 1.7 | 7.0 | 1.2 | 2.0 | 6.7 | 1.1 | 1.9 | 7.1 | 0.9 | 1.4 | 4.4 | 0.6 | 1.4 |
| South Africa | 0.4 | 0.2 | 2.1 | -0.1 | -0.3 | 2.3 | -0.3 | -0.6 | 2.2 | -0.3 | -0.5 | 1.9 | -0.5 | -1.0 | 2.6 |
| Tajikistan | 0.1 | -0.6 | 3.8 | 0.6 | -0.2 | 3.3 | -0.1 | -1.1 | 3.3 | 1.4 | 0.2 | 2.5 | 0.8 | -0.1 | 2.6 |
| Tanzania | 1.5 | 0.9 | 4.2 | 0.4 | 0.3 | 4.3 | 0.9 | 0.5 | 3.9 | 0.7 | 0.3 | 2.9 | 0.2 | 0.1 | 2.2 |
| Timor-Leste | 1.2 | 0.8 | 5.5 | 1.3 | 0.7 | 4.5 | 0.5 | 0.1 | 4.4 | 0.8 | 0.2 | 3.4 | 0.6 | -0.1 | 2.8 |
| Togo | 4.2 | 2.3 | 5.9 | 2.7 | 1.8 | 6.6 | 1.9 | 1.4 | 6.3 | 2.0 | 1.2 | 4.3 | 1.1 | 0.6 | 3.3 |
| Turkey | 0.9 | 0.5 | 2.2 | 0.7 | 0.4 | 2.2 | 0.8 | 0.4 | 2.0 | 0.7 | 0.4 | 2.0 | 0.9 | 0.4 | 1.8 |
| Uganda | 2.5 | 1.3 | 3.7 | 2.2 | 1.2 | 3.5 | 2.1 | 1.1 | 3.3 | 2.0 | 1.0 | 2.8 | 0.8 | 0.5 | 2.5 |
| Vietnam | 2.1 | 0.4 | 2.7 | 1.2 | 0.2 | 2.6 | 2.0 | 0.2 | 1.4 | 1.5 | 0.1 | 1.3 | 0.4 | -0.2 | 1.8 |
| Zambia | 1.5 | 0.9 | 3.5 | 1.1 | 0.6 | 3.5 | 1.2 | 0.6 | 3.0 | 1.0 | 0.5 | 1.9 | 0.3 | 0.1 | 1.8 |
| Zimbabwe | 0.1 | 0.0 | 3.7 | 0.1 | 0.0 | 3.3 | 0.3 | 0.2 | 2.7 | -0.1 | -0.2 | 2.6 | 0.0 | -0.1 | 1.8 |

**Note:** AARC is calculated as ln((rate in latest year/rate in earliest year))/ (latest year - earliest year), with positive values denote increasing rate while negative values denote decreasing rate

Table S25: Predicted average annual rate of increase (AARC) of composite coverage index in low- and middle-income countries by place of residence

| **Country** | **Rural** | | | **Urban** | | |
| --- | --- | --- | --- | --- | --- | --- |
|  | **Predicted** | | **Required** | **Predicted** | | **Required** |
|  | **1990-2018** | **2019-2030** | **2019-2030** | **1990-2018** | **2019-2030** | **2019-2030** |
| Armenia | 1.5 | 0.8 | 2.7 | 2.0 | 0.8 | 2.1 |
| Bangladesh | 2.2 | 1.1 | 2.9 | 1.3 | 0.7 | 2.0 |
| Benin | 0.5 | 0.5 | 6.2 | 0.1 | 0.1 | 5.2 |
| Bolivia | 4.3 | 1.3 | 2.4 | 1.7 | 0.7 | 2.1 |
| Brazil | 1.9 | 0.2 | 1.2 | 1.3 | 0.1 | 0.7 |
| Burkina Faso | 4.0 | 1.9 | 4.4 | 0.8 | 0.4 | 2.8 |
| Burundi | 0.2 | -0.2 | 4.2 | -0.4 | -1.1 | 3.9 |
| Cambodia | 5.2 | 1.1 | 1.7 | 3.0 | 0.9 | 1.9 |
| Cameroon | 0.8 | 0.7 | 7.1 | 0.8 | 0.6 | 3.5 |
| Chad | 4.8 | 3.6 | 10.4 | 1.4 | 1.1 | 6.6 |
| Colombia | 1.4 | 0.5 | 1.5 | 0.6 | 0.2 | 1.3 |
| Comoros | 1.2 | 0.8 | 4.3 | 1.1 | 0.6 | 3.2 |
| Congo | 1.0 | 0.7 | 5.7 | 1.3 | 0.4 | 3.2 |
| Congo DR | 2.0 | 1.5 | 6.6 | 1.4 | 0.8 | 4.8 |
| Cote d'Ivoire | 1.6 | 1.2 | 6.9 | 0.8 | 0.5 | 4.2 |
| Dominican Republic | 1.2 | 0.5 | 1.2 | 0.6 | 0.4 | 1.7 |
| Egypt | 1.5 | 0.7 | 1.8 | 0.6 | 0.4 | 1.7 |
| Ethiopia | 8.1 | 3.4 | 5.7 | 3.0 | 1.1 | 2.4 |
| Gabon | 1.0 | 0.8 | 6.0 | 0.0 | -0.1 | 4.9 |
| Ghana | 2.2 | 1.2 | 3.2 | 0.5 | 0.4 | 3.1 |
| Guatemala | 2.3 | 1.0 | 2.6 | 1.0 | 0.5 | 2.1 |
| Guinea | 0.2 | 0.4 | 8.7 | 0.1 | 0.0 | 4.2 |
| Haiti | 2.3 | 1.5 | 5.4 | 1.1 | 0.8 | 4.1 |
| Honduras | 3.4 | 0.5 | 1.1 | 1.1 | 0.1 | 1.5 |
| India | 2.2 | 1.0 | 2.7 | 0.9 | 0.5 | 1.9 |
| Indonesia | 0.8 | 0.6 | 2.9 | 0.0 | 0.0 | 2.7 |
| Jordan | 1.0 | 0.5 | 2.0 | 0.7 | 0.4 | 2.1 |
| Kazakhstan | 2.4 | 0.2 | 1.0 | 1.2 | 0.1 | 1.7 |
| Kenya | 1.0 | 0.6 | 3.0 | 0.4 | 0.2 | 2.1 |
| Kyrgyz Republic | 0.0 | -0.2 | 2.9 | -0.4 | -0.7 | 3.0 |
| Lesotho | 2.2 | 0.7 | 1.7 | 0.4 | 0.1 | 1.4 |
| Liberia | 4.6 | 1.4 | 3.0 | 0.1 | -0.4 | 3.9 |
| Madagascar | 3.1 | 1.4 | 3.5 | 2.4 | 0.9 | 1.9 |
| Malawi | 2.0 | 0.7 | 1.7 | 0.8 | 0.5 | 1.7 |
| Maldives | -0.5 | -1.0 | 4.2 | 1.1 | 0.4 | 3.1 |
| Mali | 3.7 | 2.3 | 6.6 | 1.1 | 0.7 | 3.3 |
| Morocco | 2.9 | 0.9 | 2.2 | 1.6 | 0.5 | 1.4 |
| Mozambique | 2.2 | 1.3 | 4.5 | 0.1 | 0.0 | 3.1 |
| Namibia | 1.2 | 0.6 | 1.6 | 0.5 | 0.2 | 1.2 |
| Nepal | 3.5 | 1.4 | 3.1 | 1.1 | 0.6 | 2.5 |
| Nicaragua | 0.5 | -0.2 | 3.6 | 0.0 | -0.4 | 2.9 |
| Niger | 6.9 | 2.4 | 4.0 | 1.1 | 0.6 | 2.7 |
| Nigeria | 0.6 | 0.6 | 9.9 | 0.5 | 0.4 | 4.9 |
| Pakistan | 2.9 | 1.6 | 4.0 | 1.2 | 0.7 | 2.9 |
| Peru | 2.7 | 1.1 | 2.6 | 0.9 | 0.5 | 2.2 |
| Philippines | 1.3 | 0.8 | 3.1 | 0.7 | 0.5 | 2.6 |
| Rwanda | 2.1 | 1.0 | 3.1 | 1.1 | 0.6 | 2.7 |
| Senegal | 2.8 | 1.6 | 4.4 | 1.1 | 0.7 | 3.0 |
| Sierra Leone | 7.6 | 1.1 | 1.8 | 5.2 | 0.7 | 1.4 |
| South Africa | 0.1 | -0.1 | 2.2 | -0.4 | -0.7 | 2.2 |
| Tajikistan | 0.3 | -0.5 | 3.3 | 1.1 | 0.1 | 2.6 |
| Tanzania | 0.8 | 0.5 | 4.0 | 0.3 | 0.1 | 2.4 |
| Timor-Leste | 0.9 | 0.4 | 4.4 | 0.5 | -0.1 | 3.3 |
| Togo | 2.8 | 1.8 | 6.2 | 0.9 | 0.6 | 4.1 |
| Turkey | 1.0 | 0.5 | 2.0 | 0.7 | 0.4 | 2.1 |
| Uganda | 2.0 | 1.1 | 3.4 | 0.6 | 0.4 | 2.6 |
| Vietnam | 1.6 | 0.2 | 2.2 | 0.2 | -0.2 | 2.4 |
| Yemen | 2.1 | 1.6 | 7.8 | 0.0 | -0.1 | 5.4 |
| Zambia | 1.4 | 0.8 | 3.1 | 0.7 | 0.4 | 1.9 |
| Zimbabwe | 0.1 | 0.0 | 3.2 | -0.2 | -0.3 | 2.4 |

**Note:** AARC is calculated as ln((rate in latest year/rate in earliest year))/ (latest year - earliest year), with positive values denote increasing rate while negative values denote decreasing rate

Table S26: Predicted average annual rate of increase (AARC) of composite coverage index in low- and middle-income countries by education of women

| **Country** | **Below secondary education** | | | **Secondary+ education** | | |
| --- | --- | --- | --- | --- | --- | --- |
|  | **Predicted** | | **Required** | **Predicted** | | **Required** |
|  | **1990-2018** | **2019-2030** | **2019-2030** | **1990-2018** | **2019-2030** | **2019-2030** |
| Armenia | N/A | N/A | N/A | 1.8 | 0.8 | 2.3 |
| Bangladesh | 1.9 | 1.1 | 3.5 | 1.1 | 0.6 | 2.3 |
| Benin | 0.3 | 0.3 | 6.2 | -0.2 | -0.3 | 4.1 |
| Bolivia | 3.9 | 1.2 | 2.3 | 1.3 | 0.6 | 2.1 |
| Brazil | 1.6 | 0.1 | 1.1 | 0.2 | -0.2 | 2.1 |
| Burkina Faso | 3.5 | 1.7 | 4.2 | 0.5 | 0.2 | 2.2 |
| Burundi | 0.1 | -0.3 | 4.3 | -0.3 | -0.9 | 3.4 |
| Cambodia | 5.3 | 1.1 | 1.8 | 1.9 | 0.8 | 2.0 |
| Cameroon | 0.6 | 0.6 | 7.1 | 0.9 | 0.6 | 2.9 |
| Chad | 3.3 | 2.7 | 10.4 | 0.2 | 0.2 | 6.1 |
| Colombia | 1.4 | 0.5 | 1.4 | 0.4 | 0.2 | 1.4 |
| Comoros | 1.3 | 0.8 | 4.4 | 0.5 | 0.3 | 3.5 |
| Congo | 1.7 | 1.0 | 5.0 | 1.3 | 0.5 | 3.4 |
| Congo DR | 1.6 | 1.4 | 6.9 | 0.7 | 0.5 | 5.2 |
| Cote d'Ivoire | 1.3 | 1.0 | 6.1 | 0.5 | 0.3 | 3.7 |
| Dominican Republic | 1.0 | 0.5 | 1.4 | 0.5 | 0.3 | 1.6 |
| Egypt | 1.3 | 0.7 | 2.1 | 0.3 | 0.3 | 2.0 |
| Ethiopia | 7.4 | 3.2 | 5.6 | 2.6 | 0.9 | 2.1 |
| Gabon | 0.6 | 0.4 | 5.6 | 0.0 | -0.1 | 4.9 |
| Ghana | 1.8 | 1.0 | 3.7 | 0.5 | 0.3 | 3.2 |
| Guatemala | 1.9 | 0.9 | 2.7 | 0.4 | 0.2 | 2.2 |
| Guinea | 0.0 | 0.1 | 8.1 | -0.3 | -0.4 | 3.9 |
| Haiti | 1.7 | 1.3 | 6.0 | 0.3 | 0.3 | 4.2 |
| Honduras | 2.8 | 0.4 | 1.2 | 0.7 | 0.0 | 1.6 |
| India | 2.1 | 1.1 | 3.1 | 0.6 | 0.4 | 2.1 |
| Indonesia | 0.7 | 0.5 | 3.3 | -0.1 | -0.2 | 2.8 |
| Jordan | 0.6 | 0.3 | 2.8 | 0.7 | 0.4 | 2.1 |
| Kazakhstan | N/A | N/A | N/A | 1.7 | 0.1 | 1.4 |
| Kenya | 1.1 | 0.7 | 3.0 | 0.6 | 0.3 | 1.9 |
| Kyrgyz Republic | N/A | N/A | N/A | -0.1 | -0.2 | 2.9 |
| Lesotho | 1.9 | 0.6 | 2.0 | 1.2 | 0.4 | 1.2 |
| Liberia | 3.2 | 1.2 | 3.4 | 0.7 | 0.0 | 3.1 |
| Madagascar | 3.2 | 1.5 | 3.7 | 2.2 | 0.8 | 1.8 |
| Malawi | 1.8 | 0.7 | 1.8 | 0.3 | 0.2 | 1.7 |
| Maldives | -0.2 | -0.6 | 3.7 | 0.2 | -0.1 | 3.9 |
| Mali | 2.6 | 1.7 | 6.4 | -0.2 | -0.2 | 3.6 |
| Morocco | 2.6 | 0.7 | 1.7 | 0.7 | 0.3 | 1.9 |
| Mozambique | 1.3 | 0.9 | 4.4 | -0.7 | -1.2 | 3.1 |
| Namibia | 0.9 | 0.5 | 2.2 | 0.4 | 0.2 | 1.3 |
| Nepal | 3.6 | 1.5 | 3.1 | 1.1 | 0.7 | 2.9 |
| Nicaragua | 0.3 | -0.2 | 3.6 | -0.3 | -0.6 | 3.4 |
| Niger | 5.1 | 2.1 | 4.1 | 0.5 | 0.3 | 2.5 |
| Nigeria | 0.0 | 0.1 | 11.6 | 0.1 | 0.1 | 4.1 |
| Pakistan | 2.3 | 1.4 | 4.4 | 0.7 | 0.5 | 2.6 |
| Peru | 2.2 | 1.1 | 2.8 | 0.7 | 0.5 | 2.3 |
| Philippines | 1.3 | 0.9 | 3.9 | 0.6 | 0.4 | 2.7 |
| Rwanda | 2.0 | 1.0 | 3.1 | 0.9 | 0.5 | 2.3 |
| Senegal | 2.2 | 1.3 | 4.0 | 0.3 | 0.2 | 2.9 |
| Sierra Leone | 6.6 | 1.2 | 2.0 | 5.1 | 0.6 | 1.2 |
| South Africa | -0.2 | -0.4 | 2.9 | -0.3 | -0.6 | 2.1 |
| Tajikistan | 0.1 | -0.5 | 4.0 | 0.5 | -0.2 | 3.1 |
| Tanzania | 0.6 | 0.4 | 3.7 | 0.1 | 0.0 | 2.4 |
| Timor-Leste | 0.9 | 0.5 | 4.6 | 0.1 | -0.4 | 3.8 |
| Togo | 2.4 | 1.6 | 5.9 | 0.6 | 0.4 | 4.0 |
| Turkey | 0.6 | 0.3 | 2.8 | 0.7 | 0.4 | 2.1 |
| Uganda | 2.0 | 1.1 | 3.5 | 0.7 | 0.4 | 2.6 |
| Vietnam | 1.6 | 0.3 | 2.9 | 1.4 | 0.1 | 1.6 |
| Yemen | 1.7 | 1.3 | 7.1 | -1.4 | -1.9 | 6.7 |
| Zambia | 1.1 | 0.7 | 3.1 | 0.2 | 0.1 | 2.3 |
| Zimbabwe | -0.2 | -0.2 | 3.9 | -0.3 | -0.4 | 2.7 |

**Note:** AARC is calculated as ln((rate in latest year/rate in earliest year))/ (latest year - earliest year), with positive values denote increasing rate while negative values denote decreasing rate; N/A denotes not application and refers that the indicator has no sufficient data to make projections

Table S27: Predicted average annual rate of increase (AARC) of composite coverage index in low- and middle-income countries by age of women

| **Country** | **Adolescent** | | | **Adult** | | |
| --- | --- | --- | --- | --- | --- | --- |
|  | **Predicted** | | **Required** | **Predicted** | | **Required** |
|  | **1990-2018** | **2019-2030** | **2019-2030** | **1990-2018** | **2019-2030** | **2020-2030** |
| Armenia | 3.1 | 0.7 | 1.4 | 1.7 | 0.8 | 2.3 |
| Bangladesh | 2.5 | 1.1 | 2.6 | 2.1 | 1.0 | 2.6 |
| Benin | 0.3 | 0.3 | 6.0 | 0.5 | 0.4 | 5.7 |
| Bolivia | 3.1 | 1.1 | 2.3 | 2.6 | 1.0 | 2.3 |
| Brazil | 1.5 | 0.2 | 1.2 | 1.8 | 0.1 | 0.5 |
| Burkina Faso | 3.6 | 1.5 | 3.7 | 3.4 | 1.6 | 4.0 |
| Burundi | 0.1 | -0.3 | 4.0 | 0.0 | -0.3 | 4.3 |
| Cambodia | 4.6 | 1.1 | 2.0 | 4.9 | 1.1 | 1.8 |
| Cameroon | 1.6 | 1.0 | 4.6 | 0.9 | 0.7 | 5.3 |
| Chad | 2.5 | 2.2 | 9.9 | 3.7 | 2.7 | 9.3 |
| Colombia | 1.3 | 0.5 | 1.5 | 0.9 | 0.3 | 1.2 |
| Comoros | 0.7 | 0.5 | 4.8 | 1.4 | 0.8 | 3.8 |
| Congo | 1.4 | 0.6 | 3.8 | 1.8 | 0.8 | 3.7 |
| Congo DR | 1.1 | 0.9 | 6.0 | 1.5 | 1.1 | 6.0 |
| Cote d'Ivoire | 1.8 | 1.2 | 5.5 | 1.2 | 0.9 | 5.8 |
| Dominican Republic | 1.6 | 0.7 | 1.8 | 0.8 | 0.4 | 1.4 |
| Egypt | 1.4 | 0.7 | 2.0 | 1.1 | 0.5 | 1.8 |
| Ethiopia | 6.7 | 2.9 | 5.3 | 7.0 | 3.0 | 5.3 |
| Gabon | 0.3 | 0.2 | 5.4 | 0.3 | 0.2 | 4.9 |
| Ghana | 1.5 | 0.9 | 3.4 | 1.7 | 1.0 | 3.0 |
| Guatemala | 1.9 | 1.0 | 3.1 | 1.7 | 0.8 | 2.4 |
| Guinea | -0.6 | -0.5 | 8.0 | 0.3 | 0.3 | 7.2 |
| Haiti | 1.5 | 1.1 | 5.7 | 1.9 | 1.3 | 4.9 |
| Honduras | 2.4 | 0.5 | 1.5 | 2.4 | 0.4 | 1.2 |
| India | 2.3 | 1.3 | 4.1 | 1.8 | 0.8 | 2.4 |
| Indonesia | 1.2 | 0.7 | 2.8 | 0.6 | 0.4 | 2.7 |
| Jordan | 0.7 | 0.4 | 2.5 | 0.4 | 0.3 | 2.4 |
| Kazakhstan | N/A | N/A | N/A | 1.8 | 0.1 | 1.3 |
| Kenya | 1.7 | 0.9 | 2.8 | 1.0 | 0.6 | 2.6 |
| Kyrgyz Republic | 1.7 | 0.5 | 1.1 | -0.1 | -0.3 | 2.9 |
| Lesotho | 2.3 | 0.7 | 1.6 | 1.9 | 0.6 | 1.5 |
| Liberia | 2.9 | 1.0 | 3.1 | 3.4 | 1.1 | 2.9 |
| Madagascar | 3.1 | 1.5 | 4.2 | 2.8 | 1.3 | 3.2 |
| Malawi | 1.7 | 0.8 | 2.1 | 1.8 | 0.7 | 1.6 |
| Maldives | 1.7 | 0.8 | 2.1 | -0.2 | -0.6 | 3.9 |
| Mali | 2.1 | 1.5 | 6.3 | 2.5 | 1.6 | 5.9 |
| Morocco | 2.9 | 0.6 | 1.1 | 2.5 | 0.7 | 1.6 |
| Mozambique | 1.8 | 1.0 | 3.6 | 1.5 | 0.9 | 3.9 |
| Namibia | 0.7 | 0.4 | 1.6 | 0.9 | 0.4 | 1.4 |
| Nepal | 3.5 | 1.6 | 3.7 | 3.5 | 1.3 | 2.7 |
| Nicaragua | 1.4 | 0.0 | 2.3 | -0.2 | -0.4 | 3.8 |
| Niger | 5.0 | 2.2 | 4.7 | 4.8 | 2.0 | 4.1 |
| Nigeria | 0.7 | 0.7 | 10.0 | 1.0 | 0.9 | 7.3 |
| Pakistan | 2.7 | 1.6 | 4.8 | 2.2 | 1.2 | 3.7 |
| Peru | 2.2 | 1.0 | 2.5 | 1.4 | 0.8 | 2.3 |
| Philippines | 1.3 | 0.9 | 3.9 | 1.0 | 0.6 | 2.8 |
| Rwanda | 1.5 | 0.9 | 3.8 | 1.9 | 1.0 | 3.0 |
| Senegal | 2.3 | 1.4 | 4.8 | 2.1 | 1.2 | 3.7 |
| Sierra Leone | 9.6 | 0.8 | 1.1 | 6.5 | 1.0 | 1.8 |
| South Africa | -0.3 | -0.6 | 2.5 | -0.2 | -0.3 | 2.1 |
| Tajikistan | -0.3 | -0.6 | 2.5 | 0.6 | -0.2 | 3.0 |
| Tanzania | 0.9 | 0.6 | 3.5 | 0.8 | 0.5 | 3.3 |
| Timor-Leste | 0.2 | 0.1 | 5.8 | 0.9 | 0.3 | 4.0 |
| Togo | 2.2 | 1.4 | 5.6 | 2.5 | 1.5 | 5.2 |
| Turkey | 2.2 | 1.4 | 5.6 | 2.5 | 1.5 | 5.2 |
| Uganda | 1.7 | 1.0 | 3.4 | 1.9 | 1.0 | 3.2 |
| Vietnam | 1.7 | 1.0 | 3.4 | 1.6 | 0.2 | 1.9 |
| Yemen | 1.1 | 1.0 | 7.9 | 1.9 | 1.4 | 6.3 |
| Zambia | 1.0 | 0.6 | 2.8 | 1.0 | 0.6 | 2.8 |
| Zimbabwe | 0.1 | 0.0 | 3.5 | 0.0 | -0.1 | 2.8 |

**Note:** AARC is calculated as ln((rate in latest year/rate in earliest year))/ (latest year - earliest year), with positive values denote increasing rate while negative values denote decreasing rate; N/A denotes not application and refers that the indicator has no sufficient data to make projections

Table S28: Predicted average annual rate of increase (AARC) of demand for family planning satisfied with modern contraceptive method among women 15-49 years of age in low- and middle-income countries

| **Country** | **Predicted** | | **Required** |
| --- | --- | --- | --- |
|  | **1990-2018** | **2019-2030** | **2019-2030** |
| Albania | -9.0 | -10.2 | 28.2 |
| Armenia | 2.7 | 2.0 | 7.3 |
| Bangladesh | 1.2 | 0.7 | 2.6 |
| Benin | 4.8 | 3.6 | 10.7 |
| Bolivia | 3.6 | 1.7 | 4.2 |
| Brazil | 1.3 | 0.1 | 0.6 |
| Burkina Faso | 7.8 | 2.5 | 4.0 |
| Burundi | 2.1 | 2.1 | 7.8 |
| Cambodia | 4.1 | 1.8 | 3.8 |
| Cameroon | 5.8 | 2.5 | 4.8 |
| Chad | 6.5 | 5.1 | 13.2 |
| Colombia | 1.0 | 0.4 | 1.2 |
| Comoros | 1.4 | 1.4 | 10.2 |
| Congo | 6.4 | 2.7 | 5.3 |
| Congo DR | 1.6 | 2.9 | 13.3 |
| Cote d'Ivoire | 5.2 | 2.8 | 7.0 |
| Dominican Republic | 0.7 | 0.4 | 1.3 |
| Egypt | 1.0 | 0.5 | 1.4 |
| Ethiopia | 9.5 | 2.1 | 2.9 |
| Gabon | 3.8 | 2.0 | 5.4 |
| Ghana | 3.1 | 2.1 | 7.0 |
| Guatemala | 2.0 | 1.0 | 3.3 |
| Guinea | 2.8 | 2.4 | 10.5 |
| Guyana | 18.5 | 0.2 | 0.2 |
| Haiti | 3.1 | 2.0 | 6.0 |
| Honduras | 1.8 | 0.4 | 2.0 |
| India | 0.8 | 0.5 | 2.6 |
| Indonesia | 0.3 | 0.2 | 2.1 |
| Jordan | 1.0 | 0.8 | 4.3 |
| Kazakhstan | 1.5 | 0.1 | 2.1 |
| Kenya | 3.2 | 1.0 | 2.1 |
| Kyrgyz Republic | -1.2 | -1.7 | 6.1 |
| Lesotho | 4.8 | 0.9 | 1.3 |
| Liberia | 33.8 | 0.1 | 0.1 |
| Madagascar | 7.5 | 1.7 | 2.5 |
| Malawi | 5.6 | 1.3 | 2.1 |
| Maldives | -4.9 | -8.0 | 15.0 |
| Mali | 3.4 | 2.6 | 10.0 |
| Morocco | 1.9 | 0.5 | 1.3 |
| Mozambique | 4.1 | 2.0 | 4.6 |
| Namibia | 1.4 | 0.6 | 1.4 |
| Nepal | 1.2 | 0.8 | 4.4 |
| Nicaragua | 1.5 | 0.0 | 1.5 |
| Niger | 5.9 | 3.0 | 6.3 |
| Nigeria | 3.8 | 2.3 | 6.5 |
| Pakistan | 3.1 | 2.0 | 5.9 |
| Peru | 1.5 | 0.9 | 3.5 |
| Philippines | 1.8 | 1.3 | 5.1 |
| Rwanda | 6.2 | 2.1 | 3.8 |
| Senegal | 4.9 | 2.7 | 6.2 |
| Sierra Leone | 13.4 | 1.8 | 2.2 |
| South Africa | -0.1 | -0.3 | 2.5 |
| Tajikistan | 0.4 | 0.7 | 7.1 |
| Tanzania | 3.6 | 1.8 | 4.3 |
| Timor-Leste | -0.7 | 0.0 | 8.9 |
| Togo | 5.6 | 3.1 | 6.9 |
| Turkey | 1.2 | 0.9 | 4.4 |
| Uganda | 4.3 | 2.2 | 5.0 |
| Vietnam | -0.1 | -0.3 | 4.2 |
| Yemen | 3.0 | 2.5 | 11.1 |
| Zambia | 4.6 | 1.6 | 3.0 |
| Zimbabwe | 1.3 | 0.5 | 1.4 |

**Note:** AARC is calculated as ln((rate in latest year/rate in earliest year))/ (latest year - earliest year), with positive values denote increasing rate while negative values denote decreasing rate

Table S29: Predicted average annual rate of increase (AARC) of demand for family planning satisfied with modern contraceptive method among women 15-49 years of age in low- and middle-income countries by wealth quintiles

| **Country** | **Poorest** | | | **Poorer** | | | | **Middle** | | | **Richer** | | | | **Richest** | | | |
| --- | --- | --- | --- | --- | --- | --- | --- | --- | --- | --- | --- | --- | --- | --- | --- | --- | --- | --- |
|  | **Predicted** | | **Required** | **Predicted** | | **Required** | | **Predicted** | | **Required** | **Predicted** | | **Required** | **Predicted** | | | **Required** |  |
|  | **1990-2018** | **2019-2030** | **2019-2030** | **1990-2018** | **2019-2030** | | **2019-2030** | **1990-2018** | **2019-2030** | **2019-2030** | **1990-2018** | **2019-2030** | **2019-2030** | **1990-2018** | | **2019-2030** | **2019-2030** |  |
| Albania | -9.0 | -9.8 | 28.5 | -8.2 | -8.0 | | 28.6 | -9.7 | -11.2 | 29.9 | -7.8 | -7.7 | 27.3 | -9.3 | | -13.2 | 27.0 |  |
| Armenia | 3.4 | 2.7 | 9.5 | 2.1 | 1.8 | | 9.5 | 2.4 | 1.9 | 7.7 | 3.3 | 2.0 | 5.8 | 2.2 | | 1.4 | 5.4 |  |
| Bangladesh | 1.4 | 0.8 | 2.5 | 1.5 | 0.8 | | 2.3 | 1.2 | 0.7 | 2.6 | 1.5 | 0.8 | 2.6 | 0.7 | | 0.5 | 2.9 |  |
| Benin | 6.6 | 5.1 | 13.0 | 8.2 | 5.5 | | 11.4 | 6.9 | 4.6 | 10.4 | 4.1 | 3.2 | 10.9 | 3.4 | | 2.5 | 8.6 |  |
| Bolivia | 13.0 | 1.6 | 2.0 | 7.1 | 2.0 | | 3.4 | 4.5 | 1.9 | 4.2 | 3.2 | 1.3 | 3.4 | 0.9 | | 0.5 | 4.0 |  |
| Burkina Faso | 13.9 | 3.9 | 5.1 | 14.5 | 2.8 | | 3.4 | 12.3 | 2.9 | 3.9 | 11.5 | 2.2 | 2.9 | 4.1 | | 1.1 | 2.1 |  |
| Burundi | 4.0 | 3.1 | 7.7 | 4.8 | 3.5 | | 7.6 | 2.4 | 2.3 | 7.8 | 0.6 | 1.3 | 9.1 | 0.8 | | 0.8 | 6.7 |  |
| Cambodia | 7.3 | 1.9 | 2.9 | 6.6 | 1.7 | | 2.6 | 3.9 | 1.7 | 3.7 | 3.4 | 1.7 | 4.0 | 0.8 | | 0.7 | 6.3 |  |
| Cameroon | 6.0 | 4.8 | 14.2 | 8.5 | 3.9 | | 6.7 | 6.4 | 2.9 | 5.6 | 6.3 | 2.0 | 3.4 | 4.8 | | 1.6 | 3.0 |  |
| Chad | 24.2 | 10.9 | 13.1 | 12.2 | 8.3 | | 14.7 | 15.4 | 8.2 | 11.6 | 5.6 | 5.6 | 18.6 | 4.0 | | 2.9 | 8.9 |  |
| Colombia | 2.1 | 0.7 | 1.4 | 1.1 | 0.4 | | 1.2 | 0.9 | 0.4 | 1.1 | 0.7 | 0.3 | 1.0 | 0.7 | | 0.3 | 1.0 |  |
| Comoros | 3.5 | 3.0 | 12.0 | 1.6 | 1.6 | | 10.7 | 1.8 | 1.6 | 9.7 | 3.0 | 2.1 | 7.8 | -1.1 | | -0.9 | 11.5 |  |
| Congo | 0.8 | 2.2 | 13.9 | 6.7 | 3.3 | | 6.6 | 9.8 | 2.6 | 4.0 | 7.2 | 2.3 | 4.1 | 5.6 | | 1.9 | 3.8 |  |
| Congo DR | 0.2 | 3.2 | 19.9 | 1.2 | 3.5 | | 17.1 | -1.0 | 1.9 | 18.5 | 4.1 | 3.7 | 9.9 | 1.5 | | 2.0 | 9.6 |  |
| Cote d'Ivoire | 9.4 | 5.5 | 10.3 | 6.6 | 4.0 | | 9.0 | 8.4 | 3.6 | 6.3 | 5.0 | 2.5 | 6.1 | 3.2 | | 1.8 | 5.3 |  |
| Dominican Republic | 1.1 | 0.5 | 1.4 | 0.6 | 0.3 | | 1.3 | 0.2 | 0.1 | 1.6 | 0.2 | 0.1 | 1.8 | 0.4 | | 0.3 | 1.3 |  |
| Egypt | 2.0 | 0.7 | 1.6 | 1.0 | 0.5 | | 1.7 | 0.8 | 0.4 | 1.4 | 0.5 | 0.3 | 1.4 | 0.2 | | 0.1 | 1.5 |  |
| Ethiopia | 13.1 | 4.0 | 5.2 | 14.1 | 2.6 | | 3.1 | 14.9 | 2.0 | 2.3 | 13.5 | 1.6 | 1.9 | 4.7 | | 1.1 | 1.8 |  |
| Gabon | 5.1 | 3.2 | 8.3 | 4.5 | 2.4 | | 6.1 | 2.5 | 1.7 | 6.9 | 5.1 | 2.0 | 4.1 | 3.3 | | 1.5 | 4.0 |  |
| Ghana | 5.8 | 3.2 | 7.1 | 4.7 | 2.6 | | 6.3 | 4.2 | 2.4 | 6.1 | 2.2 | 1.7 | 7.4 | 1.1 | | 0.9 | 7.3 |  |
| Guatemala | 7.6 | 2.9 | 4.9 | 5.3 | 2.1 | | 3.8 | 3.0 | 1.3 | 3.1 | 1.4 | 0.7 | 2.6 | 0.3 | | 0.2 | 2.3 |  |
| Guinea | 7.3 | 5.4 | 12.4 | 4.3 | 3.8 | | 13.6 | 3.9 | 3.4 | 12.2 | -0.5 | 0.1 | 13.7 | 3.1 | | 1.8 | 6.0 |  |
| Guyana | 14.0 | 0.7 | 1.0 | 17.3 | 0.3 | | 0.3 | 21.6 | 0.1 | 0.1 | 19.7 | 0.2 | 0.2 | 21.3 | | 0.1 | 0.1 |  |
| Haiti | 5.6 | 3.0 | 6.6 | 4.4 | 2.5 | | 6.2 | 3.7 | 2.0 | 5.2 | 1.9 | 1.4 | 6.2 | 2.0 | | 1.4 | 5.8 |  |
| Honduras | 4.5 | 1.0 | 2.1 | 2.6 | 0.6 | | 1.9 | 1.6 | 0.3 | 1.8 | 1.2 | 0.2 | 1.8 | 0.6 | | -0.1 | 2.2 |  |
| India | 1.2 | 0.8 | 3.9 | 1.4 | 0.8 | | 2.7 | 0.8 | 0.5 | 2.4 | 0.6 | 0.4 | 2.3 | 0.3 | | 0.3 | 2.4 |  |
| Indonesia | 0.6 | 0.4 | 2.2 | 0.2 | 0.2 | | 1.9 | 0.0 | 0.0 | 2.1 | -0.1 | -0.1 | 2.3 | -0.4 | | -0.5 | 3.0 |  |
| Jordan | 2.3 | 1.4 | 4.5 | 1.7 | 1.1 | | 4.0 | 1.1 | 0.8 | 4.4 | 0.7 | 0.5 | 4.2 | 0.4 | | 0.3 | 4.1 |  |
| Kazakhstan | 0.4 | 0.0 | 4.1 | -1.1 | -0.8 | | 6.1 | 3.0 | 0.2 | 0.9 | 2.1 | 0.2 | 1.5 | 1.8 | | 0.1 | 1.4 |  |
| Kenya | 5.7 | 2.2 | 4.2 | 4.8 | 1.3 | | 2.3 | 3.5 | 1.0 | 1.9 | 2.7 | 0.8 | 1.6 | 1.5 | | 0.6 | 1.9 |  |
| Kyrgyz Republic | -0.2 | -0.3 | 4.6 | -0.8 | -1.0 | | 5.2 | -1.8 | -2.8 | 7.2 | -2.0 | -3.0 | 7.7 | -1.2 | | -1.6 | 5.9 |  |
| Lesotho | 10.4 | 1.4 | 1.8 | 8.0 | 1.2 | | 1.5 | 6.5 | 0.8 | 1.1 | 4.0 | 0.8 | 1.3 | 1.3 | | 0.4 | 1.2 |  |
| Liberia | 40.5 | 0.1 | 0.1 | 44.1 | 0.0 | | 0.0 | 35.5 | 0.1 | 0.1 | 30.0 | 0.1 | 0.1 | 32.9 | | 0.0 | 0.0 |  |
| Madagascar | 13.9 | 1.4 | 1.7 | 10.9 | 1.5 | | 1.9 | 8.6 | 1.4 | 2.0 | 6.8 | 1.4 | 2.2 | 2.9 | | 1.5 | 4.6 |  |
| Malawi | 7.7 | 1.6 | 2.2 | 7.9 | 1.3 | | 1.8 | 6.5 | 1.3 | 2.0 | 5.6 | 1.2 | 1.9 | 3.2 | | 1.0 | 1.9 |  |
| Maldives | -5.4 | -9.7 | 15.9 | -4.4 | -6.7 | | 14.0 | -5.2 | -9.2 | 15.6 | -5.8 | -10.2 | 17.1 | -3.5 | | -4.9 | 12.3 |  |
| Mali | 8.1 | 5.8 | 12.9 | 5.9 | 4.5 | | 12.4 | 4.8 | 3.9 | 13.2 | 4.7 | 3.1 | 8.3 | 2.3 | | 1.5 | 6.0 |  |
| Morocco | 4.5 | 0.3 | 0.5 | 2.4 | 0.4 | | 0.9 | 1.5 | 0.5 | 1.5 | 1.1 | 0.4 | 1.9 | 0.7 | | 0.3 | 2.3 |  |
| Mozambique | 8.4 | 3.7 | 6.6 | 4.6 | 2.9 | | 7.7 | 3.8 | 2.3 | 6.6 | 5.3 | 2.0 | 3.9 | 2.5 | | 1.1 | 2.8 |  |
| Namibia | 3.9 | 1.1 | 2.1 | 4.3 | 0.8 | | 1.2 | 3.2 | 0.7 | 1.2 | 1.0 | 0.4 | 1.2 | 0.5 | | 0.2 | 0.9 |  |
| Nepal | 3.1 | 1.7 | 4.6 | 2.2 | 1.3 | | 4.0 | 1.7 | 1.0 | 4.0 | 1.0 | 0.7 | 4.3 | -0.8 | | -1.1 | 5.4 |  |
| Nicaragua | 2.6 | 0.2 | 2.1 | 2.1 | 0.0 | | 1.2 | 2.3 | 0.0 | 0.7 | 0.3 | -0.3 | 2.2 | 0.8 | | -0.2 | 1.7 |  |
| Niger | 14.9 | 3.9 | 5.0 | 9.4 | 4.2 | | 7.4 | 6.9 | 3.9 | 8.4 | 8.2 | 3.1 | 5.3 | 2.0 | | 1.1 | 4.9 |  |
| Nigeria | 3.4 | 3.6 | 17.3 | 4.9 | 3.6 | | 10.5 | 5.3 | 2.9 | 6.7 | 4.5 | 2.3 | 5.4 | 2.3 | | 1.4 | 4.6 |  |
| Pakistan | 8.5 | 3.8 | 6.2 | 5.3 | 2.8 | | 6.0 | 4.2 | 2.3 | 5.5 | 3.1 | 1.8 | 5.1 | 1.1 | | 0.9 | 5.7 |  |
| Peru | 4.1 | 1.9 | 4.3 | 1.9 | 1.2 | | 4.2 | 1.2 | 0.8 | 3.5 | 0.9 | 0.6 | 3.2 | 0.9 | | 0.6 | 2.6 |  |
| Philippines | 3.8 | 2.1 | 5.0 | 2.6 | 1.5 | | 4.4 | 1.7 | 1.2 | 4.8 | 1.0 | 0.8 | 5.3 | 0.4 | | 0.4 | 6.1 |  |
| Rwanda | 7.9 | 2.7 | 4.4 | 7.1 | 2.4 | | 4.1 | 7.4 | 2.2 | 3.5 | 6.2 | 2.2 | 3.9 | 4.0 | | 1.5 | 3.2 |  |
| Senegal | 12.3 | 5.8 | 8.4 | 10.7 | 4.6 | | 6.9 | 7.7 | 3.2 | 5.3 | 5.1 | 2.3 | 4.7 | 2.3 | | 1.4 | 4.5 |  |
| Sierra Leone | 22.4 | 1.8 | 1.9 | 21.0 | 2.0 | | 2.2 | 21.8 | 1.8 | 2.0 | 14.6 | 1.5 | 1.7 | 8.5 | | 1.3 | 1.9 |  |
| South Africa | 1.2 | 0.6 | 2.4 | 0.4 | 0.2 | | 2.4 | -0.1 | -0.2 | 2.2 | -0.4 | -0.7 | 2.3 | -1.1 | | -2.9 | 3.6 |  |
| Tajikistan | 0.6 | 1.1 | 7.6 | -0.6 | -0.2 | | 8.2 | 0.6 | 1.0 | 7.2 | 2.1 | 1.8 | 6.2 | -0.1 | | 0.0 | 6.4 |  |
| Tanzania | 6.2 | 3.0 | 5.7 | 4.1 | 2.2 | | 5.4 | 4.9 | 2.1 | 4.2 | 3.1 | 1.5 | 3.6 | 0.4 | | 0.3 | 4.7 |  |
| Timor-Leste | 3.3 | 2.9 | 7.8 | 0.7 | 1.4 | | 8.9 | 2.6 | 2.4 | 7.5 | -1.7 | -1.7 | 9.1 | -2.6 | | -3.8 | 9.6 |  |
| Togo | 9.6 | 4.5 | 7.4 | 7.5 | 3.8 | | 7.2 | 5.7 | 3.4 | 7.8 | 5.5 | 3.0 | 6.7 | 3.6 | | 2.1 | 5.7 |  |
| Turkey | 2.6 | 1.6 | 5.2 | 1.5 | 1.0 | | 5.0 | 1.1 | 0.8 | 4.7 | 1.1 | 0.8 | 3.9 | 0.5 | | 0.4 | 3.7 |  |
| Uganda | 6.8 | 3.8 | 7.8 | 7.9 | 3.2 | | 5.3 | 6.7 | 2.7 | 4.7 | 6.1 | 2.1 | 3.7 | 1.3 | | 0.8 | 3.8 |  |
| Vietnam | 1.6 | 0.2 | 2.0 | -0.2 | -0.4 | | 4.1 | -1.3 | -1.2 | 6.1 | -0.7 | -0.7 | 5.2 | -0.6 | | -0.5 | 5.4 |  |
| Yemen |  |  |  |  |  | |  |  |  |  |  |  |  |  | |  |  |  |
| Zambia | 7.6 | 2.3 | 3.6 | 5.8 | 2.2 | | 4.1 | 6.3 | 1.9 | 3.1 | 3.8 | 1.3 | 2.6 | 2.2 | | 0.8 | 1.9 |  |
| Zimbabwe | 2.3 | 0.8 | 1.8 | 2.0 | 0.7 | | 1.6 | 1.8 | 0.6 | 1.2 | 0.7 | 0.4 | 1.3 | 0.6 | | 0.3 | 1.0 |  |

**Note:** AARC is calculated as ln((rate in latest year/rate in earliest year))/ (latest year - earliest year), with positive values denote increasing rate while negative values denote decreasing rate

Table S30: Predicted average annual rate of increase (AARC) of demand for family planning satisfied with modern contraceptive method among women 15-49 years of age in low- and middle-income countries by place of residence

| **Country** | **Rural** | | | **Urban** | | |
| --- | --- | --- | --- | --- | --- | --- |
|  | **Predicted** | | **Required** | **Predicted** | | **Required** |
|  | **1990-2018** | **2019-2030** | **2019-2030** | **1990-2018** | **2019-2030** | **2019-2030** |
| Albania | -8.8 | -9.5 | 28.7 | -9.2 | -11.1 | 27.9 |
| Armenia | 2.3 | 2.0 | 9.6 | 2.9 | 1.8 | 6.0 |
| Bangladesh | 1.3 | 0.8 | 2.7 | 0.8 | 0.5 | 2.5 |
| Benin | 6.0 | 4.4 | 11.5 | 3.6 | 2.8 | 9.7 |
| Bolivia | 7.5 | 2.3 | 3.9 | 2.6 | 1.3 | 3.9 |
| Brazil | 1.7 | 0.2 | 1.1 | 0.9 | 0.1 | 0.8 |
| Burkina Faso | 11.4 | 3.2 | 4.3 | 3.5 | 1.1 | 2.4 |
| Burundi | 2.8 | 2.5 | 7.9 | 0.3 | 0.3 | 6.1 |
| Cambodia | 4.7 | 1.8 | 3.4 | 1.1 | 0.9 | 6.3 |
| Cameroon | 5.9 | 3.5 | 7.9 | 5.2 | 1.9 | 3.7 |
| Chad | 11.6 | 8.1 | 15.0 | 4.2 | 3.0 | 9.0 |
| Colombia | 1.5 | 0.6 | 1.3 | 0.8 | 0.4 | 1.1 |
| Comoros | 0.7 | 1.0 | 12.8 | 1.8 | 1.4 | 7.3 |
| Congo | 2.4 | 2.8 | 11.3 | 7.0 | 2.3 | 4.1 |
| Congo DR | 1.7 | 3.9 | 16.5 | 2.6 | 2.8 | 9.8 |
| Cote d'Ivoire | 6.4 | 3.7 | 8.6 | 4.0 | 2.2 | 6.0 |
| Dominican Republic | 1.1 | 0.4 | 0.9 | 0.5 | 0.3 | 1.5 |
| Egypt | 1.4 | 0.6 | 1.4 | 0.6 | 0.3 | 1.4 |
| Ethiopia | 12.6 | 2.4 | 3.0 | 3.5 | 0.9 | 1.7 |
| Gabon | 4.5 | 3.2 | 8.9 | 3.5 | 1.9 | 5.3 |
| Ghana | 4.3 | 2.6 | 6.6 | 1.5 | 1.2 | 7.6 |
| Guatemala | 3.2 | 1.5 | 3.8 | 1.1 | 0.6 | 2.7 |
| Guinea | 3.6 | 3.4 | 13.7 | 2.1 | 1.6 | 7.4 |
| Guyana | 18.1 | 0.2 | 0.3 | 19.2 | 0.1 | 0.2 |
| Haiti | 3.7 | 2.2 | 6.2 | 2.3 | 1.6 | 5.8 |
| Honduras | 2.9 | 0.7 | 1.9 | 1.1 | 0.1 | 1.9 |
| India | 0.9 | 0.6 | 2.8 | 0.5 | 0.3 | 2.4 |
| Indonesia | 0.4 | 0.3 | 1.8 | 0.1 | 0.1 | 2.5 |
| Jordan | 2.0 | 1.3 | 4.6 | 0.8 | 0.6 | 4.4 |
| Kazakhstan | 1.2 | 0.1 | 2.7 | 1.8 | 0.2 | 1.7 |
| Kenya | 3.5 | 1.2 | 2.4 | 1.7 | 0.7 | 1.9 |
| Kyrgyz Republic | -1.1 | -1.4 | 6.0 | -1.4 | -2.1 | 6.2 |
| Lesotho | 5.8 | 1.0 | 1.6 | 1.4 | 0.4 | 1.3 |
| Liberia | 39.4 | 0.1 | 0.1 | 28.2 | 0.1 | 0.1 |
| Madagascar | 9.7 | 1.4 | 1.9 | 3.8 | 1.7 | 4.0 |
| Malawi | 6.2 | 1.4 | 2.0 | 3.2 | 1.0 | 2.0 |
| Maldives | -5.2 | -8.9 | 15.5 | -4.5 | -7.0 | 14.2 |
| Mali | 5.9 | 4.3 | 11.1 | 2.8 | 1.8 | 6.2 |
| Morocco | 2.8 | 0.5 | 0.9 | 0.9 | 0.3 | 2.1 |
| Mozambique | 5.9 | 2.7 | 5.6 | 2.3 | 1.2 | 3.6 |
| Namibia | 2.7 | 0.8 | 1.7 | 0.6 | 0.3 | 1.3 |
| Nepal | 1.3 | 0.9 | 4.6 | -0.6 | -0.8 | 4.8 |
| Nicaragua | 2.4 | 0.1 | 1.4 | 1.2 | -0.1 | 1.4 |
| Niger | 9.7 | 3.9 | 6.3 | 2.7 | 1.4 | 4.3 |
| Nigeria | 4.9 | 3.1 | 7.9 | 2.1 | 1.4 | 5.6 |
| Pakistan | 4.7 | 2.6 | 5.9 | 1.7 | 1.2 | 5.6 |
| Peru | 3.3 | 1.6 | 4.0 | 1.1 | 0.7 | 3.1 |
| Philippines | 2.5 | 1.5 | 4.7 | 1.2 | 1.0 | 5.4 |
| Rwanda | 6.8 | 2.3 | 3.9 | 3.3 | 1.5 | 3.7 |
| Senegal | 9.1 | 4.6 | 7.6 | 3.3 | 1.8 | 4.5 |
| Sierra Leone | 19.8 | 1.9 | 2.1 | 10.6 | 1.3 | 1.7 |
| South Africa | 0.6 | 0.3 | 2.3 | -0.5 | -0.9 | 2.7 |
| Tajikistan | 0.1 | 0.5 | 7.4 | 1.1 | 1.1 | 6.2 |
| Tanzania | 5.1 | 2.2 | 4.3 | 1.3 | 0.9 | 4.1 |
| Timor-Leste | 0.8 | 1.2 | 8.1 | -2.8 | -3.7 | 10.4 |
| Togo | 6.9 | 3.8 | 7.6 | 4.0 | 2.3 | 6.3 |
| Turkey | 1.6 | 1.1 | 5.4 | 0.8 | 0.6 | 4.3 |
| Uganda | 5.7 | 2.6 | 5.2 | 0.8 | 0.5 | 4.0 |
| Vietnam | -0.4 | -0.5 | 4.7 | 0.7 | 0.1 | 3.4 |
| Yemen | 4.9 | 3.7 | 11.4 | -0.1 | 0.0 | 10.9 |
| Zambia | 7.2 | 2.0 | 3.0 | 3.4 | 1.1 | 2.2 |
| Zimbabwe | 1.8 | 0.6 | 1.5 | 0.6 | 0.3 | 1.3 |

**Note:** AARC is calculated as ln((rate in latest year/rate in earliest year))/ (latest year - earliest year), with positive values denote increasing rate while negative values denote decreasing rate

Table S31: Predicted average annual rate of increase (AARC) of demand for family planning satisfied with modern contraceptive method among women 15-49 years of age in low- and middle-income countries by education

| **Country** | **Below secondary education** | | | **Secondary+ education** | | |
| --- | --- | --- | --- | --- | --- | --- |
|  | **Predicted** | | **Required** | **Predicted** | | **Required** |
|  | **1990-2018** | **2019-2030** | **2019-2030** | **1990-2018** | **2019-2030** | **2019-2030** |
| Albania | -8.5 | -8.2 | 28.7 | -9.1 | -11.6 | 27.4 |
| Armenia | 1.1 | 1.2 | 9.5 | 2.9 | 2.0 | 7.1 |
| Bangladesh | 1.3 | 0.8 | 2.6 | 1.0 | 0.6 | 2.7 |
| Benin | 5.1 | 4.0 | 11.9 | 2.6 | 1.9 | 7.7 |
| Bolivia | 6.2 | 2.0 | 3.7 | 2.0 | 1.1 | 4.0 |
| Brazil | 1.2 | 0.1 | 1.0 | -0.1 | -0.3 | 2.2 |
| Burkina Faso | 9.0 | 2.9 | 4.5 | 2.5 | 0.7 | 1.6 |
| Burundi | 2.5 | 2.3 | 8.0 | 0.2 | 0.1 | 6.2 |
| Cambodia | 4.7 | 1.8 | 3.5 | 2.3 | 1.4 | 4.9 |
| Cameroon | 5.0 | 3.2 | 8.6 | 5.7 | 1.7 | 3.0 |
| Chad | 7.0 | 5.9 | 15.4 | 2.1 | 1.6 | 7.7 |
| Colombia | 1.2 | 0.5 | 1.2 | 0.8 | 0.4 | 1.2 |
| Comoros | 1.1 | 1.3 | 11.5 | 0.3 | 0.4 | 9.7 |
| Congo | 7.1 | 3.9 | 7.7 | 5.8 | 2.3 | 4.8 |
| Congo DR | 0.5 | 3.0 | 17.7 | 1.8 | 2.4 | 10.7 |
| Cote d'Ivoire | 5.4 | 3.2 | 8.0 | 3.4 | 1.7 | 4.8 |
| Dominican Republic | 0.8 | 0.3 | 1.0 | 0.5 | 0.3 | 1.6 |
| Egypt | 1.2 | 0.5 | 1.5 | 0.4 | 0.3 | 1.5 |
| Ethiopia | 10.8 | 2.3 | 3.0 | 3.4 | 0.9 | 1.7 |
| Gabon | 3.6 | 2.6 | 8.7 | 3.3 | 1.8 | 5.0 |
| Ghana | 3.8 | 2.5 | 7.5 | 0.6 | 0.6 | 8.1 |
| Guatemala | 2.5 | 1.3 | 3.6 | 0.4 | 0.2 | 3.0 |
| Guinea | 2.3 | 2.5 | 13.2 | 1.0 | 0.8 | 6.4 |
| Guyana | 17.1 | 0.3 | 0.4 | 19.2 | 0.2 | 0.2 |
| Haiti | 3.3 | 2.1 | 6.5 | 1.6 | 1.2 | 5.9 |
| Honduras | 2.2 | 0.5 | 1.9 | 0.9 | 0.1 | 2.2 |
| India | 1.1 | 0.6 | 2.2 | 0.5 | 0.4 | 3.1 |
| Indonesia | 0.5 | 0.3 | 1.9 | 0.0 | 0.0 | 2.3 |
| Jordan | 0.9 | 0.7 | 5.3 | 0.9 | 0.7 | 4.3 |
| Kazakhstan | N/A | N/A | N/a | 1.4 | 0.1 | 2.3 |
| Kenya | 3.8 | 1.2 | 2.4 | 2.0 | 0.7 | 1.7 |
| Kyrgyz Republic | 17.9 | 0.0 | 0.0 | -1.2 | -1.7 | 6.1 |
| Lesotho | 5.6 | 1.1 | 1.8 | 2.8 | 0.6 | 1.2 |
| Liberia | 37.7 | 0.1 | 0.1 | 26.1 | 0.1 | 0.1 |
| Madagascar | 9.9 | 1.4 | 1.8 | 4.3 | 1.8 | 3.8 |
| Malawi | 6.1 | 1.4 | 2.0 | 1.1 | 0.7 | 2.9 |
| Maldives | -4.9 | -9.7 | 14.4 | -4.0 | -4.4 | 14.9 |
| Mali | 4.0 | 3.1 | 11.0 | 0.6 | 0.5 | 6.3 |
| Morocco | 2.1 | 0.5 | 1.1 | 0.5 | 0.2 | 2.5 |
| Mozambique | 3.8 | 2.2 | 6.0 | 0.4 | 0.2 | 3.4 |
| Namibia | 1.8 | 0.9 | 2.5 | 0.8 | 0.4 | 1.3 |
| Nepal | 2.0 | 1.1 | 3.3 | -1.0 | -1.1 | 7.5 |
| Nicaragua | 2.2 | 0.0 | 1.2 | 0.4 | -0.3 | 2.4 |
| Niger | 6.4 | 3.2 | 6.7 | 0.9 | 0.6 | 4.1 |
| Nigeria | 3.7 | 2.9 | 10.7 | 1.9 | 1.3 | 5.4 |
| Pakistan | 3.7 | 2.2 | 6.0 | 1.1 | 0.9 | 6.0 |
| Peru | 2.3 | 1.3 | 4.2 | 0.9 | 0.6 | 3.3 |
| Philippines | 2.4 | 1.5 | 5.1 | 1.5 | 1.1 | 5.2 |
| Rwanda | 6.8 | 2.3 | 3.9 | 2.7 | 1.3 | 3.4 |
| Senegal | 6.0 | 3.2 | 6.5 | 0.8 | 0.7 | 5.1 |
| Sierra Leone | 13.9 | 2.4 | 3.0 | 11.0 | 1.1 | 1.4 |
| South Africa | 0.0 | -0.1 | 3.7 | -0.4 | -0.7 | 2.5 |
| Tajikistan | -0.9 | -0.2 | 8.9 | 0.3 | 0.6 | 7.1 |
| Tanzania | 3.8 | 1.9 | 4.3 | 0.7 | 0.5 | 4.8 |
| Timor-Leste | 0.2 | 0.8 | 8.5 | -1.5 | -1.2 | 9.2 |
| Togo | 6.2 | 3.6 | 8.0 | 2.9 | 1.8 | 5.7 |
| Turkey | 1.4 | 1.0 | 4.7 | 0.0 | 0.0 | 4.5 |
| Uganda | 5.2 | 2.6 | 5.6 | 1.1 | 0.7 | 4.2 |
| Vietnam | 1.5 | 0.2 | 2.2 | -0.8 | -0.8 | 5.5 |
| Yemen | 3.7 | 2.8 | 10.1 | -4.5 | -5.3 | 18.1 |
| Zambia | 6.0 | 1.9 | 3.1 | 2.0 | 1.0 | 2.7 |
| Zimbabwe | 1.7 | 0.7 | 1.8 | 0.6 | 0.3 | 1.3 |

**Note:** AARC is calculated as ln((rate in latest year/rate in earliest year))/ (latest year - earliest year), with positive values denote increasing rate while negative values denote decreasing rate; N/A denotes not application and refers that the indicator has no sufficient data to make projections

Table S32: Predicted average annual rate of increase (AARC) of demand for family planning satisfied with modern contraceptive method among women 15-49 years of age in low- and middle-income countries by age

| **Country** | **Adolescent** | | | **Adult** | | |
| --- | --- | --- | --- | --- | --- | --- |
|  | **Predicted** | | **Required** | **Predicted** | | **Required** |
|  | **1990-2018** | **2019-2030** | **2019-2030** | **1990-2018** | **2019-2030** | **2019-2030** |
| Albania | -9.0 | -12.8 | 26.0 | -8.9 | -9.7 | 28.0 |
| Armenia | 2.5 | 2.3 | 9.4 | 2.7 | 2.0 | 7.2 |
| Bangladesh | 2.4 | 1.1 | 2.7 | 1.1 | 0.7 | 2.6 |
| Benin | 4.4 | 3.2 | 9.2 | 4.8 | 3.6 | 10.9 |
| Bolivia | 6.5 | 2.5 | 4.6 | 3.6 | 1.6 | 4.1 |
| Brazil | 0.8 | 0.2 | 3.6 | 1.3 | 0.1 | 0.5 |
| Burkina Faso | 6.5 | 2.0 | 3.5 | 8.0 | 2.6 | 4.0 |
| Burundi | 7.1 | 3.4 | 5.8 | 2.1 | 2.1 | 7.8 |
| Cambodia | 7.2 | 2.6 | 4.4 | 4.0 | 1.8 | 3.9 |
| Cameroon | 10.5 | 1.4 | 1.8 | 4.8 | 2.5 | 5.7 |
| Chad | 2.9 | 3.2 | 17.4 | 7.0 | 5.2 | 12.7 |
| Colombia | 2.3 | 0.9 | 2.1 | 1.0 | 0.4 | 1.0 |
| Comoros | 1.3 | 1.4 | 11.4 | 1.5 | 1.4 | 10.1 |
| Congo | 10.4 | 2.2 | 3.1 | 5.5 | 2.7 | 6.0 |
| Congo DR | -0.1 | 1.6 | 14.2 | 2.2 | 3.3 | 12.8 |
| Cote d'Ivoire | 7.0 | 2.9 | 5.5 | 5.0 | 2.8 | 7.1 |
| Dominican Republic | 3.3 | 1.2 | 2.5 | 0.6 | 0.3 | 1.1 |
| Egypt | 2.2 | 0.9 | 2.2 | 0.9 | 0.4 | 1.4 |
| Ethiopia | 11.4 | 2.4 | 3.1 | 9.2 | 2.1 | 2.9 |
| Gabon | 5.4 | 2.0 | 3.9 | 3.3 | 2.0 | 6.0 |
| Ghana | 1.7 | 1.5 | 9.7 | 3.2 | 2.1 | 6.8 |
| Guatemala | 3.8 | 2.1 | 5.5 | 1.9 | 1.0 | 3.1 |
| Guinea | 1.4 | 1.7 | 11.3 | 3.1 | 2.6 | 10.4 |
| Guyana | 36.6 | 0.0 | 0.0 | 17.0 | 0.3 | 0.3 |
| Haiti | 5.5 | 3.1 | 6.8 | 2.9 | 1.9 | 5.9 |
| Honduras | 2.7 | 1.0 | 3.2 | 1.9 | 0.4 | 1.8 |
| India | 4.5 | 3.4 | 10.6 | 0.6 | 0.4 | 2.5 |
| Indonesia | 0.8 | 0.4 | 1.3 | 0.2 | 0.2 | 2.2 |
| Jordan | 3.8 | 2.2 | 5.6 | 1.0 | 0.7 | 4.3 |
| Kazakhstan | -0.2 | 0.0 | 7.6 | 1.5 | 0.1 | 2.0 |
| Kenya | 6.6 | 2.0 | 3.2 | 3.0 | 1.0 | 2.1 |
| Kyrgyz Republic | -2.0 | -2.3 | 10.3 | -1.2 | -1.7 | 6.0 |
| Lesotho | 6.0 | 1.3 | 2.1 | 4.7 | 0.8 | 1.3 |
| Liberia | 37.9 | 0.0 | 0.0 | 32.4 | 0.1 | 0.1 |
| Madagascar | 13.0 | 1.9 | 2.3 | 7.5 | 1.6 | 2.2 |
| Malawi | 6.7 | 2.1 | 3.4 | 5.5 | 1.2 | 1.9 |
| Maldives | -9.7 | -12.6 | 28.1 | -4.8 | -8.0 | 14.8 |
| Mali | 4.4 | 3.3 | 10.4 | 3.3 | 2.6 | 9.7 |
| Morocco | 2.2 | 0.5 | 1.1 | 1.8 | 0.5 | 1.3 |
| Mozambique | 7.3 | 2.7 | 4.6 | 3.8 | 1.8 | 4.6 |
| Namibia | 1.4 | 0.6 | 1.9 | 1.4 | 0.6 | 1.4 |
| Nepal | 4.3 | 3.2 | 9.9 | 0.9 | 0.7 | 4.2 |
| Nicaragua | 3.9 | 0.2 | 1.3 | 1.2 | -0.1 | 1.6 |
| Niger | 8.1 | 4.4 | 8.5 | 5.6 | 2.8 | 6.2 |
| Nigeria | 4.4 | 2.6 | 6.5 | 3.8 | 2.3 | 6.5 |
| Pakistan | 5.3 | 4.0 | 11.2 | 3.0 | 1.9 | 5.8 |
| Peru | 4.0 | 1.7 | 3.5 | 1.4 | 0.9 | 3.4 |
| Philippines | 3.2 | 2.4 | 8.8 | 1.8 | 1.2 | 5.0 |
| Rwanda | 4.1 | 2.9 | 8.8 | 6.3 | 2.1 | 3.7 |
| Senegal | 5.9 | 4.3 | 11.0 | 4.8 | 2.7 | 6.0 |
| Sierra Leone | 17.4 | 1.2 | 1.4 | 12.8 | 2.0 | 2.5 |
| South Africa | -0.1 | -0.2 | 2.6 | -0.2 | -0.3 | 2.5 |
| Tajikistan | 4.0 | 6.5 | 14.1 | 0.3 | 0.6 | 7.1 |
| Tanzania | 5.6 | 2.5 | 5.0 | 3.5 | 1.7 | 4.2 |
| Timor-Leste | 0.5 | 2.4 | 13.2 | -0.6 | 0.0 | 8.7 |
| Togo | 4.4 | 2.5 | 6.4 | 5.9 | 3.2 | 7.0 |
| Turkey | 0.7 | 0.8 | 11.1 | 1.2 | 0.8 | 4.4 |
| Uganda | 3.8 | 2.3 | 6.5 | 4.3 | 2.2 | 4.9 |
| Vietnam | -6.1 | -3.4 | 19.1 | 0.0 | -0.3 | 4.1 |
| Yemen | 1.0 | 1.7 | 33.2 | 4.1 | 2.7 | 7.8 |
| Zambia | 5.4 | 2.3 | 4.6 | 4.5 | 1.5 | 2.8 |
| Zimbabwe | 1.2 | 0.7 | 3.2 | 1.3 | 0.5 | 1.3 |

**Note:** AARC is calculated as ln((rate in latest year/rate in earliest year))/ (latest year - earliest year), with positive values denote increasing rate while negative values denote decreasing rate

Table S33: Predicted average annual rate of increase (AARC) of antenatal care visit among women 15-49 years of age in low- and middle-income countries

| **Country** | **Predicted** | | **Required** |
| --- | --- | --- | --- |
|  | **1990-2018** | **2019-2030** | **2019-2030** |
| Afghanistan | -1.7 | -3.4 | 6.5 |
| Albania | -0.5 | -1.5 | 1.9 |
| Angola | -0.3 | -0.8 | 2.3 |
| Armenia | 2.8 | 0.0 | 0.0 |
| Bangladesh | 6.0 | 1.5 | 2.4 |
| Benin | -0.8 | -1.6 | 3.3 |
| Bolivia | 3.0 | 0.2 | 0.3 |
| Brazil | 1.7 | 0.0 | 0.0 |
| Burkina Faso | 0.9 | 0.5 | 3.5 |
| Burundi | -3.1 | -55.6 | 11.5 |
| Cambodia | 10.9 | 0.1 | 0.1 |
| Cameroon | 0.5 | 0.3 | 1.4 |
| Chad | 4.8 | 2.0 | 4.1 |
| Colombia | 0.6 | 0.1 | 0.2 |
| Comoros | 1.1 | 0.2 | 0.5 |
| Congo | 4.3 | 0.2 | 0.3 |
| Congo DR | 3.3 | 0.7 | 1.8 |
| Cote d'Ivoire | 1.4 | 0.3 | 0.8 |
| Dominican Republic | 0.0 | -0.1 | 0.4 |
| Egypt | 3.0 | 0.5 | 0.7 |
| Ethiopia | 12.7 | 3.0 | 3.9 |
| Gabon | 0.1 | 0.0 | 0.5 |
| Ghana | 0.5 | 0.1 | 0.3 |
| Guatemala | 1.6 | 0.2 | 0.2 |
| Guinea | 0.3 | 0.0 | 2.6 |
| Guyana | 5.0 | 0.1 | 0.1 |
| Haiti | 1.8 | 0.4 | 0.7 |
| Honduras | 3.2 | 0.1 | 0.1 |
| India | 1.9 | 0.7 | 1.6 |
| Indonesia | 0.3 | 0.2 | 1.3 |
| Jordan | 0.3 | 0.0 | 0.1 |
| Kazakhstan | 0.0 | -0.3 | 0.9 |
| Kenya | -0.4 | -1.0 | 1.7 |
| Kyrgyz Republic | -0.1 | -0.2 | 0.5 |
| Lesotho | 0.9 | 0.1 | 0.4 |
| Liberia | 4.5 | 0.1 | 0.1 |
| Madagascar | 2.2 | 0.5 | 0.9 |
| Malawi | 0.3 | 0.1 | 0.2 |
| Maldives | 14.3 | 0.1 | 0.1 |
| Mali | 2.9 | 1.4 | 3.6 |
| Morocco | 3.8 | 0.6 | 1.0 |
| Mozambique | 0.8 | 0.4 | 2.0 |
| Namibia | 0.4 | 0.1 | 0.2 |
| Nepal | 8.7 | 0.6 | 0.7 |
| Nicaragua | 0.5 | -0.3 | 1.9 |
| Niger | 4.8 | 0.8 | 1.2 |
| Nigeria | -0.1 | -0.1 | 4.8 |
| Pakistan | 4.5 | 0.9 | 1.3 |
| Peru | 2.2 | 0.1 | 0.1 |
| Philippines | 0.9 | 0.2 | 0.3 |
| Rwanda | 0.5 | 0.0 | 0.1 |
| Senegal | 2.3 | 0.2 | 0.3 |
| Sierra Leone | -0.2 | -1.3 | 2.4 |
| South Africa | -0.1 | -0.3 | 0.6 |
| Tajikistan | 6.9 | 0.7 | 1.0 |
| Tanzania | 0.0 | -0.1 | 1.7 |
| Timor-Leste | 0.2 | -0.5 | 1.6 |
| Togo | -0.4 | -0.6 | 4.8 |
| Turkey | 2.6 | 0.1 | 0.1 |
| Uganda | 0.5 | 0.1 | 0.3 |
| Vietnam | 6.8 | 0.1 | 0.1 |
| Yemen | 3.0 | 1.4 | 3.6 |
| Zambia | 0.2 | 0.1 | 0.3 |
| Zimbabwe | -0.4 | -1.1 | 1.7 |

**Note:** AARC is calculated as ln((rate in latest year/rate in earliest year))/ (latest year - earliest year), with positive values denote increasing rate while negative values denote decreasing rate

Table S34: Predicted average annual rate of increase (AARC) of antenatal care visit among women 15-49 years of age in low- and middle-income countries by wealth quintiles

| **Country** | **Poorest** | | | | **Poorer** | | | | **Middle** | | | | **Richer** | | | | **Richest** | | |
| --- | --- | --- | --- | --- | --- | --- | --- | --- | --- | --- | --- | --- | --- | --- | --- | --- | --- | --- | --- |
|  | **Predicted** | | **Required** | **Predicted** | | | **Required** | **Predicted** | | | **Required** | **Predicted** | | | **Required** | **Predicted** | | | **Required** |
|  | **1990-2018** | **2019-2030** | **2019-2030** | **1990-2018** | | **2019-2030** | **2019-2030** | **1990-2018** | | **2019-2030** | **2019-2030** | **1990-2018** | | **2019-2030** | **2019-2030** | **1990-2018** | | **2019-2030** | **2019-2030** |
| Afghanistan | 1.1 | 0.9 | 6.0 | -3.0 | | -6.4 | 9.2 | -2.6 | | -5.3 | 8.3 | -0.9 | | -1.9 | 5.1 | -1.0 | | -3.5 | 3.6 |
| Albania | -1.0 | -3.4 | 3.2 | -0.6 | | -1.7 | 2.5 | -0.3 | | -1.1 | 1.7 | -0.4 | | -1.3 | 1.8 | 0.0 | | -0.2 | 0.3 |
| Angola | 0.1 | -0.1 | 5.3 | -1.1 | | -2.8 | 3.8 | -0.5 | | -2.1 | 1.7 | -0.3 | | -2.1 | 0.9 | -0.2 | | -1.0 | 0.6 |
| Armenia | 3.9 | 0.1 | 0.1 | 11.2 | | 0.0 | 0.0 | 0.9 | | 0.0 | 0.1 | 4.3 | | 0.0 | 0.0 | 3.1 | | 0.0 | 0.0 |
| Bangladesh | 7.5 | 3.3 | 5.8 | 8.2 | | 2.3 | 3.4 | 7.2 | | 1.5 | 2.2 | 6.3 | | 0.8 | 1.1 | 3.1 | | 0.3 | 0.4 |
| Benin | -1.4 | -2.0 | 6.4 | -1.1 | | -1.9 | 4.3 | -1.1 | | -2.8 | 3.6 | -0.6 | | -2.1 | 1.9 | -0.4 | | -3.1 | 1.2 |
| Bolivia | 6.5 | 0.4 | 0.6 | 3.9 | | 0.2 | 0.3 | 3.1 | | 0.1 | 0.1 | 1.5 | | 0.1 | 0.1 | 0.3 | | 0.0 | 0.1 |
| Burkina Faso | 1.8 | 1.0 | 4.8 | 1.3 | | 0.8 | 4.5 | 1.6 | | 0.7 | 3.4 | 0.6 | | 0.3 | 3.0 | -0.3 | | -0.7 | 1.6 |
| Burundi | -3.3 | -54.9 | 12.3 | -3.0 | | -53.9 | 11.3 | -3.0 | | -57.6 | 11.6 | -3.3 | | -56.9 | 12.2 | -2.4 | | -50.5 | 9.0 |
| Cambodia | 12.7 | 0.2 | 0.3 | 12.7 | | 0.1 | 0.1 | 12.0 | | 0.1 | 0.1 | 11.9 | | 0.0 | 0.0 | 5.6 | | 0.0 | 0.0 |
| Cameroon | 0.6 | 0.4 | 4.3 | 1.0 | | 0.5 | 1.6 | 0.6 | | 0.2 | 0.6 | 0.3 | | 0.1 | 0.2 | 0.1 | | 0.0 | 0.1 |
| Chad | 11.1 | 3.4 | 5.0 | 8.3 | | 2.6 | 4.2 | 5.5 | | 2.3 | 4.7 | 3.0 | | 1.7 | 5.0 | 2.0 | | 0.7 | 1.7 |
| Colombia | 1.5 | 0.3 | 0.5 | 0.9 | | 0.1 | 0.1 | 0.5 | | 0.0 | 0.1 | 0.2 | | 0.0 | 0.0 | 0.3 | | 0.0 | 0.0 |
| Comoros | 3.0 | 0.4 | 0.6 | 1.1 | | 0.3 | 0.7 | 1.0 | | 0.1 | 0.3 | 0.3 | | 0.1 | 0.3 | -0.1 | | -0.2 | 0.6 |
| Congo | 3.1 | 0.5 | 1.1 | 3.7 | | 0.3 | 0.5 | 6.9 | | 0.1 | 0.1 | 7.2 | | 0.1 | 0.1 | 10.9 | | 0.0 | 0.0 |
| Congo DR | 2.8 | 1.0 | 2.9 | 3.8 | | 0.9 | 2.0 | 2.6 | | 0.7 | 2.0 | 4.9 | | 0.7 | 1.1 | 3.7 | | 0.3 | 0.4 |
| Cote d'Ivoire | 3.1 | 0.7 | 1.5 | 1.2 | | 0.4 | 1.4 | 1.8 | | 0.2 | 0.4 | 0.6 | | 0.1 | 0.2 | 0.2 | | 0.0 | 0.1 |
| Dominican Republic | -1.0 | -5.9 | 2.9 | -0.2 | | -1.8 | 0.8 | 0.1 | | 0.0 | 0.2 | -0.3 | | -2.3 | 0.9 | -0.4 | | -3.2 | 1.3 |
| Egypt | 8.2 | 0.7 | 0.8 | 6.7 | | 0.5 | 0.6 | 4.6 | | 0.3 | 0.4 | 3.0 | | 0.2 | 0.3 | 1.0 | | 0.1 | 0.2 |
| Ethiopia | 16.5 | 5.1 | 6.4 | 16.6 | | 3.5 | 4.2 | 15.7 | | 3.1 | 3.7 | 15.5 | | 2.6 | 3.0 | 8.7 | | 0.8 | 1.0 |
| Gabon | 0.5 | 0.2 | 1.1 | 0.1 | | 0.0 | 0.5 | -0.1 | | -0.5 | 0.4 | 0.0 | | 0.0 | 0.1 | -0.1 | | -0.6 | 0.5 |
| Ghana | 0.8 | 0.3 | 0.8 | 0.7 | | 0.2 | 0.3 | 0.5 | | 0.1 | 0.2 | 0.2 | | 0.0 | 0.1 | 0.1 | | 0.0 | 0.1 |
| Guatemala | 2.6 | 0.2 | 0.3 | 2.1 | | 0.2 | 0.3 | 1.4 | | 0.2 | 0.2 | 1.0 | | 0.1 | 0.1 | 0.2 | | 0.0 | 0.1 |
| Guinea | -0.3 | -0.4 | 5.7 | 1.2 | | 0.6 | 3.0 | 0.3 | | 0.0 | 2.5 | 0.0 | | -0.2 | 1.2 | -0.1 | | -0.3 | 0.5 |
| Guyana | 9.3 | 0.1 | 0.2 | 1.2 | | -0.1 | 0.3 | 3.4 | | 0.0 | 0.1 | 0.0 | | -0.6 | 0.5 | 11.1 | | 0.0 | 0.0 |
| Haiti | 3.6 | 0.8 | 1.3 | 2.8 | | 0.4 | 0.7 | 1.7 | | 0.3 | 0.6 | 0.7 | | 0.2 | 0.5 | 0.5 | | 0.1 | 0.1 |
| Honduras | 4.4 | 0.1 | 0.2 | 3.1 | | 0.1 | 0.2 | 4.5 | | 0.1 | 0.1 | 1.6 | | 0.0 | 0.1 | 0.5 | | 0.0 | 0.0 |
| India | 3.1 | 1.5 | 3.7 | 3.2 | | 0.9 | 1.7 | 2.3 | | 0.5 | 0.9 | 1.2 | | 0.3 | 0.6 | 0.2 | | 0.1 | 0.4 |
| Indonesia | -0.5 | -0.8 | 3.6 | -0.5 | | -1.0 | 2.1 | -0.4 | | -0.9 | 1.4 | -0.3 | | -1.3 | 1.1 | -0.7 | | -7.1 | 2.3 |
| Jordan | 0.5 | 0.1 | 0.1 | 0.3 | | 0.0 | 0.0 | 0.2 | | 0.0 | 0.0 | 0.2 | | 0.0 | 0.0 | 0.1 | | 0.0 | 0.0 |
| Kazakhstan | 0.8 | 0.0 | 0.1 | -0.4 | | -0.7 | 1.4 | 0.9 | | 0.0 | 0.2 | -2.0 | | -3.1 | 5.9 | -11.1 | | -12.2 | 29.7 |
| Kenya | -0.7 | -1.4 | 2.9 | -0.6 | | -1.6 | 2.0 | -0.5 | | -1.6 | 1.8 | -0.2 | | -0.5 | 1.0 | -0.2 | | -0.5 | 0.7 |
| Kyrgyz Republic | -0.2 | -0.4 | 0.7 | -0.1 | | -0.3 | 0.5 | -0.2 | | -0.6 | 0.7 | 0.0 | | -0.1 | 0.4 | 0.0 | | 0.0 | 0.1 |
| Lesotho | 1.0 | 0.2 | 0.6 | 0.7 | | 0.2 | 0.7 | 0.5 | | 0.1 | 0.5 | 3.4 | | 0.1 | 0.1 | 0.3 | | 0.0 | 0.2 |
| Liberia | 3.9 | 0.2 | 0.3 | 14.0 | | 0.0 | 0.0 | 4.9 | | 0.1 | 0.1 | 0.6 | | 0.0 | 0.1 | 3.6 | | 0.0 | 0.0 |
| Madagascar | 2.6 | 0.9 | 2.0 | 2.7 | | 0.6 | 1.1 | 2.5 | | 0.5 | 0.8 | 2.0 | | 0.2 | 0.4 | 1.1 | | 0.1 | 0.2 |
| Malawi | 0.5 | 0.1 | 0.3 | 0.5 | | 0.1 | 0.2 | 0.3 | | 0.1 | 0.2 | 0.0 | | 0.0 | 0.3 | 0.1 | | 0.0 | 0.1 |
| Maldives | 8.6 | 0.1 | 0.1 | 12.6 | | 0.1 | 0.1 | 18.0 | | 0.0 | 0.0 | 9.9 | | 0.1 | 0.2 | 59.6 | | 0.0 | 0.0 |
| Mali | 6.6 | 2.7 | 4.9 | 3.7 | | 2.1 | 5.6 | 3.5 | | 1.8 | 4.7 | 2.7 | | 1.0 | 2.4 | 1.0 | | 0.2 | 0.5 |
| Morocco | 8.9 | 1.0 | 1.4 | 6.1 | | 0.6 | 0.9 | 4.0 | | 0.4 | 0.6 | 2.3 | | 0.1 | 0.2 | 1.2 | | 0.1 | 0.1 |
| Mozambique | 2.4 | 1.1 | 2.7 | 0.4 | | 0.2 | 3.0 | 1.5 | | 0.6 | 1.7 | -0.2 | | -0.4 | 1.6 | -0.4 | | -1.7 | 1.2 |
| Namibia | 0.7 | 0.2 | 0.4 | 0.4 | | 0.1 | 0.3 | 0.5 | | 0.1 | 0.1 | 0.5 | | 0.1 | 0.1 | 0.2 | | 0.1 | 0.1 |
| Nepal | 12.3 | 1.2 | 1.4 | 11.0 | | 0.7 | 0.8 | 10.0 | | 0.5 | 0.5 | 8.6 | | 0.2 | 0.3 | 3.6 | | 0.1 | 0.1 |
| Nicaragua | -0.8 | -0.6 | 5.5 | 0.7 | | -0.2 | 1.5 | 1.5 | | -0.1 | 0.6 | 0.6 | | -0.2 | 0.9 | 0.1 | | -0.4 | 1.1 |
| Niger | 8.4 | 1.0 | 1.4 | 8.7 | | 0.7 | 0.9 | 9.0 | | 0.6 | 0.7 | 5.6 | | 0.7 | 1.0 | 1.6 | | 0.2 | 0.3 |
| Nigeria | -1.3 | -1.1 | 12.9 | -0.2 | | -0.1 | 7.7 | 0.0 | | -0.1 | 4.1 | 0.3 | | 0.2 | 1.6 | -0.1 | | -0.2 | 0.7 |
| Pakistan | 7.8 | 2.1 | 3.2 | 7.7 | | 1.3 | 1.7 | 7.2 | | 0.6 | 0.7 | 4.4 | | 0.3 | 0.4 | 1.3 | | 0.1 | 0.1 |
| Peru | 4.7 | 0.2 | 0.3 | 2.7 | | 0.1 | 0.2 | 1.4 | | 0.1 | 0.1 | 0.6 | | 0.1 | 0.1 | 0.3 | | 0.0 | 0.0 |
| Philippines | 1.7 | 0.4 | 0.9 | 1.4 | | 0.1 | 0.2 | 0.7 | | 0.1 | 0.2 | 0.6 | | 0.0 | 0.0 | 0.2 | | 0.0 | 0.1 |
| Rwanda | 0.5 | 0.1 | 0.1 | 0.6 | | 0.0 | 0.1 | 0.4 | | 0.0 | 0.0 | 0.6 | | 0.0 | 0.0 | 0.7 | | 0.0 | 0.0 |
| Senegal | 6.2 | 0.5 | 0.7 | 5.9 | | 0.3 | 0.3 | 4.3 | | 0.1 | 0.1 | 2.6 | | 0.0 | 0.0 | 3.0 | | 0.0 | 0.0 |
| Sierra Leone | 0.3 | -0.7 | 3.0 | -0.4 | | -1.6 | 3.2 | -1.0 | | -3.5 | 3.6 | 0.1 | | -0.9 | 1.7 | 0.0 | | -0.9 | 0.7 |
| South Africa | -0.1 | -0.3 | 0.6 | -0.2 | | -0.4 | 0.8 | -0.2 | | -0.5 | 0.5 | 0.0 | | -0.1 | 0.4 | -0.1 | | -0.2 | 0.8 |
| Tajikistan | 11.0 | 1.3 | 1.6 | 8.7 | | 1.0 | 1.3 | 5.8 | | 0.6 | 0.9 | 2.6 | | 0.3 | 0.7 | 6.2 | | 0.3 | 0.4 |
| Tanzania | 0.2 | 0.1 | 2.3 | -0.2 | | -0.4 | 2.2 | 0.1 | | 0.0 | 1.8 | 0.3 | | 0.1 | 1.1 | 0.0 | | 0.0 | 0.6 |
| Timor-Leste | 0.7 | -0.1 | 2.6 | 0.3 | | -0.4 | 2.3 | -0.1 | | -0.8 | 1.6 | 0.3 | | -0.2 | 0.7 | -0.1 | | -0.8 | 0.6 |
| Togo | -2.1 | -2.4 | 11.1 | -1.6 | | -1.9 | 8.6 | -1.0 | | -1.3 | 5.9 | 0.2 | | 0.1 | 1.7 | 0.1 | | 0.0 | 0.5 |
| Turkey | 5.6 | 0.3 | 0.4 | 3.6 | | 0.1 | 0.1 | 2.5 | | 0.0 | 0.0 | 1.2 | | 0.0 | 0.0 | 0.4 | | 0.0 | 0.0 |
| Uganda | 0.7 | 0.2 | 0.3 | 0.7 | | 0.1 | 0.3 | 0.5 | | 0.1 | 0.2 | 0.3 | | 0.1 | 0.3 | 0.1 | | 0.0 | 0.2 |
| Vietnam | 7.8 | 0.3 | 0.5 | 6.8 | | 0.2 | 0.2 | 8.7 | | 0.0 | 0.0 | 6.5 | | 0.0 | 0.1 | 10.0 | | 0.0 | 0.0 |
| Zambia | 0.3 | 0.1 | 0.6 | 0.2 | | 0.1 | 0.5 | 0.3 | | 0.1 | 0.3 | 0.0 | | 0.0 | 0.2 | 0.0 | | -0.1 | 0.1 |
| Zimbabwe | -0.7 | -1.8 | 2.4 | -0.5 | | -1.3 | 1.9 | -0.4 | | -0.9 | 1.7 | -0.4 | | -1.0 | 1.6 | -0.1 | | -0.2 | 0.5 |

**Note:** AARC is calculated as ln((rate in latest year/rate in earliest year))/ (latest year - earliest year), with positive values denote increasing rate while negative values denote decreasing rate

Table S35: Predicted average annual rate of increase (AARC) of antenatal care visit among women 15-49 years of age in low- and middle-income countries by place of residence

| **Country** | **Rural** | | | **Urban** | | |
| --- | --- | --- | --- | --- | --- | --- |
|  | **Predicted** | | **Required** | **Predicted** | | **Required** |
|  | **1990-2018** | **2019-2030** | **2019-2030** | **1990-2018** | **2019-2030** | **2019-2030** |
| Afghanistan | -1.3 | -1.9 | 6.7 | -2.4 | -18.3 | 7.6 |
| Albania | -0.5 | -1.4 | 2.3 | -0.5 | -2.9 | 1.7 |
| Angola | -0.9 | -1.7 | 4.7 | -0.5 | -5.0 | 1.7 |
| Armenia | 3.7 | 0.0 | 0.0 | 2.3 | 0.0 | 0.0 |
| Bangladesh | 6.4 | 1.9 | 3.0 | 2.5 | 0.7 | 1.3 |
| Benin | -0.9 | -1.7 | 3.9 | -0.7 | -1.7 | 2.5 |
| Bolivia | 4.9 | 0.4 | 0.5 | 1.9 | 0.1 | 0.1 |
| Brazil | 2.6 | 0.1 | 0.2 | 0.9 | 0.0 | 0.0 |
| Burkina Faso | 1.2 | 0.6 | 3.9 | -0.8 | -2.6 | 2.6 |
| Burundi | -3.2 | -56.4 | 11.8 | -2.3 | -49.6 | 8.7 |
| Cambodia | 11.2 | 0.1 | 0.1 | 9.6 | 0.0 | 0.0 |
| Cameroon | 0.4 | 0.3 | 2.5 | 0.2 | 0.1 | 0.4 |
| Chad | 6.5 | 2.5 | 4.6 | 2.2 | 0.7 | 1.8 |
| Colombia | 1.1 | 0.2 | 0.4 | 0.5 | 0.1 | 0.1 |
| Comoros | 1.3 | 0.3 | 0.5 | 0.6 | 0.2 | 0.4 |
| Congo | 2.9 | 0.4 | 0.8 | 5.7 | 0.1 | 0.1 |
| Congo DR | 3.8 | 0.9 | 2.0 | 4.0 | 0.5 | 0.9 |
| Cote d'Ivoire | 1.8 | 0.5 | 1.3 | 0.5 | 0.1 | 0.2 |
| Dominican Republic | 0.0 | -0.1 | 0.3 | -0.1 | -0.2 | 0.4 |
| Egypt | 4.2 | 0.6 | 0.8 | 1.6 | 0.3 | 0.5 |
| Ethiopia | 14.8 | 3.5 | 4.3 | 7.6 | 0.5 | 0.7 |
| Gabon | 0.2 | 0.0 | 1.4 | -0.1 | -0.3 | 0.5 |
| Ghana | 0.6 | 0.2 | 0.5 | 0.1 | 0.0 | 0.2 |
| Guatemala | 1.9 | 0.2 | 0.3 | 1.0 | 0.1 | 0.2 |
| Guinea | 0.3 | 0.1 | 3.5 | 0.0 | -0.1 | 0.4 |
| Guyana | 4.6 | 0.1 | 0.2 | 12.7 | 0.0 | 0.0 |
| Haiti | 2.6 | 0.5 | 0.8 | 0.5 | 0.2 | 0.6 |
| Honduras | 4.1 | 0.1 | 0.1 | 1.8 | 0.1 | 0.1 |
| India | 2.3 | 0.9 | 1.9 | 0.5 | 0.2 | 0.8 |
| Indonesia | 0.4 | 0.2 | 1.7 | -0.1 | -0.3 | 0.9 |
| Jordan | 0.6 | 0.0 | 0.1 | 0.2 | 0.0 | 0.1 |
| Kazakhstan | 0.3 | -0.1 | 0.3 | -0.5 | -0.7 | 2.1 |
| Kenya | -0.5 | -1.3 | 2.0 | -0.3 | -0.9 | 1.0 |
| Kyrgyz Republic | -0.1 | -0.4 | 0.6 | 0.0 | 0.0 | 0.1 |
| Lesotho | 1.0 | 0.2 | 0.4 | 0.1 | 0.0 | 0.3 |
| Liberia | 5.8 | 0.2 | 0.2 | 0.0 | -0.2 | 0.2 |
| Madagascar | 2.3 | 0.6 | 1.0 | 2.2 | 0.2 | 0.3 |
| Malawi | 0.3 | 0.1 | 0.2 | 0.0 | 0.0 | 0.1 |
| Maldives | 12.4 | 0.1 | 0.1 | 18.9 | 0.0 | 0.0 |
| Mali | 4.5 | 2.1 | 4.4 | 1.4 | 0.3 | 0.7 |
| Morocco | 5.4 | 1.1 | 1.9 | 1.7 | 0.2 | 0.5 |
| Mozambique | 1.1 | 0.6 | 2.4 | -0.4 | -1.1 | 1.6 |
| Namibia | 0.5 | 0.1 | 0.3 | 0.2 | 0.1 | 0.2 |
| Nepal | 9.1 | 0.7 | 0.8 | 2.2 | 0.3 | 0.4 |
| Nicaragua | 0.6 | -0.2 | 2.5 | 0.9 | -0.2 | 0.9 |
| Niger | 6.4 | 0.9 | 1.2 | 0.6 | 0.1 | 0.3 |
| Nigeria | -0.5 | -0.5 | 6.9 | -0.2 | -0.3 | 1.8 |
| Pakistan | 6.8 | 1.1 | 1.6 | 2.0 | 0.3 | 0.5 |
| Peru | 4.0 | 0.2 | 0.2 | 1.1 | 0.1 | 0.1 |
| Philippines | 1.4 | 0.2 | 0.3 | 0.5 | 0.1 | 0.3 |
| Rwanda | 0.5 | 0.0 | 0.1 | 0.5 | 0.0 | 0.0 |
| Senegal | 3.2 | 0.3 | 0.5 | 0.5 | 0.1 | 0.1 |
| Sierra Leone | -0.5 | -1.7 | 3.2 | -0.1 | -1.5 | 1.3 |
| South Africa | 0.0 | 0.0 | 0.4 | -0.2 | -0.6 | 0.8 |
| Tajikistan | 6.8 | 0.8 | 1.2 | 10.9 | 0.3 | 0.4 |
| Tanzania | 0.0 | -0.1 | 1.9 | -0.1 | -0.2 | 0.8 |
| Timor-Leste | 0.1 | -0.6 | 1.9 | 0.3 | -0.2 | 0.6 |
| Togo | -1.5 | -1.8 | 8.1 | -0.2 | -0.3 | 1.3 |
| Turkey | 3.6 | 0.3 | 0.4 | 1.8 | 0.1 | 0.1 |
| Uganda | 0.5 | 0.1 | 0.3 | 0.2 | 0.1 | 0.2 |
| Vietnam | 7.4 | 0.1 | 0.2 | 4.3 | 0.0 | 0.0 |
| Yemen | 3.6 | 1.8 | 4.6 | 1.1 | 0.5 | 1.4 |
| Zambia | 0.4 | 0.1 | 0.4 | 0.0 | 0.0 | 0.1 |
| Zimbabwe | -0.5 | -1.2 | 1.9 | -0.3 | -0.9 | 1.2 |

**Note:** AARC is calculated as ln((rate in latest year/rate in earliest year))/ (latest year - earliest year), with positive values denote increasing rate while negative values denote decreasing rate

Table S36: Predicted average annual rate of increase (AARC) of antenatal care visit among women 15-49 years of age in low- and middle-income countries by education

| **Country** | **Below secondary education** | | | **Secondary+ education** | | |
| --- | --- | --- | --- | --- | --- | --- |
|  | **Predicted** | | **Required** | **Predicted** | | **Required** |
|  | **1990-2018** | **2019-2030** | **2019-2030** | **1990-2018** | **2019-2030** | **2019-2030** |
| Afghanistan | -3.4 | -13.5 | 10.0 | -1.0 | -5.5 | 3.1 |
| Albania | -0.8 | -2.8 | 2.7 | -0.3 | -1.1 | 1.3 |
| Angola | -0.9 | -2.3 | 3.6 | -0.9 | -58.8 | 4.1 |
| Armenia | 0.0 | 0.0 | 0.0 | 2.9 | 0.0 | 0.0 |
| Bangladesh | 5.9 | 2.6 | 4.8 | 2.2 | 0.8 | 1.7 |
| Benin | -1.0 | -1.9 | 3.8 | -0.5 | -6.0 | 1.6 |
| Bolivia | 4.6 | 0.3 | 0.4 | 1.1 | 0.1 | 0.1 |
| Brazil | 1.7 | 0.1 | 0.3 | -1.6 | -3.1 | 4.6 |
| Burkina Faso | 0.8 | 0.4 | 3.8 | 0.0 | -0.2 | 0.4 |
| Burundi | -3.2 | -56.0 | 11.8 | -2.0 | -38.0 | 7.2 |
| Cambodia | 11.1 | 0.1 | 0.2 | 6.2 | 0.0 | 0.0 |
| Cameroon | 0.4 | 0.2 | 2.3 | 0.1 | 0.0 | 0.2 |
| Chad | 4.7 | 2.1 | 4.7 | 0.6 | 0.2 | 1.6 |
| Colombia | 0.8 | 0.3 | 0.7 | 0.3 | 0.0 | 0.1 |
| Comoros | 1.1 | 0.3 | 0.6 | 0.3 | 0.1 | 0.3 |
| Congo | 2.9 | 0.4 | 1.0 | 6.8 | 0.1 | 0.1 |
| Congo DR | 3.9 | 1.0 | 2.1 | 2.1 | 0.4 | 1.2 |
| Cote d'Ivoire | 1.6 | 0.3 | 0.9 | 0.2 | 0.0 | 0.3 |
| Dominican Republic | -0.1 | -0.4 | 0.5 | 0.0 | -0.1 | 0.3 |
| Egypt | 3.7 | 0.9 | 1.6 | 1.2 | 0.3 | 0.6 |
| Ethiopia | 13.6 | 3.3 | 4.2 | 7.4 | 0.3 | 0.4 |
| Gabon | 0.1 | -0.1 | 0.9 | -0.1 | -0.3 | 0.4 |
| Ghana | 0.5 | 0.2 | 0.6 | 0.0 | 0.0 | 0.2 |
| Guatemala | 1.7 | 0.2 | 0.3 | 0.2 | 0.1 | 0.1 |
| Guinea | 0.2 | 0.0 | 2.8 | -0.1 | -0.5 | 0.6 |
| Guyana | 2.3 | 0.1 | 0.7 | 6.7 | 0.0 | 0.1 |
| Haiti | 1.8 | 0.5 | 1.1 | 0.3 | 0.1 | 0.3 |
| Honduras | 2.9 | 0.1 | 0.2 | 1.1 | 0.0 | 0.1 |
| India | 2.0 | 1.0 | 3.0 | 0.1 | 0.0 | 0.9 |
| Indonesia | 0.4 | 0.2 | 1.9 | -0.3 | -0.6 | 1.2 |
| Jordan | 0.6 | 0.1 | 0.2 | 0.1 | 0.0 | 0.1 |
| Kazakhstan | N/A | N/A | N/A | 0.0 | -0.4 | 1.0 |
| Kenya | -0.5 | -1.2 | 2.0 | -0.4 | -1.7 | 1.1 |
| Kyrgyz Republic | 0.0 | 0.0 | 0.0 | -0.1 | -0.2 | 0.5 |
| Lesotho | 0.7 | 0.2 | 0.7 | 0.6 | 0.1 | 0.2 |
| Liberia | 4.6 | 0.1 | 0.2 | 0.7 | 0.0 | 0.1 |
| Madagascar | 2.4 | 0.6 | 1.2 | 1.5 | 0.1 | 0.2 |
| Malawi | 0.3 | 0.1 | 0.3 | 0.0 | 0.0 | 0.1 |
| Maldives | 21.0 | 0.0 | 0.0 | 13.9 | 0.1 | 0.1 |
| Mali | 3.0 | 1.5 | 3.9 | -0.2 | -0.4 | 1.3 |
| Morocco | 4.3 | 0.7 | 1.2 | 0.3 | 0.1 | 0.5 |
| Mozambique | 0.6 | 0.4 | 2.3 | -0.5 | -2.5 | 1.5 |
| Namibia | 0.4 | 0.2 | 0.6 | 0.2 | 0.1 | 0.1 |
| Nepal | 9.2 | 0.9 | 1.1 | 2.5 | 0.2 | 0.3 |
| Nicaragua | 0.3 | -0.3 | 2.6 | 0.6 | -0.2 | 0.7 |
| Niger | 5.0 | 0.8 | 1.2 | 0.6 | 0.0 | 0.1 |
| Nigeria | -0.9 | -1.0 | 7.8 | -0.4 | -0.7 | 1.6 |
| Pakistan | 5.2 | 1.2 | 1.9 | 1.1 | 0.2 | 0.3 |
| Peru | 3.7 | 0.2 | 0.3 | 0.9 | 0.1 | 0.1 |
| Philippines | 1.3 | 0.4 | 1.1 | 0.5 | 0.1 | 0.2 |
| Rwanda | 0.5 | 0.0 | 0.1 | 0.1 | 0.0 | 0.1 |
| Senegal | 2.3 | 0.3 | 0.4 | 0.1 | 0.0 | 0.1 |
| Sierra Leone | -0.2 | -1.3 | 2.7 | -0.8 | -5.0 | 2.4 |
| South Africa | -0.3 | -0.8 | 1.1 | -0.1 | -0.3 | 0.6 |
| Tajikistan | 4.4 | 1.2 | 2.4 | 7.6 | 0.7 | 0.9 |
| Tanzania | -0.1 | -0.1 | 1.8 | -0.2 | -0.5 | 1.0 |
| Timor-Leste | -0.1 | -1.0 | 2.5 | -0.3 | -1.4 | 1.1 |
| Togo | -1.0 | -1.3 | 6.3 | -0.6 | -1.5 | 2.4 |
| Turkey | 3.0 | 0.1 | 0.2 | 0.3 | 0.0 | 0.1 |
| Uganda | 0.5 | 0.1 | 0.3 | 0.0 | 0.0 | 0.2 |
| Vietnam | 7.0 | 0.3 | 0.6 | 6.9 | 0.0 | 0.1 |
| Yemen | 3.0 | 1.5 | 4.1 | -0.3 | -0.5 | 1.8 |
| Zambia | 0.2 | 0.1 | 0.5 | 0.0 | -0.1 | 0.2 |
| Zimbabwe | -0.7 | -1.8 | 2.6 | -0.4 | -1.4 | 1.5 |

**Note:** AARC is calculated as ln((rate in latest year/rate in earliest year))/ (latest year - earliest year), with positive values denote increasing rate while negative values denote decreasing rate; N/A denotes not application and refers that the indicator has no sufficient data to make projections

Table S37: Predicted average annual rate of increase (AARC) of antenatal care visit among women 15-49 years of age in low- and middle-income countries by age

| Country | **Adolescent** | | | **Adult** | | |
| --- | --- | --- | --- | --- | --- | --- |
|  | **Predicted** | | **Required** | **Predicted** | | **Required** |
|  | **1990-2018** | **2019-2030** | **2019-2030** | **1990-2018** | **2019-2030** | **2019-2030** |
| Afghanistan | -1.5 | -2.7 | 6.6 | -1.8 | -3.9 | 6.6 |
| Albania | -1.6 | -15.3 | 4.9 | -0.4 | -1.3 | 1.9 |
| Angola | -0.8 | -2.8 | 2.7 | -0.2 | -0.7 | 2.3 |
| Armenia | 9.2 | 0.0 | 0.0 | 2.8 | 0.0 | 0.0 |
| Bangladesh | 6.2 | 1.5 | 2.2 | 5.9 | 1.6 | 2.4 |
| Benin | -1.1 | -2.3 | 4.1 | -0.8 | -1.6 | 3.3 |
| Bolivia | 3.1 | 0.2 | 0.3 | 2.9 | 0.2 | 0.3 |
| Brazil | 1.7 | 0.0 | 0.0 | 1.7 | 0.0 | 0.0 |
| Burkina Faso | 0.4 | 0.2 | 4.1 | 0.8 | 0.4 | 3.6 |
| Burundi | -2.1 | -30.5 | 7.4 | -3.1 | -56.8 | 11.7 |
| Cambodia | 9.1 | 0.1 | 0.1 | 10.9 | 0.1 | 0.1 |
| Cameroon | 0.5 | 0.3 | 1.3 | 0.4 | 0.3 | 1.4 |
| Chad | 4.5 | 1.8 | 3.9 | 5.0 | 2.0 | 4.1 |
| Colombia | 0.6 | 0.1 | 0.2 | 0.7 | 0.1 | 0.2 |
| Comoros | 1.0 | 0.2 | 0.4 | 1.1 | 0.2 | 0.5 |
| Congo | 2.7 | 0.2 | 0.5 | 4.8 | 0.2 | 0.3 |
| Congo DR | 5.3 | 0.8 | 1.3 | 3.3 | 0.7 | 1.7 |
| Cote d'Ivoire | 1.8 | 0.3 | 0.6 | 1.4 | 0.3 | 0.8 |
| Dominican Republic | -0.1 | -0.3 | 0.4 | 0.0 | -0.1 | 0.3 |
| Egypt | 3.3 | 0.4 | 0.6 | 3.0 | 0.5 | 0.7 |
| Ethiopia | 14.0 | 2.5 | 3.0 | 12.5 | 3.1 | 3.9 |
| Gabon | -0.2 | -0.7 | 0.9 | 0.2 | 0.0 | 0.4 |
| Ghana | 0.2 | 0.1 | 0.4 | 0.5 | 0.1 | 0.3 |
| Guatemala | 1.4 | 0.2 | 0.3 | 1.6 | 0.2 | 0.2 |
| Guinea | 0.3 | 0.0 | 2.2 | 0.3 | 0.0 | 2.6 |
| Guyana | -1.0 | -4.6 | 3.2 | 5.8 | 0.1 | 0.1 |
| Haiti | 1.5 | 0.4 | 0.8 | 1.9 | 0.4 | 0.7 |
| Honduras | 4.9 | 0.1 | 0.1 | 3.0 | 0.1 | 0.1 |
| India | 1.8 | 0.7 | 1.5 | 1.9 | 0.7 | 1.6 |
| Indonesia | 0.5 | 0.3 | 1.4 | 0.3 | 0.1 | 1.3 |
| Jordan | 0.1 | 0.0 | 0.0 | 0.3 | 0.0 | 0.1 |
| Kazakhstan | 16.9 | 0.0 | 0.0 | 0.0 | -0.3 | 0.9 |
| Kenya | -0.6 | -1.2 | 2.4 | -0.4 | -1.0 | 1.6 |
| Kyrgyz Republic | 0.0 | 0.0 | 0.2 | -0.1 | -0.2 | 0.5 |
| Lesotho | 1.3 | 0.1 | 0.3 | 0.9 | 0.1 | 0.4 |
| Liberia | 3.9 | 0.1 | 0.1 | 4.5 | 0.1 | 0.1 |
| Madagascar | 2.8 | 0.6 | 1.1 | 2.2 | 0.5 | 0.9 |
| Malawi | 0.2 | 0.1 | 0.2 | 0.3 | 0.1 | 0.2 |
| Maldives | 40.0 | 0.0 | 0.0 | 14.8 | 0.1 | 0.1 |
| Mali | 2.5 | 1.3 | 3.5 | 2.9 | 1.4 | 3.6 |
| Morocco | 4.0 | 0.7 | 1.3 | 3.8 | 0.6 | 0.9 |
| Mozambique | 0.8 | 0.4 | 1.8 | 0.8 | 0.4 | 2.0 |
| Namibia | 0.3 | 0.1 | 0.3 | 0.4 | 0.1 | 0.2 |
| Nepal | 7.7 | 0.4 | 0.5 | 8.8 | 0.6 | 0.7 |
| Nicaragua | 1.6 | -0.1 | 0.9 | 0.3 | -0.4 | 2.2 |
| Niger | 4.5 | 0.9 | 1.5 | 4.8 | 0.8 | 1.2 |
| Nigeria | -0.3 | -0.3 | 7.3 | -0.1 | -0.1 | 4.6 |
| Pakistan | 4.7 | 0.9 | 1.3 | 4.5 | 0.9 | 1.3 |
| Peru | 2.4 | 0.2 | 0.3 | 2.2 | 0.1 | 0.1 |
| Philippines | 1.4 | 0.2 | 0.3 | 0.9 | 0.2 | 0.3 |
| Rwanda | 3.4 | 0.0 | 0.0 | 0.5 | 0.0 | 0.1 |
| Senegal | 2.4 | 0.3 | 0.4 | 2.2 | 0.2 | 0.3 |
| Sierra Leone | -0.7 | -2.6 | 3.0 | -0.1 | -1.1 | 2.3 |
| South Africa | -0.2 | -0.6 | 0.7 | -0.1 | -0.3 | 0.6 |
| Tajikistan | -0.3 | -1.6 | 2.4 | 7.3 | 0.7 | 1.0 |
| Tanzania | 0.1 | 0.0 | 1.5 | 0.0 | 0.0 | 1.7 |
| Timor-Leste | -0.3 | -1.2 | 2.3 | 0.3 | -0.4 | 1.5 |
| Togo | -1.3 | -1.8 | 6.3 | -0.4 | -0.6 | 4.8 |
| Turkey | 2.0 | 0.1 | 0.1 | 2.7 | 0.1 | 0.1 |
| Uganda | 0.2 | 0.1 | 0.4 | 0.5 | 0.1 | 0.3 |
| Vietnam | 2.4 | 0.7 | 4.0 | 6.9 | 0.1 | 0.1 |
| Yemen | 3.1 | 1.4 | 3.3 | 3.0 | 1.4 | 3.6 |
| Zambia | 0.3 | 0.1 | 0.2 | 0.2 | 0.1 | 0.4 |
| Zimbabwe | -0.4 | -1.0 | 1.6 | -0.4 | -1.1 | 1.7 |

**Note:** AARC is calculated as ln((rate in latest year/rate in earliest year))/ (latest year - earliest year), with positive values denote increasing rate while negative values denote decreasing rate

Table S38: Predicted average annual rate of increase (AARC) of skilled birth attendance among women 15-49 years of age in low- and middle-income countries

| **Country** | **Predicted** | | **Required** |
| --- | --- | --- | --- |
|  | **1990-2018** | **2019-2030** | **2019-2030** |
| Afghanistan | 3.4 | 2.2 | 5.9 |
| Albania | -0.6 | -1.4 | 3.0 |
| Angola | -4.6 | -8.6 | 13.6 |
| Armenia | 2.4 | 0.0 | 0.0 |
| Bangladesh | 8.1 | 3.7 | 6.3 |
| Benin | 2.1 | 0.6 | 1.1 |
| Bolivia | 2.5 | 0.7 | 1.6 |
| Brazil | 1.6 | 0.0 | 0.1 |
| Burkina Faso | -0.2 | 0.2 | 11.5 |
| Burundi | 10.1 | 0.8 | 1.0 |
| Cambodia | 10.7 | 0.4 | 0.4 |
| Cameroon | 0.2 | 0.1 | 4.8 |
| Chad | 4.9 | 4.6 | 14.9 |
| Colombia | 0.7 | 0.2 | 0.3 |
| Comoros | 4.4 | 0.5 | 0.8 |
| Congo | 1.9 | 0.2 | 0.6 |
| Congo DR | 2.1 | 1.3 | 5.3 |
| Cote d'Ivoire | 4.6 | 1.6 | 3.6 |
| Dominican Republic | 0.2 | 0.0 | 0.1 |
| Egypt | 3.6 | 0.3 | 0.5 |
| Ethiopia | 10.7 | 6.1 | 10.2 |
| Gabon | 0.1 | -0.1 | 1.4 |
| Ghana | 2.5 | 1.1 | 2.9 |
| Guatemala | 6.6 | 0.2 | 0.3 |
| Guinea | 1.0 | 1.0 | 8.1 |
| Haiti | 3.8 | 2.5 | 7.3 |
| Honduras | 19.2 | 0.0 | 0.0 |
| India | 4.4 | 1.0 | 1.7 |
| Indonesia | 1.7 | 1.1 | 4.3 |
| Jordan | 0.4 | 0.0 | 0.0 |
| Kazakhstan | -1.9 | -6.3 | 5.4 |
| Kenya | 1.6 | 1.0 | 4.7 |
| Kyrgyz Republic | 0.1 | 0.0 | 0.0 |
| Lesotho | 5.2 | 0.9 | 1.4 |
| Liberia | 4.6 | 1.2 | 2.7 |
| Madagascar | 3.3 | 1.5 | 4.2 |
| Malawi | 3.0 | 0.7 | 1.2 |
| Maldives | 1.7 | 1.8 | 7.9 |
| Mali | 3.1 | 2.2 | 7.4 |
| Morocco | 5.7 | 0.4 | 0.6 |
| Mozambique | -1.2 | -0.7 | 12.7 |
| Namibia | 1.2 | 0.4 | 0.9 |
| Nepal | 10.7 | 2.6 | 3.4 |
| Nicaragua | 0.7 | -0.1 | 3.2 |
| Niger | 3.4 | 2.5 | 9.4 |
| Nigeria | 0.9 | 0.8 | 8.5 |
| Pakistan | 5.0 | 1.9 | 3.4 |
| Peru | 3.1 | 0.4 | 0.7 |
| Philippines | 3.3 | 0.8 | 1.5 |
| Rwanda | 7.2 | 0.8 | 1.1 |
| Senegal | 1.2 | 0.9 | 4.4 |
| Sierra Leone | 7.9 | 2.1 | 3.5 |
| South Africa | 0.9 | 0.1 | 0.3 |
| Tajikistan | 6.0 | 0.2 | 0.3 |
| Tanzania | 1.9 | 1.3 | 4.8 |
| Timor-Leste | 11.8 | 2.5 | 3.2 |
| Togo | 2.3 | 1.6 | 6.5 |
| Turkey | 0.6 | 0.2 | 1.0 |
| Uganda | 5.6 | 1.6 | 2.6 |
| Vietnam | 4.6 | 0.1 | 0.3 |
| Yemen | 4.3 | 2.2 | 5.5 |
| Zambia | 1.4 | 0.9 | 4.5 |
| Zimbabwe | 0.3 | 0.2 | 2.7 |

**Note:** AARC is calculated as ln((rate in latest year/rate in earliest year))/ (latest year - earliest year), with positive values denote increasing rate while negative values denote decreasing rate

Table S39: Predicted average annual rate of increase (AARC) of skilled birth attendance among women 15-49 years of age in low- and middle-income countries by wealth quintiles

| **Country** | **Poorest** | | | **Poorer** | | | | **Middle** | | | | **Richer** | | | | **Richest** | | | |
| --- | --- | --- | --- | --- | --- | --- | --- | --- | --- | --- | --- | --- | --- | --- | --- | --- | --- | --- | --- |
|  | **Predicted** | | **Required** | **Predicted** | | **Required** | **Predicted** | | | **Required** | **Predicted** | | | **Required** | **Predicted** | | | **Required** |  |
|  | **1990-2018** | **2019-2030** | **2019-2030** | **1990-2018** | **2019-2030** | **2019-2030** | **1990-2018** | | **2019-2030** | **2019-2030** | **1990-2018** | | **2019-2030** | **2019-2030** | **1990-2018** | | **2019-2030** | **2019-2030** |  |
| Afghanistan | 7.1 | 5.9 | 10.1 | 4.1 | 3.5 | 8.3 | 0.1 | | 1.0 | 8.9 | 5.7 | | 1.9 | 3.6 | 4.5 | | 0.7 | 1.4 |  |
| Albania | -1.1 | -3.6 | 3.7 | -1.0 | -3.4 | 3.4 | -0.6 | | -1.3 | 3.1 | 0.5 | | 0.1 | 2.8 | 0.4 | | 0.0 | 2.1 |  |
| Angola | -1.6 | 0.1 | 17.4 | -4.6 | -5.4 | 16.0 | -4.0 | | -7.9 | 12.1 | -4.5 | | -20.3 | 13.1 | -4.3 | | -26.4 | 13.0 |  |
| Armenia | 2.4 | 0.0 | 0.0 | 7.6 | 0.0 | 0.0 | 0.0 | | 0.0 | 0.0 | 1.9 | | 0.0 | 0.0 | -4.3 | | -26.4 | 13.0 |  |
| Bangladesh | 9.5 | 6.8 | 13.9 | 10.8 | 5.9 | 10.0 | 11.0 | | 4.4 | 6.4 | 9.5 | | 2.8 | 3.9 | 4.8 | | 1.2 | 2.0 |  |
| Benin | 4.2 | 1.4 | 2.5 | 2.9 | 0.8 | 1.7 | 1.8 | | 0.6 | 1.2 | 1.0 | | 0.2 | 0.4 | 0.1 | | 0.0 | 0.2 |  |
| Bolivia | 7.5 | 2.2 | 3.6 | 4.0 | 0.9 | 1.7 | 2.8 | | 0.4 | 0.6 | 1.1 | | 0.1 | 0.2 | 0.2 | | 0.0 | 0.0 |  |
| Burkina Faso | 0.1 | 0.8 | 17.5 | 2.4 | 2.4 | 12.9 | 0.8 | | 1.2 | 12.8 | 0.2 | | 0.5 | 10.0 | -1.0 | | -1.5 | 4.4 |  |
| Burundi | 9.6 | 1.3 | 1.6 | 12.2 | 0.9 | 1.0 | 11.7 | | 0.8 | 0.9 | 10.5 | | 0.7 | 0.9 | 10.6 | | 0.2 | 0.2 |  |
| Cambodia | 14.2 | 0.9 | 1.1 | 13.7 | 0.4 | 0.4 | 13.2 | | 0.2 | 0.2 | 11.6 | | 0.1 | 0.1 | 3.6 | | 0.0 | 0.0 |  |
| Cameroon | -1.7 | -1.3 | 15.8 | 0.6 | 0.6 | 6.9 | 1.4 | | 0.7 | 2.6 | 0.4 | | 0.2 | 1.3 | 0.0 | | 0.0 | 0.8 |  |
| Chad | 13.5 | 12.0 | 22.7 | 11.6 | 9.3 | 18.5 | 7.7 | | 6.9 | 18.0 | 3.1 | | 4.2 | 19.5 | 3.8 | | 2.0 | 5.8 |  |
| Colombia | 2.0 | 0.5 | 1.0 | 1.1 | 0.1 | 0.1 | 0.3 | | 0.0 | 0.1 | 0.0 | | 0.0 | 0.1 | 0.0 | | 0.0 | 0.1 |  |
| Comoros | 7.0 | 1.3 | 1.8 | 6.2 | 0.6 | 0.9 | 4.7 | | 0.3 | 0.3 | 3.7 | | 0.1 | 0.2 | 1.8 | | 0.2 | 0.2 |  |
| Congo | 1.4 | 0.4 | 2.4 | 4.2 | 0.3 | 0.4 | 4.1 | | 0.1 | 0.1 | 0.2 | | 0.0 | 0.1 | 0.1 | | 0.0 | 0.1 |  |
| Congo DR | 5.0 | 2.6 | 5.8 | 2.5 | 2.0 | 6.9 | 0.3 | | 0.4 | 6.9 | 0.9 | | 0.5 | 4.9 | 1.8 | | 0.6 | 2.7 |  |
| Cote d'Ivoire | 8.0 | 3.4 | 6.5 | 3.5 | 2.2 | 7.5 | 5.3 | | 1.5 | 3.1 | 4.6 | | 0.6 | 1.0 | 3.1 | | 0.4 | 0.7 |  |
| Dominican Republic | 0.3 | 0.1 | 0.2 | 0.0 | 0.0 | 0.0 | -0.1 | | -0.9 | 0.3 | 0.1 | | 0.0 | 0.0 | 0.3 | | 0.0 | 0.0 |  |
| Egypt | 7.5 | 0.7 | 0.9 | 5.5 | 0.5 | 0.7 | 4.3 | | 0.2 | 0.3 | 2.8 | | 0.1 | 0.1 | 0.8 | | 0.0 | 0.0 |  |
| Ethiopia | 14.9 | 11.3 | 18.6 | 17.5 | 9.5 | 13.0 | 16.0 | | 9.0 | 12.9 | 14.2 | | 6.6 | 9.2 | 8.4 | | 1.7 | 2.3 |  |
| Gabon | 1.3 | 0.6 | 2.3 | 0.4 | 0.1 | 1.2 | -0.1 | | -0.3 | 0.8 | 0.0 | | -0.1 | 0.6 | -1.3 | | -5.8 | 3.7 |  |
| Ghana | 2.7 | 2.1 | 8.8 | 4.5 | 1.9 | 4.1 | 3.9 | | 1.1 | 1.9 | 2.2 | | 0.4 | 0.7 | 0.6 | | 0.2 | 0.3 |  |
| Guatemala | 13.3 | 0.5 | 0.5 | 10.7 | 0.3 | 0.3 | 7.8 | | 0.1 | 0.1 | 3.7 | | 0.0 | 0.0 | 0.6 | | 0.0 | 0.0 |  |
| Guinea | -0.8 | 0.6 | 20.7 | 2.9 | 2.8 | 12.0 | 1.7 | | 1.6 | 9.6 | 0.8 | | 0.5 | 5.0 | 0.7 | | 0.2 | 0.8 |  |
| Haiti | 8.8 | 6.8 | 15.1 | 8.1 | 5.1 | 10.2 | 6.7 | | 3.3 | 6.3 | 3.0 | | 1.7 | 4.5 | 1.6 | | 0.6 | 1.6 |  |
| Honduras | 24.9 | 0.0 | 0.0 | 30.6 | 0.0 | 0.0 | 19.8 | | 0.0 | 0.0 | 12.2 | | 0.0 | 0.0 | 1.3 | | 0.0 | 0.0 |  |
| India | 8.3 | 2.5 | 3.8 | 7.3 | 1.4 | 2.0 | 5.5 | | 0.8 | 1.1 | 3.5 | | 0.4 | 0.7 | 1.0 | | 0.2 | 0.3 |  |
| Indonesia | 3.1 | 2.4 | 9.1 | 2.7 | 1.5 | 5.1 | 1.9 | | 1.0 | 3.5 | 0.4 | | 0.2 | 3.3 | -1.9 | | -4.3 | 5.9 |  |
| Jordan | 0.8 | 0.0 | 0.0 | 0.7 | 0.0 | 0.0 | 0.5 | | 0.0 | 0.0 | 0.1 | | 0.0 | 0.0 | 0.2 | | 0.0 | 0.0 |  |
| Kazakhstan | -3.7 | -8.5 | 10.4 | -46.4 | -65.3 | 124.8 | -0.2 | | -0.6 | 0.7 | 0.0 | | -0.1 | 0.0 | -47.7 | | -73.0 | 128.6 |  |
| Kenya | 1.8 | 1.6 | 10.7 | 2.0 | 1.4 | 6.5 | 1.9 | | 1.2 | 4.7 | 1.9 | | 0.8 | 2.2 | 1.1 | | 0.3 | 0.8 |  |
| Kyrgyz Republic | 0.2 | 0.0 | 0.1 | 0.2 | 0.0 | 0.0 | 0.1 | | 0.0 | 0.1 | -0.1 | | -0.6 | 0.3 | 0.0 | | -0.2 | 0.0 |  |
| Lesotho | 7.4 | 1.8 | 2.8 | 7.5 | 1.3 | 1.8 | 5.2 | | 0.8 | 1.2 | 4.4 | | 0.4 | 0.6 | 2.7 | | 0.2 | 0.3 |  |
| Liberia | 7.0 | 2.4 | 4.3 | 9.5 | 1.7 | 2.4 | 6.2 | | 1.3 | 2.4 | 1.4 | | 0.3 | 2.0 | 3.1 | | 0.3 | 0.7 |  |
| Madagascar | -2.3 | -1.7 | 15.8 | -2.3 | -2.0 | 13.6 | 0.3 | | 0.3 | 7.6 | -0.9 | | -1.1 | 5.9 | 0.1 | | 0.0 | 0.9 |  |
| Malawi | 4.0 | 0.9 | 1.6 | 3.7 | 0.8 | 1.5 | 3.4 | | 0.8 | 1.4 | 2.8 | | 0.5 | 0.9 | 1.1 | | 0.2 | 0.4 |  |
| Maldives | -2.6 | -2.6 | 12.0 | -1.9 | -1.6 | 10.8 | 0.3 | | 1.0 | 9.8 | 4.8 | | 2.6 | 5.5 | 12.0 | | 2.7 | 3.4 |  |
| Mali | 8.7 | 5.9 | 12.3 | 7.1 | 4.8 | 10.9 | 5.4 | | 3.8 | 10.3 | 3.6 | | 1.9 | 5.0 | 1.0 | | 0.4 | 1.2 |  |
| Morocco | 12.6 | 0.8 | 0.9 | 8.0 | 0.6 | 0.8 | 6.5 | | 0.2 | 0.2 | 3.9 | | 0.1 | 0.1 | 2.8 | | 0.0 | 0.0 |  |
| Mozambique | 0.9 | 1.7 | 17.3 | -1.3 | -0.2 | 16.9 | 0.8 | | 1.2 | 12.3 | -2.2 | | -2.3 | 11.0 | -1.7 | | -2.5 | 7.0 |  |
| Namibia | 1.8 | 0.8 | 2.3 | 2.1 | 0.5 | 0.9 | 1.2 | | 0.3 | 0.7 | 1.1 | | 0.1 | 0.2 | 0.3 | | 0.0 | 0.1 |  |
| Nepal | 14.2 | 6.1 | 8.4 | 14.4 | 3.7 | 4.6 | 13.5 | | 2.4 | 2.9 | 12.6 | | 1.5 | 1.7 | 6.6 | | 0.5 | 0.6 |  |
| Nicaragua | -2.4 | -0.1 | 12.6 | 1.2 | 0.1 | 3.0 | 1.7 | | -0.1 | 0.7 | 0.1 | | -0.4 | 1.5 | 4.0 | | 0.0 | 0.0 |  |
| Niger | 8.0 | 6.1 | 14.1 | 7.5 | 4.9 | 10.8 | 7.8 | | 4.7 | 9.7 | 4.7 | | 3.2 | 8.9 | 1.1 | | 0.6 | 2.7 |  |
| Nigeria | -7.0 | -6.0 | 30.1 | -1.4 | -0.1 | 16.9 | 2.5 | | 2.0 | 7.8 | 1.6 | | 0.9 | 4.2 | -0.3 | | -0.7 | 2.3 |  |
| Pakistan | 8.5 | 4.0 | 6.8 | 8.8 | 3.0 | 4.5 | 9.6 | | 1.9 | 2.5 | 5.4 | | 1.1 | 1.6 | 2.0 | | 0.4 | 0.8 |  |
| Peru | 8.3 | 1.6 | 2.2 | 4.2 | 0.5 | 0.7 | 2.3 | | 0.1 | 0.1 | 1.0 | | 0.0 | 0.0 | 0.5 | | 0.0 | 0.0 |  |
| Philippines | 6.8 | 2.5 | 4.3 | 5.2 | 1.0 | 1.5 | 2.9 | | 0.4 | 0.7 | 2.2 | | 0.2 | 0.2 | 0.7 | | 0.1 | 0.2 |  |
| Rwanda | 8.2 | 1.4 | 1.9 | 8.8 | 1.0 | 1.3 | 8.3 | | 0.9 | 1.1 | 7.7 | | 0.7 | 0.9 | 4.9 | | 0.3 | 0.3 |  |
| Senegal | 3.4 | 2.7 | 9.8 | 4.4 | 2.4 | 5.3 | 2.5 | | 1.2 | 3.1 | 0.6 | | 0.3 | 2.0 | 0.1 | | 0.0 | 1.1 |  |
| Sierra Leone | 12.8 | 3.1 | 4.2 | 9.2 | 2.7 | 4.1 | 7.1 | | 2.6 | 4.6 | 8.5 | | 1.6 | 2.5 | 6.7 | | 0.6 | 1.0 |  |
| South Africa | 1.9 | 0.3 | 0.6 | 1.3 | 0.1 | 0.2 | 0.6 | | 0.1 | 0.1 | 0.1 | | 0.0 | 0.1 | 0.0 | | -0.1 | 0.2 |  |
| Tajikistan | 10.9 | 0.5 | 0.6 | 4.4 | 0.3 | 0.5 | 4.5 | | 0.2 | 0.3 | 5.5 | | 0.1 | 0.2 | 2.1 | | 0.1 | 0.1 |  |
| Tanzania | 4.1 | 2.7 | 7.6 | 2.6 | 1.8 | 6.7 | 4.1 | | 2.0 | 4.7 | 3.9 | | 1.2 | 2.2 | 1.3 | | 0.3 | 0.5 |  |
| Timor-Leste | 13.8 | 6.2 | 7.9 | 16.9 | 4.3 | 5.0 | 17.9 | | 2.5 | 2.7 | 15.1 | | 1.3 | 1.4 | 11.6 | | 0.4 | 0.5 |  |
| Togo | 0.7 | 1.4 | 19.0 | 2.0 | 2.1 | 13.4 | 1.8 | | 1.5 | 8.4 | 2.8 | | 1.0 | 2.2 | 0.8 | | 0.3 | 1.2 |  |
| Turkey | 2.1 | 0.7 | 2.2 | 0.7 | 0.2 | 1.3 | 0.4 | | 0.1 | 0.4 | 0.1 | | 0.0 | 0.2 | 0.0 | | 0.0 | 0.0 |  |
| Uganda | 7.5 | 2.3 | 3.8 | 5.9 | 2.0 | 3.6 | 6.5 | | 1.8 | 2.8 | 5.9 | | 1.3 | 2.0 | 4.0 | | 0.4 | 0.5 |  |
| Vietnam | 4.9 | 0.6 | 1.7 | 4.9 | 0.2 | 0.4 | 5.4 | | 0.0 | 0.1 | 7.4 | | 0.0 | 0.0 | 6.7 | | 0.0 | 0.0 |  |
| Zambia | 5.8 | 2.8 | 5.7 | 5.2 | 2.4 | 5.3 | 3.9 | | 1.7 | 4.0 | 1.9 | | 0.6 | 1.2 | 0.2 | | 0.1 | 0.5 |  |
| Zimbabwe | 0.3 | 0.2 | 5.4 | 0.5 | 0.3 | 3.8 | 0.7 | | 0.4 | 2.7 | 0.5 | | 0.3 | 1.2 | 0.2 | | 0.1 | 0.4 |  |

**Note:** AARC is calculated as ln((rate in latest year/rate in earliest year))/ (latest year - earliest year), with positive values denote increasing rate while negative values denote decreasing rate

Table S40: Predicted average annual rate of increase (AARC) of skilled birth attendance among women 15-49 years of age in low- and middle-income countries by place of residence

| **Country** | **Rural** | | | **Urban** | | |
| --- | --- | --- | --- | --- | --- | --- |
|  | **Predicted** | | **Required** | **Predicted** | | **Required** |
|  | **1990-2018** | **2019-2030** | **2019-2030** | **1990-2018** | **2019-2030** | **2019-2030** |
| Afghanistan | 3.6 | 3.0 | 7.5 | 0.8 | 0.0 | 2.6 |
| Albania | -1.0 | -2.6 | 3.8 | -0.2 | -0.7 | 2.5 |
| Angola | -4.9 | -5.1 | 18.4 | -4.4 | -16.9 | 12.7 |
| Armenia | 2.8 | 0.0 | 0.0 | 1.9 | 0.0 | 0.0 |
| Bangladesh | 9.2 | 4.5 | 7.6 | 2.9 | 1.6 | 4.6 |
| Benin | 2.5 | 0.8 | 1.5 | 1.1 | 0.3 | 0.6 |
| Bolivia | 4.5 | 1.7 | 3.3 | 1.8 | 0.3 | 0.4 |
| Brazil | 2.2 | 0.1 | 0.3 | 0.9 | 0.0 | 0.1 |
| Burkina Faso | 0.3 | 0.9 | 13.6 | -1.8 | -4.0 | 5.7 |
| Burundi | 10.6 | 0.9 | 1.0 | 6.5 | 0.2 | 0.2 |
| Cambodia | 11.2 | 0.4 | 0.5 | 9.9 | 0.0 | 0.0 |
| Cameroon | -0.3 | -0.3 | 8.3 | 0.1 | 0.0 | 1.7 |
| Chad | 8.7 | 7.9 | 19.1 | 3.5 | 2.0 | 6.2 |
| Colombia | 1.3 | 0.4 | 1.0 | 0.4 | 0.0 | 0.1 |
| Comoros | 5.0 | 0.7 | 1.0 | 2.7 | 0.2 | 0.2 |
| Congo | 1.6 | 0.3 | 1.6 | 0.1 | -0.1 | 0.2 |
| Congo DR | 3.9 | 2.2 | 5.6 | 0.9 | 0.4 | 4.1 |
| Cote d'Ivoire | 5.1 | 2.4 | 5.6 | 3.3 | 0.7 | 1.4 |
| Dominican Republic | 0.4 | 0.1 | 0.1 | 0.1 | 0.0 | 0.1 |
| Egypt | 5.2 | 0.4 | 0.5 | 1.9 | 0.1 | 0.2 |
| Ethiopia | 13.8 | 8.5 | 13.1 | 6.8 | 1.1 | 1.5 |
| Gabon | 0.2 | 0.0 | 3.5 | -0.3 | -0.7 | 1.3 |
| Ghana | 3.2 | 1.7 | 4.9 | 0.6 | 0.3 | 1.2 |
| Guatemala | 9.1 | 0.3 | 0.3 | 3.6 | 0.1 | 0.1 |
| Guinea | 1.3 | 1.7 | 12.4 | 0.5 | 0.2 | 1.7 |
| Haiti | 6.6 | 4.2 | 9.4 | 1.8 | 1.1 | 4.1 |
| Honduras | 22.7 | 0.0 | 0.0 | 11.3 | 0.0 | 0.0 |
| India | 5.6 | 1.3 | 2.1 | 1.6 | 0.4 | 0.9 |
| Indonesia | 2.4 | 1.6 | 6.5 | -0.3 | -0.4 | 3.5 |
| Jordan | 0.9 | 0.0 | 0.0 | 0.2 | 0.0 | 0.0 |
| Kazakhstan | -0.5 | -1.9 | 1.6 | -47.0 | -63.4 | 126.1 |
| Kenya | 1.3 | 1.0 | 6.5 | 0.5 | 0.3 | 1.9 |
| Kyrgyz Republic | 0.1 | 0.0 | 0.1 | 0.0 | 0.0 | 0.0 |
| Lesotho | 5.7 | 1.0 | 1.7 | 0.1 | -0.1 | 1.0 |
| Liberia | 7.4 | 1.9 | 3.2 | -1.0 | -2.4 | 3.9 |
| Madagascar | 3.1 | 1.6 | 5.3 | 4.6 | 0.3 | 0.4 |
| Malawi | 3.4 | 0.7 | 1.3 | 0.6 | 0.2 | 0.7 |
| Maldives | -2.6 | -2.4 | 12.2 | 10.8 | 2.5 | 3.3 |
| Mali | 7.8 | 4.5 | 9.0 | 1.3 | 0.5 | 1.3 |
| Morocco | 7.6 | 1.1 | 1.6 | 3.4 | 0.1 | 0.1 |
| Mozambique | -0.5 | 0.2 | 14.8 | -3.0 | -4.7 | 10.4 |
| Namibia | 1.5 | 0.6 | 1.4 | 0.3 | 0.1 | 0.3 |
| Nepal | 11.4 | 3.2 | 4.4 | 2.8 | 1.0 | 2.2 |
| Nicaragua | 1.3 | 0.3 | 4.8 | 1.4 | -0.1 | 0.5 |
| Niger | 6.9 | 4.6 | 10.8 | 1.0 | 0.4 | 1.5 |
| Nigeria | 0.0 | 0.2 | 12.9 | 0.3 | 0.2 | 4.2 |
| Pakistan | 7.8 | 2.5 | 3.9 | 2.7 | 0.9 | 1.8 |
| Peru | 7.2 | 1.1 | 1.4 | 1.6 | 0.1 | 0.1 |
| Philippines | 5.3 | 1.3 | 2.2 | 1.9 | 0.4 | 0.8 |
| Rwanda | 7.9 | 1.0 | 1.3 | 2.7 | 0.4 | 0.7 |
| Senegal | 2.8 | 1.9 | 6.2 | -0.1 | -0.2 | 1.9 |
| Sierra Leone | 10.7 | 2.7 | 3.8 | 5.5 | 0.9 | 1.7 |
| South Africa | 1.6 | 0.2 | 0.3 | 0.1 | 0.0 | 0.2 |
| Tajikistan | 6.7 | 0.3 | 0.4 | 4.3 | 0.1 | 0.2 |
| Tanzania | 2.5 | 1.7 | 6.1 | 0.5 | 0.3 | 1.3 |
| Timor-Leste | 13.2 | 3.6 | 4.5 | 12.8 | 0.6 | 0.7 |
| Togo | 1.8 | 1.8 | 11.9 | 0.6 | 0.3 | 1.9 |
| Turkey | 1.0 | 0.3 | 2.0 | 0.2 | 0.0 | 0.8 |
| Uganda | 6.0 | 1.8 | 3.0 | 2.3 | 0.5 | 0.9 |
| Vietnam | 5.1 | 0.2 | 0.3 | 2.8 | 0.0 | 0.0 |
| Yemen | 5.1 | 3.0 | 7.3 | 1.8 | 0.8 | 2.1 |
| Zambia | 3.6 | 2.1 | 5.8 | 0.8 | 0.3 | 0.9 |
| Zimbabwe | 0.5 | 0.3 | 3.7 | 0.0 | 0.0 | 0.8 |

**Note:** AARC is calculated as ln((rate in latest year/rate in earliest year))/ (latest year - earliest year), with positive values denote increasing rate while negative values denote decreasing rate

Table S41: Predicted average annual rate of increase (AARC) of skilled birth attendance among women 15-49 years of age in low- and middle-income countries by education

| **Country** | **Below secondary education** | | | **Secondary+ education** | | |
| --- | --- | --- | --- | --- | --- | --- |
|  | **Predicted** | | **Required** | **Predicted** | | **Required** |
|  | **1990-2018** | **2019-2030** | **2019-2030** | **1990-2018** | **2019-2030** | **2019-2030** |
| Afghanistan | -3.8 | -8.2 | 11.3 | 0.2 | -0.6 | 2.2 |
| Albania | -0.6 | -1.6 | 3.1 | -0.4 | -1.1 | 2.9 |
| Angola | -5.9 | -11.3 | 17.1 | -4.5 | -29.0 | 13.5 |
| Armenia | 4.7 | 0.0 | 0.0 | 2.4 | 0.0 | 0.0 |
| Bangladesh | 8.2 | 5.5 | 11.4 | 3.0 | 1.9 | 5.7 |
| Benin | 2.1 | 0.6 | 1.3 | 0.2 | 0.1 | 0.2 |
| Bolivia | 4.3 | 1.2 | 2.2 | 0.9 | 0.2 | 0.4 |
| Brazil | 1.5 | 0.1 | 0.3 | 0.3 | 0.0 | 0.2 |
| Burkina Faso | -0.4 | 0.0 | 12.5 | -0.5 | -1.0 | 2.6 |
| Burundi | 10.2 | 0.9 | 1.0 | 2.7 | 0.2 | 0.3 |
| Cambodia | 11.3 | 0.5 | 0.6 | 5.2 | 0.1 | 0.1 |
| Cameroon | -0.5 | -0.5 | 7.9 | 0.2 | 0.1 | 1.0 |
| Chad | 4.5 | 4.7 | 17.4 | 0.2 | 0.4 | 7.6 |
| Colombia | 0.9 | 0.4 | 1.1 | 0.1 | 0.1 | 0.2 |
| Comoros | 4.5 | 0.7 | 1.1 | 2.1 | 0.2 | 0.2 |
| Congo | 1.7 | 0.3 | 1.3 | 1.6 | 0.1 | 0.2 |
| Congo DR | 3.1 | 1.9 | 5.6 | 0.3 | 0.1 | 5.1 |
| Cote d'Ivoire | 5.0 | 1.7 | 3.8 | 1.6 | 0.5 | 2.0 |
| Dominican Republic | 0.3 | 0.1 | 0.1 | 0.0 | 0.0 | 0.0 |
| Egypt | 4.6 | 0.7 | 1.1 | 1.3 | 0.2 | 0.4 |
| Ethiopia | 13.1 | 7.3 | 11.2 | 4.4 | 0.9 | 1.4 |
| Gabon | 0.4 | 0.1 | 1.8 | -0.3 | -0.8 | 1.6 |
| Ghana | 2.0 | 1.3 | 5.6 | 0.4 | 0.2 | 2.1 |
| Guatemala | 7.6 | 0.3 | 0.3 | 1.1 | 0.1 | 0.1 |
| Guinea | 0.7 | 0.9 | 9.3 | -0.6 | -1.2 | 3.1 |
| Haiti | 3.0 | 2.6 | 11.8 | 0.3 | 0.3 | 4.3 |
| Honduras | 20.3 | 0.0 | 0.0 | 3.9 | 0.0 | 0.0 |
| India | 5.3 | 1.8 | 3.2 | 1.2 | 0.4 | 1.0 |
| Indonesia | 2.3 | 1.6 | 6.7 | -1.0 | -1.4 | 5.2 |
| Jordan | 0.9 | 0.0 | 0.0 | 0.2 | 0.0 | 0.0 |
| Kazakhstan | 0.0 | -0.1 | 0.0 | -2.2 | -6.9 | 6.2 |
| Kenya | 1.6 | 1.2 | 6.4 | 0.8 | 0.4 | 1.7 |
| Kyrgyz Republic | 0.0 | 0.0 | 0.0 | 0.1 | 0.0 | 0.0 |
| Lesotho | 4.6 | 1.3 | 2.6 | 3.6 | 0.4 | 0.7 |
| Liberia | 4.6 | 1.6 | 3.5 | 1.3 | 0.2 | 1.7 |
| Madagascar | 2.9 | 1.7 | 6.1 | 3.6 | 0.4 | 0.6 |
| Malawi | 3.0 | 0.7 | 1.4 | 0.1 | 0.0 | 0.9 |
| Maldives | -1.4 | -1.0 | 10.2 | 3.5 | 2.7 | 7.3 |
| Mali | 3.3 | 2.4 | 8.1 | -0.6 | -0.9 | 2.9 |
| Morocco | 6.4 | 0.6 | 0.8 | 1.9 | 0.0 | 0.0 |
| Mozambique | -1.8 | -1.4 | 14.3 | -3.8 | -8.4 | 11.1 |
| Namibia | 1.0 | 0.6 | 2.5 | 0.3 | 0.1 | 0.5 |
| Nepal | 11.4 | 4.1 | 5.7 | 3.9 | 1.2 | 2.2 |
| Nicaragua | 0.1 | -0.1 | 5.3 | 1.1 | -0.1 | 0.4 |
| Niger | 3.4 | 2.7 | 10.2 | 0.0 | -0.1 | 2.4 |
| Nigeria | -0.8 | -0.7 | 15.1 | -0.3 | -0.4 | 3.5 |
| Pakistan | 6.0 | 2.5 | 4.6 | 1.4 | 0.5 | 1.3 |
| Peru | 5.8 | 1.1 | 1.7 | 1.2 | 0.2 | 0.2 |
| Philippines | 4.4 | 2.1 | 4.8 | 2.3 | 0.5 | 1.0 |
| Rwanda | 7.8 | 0.9 | 1.2 | 2.8 | 0.2 | 0.3 |
| Senegal | 1.3 | 1.0 | 4.9 | -0.4 | -0.8 | 2.0 |
| Sierra Leone | 8.0 | 2.5 | 4.1 | 2.3 | 0.6 | 2.8 |
| South Africa | 1.4 | 0.3 | 0.6 | 0.3 | 0.1 | 0.2 |
| Tajikistan | 3.8 | 0.6 | 1.1 | 6.5 | 0.2 | 0.3 |
| Tanzania | 1.8 | 1.2 | 5.4 | 0.1 | 0.1 | 1.5 |
| Timor-Leste | 11.3 | 4.5 | 6.2 | 8.5 | 1.5 | 2.1 |
| Togo | 2.0 | 1.6 | 8.0 | 0.0 | -0.1 | 3.7 |
| Turkey | 0.5 | 0.1 | 1.6 | -0.1 | -0.2 | 0.3 |
| Uganda | 5.8 | 1.9 | 3.3 | 2.5 | 0.6 | 1.1 |
| Vietnam | 4.7 | 0.4 | 0.9 | 5.1 | 0.0 | 0.1 |
| Yemen | 4.1 | 2.4 | 6.5 | -0.9 | -1.8 | 3.5 |
| Zambia | 1.6 | 1.1 | 5.8 | 0.1 | 0.0 | 2.3 |
| Zimbabwe | 0.1 | 0.1 | 5.1 | -0.3 | -0.5 | 2.2 |

**Note:** AARC is calculated as ln((rate in latest year/rate in earliest year))/ (latest year - earliest year), with positive values denote increasing rate while negative values denote decreasing rate

Table S42: Predicted average annual rate of increase (AARC) of skilled birth attendance among women 15-49 years of age in low- and middle-income countries by age

| **Country** | **Adolescent** | | | **Adult** | | |
| --- | --- | --- | --- | --- | --- | --- |
|  | **Predicted** | | **Required** | **Predicted** | | **Required** |
|  | **1990-2018** | **2019-2030** | **2019-2030** | **1990-2018** | **2019-2030** | **2019-2030** |
| Afghanistan | 0.0 | 0.3 | 7.3 | 3.5 | 2.2 | 5.8 |
| Albania | -0.2 | -0.5 | 4.6 | -0.6 | -1.4 | 3.0 |
| Angola | -4.5 | -9.9 | 13.2 | -4.4 | -7.8 | 13.5 |
| Armenia | 0.1 | 0.0 | 0.0 | 2.6 | 0.0 | 0.0 |
| Bangladesh | 8.9 | 3.8 | 6.2 | 8.0 | 3.6 | 6.2 |
| Benin | 1.9 | 0.6 | 1.3 | 2.1 | 0.6 | 1.1 |
| Bolivia | 1.9 | 0.6 | 1.4 | 2.6 | 0.8 | 1.6 |
| Brazil | 0.9 | 0.0 | 0.4 | 1.6 | 0.0 | 0.0 |
| Burkina Faso | -0.7 | -0.3 | 11.3 | -0.3 | 0.1 | 11.7 |
| Burundi | 5.8 | 0.4 | 0.5 | 10.5 | 0.8 | 1.0 |
| Cambodia | 9.6 | 0.4 | 0.4 | 10.8 | 0.4 | 0.4 |
| Cameroon | 0.5 | 0.3 | 4.4 | 0.1 | 0.1 | 4.8 |
| Chad | 4.0 | 3.9 | 14.2 | 4.8 | 4.6 | 15.3 |
| Colombia | 0.6 | 0.1 | 0.3 | 0.7 | 0.2 | 0.3 |
| Comoros | 4.7 | 0.5 | 0.7 | 4.3 | 0.5 | 0.8 |
| Congo | 0.9 | 0.1 | 0.6 | 2.0 | 0.2 | 0.6 |
| Congo DR | 2.7 | 1.3 | 4.1 | 2.1 | 1.3 | 5.4 |
| Cote d'Ivoire | 3.6 | 1.6 | 4.8 | 4.6 | 1.6 | 3.6 |
| Dominican Republic | 0.3 | 0.0 | 0.0 | 0.2 | 0.0 | 0.1 |
| Egypt | 3.6 | 0.3 | 0.4 | 3.7 | 0.3 | 0.4 |
| Ethiopia | 8.7 | 5.4 | 10.1 | 11.0 | 6.1 | 10.1 |
| Gabon | 0.1 | -0.1 | 1.3 | 0.1 | -0.1 | 1.4 |
| Ghana | 2.3 | 1.0 | 2.9 | 2.6 | 1.1 | 2.9 |
| Guatemala | 6.4 | 0.2 | 0.2 | 6.6 | 0.2 | 0.3 |
| Guinea | 0.1 | 0.3 | 8.2 | 1.2 | 1.1 | 8.0 |
| Haiti | 1.9 | 1.5 | 7.7 | 4.1 | 2.6 | 7.2 |
| Honduras | 19.1 | 0.0 | 0.0 | 19.5 | 0.0 | 0.0 |
| India | 5.0 | 0.9 | 1.5 | 4.3 | 1.0 | 1.7 |
| Indonesia | 2.2 | 1.4 | 5.6 | 1.7 | 1.0 | 4.3 |
| Jordan | 0.2 | 0.0 | 0.0 | 0.4 | 0.0 | 0.0 |
| Kazakhstan | 0.0 | -0.1 | 0.0 | -1.9 | -6.1 | 5.4 |
| Kenya | 1.6 | 1.0 | 3.9 | 1.6 | 1.1 | 4.7 |
| Kyrgyz Republic | 0.4 | 0.0 | 0.0 | 0.1 | 0.0 | 0.0 |
| Lesotho | 5.3 | 0.6 | 0.9 | 5.2 | 0.9 | 1.4 |
| Liberia | 6.9 | 1.1 | 1.7 | 4.5 | 1.3 | 2.8 |
| Madagascar | 4.1 | 1.8 | 4.3 | 3.3 | 1.5 | 4.0 |
| Malawi | 3.1 | 0.5 | 0.8 | 3.0 | 0.7 | 1.3 |
| Maldives | 2.7 | 2.8 | 9.6 | 1.8 | 1.8 | 7.9 |
| Mali | 2.0 | 1.6 | 7.6 | 3.3 | 2.2 | 7.5 |
| Morocco | 4.2 | 0.5 | 0.7 | 5.9 | 0.4 | 0.6 |
| Mozambique | -0.8 | -0.5 | 11.2 | -1.3 | -0.8 | 12.9 |
| Namibia | 0.4 | 0.2 | 1.3 | 1.3 | 0.4 | 0.8 |
| Nepal | 10.1 | 1.9 | 2.5 | 10.8 | 2.7 | 3.5 |
| Nicaragua | -0.1 | -0.5 | 4.0 | 1.1 | 0.0 | 2.6 |
| Niger | 3.5 | 2.5 | 8.7 | 3.4 | 2.6 | 9.4 |
| Nigeria | -0.5 | -0.3 | 13.3 | 0.9 | 0.8 | 8.2 |
| Pakistan | 5.0 | 1.8 | 3.3 | 5.1 | 1.8 | 3.3 |
| Peru | 3.6 | 0.4 | 0.6 | 3.0 | 0.4 | 0.7 |
| Philippines | 4.7 | 0.8 | 1.2 | 3.2 | 0.8 | 1.5 |
| Rwanda | 6.9 | 0.3 | 0.3 | 7.3 | 0.9 | 1.2 |
| Senegal | 1.6 | 1.0 | 4.1 | 1.3 | 0.9 | 4.4 |
| Sierra Leone | 11.3 | 1.7 | 2.3 | 7.3 | 2.2 | 3.8 |
| South Africa | 0.4 | 0.1 | 0.3 | 0.9 | 0.1 | 0.3 |
| Tajikistan | 0.4 | -0.3 | 0.5 | 6.1 | 0.2 | 0.3 |
| Tanzania | 1.9 | 1.1 | 3.7 | 1.9 | 1.3 | 4.9 |
| Timor-Leste | 14.3 | 2.2 | 2.6 | 11.5 | 2.5 | 3.2 |
| Togo | 0.2 | 0.4 | 9.3 | 2.3 | 1.6 | 6.4 |
| Turkey | 0.0 | -0.2 | 1.7 | 0.7 | 0.2 | 1.0 |
| Uganda | 4.6 | 1.2 | 2.1 | 5.8 | 1.6 | 2.6 |
| Vietnam | 6.3 | 0.1 | 0.1 | 4.5 | 0.1 | 0.3 |
| Yemen | 3.3 | 1.8 | 4.9 | 4.3 | 2.3 | 5.5 |
| Zambia | 1.9 | 0.9 | 2.9 | 1.3 | 0.9 | 4.7 |
| Zimbabwe | 0.2 | 0.1 | 2.6 | 0.4 | 0.3 | 2.7 |

**Note:** AARC is calculated as ln((rate in latest year/rate in earliest year))/ (latest year - earliest year), with positive values denote increasing rate while negative values denote decreasing rate

Table S43: Predicted average annual rate of increase (AARC) of BCG immunization among children 12-23 months of age in low- and middle-income countries

| country | **Predicted** | | **Required** |
| --- | --- | --- | --- |
|  | **1990-2018** | **2019-2030** | **2019-2030** |
| Albania | 16.0 | 0.0 | 0.0 |
| Armenia | 0.8 | 0.0 | 0.0 |
| Bangladesh | 0.9 | 0.1 | 0.1 |
| Benin | 0.1 | 0.0 | 1.1 |
| Bolivia | 2.0 | 0.0 | 0.0 |
| Brazil | 1.6 | 0.0 | 0.0 |
| Burkina Faso | 1.6 | 0.1 | 0.2 |
| Burundi | -0.1 | -1.0 | 0.3 |
| Cambodia | 3.1 | 0.1 | 0.1 |
| Cameroon | 0.9 | 0.3 | 0.8 |
| Chad | 2.6 | 1.4 | 4.3 |
| Colombia | 0.2 | 0.1 | 0.2 |
| Comoros | -0.4 | -0.8 | 1.8 |
| Congo | 1.1 | 0.1 | 0.4 |
| Congo DR | 3.3 | 0.6 | 1.2 |
| Cote d'Ivoire | 0.5 | 0.2 | 1.4 |
| Dominican Republic | 0.8 | 0.2 | 0.3 |
| Egypt | 0.3 | 0.0 | 0.0 |
| Ethiopia | 2.7 | 1.1 | 2.6 |
| Gabon | 0.3 | 0.1 | 0.8 |
| Ghana | 0.9 | 0.1 | 0.2 |
| Guatemala | 1.2 | 0.1 | 0.1 |
| Guinea | 0.7 | 0.3 | 1.5 |
| Haiti | 0.8 | 0.4 | 1.6 |
| Honduras | 0.4 | 0.0 | 0.1 |
| India | 2.1 | 0.4 | 0.7 |
| Indonesia | 0.8 | 0.3 | 0.8 |
| Jordan | 7.5 | 0.2 | 0.2 |
| Kazakhstan | -8.8 | -10.1 | 23.7 |
| Kenya | 0.1 | 0.0 | 0.4 |
| Kyrgyz Republic | 0.1 | 0.0 | 0.1 |
| Lesotho | 0.4 | 0.0 | 0.2 |
| Liberia | 5.2 | 0.3 | 0.3 |
| Madagascar | 0.9 | 0.4 | 1.6 |
| Malawi | 0.4 | 0.1 | 0.2 |
| Maldives | -0.4 | -14.8 | 1.5 |
| Mali | 0.8 | 0.3 | 1.6 |
| Morocco | 0.5 | 0.0 | 0.0 |
| Mozambique | 1.0 | 0.3 | 0.5 |
| Namibia | 0.3 | 0.1 | 0.4 |
| Nepal | 1.9 | 0.1 | 0.1 |
| Nicaragua | -0.2 | -0.7 | 1.4 |
| Niger | 3.9 | 0.6 | 1.0 |
| Nigeria | -0.6 | -0.7 | 6.9 |
| Pakistan | 1.2 | 0.5 | 1.1 |
| Peru | 0.1 | 0.0 | 0.5 |
| Philippines | 0.2 | 0.1 | 0.6 |
| Rwanda | 0.3 | 0.0 | 0.1 |
| Senegal | 0.8 | 0.2 | 0.3 |
| Sierra Leone | 10.9 | 0.1 | 0.1 |
| South Africa | -0.3 | -0.8 | 0.9 |
| Tajikistan | -0.2 | -3.8 | 0.7 |
| Tanzania | 0.1 | 0.1 | 0.4 |
| Timor-Leste | 1.2 | 0.2 | 1.9 |
| Togo | 2.0 | 0.2 | 0.3 |
| Turkey | 0.6 | 0.1 | 0.3 |
| Uganda | 1.2 | 0.2 | 0.3 |
| Vietnam | 0.7 | 0.0 | 0.3 |
| Yemen | 0.5 | 0.3 | 3.3 |
| Zambia | 0.0 | 0.0 | 0.6 |
| Zimbabwe | -0.5 | -1.0 | 1.9 |

**Note:** AARC is calculated as ln((rate in latest year/rate in earliest year))/ (latest year - earliest year), with positive values denote increasing rate while negative values denote decreasing rate

Table S44: Predicted average annual rate of increase (AARC) of BCG immunization among children 12-23 months of age in low- and middle-income countries by wealth quintiles

| **Country** | **Poorest** | | | **Poorer** | | | **Middle** | | | **Richer** | | | **Richest** | | |
| --- | --- | --- | --- | --- | --- | --- | --- | --- | --- | --- | --- | --- | --- | --- | --- |
|  | **Predicted** | | **Required** | **Predicted** | | **Required** | **Predicted** | | **Required** | **Predicted** | | **Required** | **Predicted** | | **Required** |
|  | **1990-2018** | **2019-2030** | **2019-2030** | **1990-2018** | **2019-2030** | **2019-2030** | **1990-2018** | **2019-2030** | **2019-2030** | **1990-2018** | **2019-2030** | **2019-2030** | **1990-2018** | **2019-2030** | **2019-2030** |
| Albania | 33.7 | 0.0 | 0.0 | 0.0 | 0.0 | 0.0 | 0.0 | 0.0 | 0.0 | 0.0 | 0.0 | 0.0 | 0.0 | 0.0 | 0.0 |
| Armenia | 2.0 | 0.0 | 0.1 | 0.5 | 0.0 | 0.0 | 0.5 | 0.0 | 0.1 | 0.8 | 0.0 | 0.0 | 0.2 | 0.0 | 0.0 |
| Bangladesh | 1.3 | 0.1 | 0.2 | 1.0 | 0.1 | 0.1 | 1.1 | 0.0 | 0.1 | 0.4 | 0.1 | 0.1 | 1.2 | 0.0 | 0.0 |
| Benin | -0.1 | -0.1 | 2.8 | 0.1 | 0.0 | 1.4 | 0.0 | -0.1 | 0.8 | 0.0 | -0.1 | 0.5 | 0.1 | 0.0 | 0.2 |
| Bolivia | 3.2 | 0.0 | 0.0 | 3.1 | 0.0 | 0.0 | 1.4 | 0.0 | 0.0 | 1.1 | 0.0 | 0.0 | 0.0 | 0.0 | 0.0 |
| Burkina Faso | 1.9 | 0.3 | 0.6 | 1.6 | 0.2 | 0.4 | 2.5 | 0.1 | 0.1 | 2.6 | 0.0 | 0.0 | 0.3 | 0.1 | 0.1 |
| Burundi | -0.2 | -2.4 | 0.7 | 0.0 | -0.4 | 0.2 | 0.0 | -0.1 | 0.1 | -0.1 | -2.6 | 0.3 | 0.0 | -1.6 | 0.1 |
| Cambodia | 3.2 | 0.2 | 0.3 | 2.7 | 0.2 | 0.3 | 5.5 | 0.0 | 0.0 | 4.9 | 0.0 | 0.0 | 1.4 | 0.1 | 0.1 |
| Cameroon | 0.8 | 0.5 | 2.6 | 2.2 | 0.4 | 0.7 | 1.4 | 0.2 | 0.3 | 0.4 | 0.2 | 0.4 | 0.4 | 0.0 | 0.0 |
| Chad | 7.1 | 2.9 | 5.2 | 3.4 | 1.7 | 4.7 | 3.4 | 1.7 | 4.4 | 1.3 | 0.9 | 4.9 | 0.7 | 0.4 | 2.1 |
| Colombia | 0.4 | 0.1 | 0.3 | 0.2 | 0.1 | 0.1 | 0.1 | 0.0 | 0.1 | 0.1 | 0.0 | 0.1 | 0.2 | 0.0 | 0.1 |
| Comoros | -0.8 | -1.5 | 3.4 | -0.1 | -0.3 | 2.0 | -0.2 | -0.4 | 1.0 | -0.7 | -4.3 | 2.1 | -0.9 | -4.4 | 2.6 |
| Congo | 3.1 | 0.3 | 0.7 | 2.1 | 0.2 | 0.4 | 0.1 | -0.1 | 0.4 | 0.2 | 0.0 | 0.1 | -0.6 | -14.4 | 2.1 |
| Congo DR | 3.2 | 0.8 | 2.0 | 5.3 | 0.7 | 1.2 | 5.7 | 0.7 | 1.1 | 1.4 | 0.2 | 1.0 | 0.3 | 0.0 | 0.4 |
| Cote d'Ivoire | 1.4 | 0.7 | 2.5 | 0.3 | 0.0 | 2.0 | 0.6 | 0.3 | 1.3 | 0.3 | 0.1 | 0.2 | 0.0 | 0.0 | 0.2 |
| Dominican Republic | 0.4 | 0.1 | 0.9 | 0.8 | 0.1 | 0.2 | 0.3 | 0.1 | 0.3 | -0.6 | -3.8 | 1.7 | 0.4 | 0.1 | 0.4 |
| Egypt | 0.2 | 0.0 | 0.1 | 0.3 | 0.0 | 0.0 | 0.1 | 0.0 | 0.0 | 0.0 | 0.0 | 0.0 | 0.0 | -0.5 | 0.1 |
| Ethiopia | 2.5 | 1.4 | 4.1 | 3.5 | 1.2 | 2.6 | 3.3 | 1.2 | 2.6 | 3.0 | 1.0 | 2.1 | 1.7 | 0.5 | 1.2 |
| Gabon | 1.7 | 0.3 | 0.7 | 0.2 | 0.0 | 0.7 | -0.3 | -0.7 | 1.3 | -0.1 | -0.2 | 0.7 | 0.1 | 0.0 | 0.3 |
| Ghana | 1.8 | 0.2 | 0.3 | 1.1 | 0.1 | 0.2 | 0.8 | 0.1 | 0.1 | 0.8 | 0.0 | 0.0 | 0.3 | 0.0 | 0.1 |
| Guatemala | 1.8 | 0.1 | 0.1 | 1.0 | 0.1 | 0.2 | 1.5 | 0.1 | 0.1 | 1.3 | 0.0 | 0.1 | 0.3 | 0.1 | 0.2 |
| Guinea | 1.8 | 0.7 | 2.2 | 1.2 | 0.5 | 1.7 | 0.7 | 0.3 | 1.2 | 0.2 | 0.0 | 1.2 | -0.2 | -0.6 | 0.9 |
| Haiti | 1.3 | 0.7 | 2.5 | 1.4 | 0.6 | 1.8 | 0.5 | 0.3 | 1.6 | 0.8 | 0.3 | 0.8 | 0.5 | 0.2 | 0.6 |
| Honduras | 0.8 | 0.0 | 0.0 | 0.3 | 0.0 | 0.1 | 0.3 | 0.0 | 0.1 | 0.0 | -0.1 | 0.1 | 0.8 | 0.0 | 0.0 |
| India | 3.9 | 0.7 | 1.1 | 3.3 | 0.5 | 0.7 | 2.1 | 0.3 | 0.5 | 1.2 | 0.2 | 0.4 | 0.3 | 0.1 | 0.3 |
| Indonesia | 1.2 | 0.5 | 1.7 | 0.7 | 0.3 | 1.0 | 0.7 | 0.2 | 0.6 | 0.2 | 0.1 | 0.5 | -0.1 | -0.2 | 0.6 |
| Jordan | 8.0 | 0.2 | 0.3 | 7.3 | 0.1 | 0.2 | 8.1 | 0.1 | 0.1 | 7.5 | 0.1 | 0.2 | 7.5 | 0.2 | 0.3 |
| Kazakhstan | -11.8 | -8.8 | 31.4 | -1.8 | -3.0 | 5.2 | -6.4 | -7.4 | 17.5 | -13.5 | -13.1 | 36.0 | -21.5 | -14.8 | 56.8 |
| Kenya | 0.1 | -0.1 | 1.0 | 0.4 | 0.1 | 0.2 | 0.1 | 0.0 | 0.2 | 0.0 | 0.0 | 0.2 | 0.0 | 0.0 | 0.2 |
| Kyrgyz Republic | 0.5 | 0.0 | 0.0 | 0.2 | 0.0 | 0.0 | 0.1 | 0.0 | 0.1 | 0.1 | 0.0 | 0.1 | 0.0 | 0.0 | 0.1 |
| Lesotho | 1.0 | 0.1 | 0.3 | -0.4 | -4.2 | 1.3 | 0.4 | 0.1 | 0.1 | 2.0 | 0.0 | 0.1 | 0.8 | 0.0 | 0.0 |
| Liberia | 6.2 | 0.6 | 0.8 | 14.6 | 0.1 | 0.1 | 6.4 | 0.2 | 0.2 | 0.2 | -0.1 | 0.4 | 2.6 | 0.1 | 0.1 |
| Madagascar | 2.4 | 0.9 | 2.7 | 2.3 | 0.6 | 1.4 | 3.4 | 0.2 | 0.4 | 1.3 | 0.1 | 0.3 | 0.5 | 0.1 | 0.2 |
| Malawi | 0.5 | 0.1 | 0.3 | 0.4 | 0.1 | 0.3 | 0.4 | 0.1 | 0.2 | 0.3 | 0.1 | 0.2 | 0.1 | 0.1 | 0.2 |
| Maldives | -0.5 | -17.5 | 1.8 | -0.4 | -19.6 | 1.6 | -0.5 | -27.3 | 2.0 | 0.1 | 0.0 | 0.2 | -1.2 | -80.7 | 6.0 |
| Mali | 1.5 | 0.7 | 2.3 | 1.0 | 0.5 | 2.1 | 0.7 | 0.3 | 2.1 | 1.0 | 0.3 | 0.8 | 0.0 | -0.1 | 0.7 |
| Morocco | 1.2 | 0.0 | 0.0 | 0.3 | 0.0 | 0.1 | 0.3 | 0.0 | 0.0 | 0.4 | 0.0 | 0.0 | 0.1 | 0.0 | 0.1 |
| Mozambique | 2.6 | 0.5 | 0.8 | 1.3 | 0.3 | 0.6 | 1.6 | 0.2 | 0.3 | 0.1 | 0.1 | 0.3 | -0.1 | -0.5 | 0.3 |
| Namibia | 0.7 | 0.2 | 0.3 | 0.4 | 0.1 | 0.3 | 0.2 | 0.1 | 0.6 | 0.3 | 0.1 | 0.3 | 0.2 | 0.1 | 0.2 |
| Nepal | 4.1 | 0.1 | 0.1 | 1.7 | 0.1 | 0.2 | 1.2 | 0.1 | 0.2 | 1.5 | 0.0 | 0.1 | 0.7 | 0.0 | 0.0 |
| Nicaragua | -1.0 | -1.4 | 3.7 | 0.4 | -0.2 | 0.3 | 0.2 | -0.3 | 0.6 | -6.8 | -11.5 | 18.9 | 0.5 | -0.2 | 0.3 |
| Niger | 7.3 | 0.9 | 1.2 | 6.1 | 0.6 | 0.8 | 7.8 | 0.5 | 0.6 | 3.7 | 0.6 | 1.0 | 0.5 | 0.2 | 0.6 |
| Nigeria | -4.5 | -4.1 | 20.2 | 0.3 | 0.6 | 9.9 | 3.0 | 1.4 | 3.9 | 2.6 | 0.7 | 1.7 | 0.2 | -0.1 | 0.9 |
| Pakistan | 1.8 | 0.9 | 2.7 | 1.4 | 0.6 | 1.5 | 1.6 | 0.4 | 0.8 | 1.0 | 0.2 | 0.5 | 0.6 | 0.1 | 0.2 |
| Peru | 0.1 | 0.0 | 1.2 | 0.1 | 0.0 | 0.5 | 0.0 | 0.0 | 0.3 | -0.1 | -0.2 | 0.4 | 0.0 | 0.0 | 0.2 |
| Philippines | 0.3 | 0.2 | 1.3 | 0.3 | 0.1 | 0.4 | 0.0 | -0.1 | 0.5 | 0.1 | 0.0 | 0.3 | 0.0 | 0.0 | 0.2 |
| Rwanda | 0.2 | 0.1 | 0.2 | 0.5 | 0.0 | 0.1 | 0.2 | 0.0 | 0.1 | 2.0 | 0.0 | 0.0 | 0.2 | 0.0 | 0.0 |
| Senegal | -0.2 | -0.4 | 1.0 | 0.4 | 0.1 | 0.4 | 1.2 | 0.1 | 0.2 | 2.0 | 0.1 | 0.1 | 8.0 | 0.0 | 0.0 |
| Sierra Leone | 12.8 | 0.1 | 0.1 | 9.1 | 0.1 | 0.1 | 13.6 | 0.0 | 0.0 | 16.7 | 0.0 | 0.0 | 3.7 | 0.2 | 0.3 |
| South Africa | -0.4 | -1.0 | 1.3 | -0.1 | -0.4 | 0.6 | -0.2 | -0.5 | 0.8 | -0.1 | -0.3 | 0.5 | -0.6 | -3.3 | 1.8 |
| Tajikistan | -0.3 | -5.5 | 0.9 | -0.1 | -1.8 | 0.4 | -0.3 | -7.8 | 0.9 | -0.2 | -1.9 | 0.6 | -0.2 | -4.2 | 0.7 |
| Tanzania | 0.3 | 0.1 | 0.6 | -0.1 | -0.2 | 0.6 | 0.3 | 0.1 | 0.3 | 0.0 | -0.1 | 0.4 | -0.4 | -8.5 | 1.4 |
| Timor-Leste | 1.7 | 0.6 | 3.0 | 0.9 | 0.1 | 2.6 | 0.6 | -0.2 | 2.0 | 0.7 | -0.1 | 1.3 | 1.5 | 0.2 | 0.8 |
| Togo | 3.8 | 0.2 | 0.3 | 1.5 | 0.3 | 0.5 | 1.4 | 0.2 | 0.4 | 2.3 | 0.1 | 0.1 | 0.7 | 0.1 | 0.1 |
| Turkey | 2.1 | 0.2 | 0.4 | 0.3 | 0.1 | 0.5 | 0.3 | 0.1 | 0.3 | 0.4 | 0.0 | 0.1 | -0.2 | -1.9 | 0.7 |
| Uganda | 1.4 | 0.2 | 0.3 | 1.7 | 0.2 | 0.2 | 1.2 | 0.2 | 0.3 | 0.8 | 0.2 | 0.5 | 0.6 | 0.1 | 0.3 |
| Vietnam | 0.4 | -0.1 | 1.6 | 2.8 | 0.0 | 0.0 | -1.0 | -2.5 | 3.0 | 0.3 | 0.0 | 0.1 | 15.7 | 0.0 | 0.0 |
| Zambia | -0.1 | -0.2 | 0.7 | -0.4 | -1.3 | 1.4 | -0.1 | -0.3 | 0.9 | -0.3 | -1.7 | 0.8 | -1.0 | -11.8 | 3.1 |
| Zimbabwe | -0.6 | -1.4 | 2.2 | -0.5 | -1.1 | 2.3 | -0.1 | -0.3 | 1.3 | -0.5 | -1.3 | 1.7 | -0.4 | -1.0 | 1.7 |

**Note:** AARC is calculated as ln((rate in latest year/rate in earliest year))/ (latest year - earliest year), with positive values denote increasing rate while negative values denote decreasing rate

Table S45: Predicted average annual rate of increase (AARC) of BCG immunization among children 12-23 months of age in low- and middle-income countries by place of residence

| **Country** | **Rural** | | | **Urban** | | |
| --- | --- | --- | --- | --- | --- | --- |
|  | **Predicted** | | **Required** | **Predicted** | | **Required** |
|  | **1990-2018** | **2019-2030** | **2019-2030** | **1990-2018** | **2019-2030** | **2019-2030** |
| Albania | 18.5 | 0.0 | 0.0 | 10.7 | 0.0 | 0.0 |
| Armenia | 0.9 | 0.0 | 0.1 | 0.6 | 0.0 | 0.0 |
| Bangladesh | 0.9 | 0.1 | 0.1 | 0.6 | 0.0 | 0.1 |
| Benin | 0.0 | -0.1 | 1.4 | 0.2 | 0.1 | 0.6 |
| Bolivia | 3.0 | 0.0 | 0.0 | 1.1 | 0.0 | 0.0 |
| Brazil | 1.9 | 0.0 | 0.1 | 1.1 | 0.0 | 0.0 |
| Burkina Faso | 1.8 | 0.2 | 0.3 | 0.3 | 0.0 | 0.1 |
| Burundi | -0.1 | -0.9 | 0.3 | 0.0 | -3.6 | 0.2 |
| Cambodia | 3.1 | 0.1 | 0.2 | 3.5 | 0.1 | 0.1 |
| Cameroon | 1.1 | 0.4 | 1.3 | 0.5 | 0.1 | 0.3 |
| Chad | 3.7 | 1.8 | 4.6 | 0.5 | 0.3 | 2.5 |
| Colombia | 0.2 | 0.1 | 0.3 | 0.2 | 0.1 | 0.1 |
| Comoros | -0.5 | -1.1 | 2.1 | -0.2 | -0.4 | 1.5 |
| Congo | 1.4 | 0.2 | 0.8 | 0.0 | -0.2 | 0.3 |
| Congo DR | 4.4 | 0.7 | 1.4 | 2.2 | 0.2 | 0.5 |
| Cote d'Ivoire | 0.6 | 0.3 | 2.1 | 0.1 | 0.1 | 0.5 |
| Dominican Republic | 1.2 | 0.1 | 0.2 | 0.6 | 0.1 | 0.4 |
| Egypt | 0.4 | 0.0 | 0.0 | 0.1 | 0.0 | 0.0 |
| Ethiopia | 2.8 | 1.2 | 2.9 | 1.7 | 0.4 | 0.9 |
| Gabon | 0.8 | 0.3 | 1.0 | 0.0 | -0.1 | 0.8 |
| Ghana | 1.2 | 0.1 | 0.2 | 0.3 | 0.1 | 0.2 |
| Guatemala | 1.4 | 0.1 | 0.1 | 0.9 | 0.1 | 0.1 |
| Guinea | 0.9 | 0.4 | 1.7 | 0.2 | 0.1 | 0.6 |
| Haiti | 1.0 | 0.5 | 1.9 | 0.3 | 0.2 | 1.1 |
| Honduras | 0.9 | 0.0 | 0.0 | 0.1 | -0.1 | 0.1 |
| India | 2.5 | 0.4 | 0.7 | 0.9 | 0.2 | 0.6 |
| Indonesia | 1.0 | 0.4 | 1.0 | 0.3 | 0.1 | 0.6 |
| Jordan | 11.9 | 0.2 | 0.2 | 6.6 | 0.2 | 0.2 |
| Kazakhstan | -3.0 | -4.5 | 8.4 | -100.6 | -131.1 | 268.7 |
| Kenya | 0.1 | 0.0 | 0.4 | 0.1 | 0.0 | 0.2 |
| Kyrgyz Republic | 0.1 | 0.0 | 0.1 | 0.1 | 0.0 | 0.0 |
| Lesotho | 0.3 | 0.0 | 0.2 | 0.5 | 0.1 | 0.1 |
| Liberia | 5.8 | 0.4 | 0.5 | 1.2 | 0.1 | 0.3 |
| Madagascar | 1.0 | 0.4 | 1.8 | 0.9 | 0.2 | 0.3 |
| Malawi | 0.4 | 0.1 | 0.2 | 0.2 | 0.1 | 0.1 |
| Maldives | -0.5 | -12.7 | 1.5 | -0.5 | -66.0 | 2.9 |
| Mali | 1.1 | 0.5 | 1.8 | 0.3 | 0.1 | 0.5 |
| Morocco | 0.7 | 0.0 | 0.0 | 0.2 | 0.0 | 0.0 |
| Mozambique | 1.5 | 0.3 | 0.6 | -0.1 | -0.4 | 0.6 |
| Namibia | 0.6 | 0.1 | 0.3 | 0.1 | 0.0 | 0.5 |
| Nepal | 1.7 | 0.1 | 0.2 | 1.0 | 0.1 | 0.1 |
| Nicaragua | -1.0 | -1.5 | 3.3 | 0.7 | -0.1 | 0.1 |
| Niger | 5.2 | 0.7 | 1.0 | 0.5 | 0.2 | 0.4 |
| Nigeria | -1.4 | -1.6 | 10.1 | -0.2 | -0.3 | 3.1 |
| Pakistan | 1.4 | 0.6 | 1.4 | 0.6 | 0.2 | 0.5 |
| Peru | 0.2 | 0.1 | 0.7 | 0.0 | 0.0 | 0.4 |
| Philippines | 0.3 | 0.1 | 0.7 | 0.1 | 0.0 | 0.5 |
| Rwanda | 0.3 | 0.0 | 0.1 | 0.2 | 0.0 | 0.0 |
| Senegal | 0.9 | 0.2 | 0.4 | 0.6 | 0.1 | 0.1 |
| Sierra Leone | 13.4 | 0.1 | 0.1 | 3.5 | 0.1 | 0.2 |
| South Africa | -0.2 | -0.4 | 0.8 | -0.3 | -1.4 | 1.1 |
| Tajikistan | -0.2 | -2.9 | 0.6 | -0.3 | -9.5 | 1.2 |
| Tanzania | 0.1 | 0.1 | 0.5 | -0.1 | -0.9 | 0.3 |
| Timor-Leste | 1.3 | 0.3 | 2.1 | 0.8 | 0.0 | 1.2 |
| Togo | 2.2 | 0.2 | 0.4 | 1.1 | 0.1 | 0.1 |
| Turkey | 1.2 | 0.2 | 0.3 | 0.3 | 0.1 | 0.3 |
| Uganda | 1.3 | 0.2 | 0.3 | 0.3 | 0.1 | 0.3 |
| Vietnam | 0.7 | 0.0 | 0.5 | 0.9 | 0.0 | 0.0 |
| Yemen | 0.4 | 0.3 | 4.3 | -0.1 | -0.1 | 1.5 |
| Zambia | 0.1 | 0.0 | 0.6 | -0.1 | -0.2 | 0.4 |
| Zimbabwe | -0.4 | -0.9 | 2.0 | -0.4 | -1.1 | 1.6 |

**Note:** AARC is calculated as ln((rate in latest year/rate in earliest year))/ (latest year - earliest year), with positive values denote increasing rate while negative values denote decreasing rate

Table S46: Predicted average annual rate of increase (AARC) of BCG immunization among children 12-23 months of age in low- and middle-income countries by education of mother

| **Country** | **Below secondary education** | | | **Secondary+ education** | | |
| --- | --- | --- | --- | --- | --- | --- |
|  | **Predicted** | | **Required** | **Predicted** | | **Required** |
|  | **1990-2018** | **2019-2030** | **2019-2030** | **1990-2018** | **2019-2030** | **2019-2030** |
| Albania | 20.4 | 0.0 | 0.0 | 0.0 | 0.0 | 0.0 |
| Armenia | 0.0 | 0.0 | 0.0 | 0.7 | 0.0 | 0.0 |
| Bangladesh | 0.9 | 0.1 | 0.2 | 0.3 | 0.0 | 0.0 |
| Benin | 0.0 | 0.0 | 1.3 | 0.0 | -0.1 | 0.3 |
| Bolivia | 2.7 | 0.0 | 0.0 | 1.1 | 0.0 | 0.0 |
| Brazil | 1.7 | 0.0 | 0.0 | 0.0 | -0.2 | 0.6 |
| Burkina Faso | 1.6 | 0.2 | 0.3 | -1.1 | -15.8 | 3.5 |
| Burundi | -0.1 | -0.9 | 0.3 | -0.2 | -48.1 | 0.8 |
| Cambodia | 3.1 | 0.2 | 0.2 | 1.2 | 0.1 | 0.1 |
| Cameroon | 1.0 | 0.4 | 1.3 | 0.5 | 0.1 | 0.1 |
| Chad | 2.6 | 1.5 | 4.8 | -0.6 | -1.1 | 2.5 |
| Colombia | 0.2 | 0.1 | 0.3 | 0.2 | 0.0 | 0.1 |
| Comoros | -0.4 | -0.8 | 2.0 | -3.7 | -40.1 | 12.2 |
| Congo | 1.1 | 0.1 | 0.8 | 0.9 | 0.1 | 0.2 |
| Congo DR | 4.2 | 0.8 | 1.4 | 1.4 | 0.2 | 0.7 |
| Cote d'Ivoire | 0.5 | 0.2 | 1.5 | -0.2 | -1.4 | 0.5 |
| Dominican Republic | 0.7 | 0.2 | 0.6 | 0.7 | 0.1 | 0.1 |
| Egypt | 0.4 | 0.0 | 0.0 | 0.1 | 0.0 | 0.0 |
| Ethiopia | 2.7 | 1.2 | 2.8 | 1.5 | 0.1 | 0.2 |
| Gabon | 0.4 | 0.1 | 1.1 | 0.1 | -0.1 | 0.6 |
| Ghana | 0.9 | 0.2 | 0.3 | -0.5 | -4.9 | 1.6 |
| Guatemala | 1.4 | 0.1 | 0.1 | 0.3 | 0.1 | 0.2 |
| Guinea | 0.6 | 0.3 | 1.7 | -0.2 | -0.7 | 0.7 |
| Haiti | 0.7 | 0.4 | 2.2 | -0.1 | -0.3 | 1.2 |
| Honduras | 0.4 | 0.0 | 0.1 | 0.5 | 0.0 | 0.0 |
| India | 2.6 | 0.6 | 1.0 | 0.3 | 0.1 | 0.4 |
| Indonesia | 0.8 | 0.4 | 1.6 | 0.1 | 0.0 | 0.6 |
| Jordan | 7.4 | 0.6 | 0.7 | 7.5 | 0.1 | 0.2 |
| Kazakhstan | N/A | N/A | NA | -8.6 | -9.5 | 23.0 |
| Kenya | 0.1 | 0.0 | 0.5 | 0.1 | 0.0 | 0.2 |
| Kyrgyz Republic | 0.0 | 0.0 | 0.0 | 0.1 | 0.0 | 0.1 |
| Lesotho | 0.2 | 0.0 | 0.4 | 0.3 | 0.0 | 0.1 |
| Liberia | 5.7 | 0.3 | 0.4 | 1.8 | 0.1 | 0.2 |
| Madagascar | 0.9 | 0.4 | 2.1 | 0.9 | 0.1 | 0.1 |
| Malawi | 0.3 | 0.1 | 0.3 | 0.1 | 0.0 | 0.1 |
| Maldives | -0.5 | -21.4 | 1.9 | -0.4 | -12.1 | 1.4 |
| Mali | 0.8 | 0.4 | 1.8 | -0.3 | -1.0 | 0.9 |
| Morocco | 0.6 | 0.0 | 0.0 | -2.0 | -23.8 | 6.4 |
| Mozambique | 1.1 | 0.3 | 0.5 | -0.2 | -1.9 | 0.6 |
| Namibia | 0.4 | 0.2 | 0.5 | 0.2 | 0.1 | 0.3 |
| Nepal | 1.9 | 0.1 | 0.2 | 0.0 | 0.0 | 0.1 |
| Nicaragua | -0.1 | -0.6 | 1.5 | -0.6 | -1.5 | 1.8 |
| Niger | 4.0 | 0.7 | 1.1 | 0.5 | 0.1 | 0.1 |
| Nigeria | -2.3 | -2.7 | 12.4 | -0.2 | -0.3 | 1.6 |
| Pakistan | 1.1 | 0.5 | 1.7 | 0.5 | 0.1 | 0.2 |
| Peru | 0.1 | 0.0 | 0.9 | -0.1 | -0.2 | 0.4 |
| Philippines | 0.0 | 0.0 | 1.6 | 0.1 | 0.0 | 0.4 |
| Rwanda | 0.3 | 0.0 | 0.1 | 0.5 | 0.0 | 0.0 |
| Senegal | 0.8 | 0.2 | 0.4 | 0.1 | 0.0 | 0.0 |
| Sierra Leone | 10.6 | 0.1 | 0.1 | 5.2 | 0.0 | 0.1 |
| South Africa | -0.2 | -0.3 | 0.9 | -0.3 | -1.3 | 1.0 |
| Tajikistan | -0.2 | -2.1 | 0.7 | -0.2 | -3.9 | 0.7 |
| Tanzania | 0.1 | 0.1 | 0.4 | -0.5 | -12.1 | 1.6 |
| Timor-Leste | 0.7 | 0.0 | 2.6 | 0.5 | -0.2 | 1.6 |
| Togo | 2.0 | 0.2 | 0.3 | 0.2 | 0.0 | 0.1 |
| Turkey | 0.7 | 0.2 | 0.5 | -0.1 | -0.3 | 0.4 |
| Uganda | 1.2 | 0.2 | 0.3 | 0.2 | 0.1 | 0.3 |
| Vietnam | 0.1 | -0.4 | 1.9 | 1.2 | 0.0 | 0.0 |
| Yemen | 0.3 | 0.2 | 3.9 | -0.6 | -2.2 | 1.9 |
| Zambia | 0.0 | 0.0 | 0.6 | -0.4 | -2.6 | 1.1 |
| Zimbabwe | -0.6 | -1.1 | 2.3 | -0.6 | -1.7 | 2.0 |

**Note:** AARC is calculated as ln((rate in latest year/rate in earliest year))/ (latest year - earliest year), with positive values denote increasing rate while negative values denote decreasing rate

Table S47: Predicted average annual rate of increase (AARC) of BCG immunization among children 12-23 months of age in low- and middle-income countries by age of mother

| **Country** | **Adolescent mother** | | | **Adult mother** | | |
| --- | --- | --- | --- | --- | --- | --- |
|  | **Predicted** | | **Required** | **Predicted** | | **Required** |
|  | **1990-2018** | **2019-2030** | **2019-2030** | **1990-2018** | **2019-2030** | **2019-2030** |
| Albania | 39.9 | 0.0 | 0.0 | 13.3 | 0.0 | 0.0 |
| Armenia | 3.3 | 0.0 | 0.0 | 0.7 | 0.0 | 0.0 |
| Bangladesh | 1.1 | 0.1 | 0.1 | 0.9 | 0.1 | 0.1 |
| Benin | -0.1 | -0.2 | 1.5 | 0.1 | 0.0 | 1.1 |
| Bolivia | 2.6 | 0.0 | 0.0 | 1.9 | 0.0 | 0.0 |
| Brazil | 1.2 | 0.0 | 0.0 | 1.6 | 0.0 | 0.0 |
| Burkina Faso | 1.7 | 0.3 | 0.5 | 1.6 | 0.1 | 0.2 |
| Burundi | 37.2 | 0.0 | 0.0 | -0.1 | -1.0 | 0.3 |
| Cambodia | 8.1 | 0.0 | 0.0 | 3.0 | 0.1 | 0.2 |
| Cameroon | 0.9 | 0.3 | 1.0 | 0.9 | 0.3 | 0.8 |
| Chad | 1.3 | 0.9 | 5.4 | 2.8 | 1.5 | 4.2 |
| Colombia | 0.0 | 0.0 | 0.4 | 0.3 | 0.1 | 0.1 |
| Comoros | -0.5 | -1.0 | 1.9 | -0.4 | -0.8 | 1.9 |
| Congo | -0.7 | -7.8 | 2.3 | 1.3 | 0.1 | 0.4 |
| Congo DR | 6.8 | 0.4 | 0.6 | 3.0 | 0.6 | 1.2 |
| Cote d'Ivoire | 0.1 | 0.0 | 2.4 | 0.5 | 0.2 | 1.3 |
| Dominican Republic | 1.4 | 0.1 | 0.1 | 0.7 | 0.2 | 0.3 |
| Egypt | 0.1 | 0.0 | 0.1 | 0.3 | 0.0 | 0.0 |
| Ethiopia | 1.4 | 0.8 | 3.7 | 2.7 | 1.1 | 2.6 |
| Gabon | -1.4 | -4.3 | 4.3 | 0.6 | 0.1 | 0.4 |
| Ghana | 1.1 | 0.1 | 0.2 | 0.9 | 0.1 | 0.2 |
| Guatemala | 2.0 | 0.1 | 0.1 | 1.2 | 0.1 | 0.1 |
| Guinea | 0.4 | 0.1 | 1.4 | 0.7 | 0.3 | 1.5 |
| Haiti | 0.8 | 0.4 | 1.4 | 0.8 | 0.4 | 1.6 |
| Honduras | 23.9 | 0.0 | 0.0 | 0.3 | 0.0 | 0.1 |
| India | 2.2 | 0.4 | 0.6 | 2.1 | 0.4 | 0.7 |
| Indonesia | 1.0 | 0.4 | 1.1 | 0.8 | 0.3 | 0.8 |
| Jordan | 7.7 | 0.0 | 0.0 | 7.5 | 0.2 | 0.2 |
| Kazakhstan | -14.4 | -8.9 | 38.1 | -8.5 | -9.1 | 22.8 |
| Kenya | 0.2 | 0.1 | 0.3 | 0.1 | 0.0 | 0.4 |
| Kyrgyz Republic | 0.0 | 0.0 | 0.0 | 0.1 | 0.0 | 0.1 |
| Lesotho | 0.0 | 0.0 | 0.0 | 0.4 | 0.1 | 0.2 |
| Liberia | 8.4 | 0.1 | 0.1 | 5.0 | 0.3 | 0.4 |
| Madagascar | 0.9 | 0.4 | 2.3 | 0.9 | 0.4 | 1.5 |
| Malawi | 0.4 | 0.1 | 0.2 | 0.3 | 0.1 | 0.2 |
| Maldives | -4.9 | -110.6 | 21.0 | -0.4 | -14.1 | 1.4 |
| Mali | 0.1 | -0.1 | 2.2 | 0.9 | 0.4 | 1.5 |
| Morocco | 0.9 | 0.0 | 0.0 | 0.5 | 0.0 | 0.0 |
| Mozambique | 0.6 | 0.2 | 0.5 | 1.1 | 0.3 | 0.5 |
| Namibia | 0.3 | 0.0 | 0.1 | 0.3 | 0.1 | 0.4 |
| Nepal | 1.8 | 0.1 | 0.1 | 1.9 | 0.1 | 0.1 |
| Nicaragua | 0.7 | -0.1 | 0.4 | -0.4 | -0.9 | 1.8 |
| Niger | 4.5 | 0.6 | 0.9 | 3.9 | 0.6 | 1.0 |
| Nigeria | -1.8 | -1.8 | 12.4 | -0.5 | -0.6 | 6.6 |
| Pakistan | 1.7 | 0.8 | 2.1 | 1.1 | 0.4 | 1.1 |
| Peru | 0.3 | 0.1 | 0.4 | 0.1 | 0.0 | 0.5 |
| Philippines | 0.1 | 0.1 | 0.7 | 0.2 | 0.1 | 0.6 |
| Rwanda | 2.1 | 0.0 | 0.0 | 0.3 | 0.0 | 0.1 |
| Senegal | 0.8 | 0.3 | 0.6 | 0.8 | 0.2 | 0.3 |
| Sierra Leone | 16.2 | 0.0 | 0.0 | 10.3 | 0.1 | 0.1 |
| South Africa | -0.2 | -0.4 | 1.1 | -0.3 | -0.8 | 0.9 |
| Tajikistan | -0.9 | -116.6 | 6.2 | -0.2 | -3.6 | 0.7 |
| Tanzania | 0.1 | 0.0 | 0.4 | 0.1 | 0.1 | 0.4 |
| Timor-Leste | -0.7 | -1.9 | 3.9 | 1.3 | 0.2 | 1.8 |
| Togo | 1.8 | 0.1 | 0.2 | 2.1 | 0.2 | 0.3 |
| Turkey | 1.0 | 0.0 | 0.0 | 0.6 | 0.1 | 0.3 |
| Uganda | 0.8 | 0.1 | 0.2 | 1.2 | 0.2 | 0.3 |
| Vietnam | -78.3 | -127.4 | 211.7 | 0.8 | 0.0 | 0.3 |
| Yemen | 0.5 | 0.3 | 2.7 | 0.4 | 0.3 | 3.5 |
| Zambia | 0.1 | 0.0 | 0.3 | 0.0 | -0.1 | 0.6 |
| Zimbabwe | -0.1 | -0.2 | 1.5 | -0.5 | -1.1 | 1.9 |

**Note:** AARC is calculated as ln((rate in latest year/rate in earliest year))/ (latest year - earliest year), with positive values denote increasing rate while negative values denote decreasing rate

Table S48: Predicted average annual rate of increase (AARC) of BCG immunization among children 12-23 months of age in low- and middle-income countries by child sex

| **Country** | **Male** | | | **Female** | | |
| --- | --- | --- | --- | --- | --- | --- |
|  | **Predicted** | | **Required** | **Predicted** | | **Required** |
|  | **1990-2018** | **2019-2030** | **2019-2030** | **1990-2018** | **2019-2030** | **2019-2030** |
| Albania | 23.4 | 0.0 | 0.0 | 0.0 | 0.0 | 0.0 |
| Armenia | 0.8 | 0.0 | 0.0 | 0.8 | 0.0 | 0.0 |
| Bangladesh | 0.8 | 0.1 | 0.1 | 1.1 | 0.1 | 0.1 |
| Benin | 0.1 | 0.1 | 1.1 | 0.0 | 0.0 | 1.1 |
| Bolivia | 1.7 | 0.0 | 0.0 | 2.3 | 0.0 | 0.0 |
| Brazil | 1.6 | 0.0 | 0.0 | 1.5 | 0.0 | 0.0 |
| Burkina Faso | 1.8 | 0.1 | 0.2 | 1.4 | 0.2 | 0.3 |
| Burundi | -0.1 | -1.2 | 0.3 | -0.1 | -0.8 | 0.3 |
| Cambodia | 3.1 | 0.1 | 0.1 | 3.1 | 0.1 | 0.2 |
| Cameroon | 0.9 | 0.3 | 0.9 | 1.0 | 0.3 | 0.8 |
| Chad | 2.5 | 1.4 | 4.4 | 2.8 | 1.5 | 4.2 |
| Colombia | 0.2 | 0.1 | 0.2 | 0.3 | 0.1 | 0.2 |
| Comoros | -0.6 | -1.2 | 2.3 | -0.3 | -0.5 | 1.4 |
| Congo | 1.8 | 0.1 | 0.3 | 0.6 | 0.0 | 0.5 |
| Congo DR | 2.7 | 0.5 | 1.2 | 3.7 | 0.6 | 1.1 |
| Cote d'Ivoire | 0.5 | 0.3 | 1.3 | 0.4 | 0.1 | 1.5 |
| Dominican Republic | 0.8 | 0.2 | 0.3 | 0.8 | 0.2 | 0.3 |
| Egypt | 0.3 | 0.0 | 0.0 | 0.3 | 0.0 | 0.0 |
| Ethiopia | 2.2 | 1.0 | 2.7 | 3.3 | 1.2 | 2.5 |
| Gabon | 0.3 | 0.0 | 0.8 | 0.3 | 0.0 | 0.7 |
| Ghana | 0.9 | 0.1 | 0.2 | 1.0 | 0.1 | 0.2 |
| Guatemala | 1.0 | 0.1 | 0.1 | 1.5 | 0.1 | 0.1 |
| Guinea | 0.8 | 0.3 | 1.4 | 0.5 | 0.3 | 1.6 |
| Haiti | 0.7 | 0.4 | 1.8 | 0.9 | 0.4 | 1.4 |
| Honduras | 0.0 | -0.1 | 0.1 | 1.7 | 0.0 | 0.0 |
| India | 2.0 | 0.4 | 0.6 | 2.2 | 0.4 | 0.7 |
| Indonesia | 0.8 | 0.3 | 0.7 | 0.8 | 0.3 | 0.8 |
| Jordan | 7.4 | 0.2 | 0.2 | 7.7 | 0.1 | 0.1 |
| Kazakhstan | -16.7 | -12.4 | 44.3 | -4.6 | -6.1 | 12.5 |
| Kenya | 0.1 | 0.0 | 0.4 | 0.1 | 0.0 | 0.4 |
| Kyrgyz Republic | 0.1 | 0.0 | 0.0 | 0.1 | 0.0 | 0.1 |
| Lesotho | 1.7 | 0.0 | 0.1 | -0.1 | -0.4 | 0.5 |
| Liberia | 5.6 | 0.3 | 0.4 | 4.6 | 0.2 | 0.3 |
| Madagascar | 0.4 | 0.2 | 2.6 | 1.3 | 0.4 | 0.9 |
| Malawi | 0.3 | 0.1 | 0.2 | 0.4 | 0.1 | 0.2 |
| Maldives | -0.6 | -39.6 | 2.5 | -0.4 | -7.6 | 1.2 |
| Mali | 0.7 | 0.3 | 1.5 | 0.8 | 0.4 | 1.7 |
| Morocco | 0.4 | 0.0 | 0.0 | 0.6 | 0.0 | 0.0 |
| Mozambique | 0.7 | 0.2 | 0.6 | 1.4 | 0.3 | 0.5 |
| Namibia | 0.4 | 0.1 | 0.3 | 0.3 | 0.1 | 0.5 |
| Nepal | 2.1 | 0.1 | 0.1 | 1.7 | 0.1 | 0.2 |
| Nicaragua | -0.6 | -1.2 | 2.2 | 0.1 | -0.4 | 1.0 |
| Niger | 4.1 | 0.6 | 0.9 | 3.8 | 0.7 | 1.1 |
| Nigeria | -0.4 | -0.4 | 6.7 | -0.8 | -0.9 | 7.1 |
| Pakistan | 1.1 | 0.4 | 1.0 | 1.2 | 0.5 | 1.2 |
| Peru | 0.1 | 0.0 | 0.5 | 0.1 | 0.0 | 0.5 |
| Philippines | 0.2 | 0.1 | 0.6 | 0.1 | 0.1 | 0.6 |
| Rwanda | 0.4 | 0.0 | 0.1 | 0.2 | 0.0 | 0.1 |
| Senegal | 0.8 | 0.2 | 0.3 | 0.7 | 0.2 | 0.3 |
| Sierra Leone | 7.8 | 0.1 | 0.1 | 13.8 | 0.1 | 0.1 |
| South Africa | -0.1 | -0.4 | 0.6 | -0.4 | -1.3 | 1.3 |
| Tajikistan | -0.2 | -3.7 | 0.7 | -0.2 | -3.9 | 0.7 |
| Tanzania | 0.1 | 0.1 | 0.3 | 0.1 | 0.0 | 0.5 |
| Timor-Leste | 1.0 | 0.1 | 1.9 | 1.5 | 0.3 | 1.8 |
| Togo | 1.7 | 0.2 | 0.3 | 2.4 | 0.2 | 0.2 |
| Turkey | 0.7 | 0.1 | 0.2 | 0.5 | 0.2 | 0.4 |
| Uganda | 1.1 | 0.2 | 0.3 | 1.3 | 0.2 | 0.3 |
| Vietnam | 1.4 | 0.0 | 0.1 | 0.0 | -0.4 | 1.1 |
| Yemen | 0.3 | 0.2 | 3.6 | 0.7 | 0.4 | 3.1 |
| Zambia | 0.0 | 0.0 | 0.6 | 0.0 | 0.0 | 0.5 |
| Zimbabwe | -0.5 | -1.0 | 2.0 | -0.4 | -1.0 | 1.7 |

**Note:** AARC is calculated as ln((rate in latest year/rate in earliest year))/ (latest year - earliest year), with positive values denote increasing rate while negative values denote decreasing rate

Table S49: Predicted average annual rate of increase (AARC) of three doses of DPT immunization among children 12-23 months of age in low- and middle-income countries

| **Country** | **Predicted** | | **Required** |
| --- | --- | --- | --- |
|  | **1990-2018** | **2019-2030** | **2019-2030** |
| Armenia | 0.8 | 0.2 | 0.7 |
| Bangladesh | 2.1 | 0.2 | 0.4 |
| Benin | 0.1 | 0.0 | 3.4 |
| Bolivia | 4.6 | 0.3 | 0.3 |
| Brazil | 2.0 | 0.0 | 0.2 |
| Burkina Faso | 5.5 | 0.3 | 0.4 |
| Burundi | 0.6 | 0.0 | 0.3 |
| Cambodia | 4.1 | 0.5 | 0.7 |
| Cameroon | 2.1 | 0.9 | 2.3 |
| Chad | 3.2 | 2.4 | 9.2 |
| Colombia | 0.6 | 0.3 | 1.1 |
| Comoros | 0.4 | 0.2 | 2.7 |
| Congo | -2.2 | -3.6 | 7.6 |
| Congo DR | 4.3 | 1.5 | 3.3 |
| Cote d'Ivoire | 1.4 | 0.7 | 3.2 |
| Dominican Republic | 1.5 | 0.5 | 1.4 |
| Egypt | 1.1 | 0.1 | 0.1 |
| Ethiopia | 5.8 | 2.6 | 5.0 |
| Gabon | -3.8 | -3.7 | 16.2 |
| Ghana | 1.8 | 0.4 | 0.6 |
| Guatemala | 1.7 | 0.5 | 1.2 |
| Guinea | 0.4 | 0.3 | 6.0 |
| Haiti | 1.7 | 1.1 | 4.4 |
| Honduras | 0.9 | 0.1 | 0.3 |
| India | 2.3 | 0.9 | 2.2 |
| Indonesia | 1.3 | 0.7 | 2.4 |
| Jordan | 0.0 | 0.0 | 0.3 |
| Kazakhstan | 11.2 | 0.0 | 0.0 |
| Kenya | 0.6 | 0.3 | 1.1 |
| Kyrgyz Republic | 0.3 | 0.1 | 1.4 |
| Lesotho | 0.4 | 0.1 | 1.4 |
| Liberia | 6.9 | 1.0 | 1.6 |
| Madagascar | 2.1 | 0.8 | 2.0 |
| Malawi | 0.8 | 0.2 | 0.6 |
| Maldives | -0.8 | -13.2 | 2.5 |
| Mali | 3.4 | 1.1 | 2.5 |
| Morocco | 1.3 | 0.0 | 0.1 |
| Mozambique | 1.7 | 0.6 | 1.6 |
| Namibia | 1.0 | 0.4 | 1.1 |
| Nepal | 2.4 | 0.4 | 0.6 |
| Nicaragua | 0.9 | -0.2 | 1.6 |
| Niger | 6.1 | 1.5 | 2.3 |
| Nigeria | 1.0 | 1.0 | 9.2 |
| Pakistan | 2.4 | 1.1 | 2.8 |
| Peru | 0.5 | 0.3 | 1.8 |
| Philippines | 0.2 | 0.1 | 1.6 |
| Rwanda | 1.0 | 0.1 | 0.2 |
| Senegal | 2.3 | 0.5 | 0.7 |
| Sierra Leone | 6.9 | 0.7 | 1.0 |
| South Africa | -0.9 | -1.4 | 4.5 |
| Tajikistan | -0.1 | -1.1 | 1.5 |
| Tanzania | 0.6 | 0.2 | 1.6 |
| Timor-Leste | -0.6 | -1.3 | 4.7 |
| Togo | 4.8 | 0.7 | 1.0 |
| Turkey | 1.5 | 0.4 | 1.0 |
| Uganda | 2.3 | 1.1 | 3.3 |
| Vietnam | 1.3 | 0.1 | 2.0 |
| Yemen | 1.0 | 0.7 | 4.3 |
| Zambia | -0.5 | -0.7 | 4.0 |
| Zimbabwe | -0.3 | -0.5 | 2.8 |

**Note:** AARC is calculated as ln((rate in latest year/rate in earliest year))/ (latest year - earliest year), with positive values denote increasing rate while negative values denote decreasing rate

Table S50: Predicted average annual rate of increase (AARC) of three doses of DPT immunization among children 12-23 months of age in low- and middle-income countries by wealth quintiles

| **Country** | **Poorest** | | | **Poorer** | | | **Middle** | | | **Richer** | | | | **Richest** | | |
| --- | --- | --- | --- | --- | --- | --- | --- | --- | --- | --- | --- | --- | --- | --- | --- | --- |
|  | **Predicted** | | **Required** | **Predicted** | | **Required** | **Predicted** | | **Required** | **Predicted** | | **Required** | | **Predicted** | | **Required** |
|  | **1990-2018** | **2019-2030** | **2019-2030** | **1990-2018** | **2019-2030** | **2019-2030** | **1990-2018** | **2019-2030** | **2019-2030** | **1990-2018** | **2019-2030** | | **2019-2030** | **1990-2018** | **2019-2030** | **2019-2030** |
| Armenia | 2.0 | 0.2 | 0.3 | 0.3 | 0.0 | 0.9 | 0.7 | 0.2 | 0.7 | -0.5 | -1.2 | | 2.0 | 2.0 | 0.2 | 0.4 |
| Bangladesh | 2.4 | 0.4 | 0.7 | 2.7 | 0.3 | 0.4 | 2.0 | 0.2 | 0.3 | 1.9 | 0.2 | | 0.2 | 1.4 | 0.1 | 0.1 |
| Benin | 0.3 | 0.3 | 5.3 | 0.1 | 0.1 | 4.0 | 0.1 | 0.0 | 3.1 | 0.0 | -0.1 | | 2.8 | -0.3 | -0.6 | 2.0 |
| Bolivia | 6.7 | 0.2 | 0.3 | 5.6 | 0.2 | 0.3 | 5.4 | 0.2 | 0.3 | 3.0 | 0.3 | | 0.4 | 1.5 | 0.3 | 0.7 |
| Burkina Faso | 7.8 | 0.4 | 0.5 | 6.1 | 0.4 | 0.5 | 7.0 | 0.2 | 0.2 | 5.2 | 0.2 | | 0.3 | 2.6 | 0.2 | 0.4 |
| Burundi | 0.7 | 0.1 | 0.4 | 1.2 | 0.1 | 0.2 | 0.7 | 0.1 | 0.2 | 0.1 | -0.2 | | 0.3 | 0.4 | 0.0 | 0.5 |
| Cambodia | 4.2 | 0.9 | 1.6 | 4.0 | 0.6 | 1.0 | 3.6 | 0.4 | 0.7 | 4.9 | 0.2 | | 0.2 | 3.4 | 0.1 | 0.2 |
| Cameroon | 1.7 | 1.2 | 5.5 | 3.7 | 1.1 | 2.0 | 3.3 | 0.8 | 1.4 | 1.6 | 0.6 | | 1.5 | 1.1 | 0.4 | 0.9 |
| Chad | 7.4 | 5.0 | 11.0 | 5.2 | 3.5 | 9.1 | 6.5 | 3.9 | 8.3 | 1.6 | 1.5 | | 9.8 | 0.2 | 0.2 | 7.3 |
| Colombia | 1.0 | 0.4 | 1.6 | 0.8 | 0.3 | 0.9 | 0.6 | 0.2 | 0.7 | 0.1 | 0.0 | | 1.1 | 0.2 | 0.1 | 0.9 |
| Comoros | -0.1 | -0.2 | 5.2 | 0.5 | 0.3 | 3.6 | 1.1 | 0.5 | 1.6 | 0.6 | 0.3 | | 1.6 | -0.8 | -1.9 | 2.7 |
| Congo | 0.1 | 0.3 | 7.6 | -1.4 | -1.9 | 6.8 | -2.8 | -5.9 | 8.5 | -2.1 | -5.3 | | 6.4 | -4.5 | -17.9 | 13.1 |
| Congo DR | 7.5 | 2.6 | 4.3 | 4.7 | 2.1 | 4.6 | 3.6 | 1.6 | 4.0 | 4.7 | 1.2 | | 2.2 | 2.6 | 0.5 | 1.3 |
| Cote d'Ivoire | 3.1 | 1.6 | 4.7 | 1.3 | 0.8 | 4.7 | 2.3 | 1.0 | 2.8 | 0.7 | 0.4 | | 2.0 | 0.4 | 0.1 | 1.1 |
| Dominican Republic | 2.7 | 0.7 | 1.5 | 3.2 | 0.5 | 0.7 | 1.1 | 0.4 | 1.6 | -0.3 | -0.5 | | 2.7 | 3.5 | 0.3 | 0.4 |
| Egypt | 1.1 | 0.1 | 0.2 | 1.0 | 0.1 | 0.2 | 0.7 | 0.1 | 0.1 | 0.5 | 0.1 | | 0.1 | 0.6 | 0.0 | 0.1 |
| Ethiopia | 4.7 | 3.2 | 8.4 | 8.7 | 3.1 | 4.9 | 6.7 | 2.8 | 5.1 | 6.4 | 2.2 | | 3.8 | 4.8 | 1.2 | 2.0 |
| Gabon | 0.6 | 1.3 | 14.0 | -2.5 | -2.1 | 14.8 | -7.3 | -9.3 | 22.7 | -1.8 | -1.6 | | 11.3 | -6.0 | -7.9 | 19.0 |
| Ghana | 3.4 | 0.4 | 0.6 | 1.8 | 0.4 | 0.8 | 1.8 | 0.4 | 0.7 | 1.5 | 0.2 | | 0.4 | 0.4 | 0.2 | 0.6 |
| Guatemala | 1.7 | 0.7 | 1.9 | 1.6 | 0.6 | 1.4 | 1.9 | 0.5 | 1.0 | 2.2 | 0.4 | | 0.7 | 0.9 | 0.3 | 0.8 |
| Guinea | 0.4 | 0.6 | 9.2 | 3.5 | 1.6 | 4.2 | 0.5 | 0.4 | 5.4 | 0.0 | -0.1 | | 5.1 | -0.9 | -1.2 | 5.2 |
| Haiti | 2.4 | 1.6 | 5.8 | 2.5 | 1.5 | 4.8 | 1.3 | 0.9 | 4.3 | 1.6 | 1.0 | | 3.6 | 1.6 | 0.8 | 2.2 |
| Honduras | 0.5 | 0.0 | 0.3 | 0.8 | 0.0 | 0.4 | 0.0 | -0.6 | 0.9 | 0.6 | 0.0 | | 0.3 | 9.5 | 0.0 | 0.0 |
| India | 4.1 | 1.6 | 3.5 | 3.9 | 1.2 | 2.3 | 2.2 | 0.8 | 2.0 | 1.2 | 0.5 | | 1.7 | 0.2 | 0.2 | 1.5 |
| Indonesia | 1.7 | 1.1 | 4.4 | 1.9 | 0.9 | 2.7 | 1.2 | 0.6 | 2.2 | 1.5 | 0.6 | | 1.5 | 0.3 | 0.1 | 1.7 |
| Jordan | -0.1 | -0.1 | 0.5 | 0.1 | 0.0 | 0.2 | 0.1 | 0.0 | 0.2 | 0.0 | 0.0 | | 0.2 | 0.0 | 0.0 | 0.2 |
| Kazakhstan | 12.3 | 0.0 | 0.0 | 16.1 | 0.0 | 0.0 | 12.6 | 0.0 | 0.0 | 9.1 | 0.0 | | 0.0 | 5.4 | 0.0 | 0.0 |
| Kenya | 1.0 | 0.5 | 1.9 | 0.7 | 0.3 | 1.0 | 0.5 | 0.2 | 0.6 | 0.3 | 0.1 | | 0.8 | 0.3 | 0.1 | 1.0 |
| Kyrgyz Republic | 0.9 | 0.2 | 0.5 | 0.8 | 0.2 | 0.7 | 0.7 | 0.2 | 0.9 | -0.1 | -0.2 | | 2.3 | -0.8 | -1.6 | 3.2 |
| Lesotho | 0.6 | 0.1 | 1.9 | 0.2 | -0.1 | 1.9 | 2.1 | 0.3 | 0.5 | 0.9 | 0.2 | | 0.8 | -0.9 | -3.0 | 2.9 |
| Liberia | 11.4 | 1.5 | 2.0 | 12.7 | 0.8 | 0.9 | 5.9 | 0.9 | 1.5 | 3.7 | 0.6 | | 1.4 | 2.2 | 0.4 | 1.6 |
| Madagascar | 3.8 | 1.5 | 3.6 | 4.6 | 1.0 | 1.6 | 5.0 | 0.6 | 0.9 | 2.2 | 0.3 | | 0.6 | 1.1 | 0.2 | 0.3 |
| Malawi | 1.3 | 0.3 | 0.7 | 0.8 | 0.3 | 0.6 | 0.9 | 0.3 | 0.6 | 0.7 | 0.2 | | 0.4 | 0.2 | 0.1 | 0.6 |
| Maldives | -0.9 | -15.1 | 3.0 | -0.8 | -25.1 | 2.9 | -0.7 | -9.5 | 2.3 | -0.5 | -7.0 | | 1.4 | -1.1 | -16.5 | 3.5 |
| Mali | 5.6 | 1.6 | 3.0 | 4.3 | 1.4 | 3.0 | 4.7 | 1.3 | 2.4 | 3.4 | 1.0 | | 1.9 | 0.9 | 0.4 | 1.9 |
| Morocco | 2.7 | 0.1 | 0.1 | 1.7 | 0.0 | 0.1 | 0.8 | 0.0 | 0.1 | 0.6 | 0.0 | | 0.1 | 0.1 | 0.0 | 0.1 |
| Mozambique | 4.5 | 1.2 | 2.1 | 1.7 | 0.8 | 2.5 | 3.0 | 0.6 | 1.0 | 0.8 | 0.3 | | 0.8 | -0.1 | -0.3 | 0.8 |
| Namibia | 1.2 | 0.4 | 1.2 | 1.0 | 0.4 | 1.2 | 1.3 | 0.4 | 1.0 | 1.1 | 0.3 | | 0.7 | 0.5 | 0.2 | 1.3 |
| Nepal | 4.1 | 0.5 | 0.7 | 2.8 | 0.4 | 0.7 | 1.7 | 0.3 | 0.7 | 1.9 | 0.2 | | 0.3 | 0.7 | 0.1 | 0.3 |
| Nicaragua | 0.7 | -0.1 | 2.9 | 2.4 | 0.0 | 0.4 | 0.6 | -0.2 | 1.4 | -3.4 | -3.7 | | 9.7 | 2.7 | 0.0 | 0.4 |
| Niger | 12.1 | 1.7 | 2.1 | 11.1 | 1.2 | 1.5 | 11.9 | 1.1 | 1.4 | 7.2 | 1.2 | | 1.8 | 1.7 | 0.5 | 1.5 |
| Nigeria | -0.3 | 1.5 | 22.9 | 7.5 | 4.7 | 9.6 | 10.0 | 2.8 | 4.1 | 9.6 | 1.4 | | 1.9 | 3.2 | 0.6 | 1.2 |
| Pakistan | 2.6 | 1.9 | 7.3 | 3.4 | 1.4 | 3.0 | 2.7 | 1.1 | 2.4 | 2.3 | 0.7 | | 1.3 | 1.7 | 0.4 | 0.9 |
| Peru | 1.1 | 0.6 | 1.9 | 0.6 | 0.4 | 1.8 | 0.2 | 0.1 | 1.9 | -0.2 | -0.3 | | 2.0 | -0.1 | -0.2 | 1.7 |
| Philippines | 0.4 | 0.3 | 2.9 | 0.3 | 0.2 | 1.5 | 0.0 | 0.0 | 1.5 | 0.3 | 0.1 | | 0.8 | 0.0 | 0.0 | 0.8 |
| Rwanda | 0.8 | 0.2 | 0.4 | 1.3 | 0.1 | 0.2 | 0.9 | 0.1 | 0.2 | 3.7 | 0.0 | | 0.0 | 0.9 | 0.1 | 0.1 |
| Senegal | 1.5 | 0.6 | 1.6 | 2.7 | 0.5 | 0.8 | 1.9 | 0.3 | 0.6 | 3.9 | 0.3 | | 0.4 | 9.7 | 0.1 | 0.1 |
| Sierra Leone | 9.7 | 0.6 | 0.8 | 7.4 | 0.7 | 1.0 | 6.4 | 0.6 | 1.0 | 9.8 | 0.5 | | 0.7 | 0.3 | -0.4 | 3.1 |
| South Africa | 0.6 | 0.4 | 3.1 | -1.5 | -2.6 | 5.9 | -0.8 | -1.4 | 4.0 | -1.1 | -2.2 | | 4.1 | -1.9 | -4.5 | 6.2 |
| Tajikistan | -0.1 | -1.2 | 1.3 | -0.3 | -2.8 | 1.3 | 0.7 | -0.2 | 1.1 | 0.5 | -0.3 | | 1.4 | -0.8 | -4.1 | 3.0 |
| Tanzania | 1.1 | 0.5 | 2.2 | 0.2 | 0.0 | 2.0 | 1.0 | 0.3 | 1.4 | 0.3 | 0.0 | | 1.1 | 0.5 | 0.1 | 0.7 |
| Timor-Leste | -1.3 | -1.7 | 7.1 | -0.8 | -1.4 | 5.4 | -1.3 | -2.7 | 5.4 | -0.8 | -2.0 | | 3.8 | 0.2 | -0.5 | 3.1 |
| Togo | 7.5 | 0.6 | 0.8 | 5.6 | 0.9 | 1.4 | 3.9 | 0.9 | 1.5 | 3.9 | 0.7 | | 1.1 | 2.3 | 0.3 | 0.6 |
| Turkey | 3.0 | 0.9 | 1.8 | 2.0 | 0.5 | 1.2 | 1.4 | 0.3 | 0.8 | 0.9 | 0.2 | | 0.5 | 0.2 | 0.1 | 0.9 |
| Uganda | 4.1 | 1.3 | 2.6 | 2.5 | 1.1 | 3.4 | 2.2 | 1.0 | 3.5 | 2.3 | 1.1 | | 3.3 | 0.2 | 0.0 | 4.1 |
| Vietnam | -0.8 | -0.5 | 7.0 | -1.0 | -0.9 | 5.8 | 3.0 | 0.2 | 0.6 | 3.1 | 0.1 | | 0.4 | 2.9 | 0.0 | 0.0 |
| Zambia | -1.1 | -1.4 | 6.0 | -1.2 | -1.6 | 5.7 | -1.0 | -1.5 | 5.0 | -0.2 | -0.5 | | 2.7 | -0.6 | -1.4 | 2.6 |
| Zimbabwe | -0.3 | -0.5 | 3.4 | -0.7 | -1.1 | 3.6 | 0.1 | -0.1 | 2.0 | -0.2 | -0.3 | | 2.3 | -0.5 | -0.9 | 2.5 |

**Note:** AARC is calculated as ln((rate in latest year/rate in earliest year))/ (latest year - earliest year), with positive values denote increasing rate while negative values denote decreasing rate

Table S51: Predicted average annual rate of increase (AARC) of three doses of DPT immunization among children 12-23 months of age in low- and middle-income countries by place of residence

| **Country** | **Rural** | | | **Urban** | | |
| --- | --- | --- | --- | --- | --- | --- |
|  | **Predicted** | | **Required** | **Predicted** | | **Required** |
|  | **1990-2018** | **2019-2030** | **2019-2030** | **1990-2018** | **2019-2030** | **2019-2030** |
| Armenia | 0.6 | 0.1 | 0.6 | 1.0 | 0.3 | 0.8 |
| Bangladesh | 2.2 | 0.3 | 0.4 | 1.3 | 0.2 | 0.3 |
| Benin | 0.0 | 0.0 | 3.9 | 0.1 | 0.0 | 2.6 |
| Bolivia | 6.3 | 0.2 | 0.2 | 3.3 | 0.3 | 0.5 |
| Brazil | 1.1 | 0.2 | 2.2 | 1.9 | 0.0 | 0.1 |
| Burkina Faso | 6.3 | 0.3 | 0.4 | 1.7 | 0.3 | 0.6 |
| Burundi | 0.5 | 0.0 | 0.3 | 1.0 | 0.1 | 0.5 |
| Cambodia | 3.7 | 0.5 | 0.9 | 5.8 | 0.2 | 0.3 |
| Cameroon | 2.5 | 1.1 | 3.0 | 1.5 | 0.6 | 1.4 |
| Chad | 4.9 | 3.4 | 9.3 | -0.2 | -0.2 | 8.4 |
| Colombia | 0.8 | 0.3 | 1.3 | 0.4 | 0.2 | 1.0 |
| Comoros | 0.3 | 0.2 | 2.9 | 0.4 | 0.2 | 2.6 |
| Congo | -1.7 | -1.9 | 8.3 | -3.2 | -9.3 | 9.2 |
| Congo DR | 5.6 | 2.0 | 3.8 | 3.9 | 0.9 | 1.9 |
| Cote d'Ivoire | 1.9 | 1.1 | 4.1 | 0.5 | 0.3 | 2.1 |
| Dominican Republic | 2.4 | 0.5 | 0.9 | 1.1 | 0.5 | 1.6 |
| Egypt | 1.2 | 0.1 | 0.1 | 0.8 | 0.1 | 0.1 |
| Ethiopia | 6.4 | 2.9 | 5.5 | 2.8 | 0.9 | 2.1 |
| Gabon | 0.7 | 1.2 | 12.6 | -4.7 | -5.2 | 17.2 |
| Ghana | 2.4 | 0.3 | 0.5 | 0.6 | 0.3 | 0.9 |
| Guatemala | 1.6 | 0.6 | 1.3 | 1.8 | 0.5 | 1.0 |
| Guinea | 1.1 | 0.8 | 6.0 | -0.6 | -0.7 | 5.2 |
| Haiti | 2.0 | 1.3 | 4.8 | 1.5 | 0.9 | 3.5 |
| Honduras | 1.2 | 0.1 | 0.2 | 0.8 | 0.0 | 0.5 |
| India | 2.7 | 1.0 | 2.4 | 0.8 | 0.4 | 2.0 |
| Indonesia | 1.6 | 0.9 | 2.8 | 0.3 | 0.2 | 2.3 |
| Jordan | 0.1 | 0.0 | 0.4 | 0.0 | 0.0 | 0.3 |
| Kazakhstan | 13.9 | 0.0 | 0.0 | 8.0 | 0.0 | 0.0 |
| Kenya | 0.6 | 0.3 | 1.1 | 0.5 | 0.2 | 1.1 |
| Kyrgyz Republic | 0.6 | 0.2 | 1.1 | -0.7 | -1.3 | 2.7 |
| Lesotho | 0.6 | 0.2 | 1.3 | -0.1 | -0.6 | 1.7 |
| Liberia | 9.3 | 1.2 | 1.6 | 1.6 | 0.4 | 2.2 |
| Madagascar | 2.3 | 0.9 | 2.3 | 1.9 | 0.3 | 0.6 |
| Malawi | 0.9 | 0.3 | 0.6 | 0.2 | 0.1 | 0.7 |
| Maldives | -0.8 | -13.7 | 2.7 | -0.6 | -11.9 | 2.1 |
| Mali | 4.9 | 1.4 | 2.5 | 1.2 | 0.6 | 1.7 |
| Morocco | 1.8 | 0.1 | 0.1 | 0.3 | 0.0 | 0.1 |
| Mozambique | 2.5 | 0.8 | 1.7 | -0.3 | -0.6 | 1.5 |
| Namibia | 1.4 | 0.4 | 0.8 | 0.5 | 0.2 | 1.4 |
| Nepal | 2.4 | 0.4 | 0.6 | 0.9 | 0.2 | 0.7 |
| Nicaragua | 1.0 | -0.1 | 1.9 | 0.9 | -0.2 | 1.3 |
| Niger | 8.7 | 1.6 | 2.1 | 1.5 | 0.5 | 1.4 |
| Nigeria | 0.3 | 0.6 | 13.2 | 0.6 | 0.5 | 5.0 |
| Pakistan | 2.7 | 1.3 | 3.3 | 1.8 | 0.7 | 1.7 |
| Peru | 1.1 | 0.5 | 1.7 | 0.1 | 0.0 | 1.9 |
| Philippines | 0.3 | 0.2 | 1.8 | 0.2 | 0.1 | 1.3 |
| Rwanda | 1.0 | 0.1 | 0.2 | 0.9 | 0.1 | 0.2 |
| Senegal | 2.8 | 0.5 | 0.9 | 1.6 | 0.3 | 0.5 |
| Sierra Leone | 8.5 | 0.7 | 0.9 | 2.7 | 0.5 | 1.7 |
| South Africa | -0.3 | -0.4 | 3.8 | -1.4 | -2.7 | 5.0 |
| Tajikistan | 0.2 | -0.6 | 1.2 | -0.8 | -3.9 | 2.8 |
| Tanzania | 0.5 | 0.3 | 1.9 | 0.5 | 0.1 | 0.7 |
| Timor-Leste | -0.8 | -1.5 | 5.0 | -0.7 | -1.6 | 4.2 |
| Togo | 5.7 | 0.8 | 1.1 | 2.3 | 0.5 | 1.0 |
| Turkey | 2.2 | 0.7 | 1.7 | 1.0 | 0.3 | 1.0 |
| Uganda | 2.5 | 1.1 | 3.3 | 0.7 | 0.3 | 3.7 |
| Vietnam | 1.1 | 0.1 | 2.6 | 2.5 | 0.0 | 0.2 |
| Yemen | 1.2 | 0.8 | 5.1 | 0.0 | -0.1 | 2.5 |
| Zambia | -0.4 | -0.6 | 4.6 | -0.2 | -0.4 | 2.5 |
| Zimbabwe | -0.3 | -0.5 | 3.0 | -0.5 | -0.9 | 2.7 |

**Note:** AARC is calculated as ln((rate in latest year/rate in earliest year))/ (latest year - earliest year), with positive values denote increasing rate while negative values denote decreasing rate

Table S52: Predicted average annual rate of increase (AARC) of three doses of DPT immunization among children 12-23 months of age in low- and middle-income countries by education of mother

| **Country** | **Below secondary education** | | | **Secondary+ education** | | |
| --- | --- | --- | --- | --- | --- | --- |
|  | **Predicted** | | **Required** | **Predicted** | | **Required** |
|  | **1990-2018** | **2019-2030** | **2019-2030** | **1990-2018** | **2019-2030** | **2019-2030** |
| Armenia | -2.2 | -40.7 | 7.7 | 0.8 | 0.2 | 0.7 |
| Bangladesh | 2.0 | 0.4 | 0.7 | 0.9 | 0.1 | 0.2 |
| Benin | 0.0 | -0.1 | 3.8 | -0.7 | -2.4 | 2.3 |
| Bolivia | 5.7 | 0.3 | 0.4 | 3.4 | 0.2 | 0.2 |
| Brazil | 2.1 | 0.1 | 0.3 | 0.0 | -0.2 | 2.0 |
| Burkina Faso | 5.6 | 0.3 | 0.4 | 0.3 | 0.1 | 0.9 |
| Burundi | 0.5 | 0.0 | 0.3 | 1.5 | 0.1 | 0.3 |
| Cambodia | 3.7 | 0.6 | 1.1 | 2.3 | 0.2 | 0.3 |
| Cameroon | 2.3 | 1.1 | 3.1 | 1.3 | 0.5 | 1.2 |
| Chad | 2.9 | 2.4 | 10.1 | -0.1 | -0.1 | 5.7 |
| Colombia | 0.7 | 0.3 | 1.3 | 0.2 | 0.1 | 1.1 |
| Comoros | 0.3 | 0.1 | 3.3 | -0.3 | -0.5 | 2.4 |
| Congo | -1.1 | -1.2 | 7.0 | -3.0 | -7.3 | 8.8 |
| Congo DR | 5.0 | 2.0 | 4.1 | 3.4 | 1.0 | 2.3 |
| Cote d'Ivoire | 1.4 | 0.8 | 3.5 | 0.0 | -0.2 | 1.3 |
| Dominican Republic | 2.2 | 0.7 | 1.8 | 0.6 | 0.3 | 1.3 |
| Egypt | 1.1 | 0.1 | 0.2 | 0.6 | 0.1 | 0.1 |
| Ethiopia | 6.1 | 2.8 | 5.3 | 3.3 | 0.9 | 1.6 |
| Gabon | -1.3 | -0.7 | 13.4 | -5.2 | -6.1 | 18.1 |
| Ghana | 2.0 | 0.5 | 0.8 | 0.2 | 0.1 | 0.8 |
| Guatemala | 1.6 | 0.6 | 1.5 | 1.3 | 0.3 | 0.7 |
| Guinea | 0.3 | 0.3 | 6.4 | 0.4 | 0.2 | 2.8 |
| Haiti | 1.3 | 1.0 | 5.7 | 1.0 | 0.6 | 2.9 |
| Honduras | 0.7 | 0.0 | 0.4 | 1.3 | 0.1 | 0.2 |
| India | 2.7 | 1.2 | 3.4 | 0.2 | 0.1 | 1.8 |
| Indonesia | 1.1 | 0.8 | 4.2 | 0.1 | 0.0 | 2.3 |
| Jordan | -0.3 | -0.6 | 1.5 | 0.0 | 0.0 | 0.2 |
| Kazakhstan | N/A | N/A | N/A | 11.2 | 0.0 | 0.0 |
| Kenya | 0.6 | 0.3 | 1.3 | 0.3 | 0.1 | 0.7 |
| Kyrgyz Republic | N/A | N/A | N/A | 0.3 | 0.1 | 1.4 |
| Lesotho | 0.7 | 0.2 | 1.4 | -0.2 | -0.4 | 1.6 |
| Liberia | 7.1 | 1.2 | 1.9 | 3.4 | 0.6 | 1.2 |
| Madagascar | 2.3 | 1.0 | 2.6 | 1.8 | 0.2 | 0.4 |
| Malawi | 0.8 | 0.3 | 0.6 | 0.0 | 0.0 | 0.4 |
| Maldives | -0.9 | -17.7 | 2.9 | -0.8 | -11.3 | 2.4 |
| Mali | 3.6 | 1.2 | 2.6 | 0.1 | 0.0 | 1.8 |
| Morocco | 1.4 | 0.1 | 0.1 | 0.1 | 0.0 | 0.1 |
| Mozambique | 1.7 | 0.7 | 1.8 | -0.4 | -1.6 | 1.3 |
| Namibia | 1.0 | 0.4 | 1.4 | 0.9 | 0.3 | 1.0 |
| Nepal | 2.4 | 0.5 | 0.8 | 0.3 | 0.1 | 0.3 |
| Nicaragua | 1.3 | -0.1 | 1.4 | 0.4 | -0.3 | 1.5 |
| Niger | 6.3 | 1.5 | 2.4 | 0.9 | 0.4 | 1.3 |
| Nigeria | -0.9 | -0.4 | 16.5 | 0.2 | 0.1 | 3.5 |
| Pakistan | 2.1 | 1.3 | 4.0 | 1.6 | 0.4 | 0.9 |
| Peru | 1.0 | 0.5 | 1.9 | 0.0 | 0.0 | 1.9 |
| Philippines | -0.1 | -0.2 | 3.6 | 0.1 | 0.0 | 1.2 |
| Rwanda | 1.1 | 0.1 | 0.2 | 0.7 | 0.1 | 0.1 |
| Senegal | 2.3 | 0.5 | 0.8 | 0.3 | 0.1 | 0.1 |
| Sierra Leone | 6.9 | 0.7 | 1.1 | 2.1 | 0.3 | 1.4 |
| South Africa | -1.1 | -1.6 | 5.5 | -1.1 | -1.8 | 4.5 |
| Tajikistan | 2.8 | 0.4 | 0.9 | -0.2 | -1.6 | 1.6 |
| Tanzania | 0.5 | 0.2 | 1.7 | 0.3 | 0.1 | 1.0 |
| Timor-Leste | -0.9 | -1.5 | 5.6 | -1.1 | -2.9 | 4.5 |
| Togo | 4.9 | 0.8 | 1.2 | 1.9 | 0.4 | 0.8 |
| Turkey | 1.6 | 0.5 | 1.3 | 0.5 | 0.2 | 1.0 |
| Uganda | 2.4 | 1.1 | 3.5 | 0.4 | 0.1 | 3.3 |
| Vietnam | 0.3 | 0.0 | 4.3 | 1.7 | 0.2 | 1.2 |
| Yemen | 0.8 | 0.6 | 4.9 | -0.4 | -0.6 | 2.0 |
| Zambia | -0.5 | -0.6 | 4.5 | -0.9 | -1.6 | 3.4 |
| Zimbabwe | -0.5 | -0.8 | 3.7 | -0.6 | -1.2 | 2.8 |

**Note:** AARC is calculated as ln((rate in latest year/rate in earliest year))/ (latest year - earliest year), with positive values denote increasing rate while negative values denote decreasing rate

Table S53: Predicted average annual rate of increase (AARC) of three doses of DPT immunization among children 12-23 months of age in low- and middle-income countries by age of mother

| **Country** | **Adolescent mother** | | | **Adult mother** | | |
| --- | --- | --- | --- | --- | --- | --- |
|  | **Predicted** | | **Required** | **Predicted** | | **Required** |
|  | **1990-2018** | **2019-2030** | **2019-2030** | **1990-2018** | **2019-2030** | **2019-2030** |
| Armenia | 2.5 | 0.0 | 0.0 | 0.7 | 0.2 | 0.8 |
| Bangladesh | 2.3 | 0.3 | 0.4 | 2.1 | 0.2 | 0.4 |
| Benin | 0.0 | -0.1 | 3.8 | 0.1 | 0.0 | 3.4 |
| Bolivia | 5.9 | 0.2 | 0.2 | 4.5 | 0.3 | 0.4 |
| Brazil | 1.2 | 0.2 | 1.5 | 2.0 | 0.0 | 0.1 |
| Burkina Faso | 6.5 | 0.4 | 0.5 | 5.4 | 0.3 | 0.4 |
| Burundi | 0.2 | -0.3 | 1.0 | 0.5 | 0.0 | 0.3 |
| Cambodia | 3.7 | 0.5 | 0.8 | 4.0 | 0.5 | 0.7 |
| Cameroon | 2.1 | 0.9 | 2.8 | 2.1 | 0.9 | 2.2 |
| Chad | 1.7 | 1.6 | 10.3 | 3.3 | 2.5 | 9.2 |
| Colombia | 1.4 | 0.4 | 0.8 | 0.5 | 0.2 | 1.1 |
| Comoros | -2.2 | -3.7 | 8.0 | 0.6 | 0.3 | 2.5 |
| Congo | -4.2 | -8.7 | 12.2 | -1.9 | -3.1 | 7.1 |
| Congo DR | 5.7 | 1.6 | 2.8 | 4.3 | 1.5 | 3.3 |
| Cote d'Ivoire | 1.6 | 1.0 | 4.3 | 1.2 | 0.7 | 3.2 |
| Dominican Republic | 2.9 | 0.8 | 1.6 | 1.4 | 0.5 | 1.4 |
| Egypt | 1.3 | 0.1 | 0.1 | 1.0 | 0.1 | 0.1 |
| Ethiopia | 6.4 | 3.1 | 5.9 | 5.7 | 2.6 | 5.0 |
| Gabon | -4.3 | -4.1 | 18.2 | -3.7 | -3.7 | 16.0 |
| Ghana | 1.8 | 0.5 | 0.9 | 1.8 | 0.3 | 0.6 |
| Guatemala | 1.8 | 0.6 | 1.5 | 1.7 | 0.5 | 1.2 |
| Guinea | -0.5 | -0.5 | 7.2 | 0.6 | 0.5 | 5.7 |
| Haiti | 1.9 | 1.2 | 4.8 | 1.7 | 1.1 | 4.3 |
| Honduras | 0.7 | 0.0 | 0.4 | 0.9 | 0.0 | 0.3 |
| India | 2.3 | 1.0 | 2.5 | 2.1 | 0.9 | 2.3 |
| Indonesia | 1.6 | 0.9 | 3.7 | 1.2 | 0.7 | 2.4 |
| Jordan | 0.1 | 0.0 | 0.0 | 0.0 | 0.0 | 0.3 |
| Kazakhstan | 5.3 | 0.0 | 0.2 | 11.6 | 0.0 | 0.0 |
| Kenya | 1.1 | 0.3 | 0.8 | 0.5 | 0.2 | 1.1 |
| Kyrgyz Republic | 2.0 | 0.1 | 0.2 | 0.2 | 0.1 | 1.4 |
| Lesotho | -0.3 | -0.7 | 2.2 | 0.5 | 0.2 | 1.3 |
| Liberia | 4.1 | 1.0 | 2.2 | 7.3 | 1.0 | 1.5 |
| Madagascar | 2.5 | 1.0 | 3.2 | 2.1 | 0.8 | 1.9 |
| Malawi | 0.8 | 0.2 | 0.5 | 0.8 | 0.2 | 0.6 |
| Maldives | -6.5 | -29.2 | 19.2 | -0.8 | -13.8 | 2.5 |
| Mali | 3.3 | 1.2 | 2.6 | 3.4 | 1.1 | 2.5 |
| Morocco | 2.1 | 0.0 | 0.0 | 1.3 | 0.1 | 0.1 |
| Mozambique | 0.9 | 0.4 | 1.5 | 1.8 | 0.7 | 1.6 |
| Namibia | 1.1 | 0.4 | 1.2 | 0.9 | 0.4 | 1.1 |
| Nepal | 2.1 | 0.4 | 0.6 | 2.4 | 0.4 | 0.6 |
| Nicaragua | 3.1 | 0.0 | 0.4 | 0.6 | -0.2 | 1.8 |
| Niger | 5.7 | 1.7 | 3.1 | 6.1 | 1.4 | 2.3 |
| Nigeria | -0.5 | 0.0 | 16.2 | 1.1 | 1.0 | 8.7 |
| Pakistan | 3.9 | 1.7 | 3.7 | 2.3 | 1.1 | 2.7 |
| Peru | 1.0 | 0.5 | 2.0 | 0.5 | 0.3 | 1.7 |
| Philippines | 0.6 | 0.3 | 2.1 | 0.2 | 0.1 | 1.6 |
| Rwanda | 3.8 | 0.0 | 0.0 | 1.0 | 0.1 | 0.2 |
| Senegal | 2.7 | 0.6 | 1.0 | 2.2 | 0.4 | 0.7 |
| Sierra Leone | 6.0 | 0.6 | 1.0 | 6.5 | 0.7 | 1.1 |
| South Africa | -0.2 | -0.3 | 3.7 | -1.0 | -1.6 | 4.6 |
| Tajikistan | -2.3 | -140.3 | 14.3 | 0.0 | -1.0 | 1.5 |
| Tanzania | 0.5 | 0.2 | 1.7 | 0.6 | 0.2 | 1.6 |
| Timor-Leste | -1.2 | -2.4 | 5.5 | -0.7 | -1.4 | 4.8 |
| Togo | 5.9 | 0.7 | 0.9 | 4.7 | 0.7 | 1.1 |
| Turkey | 1.7 | 0.5 | 1.3 | 1.5 | 0.4 | 1.0 |
| Uganda | 2.8 | 1.1 | 2.9 | 2.2 | 1.0 | 3.4 |
| Vietnam | 2.1 | 0.3 | 1.9 | 1.3 | 0.1 | 2.0 |
| Yemen | 1.9 | 1.0 | 3.2 | 1.0 | 0.7 | 4.3 |
| Zambia | -0.2 | -0.4 | 3.5 | -0.5 | -0.7 | 4.1 |
| Zimbabwe | 0.0 | -0.1 | 2.7 | -0.4 | -0.7 | 2.9 |

**Note:** AARC is calculated as ln((rate in latest year/rate in earliest year))/ (latest year - earliest year), with positive values denote increasing rate while negative values denote decreasing rate

Table S54: Predicted average annual rate of increase (AARC) of three doses of DPT immunization among children 12-23 months of age in low- and middle-income countries by child sex

| **Country** | **Male** | | | **Female** | | |
| --- | --- | --- | --- | --- | --- | --- |
|  | **Predicted** | | **Required** | **Predicted** | | **Required** |
|  | **1990-2018** | **2019-2030** | **2019-2030** | **1990-2018** | **2019-2030** | **2019-2030** |
| Armenia | 0.9 | 0.2 | 0.7 | 0.7 | 0.2 | 0.8 |
| Bangladesh | 1.9 | 0.2 | 0.4 | 2.4 | 0.2 | 0.4 |
| Benin | 0.1 | 0.1 | 3.3 | 0.0 | 0.0 | 3.4 |
| Bolivia | 4.4 | 0.3 | 0.4 | 4.8 | 0.2 | 0.3 |
| Brazil | 2.2 | 0.0 | 0.1 | 1.5 | 0.0 | 0.4 |
| Burkina Faso | 5.7 | 0.3 | 0.4 | 5.3 | 0.4 | 0.5 |
| Burundi | 0.4 | 0.0 | 0.3 | 0.6 | 0.1 | 0.3 |
| Cambodia | 3.4 | 0.5 | 0.8 | 4.6 | 0.5 | 0.7 |
| Cameroon | 1.8 | 0.9 | 2.4 | 2.4 | 0.9 | 2.2 |
| Chad | 2.7 | 2.2 | 9.6 | 3.6 | 2.6 | 8.9 |
| Colombia | 0.6 | 0.3 | 0.9 | 0.5 | 0.2 | 1.3 |
| Comoros | 0.1 | 0.0 | 3.4 | 0.6 | 0.3 | 2.2 |
| Congo | -1.5 | -2.4 | 6.3 | -2.8 | -5.1 | 8.9 |
| Congo DR | 4.3 | 1.5 | 3.4 | 4.4 | 1.5 | 3.2 |
| Cote d'Ivoire | 1.4 | 0.8 | 3.0 | 1.2 | 0.7 | 3.6 |
| Dominican Republic | 1.5 | 0.5 | 1.3 | 1.5 | 0.5 | 1.5 |
| Egypt | 1.0 | 0.1 | 0.1 | 1.1 | 0.1 | 0.1 |
| Ethiopia | 5.1 | 2.5 | 5.2 | 6.6 | 2.7 | 4.8 |
| Gabon | -3.7 | -3.8 | 15.5 | -3.7 | -3.4 | 16.8 |
| Ghana | 1.6 | 0.4 | 0.7 | 1.9 | 0.3 | 0.5 |
| Guatemala | 1.4 | 0.5 | 1.3 | 2.0 | 0.6 | 1.1 |
| Guinea | 1.0 | 0.7 | 5.1 | 0.0 | 0.0 | 6.6 |
| Haiti | 2.0 | 1.2 | 4.3 | 1.5 | 1.0 | 4.3 |
| Honduras | 1.1 | 0.1 | 0.3 | 0.7 | 0.0 | 0.4 |
| India | 2.1 | 0.9 | 2.2 | 2.4 | 1.0 | 2.3 |
| Indonesia | 1.3 | 0.7 | 2.5 | 1.3 | 0.7 | 2.4 |
| Jordan | 0.0 | 0.0 | 0.4 | 0.0 | 0.0 | 0.3 |
| Kazakhstan | 12.0 | 0.0 | 0.0 | 10.6 | 0.0 | 0.0 |
| Kenya | 0.4 | 0.2 | 1.3 | 0.8 | 0.3 | 0.9 |
| Kyrgyz Republic | 0.1 | 0.0 | 1.5 | 0.4 | 0.1 | 1.4 |
| Lesotho | 0.6 | 0.2 | 1.3 | 0.2 | 0.0 | 1.5 |
| Liberia | 6.5 | 1.1 | 1.8 | 7.4 | 0.9 | 1.3 |
| Madagascar | 1.8 | 0.8 | 2.6 | 2.6 | 0.7 | 1.5 |
| Malawi | 0.7 | 0.2 | 0.6 | 0.9 | 0.2 | 0.5 |
| Maldives | -0.8 | -14.1 | 2.7 | -0.7 | -12.2 | 2.3 |
| Mali | 3.3 | 1.1 | 2.4 | 3.5 | 1.2 | 2.7 |
| Morocco | 1.3 | 0.1 | 0.1 | 1.4 | 0.0 | 0.1 |
| Mozambique | 1.5 | 0.6 | 1.5 | 2.0 | 0.7 | 1.6 |
| Namibia | 1.2 | 0.4 | 0.9 | 0.7 | 0.3 | 1.4 |
| Nepal | 2.0 | 0.3 | 0.6 | 2.7 | 0.4 | 0.6 |
| Nicaragua | 0.4 | -0.3 | 2.2 | 1.3 | -0.1 | 1.2 |
| Niger | 6.3 | 1.5 | 2.3 | 5.9 | 1.4 | 2.3 |
| Nigeria | 1.0 | 1.0 | 9.2 | 0.8 | 0.8 | 9.3 |
| Pakistan | 2.4 | 1.0 | 2.5 | 2.4 | 1.2 | 3.0 |
| Peru | 0.6 | 0.3 | 1.6 | 0.4 | 0.3 | 1.9 |
| Philippines | 0.3 | 0.2 | 1.5 | 0.1 | 0.1 | 1.7 |
| Rwanda | 1.2 | 0.1 | 0.2 | 0.9 | 0.1 | 0.2 |
| Senegal | 2.4 | 0.5 | 0.8 | 2.2 | 0.4 | 0.7 |
| Sierra Leone | 6.7 | 0.7 | 1.1 | 6.9 | 0.7 | 1.0 |
| South Africa | -0.6 | -0.9 | 4.0 | -1.3 | -2.2 | 5.0 |
| Tajikistan | -0.5 | -3.1 | 1.8 | 0.7 | -0.2 | 1.2 |
| Tanzania | 0.6 | 0.2 | 1.5 | 0.5 | 0.2 | 1.7 |
| Timor-Leste | -1.1 | -2.4 | 5.1 | -0.2 | -0.7 | 4.5 |
| Togo | 4.8 | 0.6 | 0.9 | 4.8 | 0.8 | 1.2 |
| Turkey | 1.6 | 0.4 | 0.9 | 1.5 | 0.5 | 1.2 |
| Uganda | 2.2 | 1.0 | 3.4 | 2.4 | 1.1 | 3.3 |
| Vietnam | 1.8 | 0.2 | 1.6 | 0.8 | 0.0 | 2.4 |
| Yemen | 0.9 | 0.6 | 4.3 | 1.2 | 0.8 | 4.1 |
| Zambia | -0.4 | -0.6 | 3.9 | -0.5 | -0.8 | 4.1 |
| Zimbabwe | -0.3 | -0.4 | 2.8 | -0.5 | -0.8 | 2.9 |

**Note:** AARC is calculated as ln((rate in latest year/rate in earliest year))/ (latest year - earliest year), with positive values denote increasing rate while negative values denote decreasing rate

Table S55: Predicted average annual rate of increase (AARC) of Measles immunization among children 12-23 months of age in low- and middle-income countries

| **Country** | **Predicted** | | **Required** |
| --- | --- | --- | --- |
|  | **1990-2018** | **2019-2030** | **2019-2030** |
| Albania | 41.5 | 0.0 | 0.0 |
| Armenia | 1.4 | 0.4 | 1.1 |
| Bangladesh | 1.4 | 0.4 | 0.9 |
| Benin | 0.3 | 0.2 | 3.5 |
| Bolivia | 2.0 | 0.7 | 1.9 |
| Brazil | 1.1 | 0.0 | 0.2 |
| Burkina Faso | 3.4 | 0.5 | 0.9 |
| Burundi | 0.0 | -0.4 | 0.7 |
| Cambodia | 2.4 | 0.6 | 1.3 |
| Cameroon | 1.5 | 0.8 | 2.4 |
| Chad | 5.7 | 2.3 | 4.4 |
| Colombia | -0.2 | -0.4 | 3.3 |
| Comoros | 1.0 | 0.5 | 2.2 |
| Congo | 2.0 | 0.5 | 2.0 |
| Congo DR | 2.3 | 0.7 | 2.4 |
| Cote d'Ivoire | 0.7 | 0.4 | 3.4 |
| Dominican Republic | 0.8 | 0.3 | 1.0 |
| Egypt | -0.3 | -0.8 | 1.5 |
| Ethiopia | 5.0 | 2.1 | 4.1 |
| Gabon | 2.6 | 0.8 | 1.9 |
| Ghana | 1.7 | 0.3 | 0.6 |
| Guatemala | -1.0 | -1.6 | 4.5 |
| Guinea | 1.4 | 0.8 | 4.0 |
| Haiti | 1.1 | 0.8 | 4.0 |
| Honduras | 0.7 | -0.1 | 1.2 |
| India | 3.3 | 0.9 | 1.7 |
| Indonesia | 1.2 | 0.6 | 1.7 |
| Jordan | -0.1 | -0.1 | 1.0 |
| Kazakhstan | 2.2 | 0.1 | 0.7 |
| Kenya | 0.6 | 0.3 | 1.3 |
| Kyrgyz Republic | 0.4 | 0.2 | 0.9 |
| Lesotho | 1.0 | 0.2 | 1.0 |
| Liberia | 1.9 | 0.7 | 2.5 |
| Madagascar | 2.0 | 0.8 | 2.4 |
| Malawi | 0.8 | 0.3 | 0.7 |
| Maldives | -0.5 | -2.8 | 1.5 |
| Mali | 2.4 | 0.9 | 2.2 |
| Morocco | 1.1 | 0.2 | 0.3 |
| Mozambique | 1.9 | 0.6 | 1.2 |
| Namibia | 0.9 | 0.3 | 0.8 |
| Nepal | 2.5 | 0.4 | 0.6 |
| Nicaragua | -7.2 | -4.7 | 19.4 |
| Niger | 4.5 | 1.3 | 2.4 |
| Nigeria | -0.1 | 0.0 | 8.3 |
| Pakistan | 1.6 | 0.9 | 3.2 |
| Peru | -0.4 | -0.6 | 3.5 |
| Philippines | 0.1 | 0.1 | 1.7 |
| Rwanda | -0.5 | -1.1 | 2.5 |
| Senegal | 1.9 | 0.7 | 1.4 |
| Sierra Leone | 7.2 | 0.6 | 1.0 |
| South Africa | 0.2 | 0.1 | 1.5 |
| Tajikistan | -0.1 | -1.0 | 2.0 |
| Tanzania | 0.4 | 0.3 | 1.3 |
| Timor-Leste | 0.5 | -0.1 | 3.3 |
| Togo | 3.6 | 1.0 | 2.0 |
| Turkey | 0.8 | 0.4 | 1.0 |
| Uganda | 1.7 | 0.7 | 1.9 |
| Vietnam | 1.2 | 0.0 | 1.0 |
| Yemen | 1.7 | 1.0 | 3.5 |
| Zambia | 0.5 | 0.2 | 1.2 |
| Zimbabwe | -0.3 | -0.5 | 2.7 |

**Note:** AARC is calculated as ln((rate in latest year/rate in earliest year))/ (latest year - earliest year), with positive values denote increasing rate while negative values denote decreasing rate

Table S56: Predicted average annual rate of increase (AARC) of Measles immunization among children 12-23 months of age in low- and middle-income countries by wealth quintiles

| **Country** | **Poorest** | | | **Poorer** | | | **Middle** | | | **Richer** | | | **Richest** | | | |
| --- | --- | --- | --- | --- | --- | --- | --- | --- | --- | --- | --- | --- | --- | --- | --- | --- |
|  | **Predicted** | | **Required** | **Predicted** | | **Required** | **Predicted** | | **Required** | **Predicted** | | **Required** | | **Predicted** | | **Required** |
|  | **1990-2018** | **2019-2030** | **2019-2030** | **1990-2018** | **2019-2030** | **2019-2030** | **1990-2018** | **2019-2030** | **2019-2030** | **1990-2018** | **2019-2030** | **2019-2030** | | **1990-2018** | **2019-2030** | **2019-2030** |
| Albania | 41.4 | 0.0 | 0.0 | 41.1 | 0.0 | 0.0 | 43.1 | 0.0 | 0.0 | 45.9 | 0.0 | 0.0 | | 35.3 | 0.0 | 0.0 |
| Armenia | 2.6 | 0.4 | 0.6 | 1.2 | 0.5 | 1.4 | 0.8 | 0.3 | 0.9 | 0.9 | 0.3 | 1.1 | | 1.3 | 0.4 | 1.5 |
| Bangladesh | 1.6 | 0.7 | 1.8 | 1.9 | 0.5 | 0.9 | 1.3 | 0.4 | 0.9 | 1.1 | 0.3 | 0.6 | | 0.8 | 0.2 | 0.4 |
| Benin | 0.1 | 0.1 | 5.8 | 0.1 | 0.1 | 4.4 | 0.3 | 0.2 | 3.0 | 0.1 | 0.0 | 2.9 | | 0.0 | 0.0 | 1.9 |
| Bolivia | 4.0 | 0.6 | 1.0 | 3.2 | 0.8 | 1.5 | 1.6 | 0.6 | 2.4 | 0.9 | 0.4 | 2.6 | | -1.0 | -1.4 | 4.9 |
| Burkina Faso | 4.2 | 0.9 | 1.6 | 3.2 | 0.6 | 1.1 | 4.3 | 0.4 | 0.6 | 3.7 | 0.4 | 0.6 | | 1.8 | 0.3 | 0.5 |
| Burundi | 0.3 | -0.1 | 0.8 | 0.2 | -0.1 | 0.5 | 0.1 | -0.2 | 0.6 | -0.1 | -0.9 | 0.7 | | -0.2 | -1.2 | 0.7 |
| Cambodia | 2.3 | 0.9 | 2.5 | 2.2 | 0.7 | 1.7 | 2.5 | 0.6 | 1.1 | 2.9 | 0.4 | 0.6 | | 1.9 | 0.2 | 0.4 |
| Cameroon | 1.4 | 1.0 | 5.0 | 2.7 | 1.1 | 2.6 | 2.4 | 0.7 | 1.4 | 1.1 | 0.5 | 1.8 | | 0.7 | 0.3 | 1.0 |
| Chad | 9.5 | 3.2 | 5.0 | 7.0 | 2.6 | 4.7 | 7.3 | 2.3 | 3.6 | 3.5 | 1.9 | 5.5 | | 3.6 | 1.4 | 3.2 |
| Colombia | -1.1 | -1.3 | 5.6 | 0.1 | -0.1 | 2.9 | 0.0 | -0.2 | 2.6 | 0.5 | 0.1 | 1.7 | | -0.9 | -1.4 | 4.1 |
| Comoros | 1.2 | 0.7 | 3.8 | 1.4 | 0.7 | 2.7 | 1.3 | 0.5 | 1.3 | 1.1 | 0.4 | 1.3 | | -0.5 | -0.8 | 2.5 |
| Congo | 2.7 | 1.1 | 3.6 | 1.9 | 0.6 | 2.6 | 3.1 | 0.5 | 1.2 | 2.0 | 0.3 | 1.0 | | 0.7 | 0.0 | 1.1 |
| Congo DR | 2.9 | 1.2 | 3.5 | 2.8 | 1.0 | 2.8 | 3.2 | 0.9 | 2.1 | 2.0 | 0.6 | 2.1 | | 0.0 | -0.5 | 1.7 |
| Cote d'Ivoire | 2.3 | 1.3 | 4.5 | 0.1 | 0.0 | 4.8 | 1.0 | 0.7 | 3.8 | 0.5 | 0.3 | 2.4 | | 0.2 | 0.0 | 0.8 |
| Dominican Republic | 1.5 | 0.4 | 1.0 | 0.5 | 0.1 | 1.1 | -0.2 | -0.4 | 2.2 | 0.4 | 0.1 | 1.3 | | -0.1 | -0.2 | 1.1 |
| Egypt | -1.3 | -3.7 | 3.8 | -1.0 | -3.3 | 3.1 | -1.3 | -5.5 | 3.8 | -1.0 | -4.7 | 3.0 | | -2.8 | -18.8 | 8.4 |
| Ethiopia | 5.7 | 2.8 | 5.7 | 7.9 | 2.4 | 3.8 | 5.5 | 2.2 | 4.0 | 4.5 | 1.9 | 4.0 | | 3.4 | 0.9 | 1.8 |
| Gabon | 6.7 | 0.9 | 1.3 | 2.8 | 0.9 | 2.0 | 2.8 | 0.7 | 1.5 | 3.0 | 0.5 | 0.9 | | -1.1 | -1.6 | 5.4 |
| Ghana | 2.9 | 0.4 | 0.6 | 2.1 | 0.4 | 0.6 | 1.7 | 0.3 | 0.5 | 1.3 | 0.2 | 0.4 | | 0.4 | 0.1 | 0.5 |
| Guatemala | -1.1 | -1.6 | 5.4 | -1.2 | -1.9 | 5.0 | -0.9 | -1.4 | 4.6 | -0.4 | -0.6 | 3.3 | | -1.2 | -3.2 | 3.9 |
| Guinea | 2.5 | 1.5 | 5.4 | 2.7 | 1.3 | 3.8 | 0.8 | 0.5 | 4.2 | 0.8 | 0.4 | 3.7 | | 1.0 | 0.3 | 2.2 |
| Haiti | 1.6 | 1.1 | 4.7 | 1.4 | 1.0 | 4.4 | 0.9 | 0.6 | 3.7 | 1.1 | 0.7 | 3.4 | | 0.8 | 0.5 | 2.7 |
| Honduras | 1.6 | 0.2 | 0.7 | 0.4 | -0.2 | 1.2 | 0.3 | -0.4 | 1.5 | 0.4 | -0.3 | 1.6 | | 1.4 | 0.1 | 0.7 |
| India | 5.8 | 1.6 | 2.5 | 5.0 | 1.2 | 1.9 | 3.4 | 0.8 | 1.5 | 2.2 | 0.6 | 1.2 | | 1.0 | 0.3 | 0.9 |
| Indonesia | 1.1 | 0.7 | 3.1 | 1.0 | 0.5 | 2.1 | 0.9 | 0.4 | 1.6 | 0.5 | 0.3 | 1.6 | | 0.1 | 0.0 | 1.4 |
| Jordan | 0.0 | -0.1 | 1.2 | -0.1 | -0.2 | 1.1 | 0.1 | 0.0 | 0.6 | -0.1 | -0.2 | 0.9 | | -0.2 | -0.3 | 1.0 |
| Kazakhstan | 2.7 | 0.1 | 0.7 | 4.8 | 0.0 | 0.1 | 0.0 | -0.4 | 2.5 | 2.8 | 0.1 | 0.3 | | 1.2 | 0.0 | 1.6 |
| Kenya | 1.0 | 0.5 | 2.4 | 0.4 | 0.2 | 1.6 | 0.4 | 0.2 | 1.1 | 0.7 | 0.2 | 0.6 | | 0.3 | 0.2 | 0.5 |
| Kyrgyz Republic | 1.0 | 0.2 | 0.4 | 0.2 | 0.1 | 1.0 | 0.1 | 0.0 | 1.0 | -0.1 | -0.2 | 1.7 | | 0.9 | 0.2 | 0.7 |
| Lesotho | 1.1 | 0.2 | 1.5 | -0.2 | -0.7 | 2.0 | 4.2 | 0.1 | 0.2 | 1.1 | 0.2 | 0.8 | | 0.8 | 0.1 | 0.7 |
| Liberia | 5.0 | 1.6 | 2.9 | 4.4 | 1.1 | 2.0 | 1.6 | 0.7 | 2.6 | -0.3 | -0.9 | 3.2 | | 0.0 | -0.4 | 1.6 |
| Madagascar | 3.5 | 1.6 | 4.1 | 4.4 | 1.2 | 2.1 | 5.0 | 0.7 | 1.0 | 2.6 | 0.4 | 0.6 | | 1.2 | 0.2 | 0.5 |
| Malawi | 1.3 | 0.4 | 0.8 | 0.9 | 0.3 | 0.7 | 0.8 | 0.3 | 0.8 | 0.7 | 0.2 | 0.5 | | 0.4 | 0.1 | 0.5 |
| Maldives | -0.6 | -5.6 | 1.8 | -0.5 | -6.0 | 1.5 | -0.5 | -2.9 | 1.7 | 0.0 | -0.3 | 0.9 | | -0.5 | -2.6 | 1.6 |
| Mali | 3.6 | 1.2 | 2.5 | 3.5 | 1.1 | 2.2 | 2.7 | 1.0 | 2.5 | 2.6 | 0.8 | 1.8 | | 0.3 | 0.1 | 1.8 |
| Morocco | 2.1 | 0.3 | 0.5 | 1.2 | 0.2 | 0.5 | 0.5 | 0.1 | 0.5 | 0.6 | 0.1 | 0.1 | | 0.3 | 0.0 | 0.1 |
| Mozambique | 4.2 | 1.0 | 1.7 | 3.0 | 0.9 | 1.7 | 2.8 | 0.6 | 1.1 | 1.0 | 0.2 | 0.4 | | 0.1 | 0.0 | 0.4 |
| Namibia | 1.6 | 0.4 | 0.7 | 1.0 | 0.3 | 0.6 | 0.6 | 0.3 | 1.2 | 1.0 | 0.3 | 0.7 | | 0.7 | 0.2 | 0.6 |
| Nepal | 4.8 | 0.3 | 0.4 | 2.9 | 0.4 | 0.6 | 1.9 | 0.5 | 1.0 | 2.1 | 0.2 | 0.3 | | 0.9 | 0.2 | 0.4 |
| Nicaragua | -5.1 | -3.4 | 14.4 | -2.1 | -2.3 | 6.4 | -7.0 | -5.0 | 18.8 | -15.6 | -7.5 | 40.9 | | -19.5 | -8.9 | 51.1 |
| Niger | 6.8 | 1.7 | 2.8 | 6.8 | 1.3 | 1.9 | 7.2 | 1.3 | 1.9 | 5.1 | 1.1 | 1.9 | | 1.0 | 0.5 | 2.1 |
| Nigeria | -2.0 | -0.4 | 18.3 | 1.2 | 1.6 | 10.7 | 3.0 | 1.9 | 5.9 | 3.6 | 1.3 | 3.0 | | 0.9 | 0.4 | 2.0 |
| Pakistan | 2.2 | 1.7 | 7.2 | 2.2 | 1.2 | 3.7 | 1.6 | 0.9 | 2.8 | 2.0 | 0.8 | 1.7 | | 0.7 | 0.4 | 1.4 |
| Peru | 0.1 | 0.0 | 3.2 | -0.2 | -0.3 | 3.0 | -0.9 | -1.4 | 4.4 | -1.2 | -2.3 | 4.5 | | -0.9 | -1.7 | 3.7 |
| Philippines | 0.1 | 0.1 | 3.1 | 0.3 | 0.2 | 1.4 | -0.2 | -0.3 | 1.7 | 0.3 | 0.2 | 1.0 | | 0.0 | -0.1 | 1.1 |
| Rwanda | -0.7 | -1.3 | 3.0 | -0.6 | -1.0 | 2.7 | -0.6 | -1.4 | 2.6 | -0.1 | -0.4 | 1.6 | | -0.4 | -0.9 | 1.9 |
| Senegal | 0.7 | 0.4 | 2.5 | 1.3 | 0.5 | 1.8 | 1.9 | 0.6 | 1.4 | 1.6 | 0.5 | 1.2 | | 3.5 | 0.3 | 0.5 |
| Sierra Leone | 11.0 | 0.5 | 0.6 | 5.8 | 0.7 | 1.2 | 6.8 | 0.7 | 1.2 | 8.2 | 0.6 | 0.9 | | 3.9 | 0.6 | 1.3 |
| South Africa | 0.7 | 0.4 | 1.6 | 0.0 | -0.1 | 1.7 | 0.1 | 0.0 | 1.2 | 0.1 | 0.0 | 1.3 | | 0.0 | -0.1 | 1.6 |
| Tajikistan | 1.0 | 0.0 | 1.8 | 0.4 | -0.5 | 1.4 | -0.1 | -1.3 | 2.1 | -0.4 | -2.1 | 2.3 | | -0.2 | -1.4 | 2.5 |
| Tanzania | 1.0 | 0.5 | 2.1 | -0.1 | -0.2 | 2.1 | 1.0 | 0.4 | 1.1 | 0.2 | 0.1 | 0.7 | | 0.0 | 0.0 | 0.7 |
| Timor-Leste | 0.4 | 0.2 | 5.3 | -0.2 | -0.6 | 4.5 | 1.0 | 0.2 | 2.6 | 0.1 | -0.6 | 2.6 | | 1.0 | 0.1 | 2.2 |
| Togo | 5.3 | 1.1 | 1.7 | 3.9 | 1.3 | 2.6 | 2.5 | 1.1 | 2.9 | 4.5 | 0.9 | 1.5 | | 2.0 | 0.6 | 1.2 |
| Turkey | 1.6 | 0.7 | 1.9 | 1.1 | 0.4 | 0.9 | 0.8 | 0.3 | 0.7 | -0.1 | -0.2 | 1.1 | | 0.4 | 0.2 | 0.6 |
| Uganda | 2.8 | 0.9 | 1.9 | 1.9 | 0.9 | 2.2 | 2.0 | 0.7 | 1.7 | 1.5 | 0.7 | 1.9 | | 0.8 | 0.4 | 1.7 |
| Vietnam | -0.1 | -0.3 | 4.5 | 1.6 | 0.1 | 0.8 | 1.3 | 0.0 | 0.5 | 0.7 | 0.0 | 0.6 | | 3.6 | 0.0 | 0.0 |
| Zambia | 0.1 | 0.0 | 1.6 | -0.4 | -0.6 | 2.3 | -0.3 | -0.5 | 2.0 | 0.1 | 0.0 | 1.1 | | 0.2 | 0.1 | 0.6 |
| Zimbabwe | -0.6 | -0.9 | 3.5 | -0.6 | -0.9 | 3.4 | -0.3 | -0.5 | 2.4 | 0.3 | 0.1 | 1.7 | | -0.3 | -0.5 | 2.0 |

**Note:** AARC is calculated as ln((rate in latest year/rate in earliest year))/ (latest year - earliest year), with positive values denote increasing rate while negative values denote decreasing rate

Table S57: Predicted average annual rate of increase (AARC) of Measles immunization among children 12-23 months of age in low- and middle-income countries by place of residence

| **Country** | **Rural** | | | **Urban** | | |
| --- | --- | --- | --- | --- | --- | --- |
|  | **Predicted** | | **Required** | **Predicted** | | **Required** |
|  | **1990-2018** | **2019-2030** | **2019-2030** | **1990-2018** | **2019-2030** | **2019-2030** |
| Albania | 40.4 | 0.0 | 0.0 | 44.3 | 0.0 | 0.0 |
| Armenia | 1.0 | 0.4 | 1.2 | 1.7 | 0.4 | 0.9 |
| Bangladesh | 1.5 | 0.5 | 1.0 | 0.7 | 0.3 | 0.8 |
| Benin | 0.2 | 0.1 | 3.9 | 0.2 | 0.2 | 2.8 |
| Bolivia | 3.5 | 0.7 | 1.2 | 0.8 | 0.4 | 2.9 |
| Brazil | 0.5 | 0.0 | 1.9 | 0.9 | 0.0 | 0.1 |
| Burkina Faso | 3.7 | 0.6 | 0.9 | 1.3 | 0.3 | 0.7 |
| Burundi | 0.1 | -0.3 | 0.6 | -0.2 | -1.0 | 0.9 |
| Cambodia | 2.3 | 0.6 | 1.4 | 3.8 | 0.3 | 0.5 |
| Cameroon | 1.5 | 0.9 | 3.3 | 1.3 | 0.5 | 1.5 |
| Chad | 6.8 | 2.5 | 4.5 | 3.3 | 1.5 | 3.7 |
| Colombia | -0.9 | -1.2 | 4.7 | 0.1 | -0.1 | 2.7 |
| Comoros | 1.0 | 0.5 | 2.1 | 1.1 | 0.5 | 2.2 |
| Congo | 1.1 | 0.5 | 4.0 | 1.7 | 0.3 | 1.2 |
| Congo DR | 3.3 | 1.0 | 2.6 | 1.0 | 0.2 | 2.2 |
| Cote d'Ivoire | 0.9 | 0.6 | 4.4 | 0.4 | 0.1 | 2.2 |
| Dominican Republic | 1.0 | 0.4 | 1.2 | 0.6 | 0.2 | 1.0 |
| Egypt | -0.2 | -0.5 | 1.4 | -0.5 | -1.3 | 1.7 |
| Ethiopia | 5.6 | 2.3 | 4.4 | 1.8 | 0.7 | 1.8 |
| Gabon | 6.7 | 0.8 | 1.0 | 1.6 | 0.6 | 2.2 |
| Ghana | 2.2 | 0.3 | 0.5 | 0.8 | 0.2 | 0.6 |
| Guatemala | -1.0 | -1.6 | 4.7 | -0.9 | -1.5 | 4.2 |
| Guinea | 1.6 | 1.0 | 4.4 | 1.0 | 0.4 | 2.8 |
| Haiti | 1.4 | 0.9 | 4.1 | 0.6 | 0.4 | 3.6 |
| Honduras | 0.8 | 0.0 | 0.9 | 0.6 | -0.1 | 1.3 |
| India | 3.8 | 1.0 | 1.8 | 1.7 | 0.6 | 1.5 |
| Indonesia | 1.6 | 0.7 | 1.8 | 0.4 | 0.3 | 1.7 |
| Jordan | 0.0 | -0.1 | 1.0 | -0.1 | -0.1 | 1.0 |
| Kazakhstan | 2.3 | 0.1 | 0.7 | 2.2 | 0.1 | 0.5 |
| Kenya | 0.5 | 0.3 | 1.5 | 0.3 | 0.1 | 0.7 |
| Kyrgyz Republic | 0.4 | 0.1 | 0.9 | 0.5 | 0.2 | 0.9 |
| Lesotho | 1.0 | 0.2 | 1.1 | 0.3 | 0.0 | 0.7 |
| Liberia | 2.3 | 1.0 | 2.9 | 0.0 | -0.2 | 2.5 |
| Madagascar | 2.1 | 0.9 | 2.7 | 2.0 | 0.4 | 0.8 |
| Malawi | 0.9 | 0.3 | 0.7 | 0.4 | 0.2 | 0.6 |
| Maldives | -0.6 | -3.9 | 1.7 | -0.2 | -0.8 | 1.0 |
| Mali | 3.1 | 1.0 | 2.3 | 1.0 | 0.5 | 1.6 |
| Morocco | 1.3 | 0.2 | 0.5 | 0.3 | 0.1 | 0.3 |
| Mozambique | 2.7 | 0.7 | 1.4 | 0.0 | 0.0 | 0.8 |
| Namibia | 1.3 | 0.3 | 0.6 | 0.6 | 0.3 | 1.1 |
| Nepal | 2.5 | 0.4 | 0.7 | 1.0 | 0.3 | 0.6 |
| Nicaragua | -5.2 | -3.9 | 14.5 | -9.1 | -6.0 | 24.2 |
| Niger | 5.8 | 1.5 | 2.3 | 1.2 | 0.6 | 1.8 |
| Nigeria | -0.6 | -0.5 | 11.0 | -0.1 | -0.2 | 5.0 |
| Pakistan | 2.0 | 1.1 | 3.7 | 0.9 | 0.6 | 2.3 |
| Peru | 0.2 | 0.1 | 2.8 | -0.8 | -1.3 | 4.0 |
| Philippines | 0.2 | 0.1 | 2.0 | 0.1 | 0.1 | 1.5 |
| Rwanda | -0.6 | -1.1 | 2.5 | -0.3 | -0.7 | 1.8 |
| Senegal | 2.2 | 0.8 | 1.6 | 1.3 | 0.5 | 1.2 |
| Sierra Leone | 8.4 | 0.6 | 0.9 | 5.0 | 0.6 | 1.2 |
| South Africa | 0.4 | 0.2 | 1.5 | 0.0 | -0.1 | 1.5 |
| Tajikistan | 0.3 | -0.6 | 1.8 | -0.6 | -2.8 | 2.7 |
| Tanzania | 0.5 | 0.3 | 1.6 | 0.1 | 0.1 | 0.7 |
| Timor-Leste | 0.5 | -0.1 | 3.6 | 0.2 | -0.4 | 2.8 |
| Togo | 4.1 | 1.2 | 2.2 | 2.1 | 0.7 | 1.7 |
| Turkey | 0.9 | 0.5 | 1.6 | 0.6 | 0.3 | 0.9 |
| Uganda | 1.9 | 0.8 | 2.0 | 0.8 | 0.4 | 1.6 |
| Vietnam | 1.1 | 0.1 | 1.2 | 2.0 | 0.0 | 0.1 |
| Yemen | 2.0 | 1.1 | 3.9 | 0.3 | 0.2 | 2.6 |
| Zambia | 0.6 | 0.3 | 1.3 | 0.4 | 0.2 | 0.9 |
| Zimbabwe | -0.4 | -0.6 | 2.9 | -0.2 | -0.3 | 1.9 |

**Note:** AARC is calculated as ln((rate in latest year/rate in earliest year))/ (latest year - earliest year), with positive values denote increasing rate while negative values denote decreasing rate

Table S58: Predicted average annual rate of increase (AARC) of Measles immunization among children 12-23 months of age in low- and middle-income countries by education of mother

| **Country** | **Below secondary education** | | | **Secondary+ education** | | |
| --- | --- | --- | --- | --- | --- | --- |
|  | **Predicted** | | **Required** | **Predicted** | | **Required** |
|  | **1990-2018** | **2019-2030** | **2019-2030** | **1990-2018** | **2019-2030** | **2019-2030** |
| Albania | 41.7 | 0.0 | 0.0 | 42.3 | 0.0 | 0.0 |
| Armenia | -0.1 | -5.7 | 0.3 | 1.2 | 0.4 | 1.2 |
| Bangladesh | 1.1 | 0.6 | 1.7 | 0.4 | 0.2 | 0.6 |
| Benin | 0.2 | 0.1 | 3.9 | -0.8 | -2.5 | 2.5 |
| Bolivia | 3.1 | 0.8 | 1.5 | 0.8 | 0.3 | 2.5 |
| Brazil | 1.0 | 0.0 | 0.6 | -0.5 | -0.7 | 2.2 |
| Burkina Faso | 3.4 | 0.5 | 0.9 | 0.3 | 0.0 | 0.7 |
| Burundi | 0.1 | -0.4 | 0.7 | -0.2 | -2.4 | 0.6 |
| Cambodia | 2.2 | 0.7 | 1.7 | 1.3 | 0.3 | 0.7 |
| Cameroon | 1.6 | 0.9 | 3.3 | 0.8 | 0.4 | 1.2 |
| Chad | 5.8 | 2.4 | 4.7 | 2.1 | 0.9 | 2.5 |
| Colombia | -0.7 | -0.8 | 4.7 | 0.0 | -0.2 | 2.5 |
| Comoros | 1.0 | 0.5 | 2.4 | 0.3 | 0.1 | 2.1 |
| Congo | 1.7 | 0.7 | 3.4 | 2.0 | 0.4 | 1.2 |
| Congo DR | 2.9 | 1.1 | 3.0 | 1.0 | 0.2 | 1.8 |
| Cote d'Ivoire | 0.8 | 0.5 | 3.8 | 0.1 | -0.1 | 1.1 |
| Dominican Republic | 1.1 | 0.4 | 1.2 | 0.3 | 0.1 | 1.1 |
| Egypt | -0.3 | -0.7 | 1.8 | -0.6 | -2.2 | 2.0 |
| Ethiopia | 5.1 | 2.2 | 4.3 | 2.6 | 0.6 | 1.2 |
| Gabon | 4.5 | 0.9 | 1.6 | 1.4 | 0.6 | 2.1 |
| Ghana | 2.0 | 0.4 | 0.7 | 0.1 | 0.0 | 0.8 |
| Guatemala | -1.1 | -1.6 | 5.0 | -1.2 | -3.3 | 3.8 |
| Guinea | 1.3 | 0.8 | 4.4 | 0.4 | 0.1 | 1.8 |
| Haiti | 1.0 | 0.7 | 4.7 | 0.3 | 0.2 | 3.2 |
| Honduras | 0.9 | 0.0 | 1.0 | 0.3 | -0.3 | 1.4 |
| India | 4.0 | 1.3 | 2.6 | 1.0 | 0.4 | 1.2 |
| Indonesia | 1.1 | 0.7 | 2.9 | 0.3 | 0.2 | 1.5 |
| Jordan | -0.6 | -1.1 | 2.7 | -0.1 | -0.1 | 0.9 |
| Kazakhstan | N/A | N/A | N/A | 2.3 | 0.1 | 0.6 |
| Kenya | 0.6 | 0.3 | 1.6 | 0.3 | 0.1 | 0.6 |
| Kyrgyz Republic | -2.0 | -34.4 | 6.7 | 0.4 | 0.1 | 0.9 |
| Lesotho | 0.4 | -0.1 | 1.6 | 1.4 | 0.3 | 0.6 |
| Liberia | 2.0 | 0.9 | 2.9 | 0.2 | -0.4 | 2.1 |
| Madagascar | 2.0 | 1.0 | 3.2 | 1.9 | 0.3 | 0.5 |
| Malawi | 0.8 | 0.3 | 0.8 | -0.1 | -0.1 | 0.6 |
| Maldives | -0.5 | -3.2 | 1.7 | -0.5 | -2.7 | 1.4 |
| Mali | 2.5 | 1.0 | 2.3 | 0.4 | 0.2 | 1.0 |
| Morocco | 1.1 | 0.2 | 0.4 | 0.3 | 0.1 | 0.2 |
| Mozambique | 1.9 | 0.6 | 1.4 | -0.1 | -0.4 | 0.6 |
| Namibia | 1.0 | 0.4 | 1.2 | 0.6 | 0.2 | 0.7 |
| Nepal | 2.6 | 0.5 | 0.9 | 0.2 | 0.1 | 0.4 |
| Nicaragua | -3.5 | -3.0 | 10.2 | -18.4 | -9.2 | 48.3 |
| Niger | 4.7 | 1.4 | 2.5 | 0.7 | 0.3 | 1.0 |
| Nigeria | -1.5 | -1.4 | 13.3 | -0.2 | -0.3 | 3.5 |
| Pakistan | 1.3 | 0.9 | 4.5 | 0.7 | 0.4 | 1.4 |
| Peru | 0.0 | -0.1 | 3.2 | -0.9 | -1.5 | 4.0 |
| Philippines | -0.3 | -0.4 | 3.7 | 0.0 | 0.0 | 1.4 |
| Rwanda | -0.6 | -1.1 | 2.6 | -0.6 | -1.8 | 1.8 |
| Senegal | 1.8 | 0.7 | 1.6 | 0.3 | 0.1 | 0.5 |
| Sierra Leone | 7.7 | 0.7 | 1.0 | 3.8 | 0.4 | 0.9 |
| South Africa | -0.5 | -0.7 | 3.5 | 0.0 | 0.0 | 1.4 |
| Tajikistan | 4.1 | 0.5 | 0.9 | -0.2 | -1.5 | 2.2 |
| Tanzania | 0.4 | 0.3 | 1.5 | -0.1 | -0.2 | 0.7 |
| Timor-Leste | 0.6 | 0.1 | 4.0 | -0.3 | -1.2 | 3.2 |
| Togo | 3.6 | 1.2 | 2.3 | 2.2 | 0.5 | 1.0 |
| Turkey | 0.9 | 0.4 | 1.2 | -0.1 | -0.1 | 1.1 |
| Uganda | 1.8 | 0.8 | 2.2 | 0.4 | 0.2 | 1.5 |
| Vietnam | -0.7 | -0.6 | 5.7 | 1.8 | 0.0 | 0.1 |
| Yemen | 1.6 | 1.0 | 3.8 | -0.2 | -0.3 | 2.7 |
| Zambia | 0.5 | 0.3 | 1.3 | 0.0 | -0.1 | 1.0 |
| Zimbabwe | -0.5 | -0.8 | 3.7 | -0.6 | -1.0 | 2.5 |

**Note:** AARC is calculated as ln((rate in latest year/rate in earliest year))/ (latest year - earliest year), with positive values denote increasing rate while negative values denote decreasing rate

Table S59: Predicted average annual rate of increase (AARC) of Measles immunization among children 12-23 months of age in low- and middle-income countries by age of mother

| **Country** | **Adolescent mother** | | | **Adult mother** | | |
| --- | --- | --- | --- | --- | --- | --- |
|  | **Predicted** | | **Required** | **Predicted** | | **Required** |
|  | **1990-2018** | **2019-2030** | **2019-2030** | **1990-2018** | **2019-2030** | **2019-2030** |
| Albania | 40.3 | 0.0 | 0.0 | 41.1 | 0.0 | 0.0 |
| Armenia | 25.3 | 0.0 | 0.0 | 1.2 | 0.4 | 1.1 |
| Bangladesh | 1.5 | 0.5 | 0.9 | 1.4 | 0.4 | 0.9 |
| Benin | -0.3 | -0.4 | 4.9 | 0.3 | 0.2 | 3.4 |
| Bolivia | 2.8 | 0.7 | 1.7 | 2.0 | 0.7 | 1.9 |
| Brazil | -0.6 | -0.7 | 3.2 | 1.1 | 0.0 | 0.2 |
| Burkina Faso | 5.4 | 0.5 | 0.8 | 3.2 | 0.5 | 0.9 |
| Burundi | -0.3 | -1.4 | 1.2 | 0.1 | -0.3 | 0.6 |
| Cambodia | 1.2 | 0.5 | 2.0 | 2.5 | 0.6 | 1.2 |
| Cameroon | 1.1 | 0.7 | 3.8 | 1.5 | 0.7 | 2.4 |
| Chad | 5.2 | 2.2 | 4.7 | 5.8 | 2.3 | 4.4 |
| Colombia | -0.4 | -0.5 | 3.5 | -0.2 | -0.4 | 3.2 |
| Comoros | -0.7 | -0.9 | 5.8 | 1.1 | 0.5 | 2.0 |
| Congo | 1.2 | 0.4 | 2.7 | 2.1 | 0.6 | 1.9 |
| Congo DR | 2.3 | 0.8 | 2.5 | 2.1 | 0.7 | 2.5 |
| Cote d'Ivoire | 1.5 | 0.8 | 3.3 | 0.6 | 0.3 | 3.5 |
| Dominican Republic | 1.1 | 0.4 | 1.6 | 0.8 | 0.3 | 1.0 |
| Egypt | -0.1 | -0.3 | 1.1 | -0.3 | -0.7 | 1.5 |
| Ethiopia | 4.9 | 2.1 | 4.3 | 5.0 | 2.0 | 4.0 |
| Gabon | 1.6 | 0.9 | 4.3 | 2.9 | 0.7 | 1.5 |
| Ghana | 1.3 | 0.5 | 1.4 | 1.7 | 0.3 | 0.5 |
| Guatemala | -1.3 | -2.1 | 5.2 | -0.9 | -1.4 | 4.4 |
| Guinea | 1.2 | 0.7 | 4.0 | 1.4 | 0.8 | 4.0 |
| Haiti | 0.8 | 0.6 | 4.5 | 1.1 | 0.8 | 3.9 |
| Honduras | 0.8 | 0.0 | 1.2 | 0.7 | 0.0 | 1.1 |
| India | 3.7 | 1.0 | 1.9 | 3.2 | 0.9 | 1.7 |
| Indonesia | 1.4 | 0.7 | 2.1 | 1.2 | 0.6 | 1.7 |
| Jordan | -0.1 | -0.2 | 1.1 | -0.1 | -0.1 | 1.0 |
| Kazakhstan | -26.0 | -11.3 | 68.2 | 2.8 | 0.1 | 0.3 |
| Kenya | 0.7 | 0.3 | 1.1 | 0.5 | 0.3 | 1.3 |
| Kyrgyz Republic | 1.6 | 0.1 | 0.2 | 0.4 | 0.1 | 0.9 |
| Lesotho | 0.2 | -0.3 | 1.1 | 1.1 | 0.2 | 1.0 |
| Liberia | -0.6 | -1.1 | 4.1 | 2.2 | 0.8 | 2.3 |
| Madagascar | 1.6 | 0.8 | 4.0 | 2.1 | 0.8 | 2.2 |
| Malawi | 0.6 | 0.3 | 0.8 | 0.8 | 0.3 | 0.7 |
| Maldives | -4.9 | -110.4 | 21.0 | -0.5 | -2.7 | 1.5 |
| Mali | 1.9 | 0.9 | 2.6 | 2.5 | 0.9 | 2.1 |
| Morocco | 1.2 | 0.1 | 0.3 | 1.1 | 0.2 | 0.3 |
| Mozambique | 0.9 | 0.4 | 1.2 | 2.0 | 0.6 | 1.3 |
| Namibia | 1.1 | 0.3 | 0.6 | 0.9 | 0.3 | 0.8 |
| Nepal | 2.1 | 0.3 | 0.6 | 2.6 | 0.4 | 0.6 |
| Nicaragua | -5.7 | -3.9 | 15.7 | -7.3 | -5.4 | 19.7 |
| Niger | 4.2 | 1.5 | 3.1 | 4.5 | 1.3 | 2.2 |
| Nigeria | -1.2 | -0.8 | 14.8 | 0.0 | 0.0 | 7.9 |
| Pakistan | 2.2 | 1.3 | 4.3 | 1.5 | 0.9 | 3.2 |
| Peru | -0.4 | -0.5 | 4.3 | -0.4 | -0.6 | 3.4 |
| Philippines | 0.5 | 0.3 | 2.1 | 0.1 | 0.1 | 1.7 |
| Rwanda | -1.3 | -2.8 | 4.3 | -0.5 | -1.0 | 2.4 |
| Senegal | 2.3 | 0.9 | 1.9 | 1.8 | 0.6 | 1.4 |
| Sierra Leone | 12.4 | 0.4 | 0.5 | 7.0 | 0.6 | 1.0 |
| South Africa | -0.4 | -0.7 | 2.9 | 0.2 | 0.1 | 1.4 |
| Tajikistan | -1.3 | -10.3 | 4.0 | 0.0 | -1.0 | 2.0 |
| Tanzania | 0.3 | 0.1 | 1.3 | 0.5 | 0.3 | 1.3 |
| Timor-Leste | 0.8 | 0.4 | 4.5 | 0.5 | -0.1 | 3.3 |
| Togo | 6.4 | 1.0 | 1.4 | 3.5 | 1.0 | 2.0 |
| Turkey | -0.6 | -0.9 | 3.4 | 0.9 | 0.4 | 1.0 |
| Uganda | 2.8 | 0.8 | 1.6 | 1.7 | 0.7 | 1.9 |
| Vietnam | -9.8 | -8.6 | 26.6 | 1.3 | 0.1 | 0.8 |
| Yemen | 2.4 | 1.1 | 3.0 | 1.7 | 0.9 | 3.5 |
| Zambia | 0.8 | 0.3 | 0.7 | 0.4 | 0.2 | 1.2 |
| Zimbabwe | -0.4 | -0.6 | 3.1 | -0.3 | -0.5 | 2.6 |

**Note:** AARC is calculated as ln((rate in latest year/rate in earliest year))/ (latest year - earliest year), with positive values denote increasing rate while negative values denote decreasing rate

Table S60: Predicted average annual rate of increase (AARC) of Measles immunization among children 12-23 months of age in low- and middle-income countries by child sex

| **Country** | **Male** | | | **Female** | | |
| --- | --- | --- | --- | --- | --- | --- |
|  | **Predicted** | | **Required** | **Predicted** | | **Required** |
|  | **1990-2018** | **2019-2030** | **2019-2030** | **1990-2018** | **2019-2030** | **2019-2030** |
| Albania | 43.4 | 0.0 | 0.0 | 39.5 | 0.0 | 0.0 |
| Armenia | 1.6 | 0.5 | 1.1 | 1.0 | 0.3 | 1.0 |
| Bangladesh | 1.2 | 0.4 | 1.0 | 1.7 | 0.4 | 0.9 |
| Benin | 0.4 | 0.3 | 3.3 | 0.1 | 0.0 | 3.7 |
| Bolivia | 1.5 | 0.6 | 2.2 | 2.5 | 0.8 | 1.7 |
| Brazil | 1.4 | 0.0 | 0.1 | 0.5 | 0.0 | 0.8 |
| Burkina Faso | 3.5 | 0.5 | 0.9 | 3.2 | 0.5 | 0.9 |
| Burundi | -0.1 | -0.6 | 0.7 | 0.2 | -0.1 | 0.6 |
| Cambodia | 2.2 | 0.6 | 1.3 | 2.8 | 0.6 | 1.2 |
| Cameroon | 1.3 | 0.7 | 2.6 | 1.7 | 0.8 | 2.3 |
| Chad | 5.2 | 2.2 | 4.7 | 6.3 | 2.3 | 4.2 |
| Colombia | -0.2 | -0.3 | 3.1 | -0.2 | -0.4 | 3.5 |
| Comoros | 0.9 | 0.4 | 2.6 | 1.2 | 0.5 | 1.7 |
| Congo | 2.5 | 0.6 | 1.9 | 1.5 | 0.4 | 2.1 |
| Congo DR | 2.7 | 0.8 | 2.3 | 1.9 | 0.7 | 2.6 |
| Cote d'Ivoire | 0.8 | 0.5 | 3.3 | 0.6 | 0.3 | 3.7 |
| Dominican Republic | 1.1 | 0.3 | 0.8 | 0.5 | 0.2 | 1.2 |
| Egypt | -0.3 | -0.7 | 1.5 | -0.3 | -0.7 | 1.5 |
| Ethiopia | 4.5 | 2.0 | 4.3 | 5.6 | 2.1 | 3.8 |
| Gabon | 2.9 | 0.8 | 1.6 | 2.4 | 0.8 | 2.1 |
| Ghana | 1.7 | 0.3 | 0.6 | 1.7 | 0.3 | 0.5 |
| Guatemala | -1.0 | -1.6 | 4.5 | -1.0 | -1.5 | 4.6 |
| Guinea | 1.4 | 0.8 | 3.8 | 1.3 | 0.7 | 4.2 |
| Haiti | 1.4 | 0.9 | 3.8 | 0.8 | 0.6 | 4.1 |
| Honduras | 0.6 | -0.1 | 1.1 | 0.8 | 0.0 | 1.1 |
| India | 3.2 | 0.9 | 1.6 | 3.4 | 1.0 | 1.8 |
| Indonesia | 1.3 | 0.6 | 1.7 | 1.2 | 0.6 | 1.7 |
| Jordan | -0.1 | -0.1 | 1.0 | -0.1 | -0.2 | 1.0 |
| Kazakhstan | 3.2 | 0.1 | 0.3 | 1.5 | 0.1 | 1.1 |
| Kenya | 0.6 | 0.3 | 1.3 | 0.6 | 0.3 | 1.3 |
| Kyrgyz Republic | 0.5 | 0.2 | 0.8 | 0.3 | 0.1 | 1.1 |
| Lesotho | 0.5 | 0.0 | 1.4 | 1.8 | 0.3 | 0.6 |
| Liberia | 1.9 | 0.8 | 2.6 | 1.9 | 0.7 | 2.3 |
| Madagascar | 1.6 | 0.8 | 3.1 | 2.3 | 0.8 | 2.0 |
| Malawi | 0.7 | 0.3 | 0.8 | 0.9 | 0.3 | 0.6 |
| Maldives | -0.5 | -3.1 | 1.5 | -0.4 | -2.4 | 1.4 |
| Mali | 2.0 | 0.8 | 2.3 | 2.8 | 1.0 | 2.1 |
| Morocco | 1.0 | 0.2 | 0.5 | 1.1 | 0.1 | 0.2 |
| Mozambique | 1.8 | 0.6 | 1.2 | 2.0 | 0.6 | 1.2 |
| Namibia | 1.0 | 0.3 | 0.7 | 0.8 | 0.3 | 0.9 |
| Nepal | 2.3 | 0.3 | 0.5 | 2.7 | 0.4 | 0.7 |
| Nicaragua | -5.1 | -4.3 | 14.0 | -9.5 | -5.7 | 25.2 |
| Niger | 4.7 | 1.3 | 2.3 | 4.2 | 1.3 | 2.5 |
| Nigeria | 0.1 | 0.1 | 8.1 | -0.2 | -0.1 | 8.3 |
| Pakistan | 1.5 | 0.9 | 2.9 | 1.7 | 1.0 | 3.6 |
| Peru | -0.5 | -0.6 | 3.7 | -0.5 | -0.7 | 3.4 |
| Philippines | 0.2 | 0.1 | 1.7 | 0.1 | 0.0 | 1.8 |
| Rwanda | -0.5 | -1.0 | 2.5 | -0.5 | -1.0 | 2.4 |
| Senegal | 1.9 | 0.7 | 1.5 | 1.8 | 0.6 | 1.4 |
| Sierra Leone | 7.5 | 0.6 | 1.0 | 7.2 | 0.6 | 0.9 |
| South Africa | 0.3 | 0.1 | 1.2 | 0.1 | 0.0 | 1.8 |
| Tajikistan | -0.2 | -1.4 | 2.1 | 0.3 | -0.5 | 1.8 |
| Tanzania | 0.5 | 0.3 | 1.2 | 0.4 | 0.2 | 1.5 |
| Timor-Leste | 0.1 | -0.5 | 3.5 | 1.0 | 0.3 | 3.1 |
| Togo | 3.5 | 1.0 | 1.8 | 3.9 | 1.1 | 2.1 |
| Turkey | 0.8 | 0.4 | 1.1 | 0.9 | 0.4 | 1.0 |
| Uganda | 1.8 | 0.7 | 1.8 | 1.7 | 0.7 | 1.9 |
| Vietnam | 1.5 | 0.1 | 0.7 | 0.8 | 0.0 | 1.4 |
| Yemen | 1.6 | 0.9 | 3.6 | 1.7 | 1.0 | 3.6 |
| Zambia | 0.5 | 0.2 | 1.2 | 0.5 | 0.2 | 1.1 |
| Zimbabwe | -0.4 | -0.6 | 2.9 | -0.3 | -0.4 | 2.4 |

**Note:** AARC is calculated as ln((rate in latest year/rate in earliest year))/ (latest year - earliest year), with positive values denote increasing rate while negative values denote decreasing rate

Table S61: Predicted average annual rate of increase (AARC) of oral rehydration therapy for diarrhoea treatment among children 0-59 months of age in low- and middle-income countries

| **Country** | **Predicted** | | **Required** |
| --- | --- | --- | --- |
|  | **1990-2018** | **2019-2030** | **2019-2030** |
| Albania | -2.7 | -6.9 | 8.1 |
| Armenia | 3.1 | 1.4 | 3.8 |
| Bangladesh | 1.8 | 0.5 | 1.1 |
| Benin | 0.2 | 0.3 | 9.4 |
| Bolivia | 0.3 | 0.3 | 7.2 |
| Brazil | 1.2 | 0.3 | 3.8 |
| Burkina Faso | 2.9 | 2.3 | 9.7 |
| Burundi | -1.0 | -0.6 | 9.0 |
| Cambodia | 3.3 | 2.3 | 7.3 |
| Cameroon | -2.1 | -2.0 | 15.0 |
| Chad | 0.5 | 0.8 | 11.7 |
| Colombia | 1.8 | 1.0 | 4.0 |
| Comoros | 4.0 | 1.4 | 2.9 |
| Congo | 3.9 | 2.4 | 6.7 |
| Congo DR | -0.9 | -0.6 | 8.2 |
| Cote d'Ivoire | 0.7 | 0.9 | 12.1 |
| Dominican Republic | 2.0 | 1.3 | 5.1 |
| Egypt | -1.1 | -1.1 | 11.4 |
| Ethiopia | 4.2 | 2.8 | 7.8 |
| Gabon | 0.3 | 0.5 | 8.5 |
| Ghana | 2.2 | 1.3 | 4.7 |
| Guatemala | 0.8 | 0.6 | 5.9 |
| Guinea | -0.8 | -0.6 | 9.8 |
| Haiti | 1.7 | 1.2 | 5.7 |
| Honduras | 1.0 | 0.4 | 4.3 |
| India | 2.9 | 1.8 | 5.8 |
| Indonesia | -0.6 | -0.6 | 7.5 |
| Jordan | 0.5 | 0.5 | 9.7 |
| Kazakhstan | 0.8 | 0.6 | 8.2 |
| Kenya | 2.4 | 1.0 | 3.0 |
| Kyrgyz Republic | 3.1 | 1.0 | 2.2 |
| Lesotho | 0.0 | -0.2 | 2.9 |
| Liberia | 0.8 | 0.3 | 4.1 |
| Madagascar | 2.0 | 1.5 | 7.6 |
| Malawi | 0.9 | 0.6 | 3.2 |
| Maldives | 6.0 | 0.9 | 1.3 |
| Mali | 4.5 | 2.5 | 6.5 |
| Morocco | 4.4 | 2.3 | 6.2 |
| Mozambique | 0.7 | 0.5 | 4.8 |
| Namibia | 1.0 | 0.5 | 2.2 |
| Nepal | 4.1 | 2.0 | 4.7 |
| Nicaragua | -2.0 | -0.8 | 8.9 |
| Niger | 4.2 | 2.3 | 5.7 |
| Nigeria | 1.4 | 1.2 | 8.5 |
| Pakistan | -0.2 | -0.1 | 7.9 |
| Peru | 1.8 | 1.4 | 7.4 |
| Philippines | 0.5 | 0.4 | 5.2 |
| Rwanda | 0.5 | 0.7 | 10.7 |
| Senegal | 0.9 | 0.9 | 11.4 |
| Sierra Leone | 5.5 | 0.3 | 0.6 |
| South Africa | -0.1 | -0.2 | 2.0 |
| Tajikistan | -0.7 | -2.0 | 4.1 |
| Tanzania | -0.5 | -0.5 | 5.8 |
| Timor-Leste | 0.0 | -0.6 | 2.5 |
| Togo | -1.0 | -0.4 | 15.0 |
| Turkey | 3.0 | 2.1 | 8.0 |
| Uganda | 0.2 | 0.2 | 6.7 |
| Vietnam | -3.2 | -1.7 | 12.7 |
| Yemen | -0.7 | -0.6 | 11.8 |
| Zambia | 0.8 | 0.5 | 3.3 |
| Zimbabwe | -0.8 | -1.1 | 4.5 |

**Note:** AARC is calculated as ln((rate in latest year/rate in earliest year))/ (latest year - earliest year), with positive values denote increasing rate while negative values denote decreasing rate
[truncated: 48,393 more chars]
